# Supplementary material for: Enantioselective reductive cross-couplings to forge C(sp2)–C(sp3) bonds by merging electrochemistry with nickel catalysis
Source: Nat Commun. 2025 Jan 28;16:1108. doi: 10.1038/s41467-025-56377-w (PMC11775263; doi:10.1038/s41467-025-56377-w)
Supplement: Supplementary file 1 — Supplementary Information [file 41467_2025_56377_MOESM1_ESM.pdf]

Supplementary Information

Enantioselective Reductive Cross-Couplings to Forge C(sp^2)–C(sp^3) Bonds by Merging Electrochemistry with Nickel Catalysis

Yun-Zhao Wang,[1] Bing Sun,[1] Jian-Feng Guo,[1] Xiao-Yu Zhu,[1] Yu-Cheng Gu,[2] Ya-Ping Han,[3] Cong Ma,[1] Tian-Sheng Mei[1,*]

[1]State Key Laboratory of Organometallic Chemistry, Shanghai Institute of Organic Chemistry, University of Chinese Academy of Sciences, Chinese Academy of Sciences, 345 Lingling Road, Shanghai 200032, P. R. China.
[2]Syngenta, Jealott's Hill International Research Centre, Berkshire RE42 6EY, U.K.
[3]School of Chemical Engineering and Technology, Hebei University of Technology, Tianjin 300130, China.

Table of Contents

1. Supplementary Notes S3

2. Supplementary Discussion S4

2.1 Cyclic Voltammetry Studies S4

2.2 Optimization Details S8

2.3 Large-Scale Synthesis and Mechanistic Studies S15

2.4 Synthetic Procedures and Characterization of Products S21

3. Supplementary Figures S97

3.1. X-Ray Crystal Structures...S97

3.2 ^{1}H NMR, ^{13}C NMR, ^{19}F NMR...S99

4. References S154

1. Supplementary Notes

Commercially available materials were used without further purification. Column chromatography was performed using either 100−200 Mesh or 300−400 Mesh silica gel. Visualization of spots on the LC plate was accomplished with UV light (254 nm) and staining over I_2 chamber.

All commercial reagents were purchased from TCI, Sigma-Aldrich, Adamas-beta, J&K, Bidepharm, Leyan, 9-Ding chemistry, and Energy Chemical of the highest purity grade. They were used without further purification unless specified. Nickel (II) bromide ethylene glycol dimethyl ether (≥97%) was purchased from Bidepharm. Manganese (99.8%) was purchased from Adamas-beta and was used as received. ^{1}H NMR and ^{13}C NMR spectra were recorded on Agilent AV 400, and Varian Inova 400 (400 MHz and 100 MHz, respectively). ^{19}F NMR spectra were recorded on Agilent AV 400, Varian Inova 400 (376 MHz) instrument. The peaks were internally referenced to TMS (0.00 ppm) or residual undeuterated solvent signal. The following abbreviations were used to explain multiplicities: s = singlet, d = doublet, t = triplet, q = quartet, m = multiplet, and br = broad. Infrared spectra were obtained on a Bio-Rad FTS-185 instrument High-resolution mass spectra were recorded at the Center for Mass Spectrometry, Shanghai Institute of Organic Chemistry. Analytical and spectral data of all those known compounds are exactly matching with the reported values. All air-and moisture-sensitive reactions were performed under an atmosphere of nitrogen-flamed dried glassware.

2. Supplementary Discussion

2.1 Cyclic Voltammetry Studies

All the voltammetric experiments were recorded with a CHI660E potentiostat at room temperature in Acetone. $^nBu_4NPF_6$ (0.1 M) was used as the supporting electrolyte, a Glass Carbon electrode and a platinum wire were used as working and counter electrodes, respectively. The working electrode potentials were measured versus $Ag/AgNO_3$ reference electrode (internal solution, 0.1 M $AgNO_3$ in DMAc:THF = 1:45). The redox potential of ferrocene/ferrocenium (Fc/Fc^+) was measured (same experimental conditions) and used to provide an internal reference. The potential values were then adjusted relative to Fc/Fc^+, and electrochemical studies in organic solvents were recorded accordingly. The scan rate was 0.1mV s^{-1}.

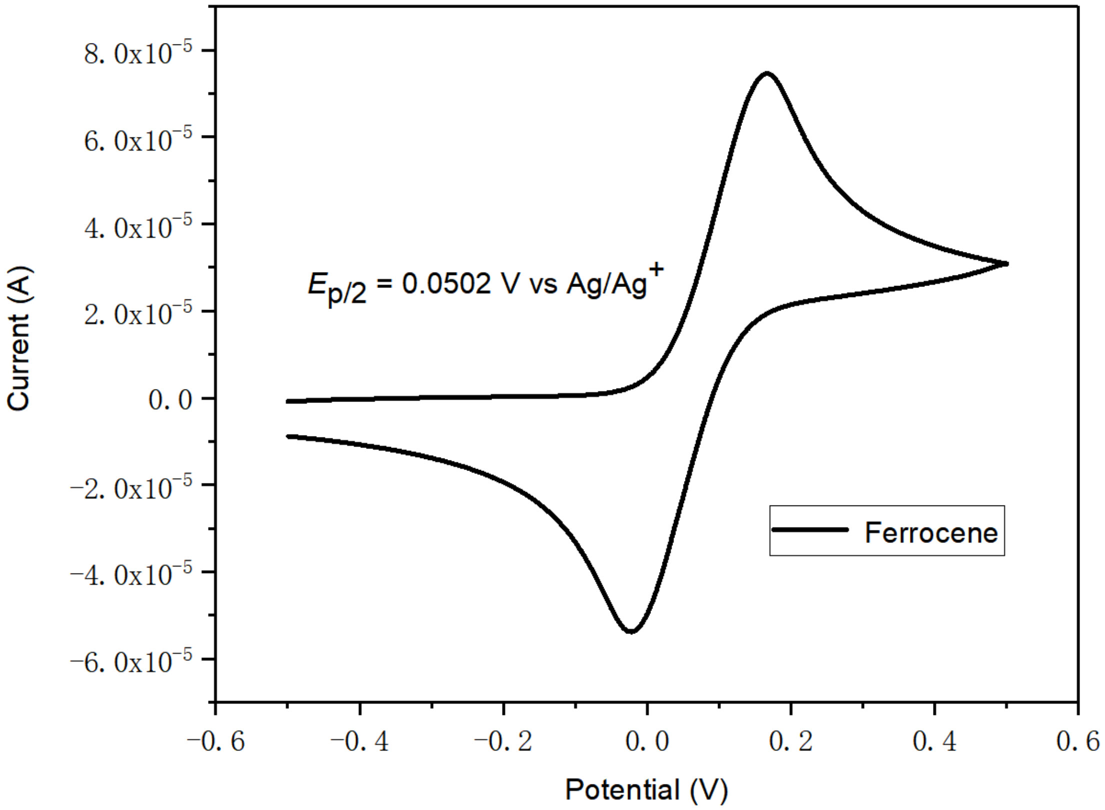

Supplementary Fig. S1 Cyclic voltammograms of 5 mM Ferrocene, DMAc:THF = 1:45 solvent, 0.1 M $^nBu_4NPF_6$ supporting electrolyte, GC working electrode, 100 mV/s scan rate.

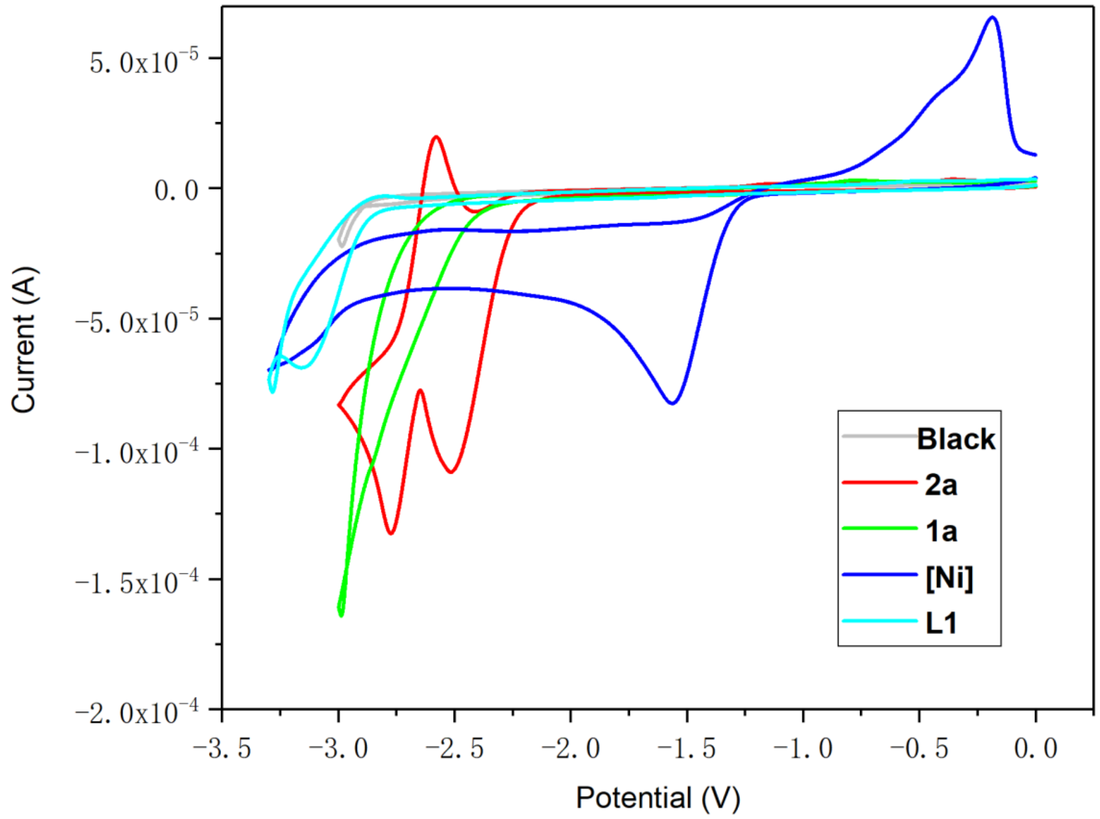

Supplementary Fig. S2 Cyclic voltammograms of black (grey line), 5 mM **2a** (red line), 5 mM **1a** (green line), 5 mM NiBr$_2$•glyme (blue line), 5 mM **L1** (cyan line), DMAc:THF = 1:45 solvent, 0.1M ^{n}Bu$_4$NPF$_6$ supporting electrolyte, GC working electrode, 100 mV/s scan rate.

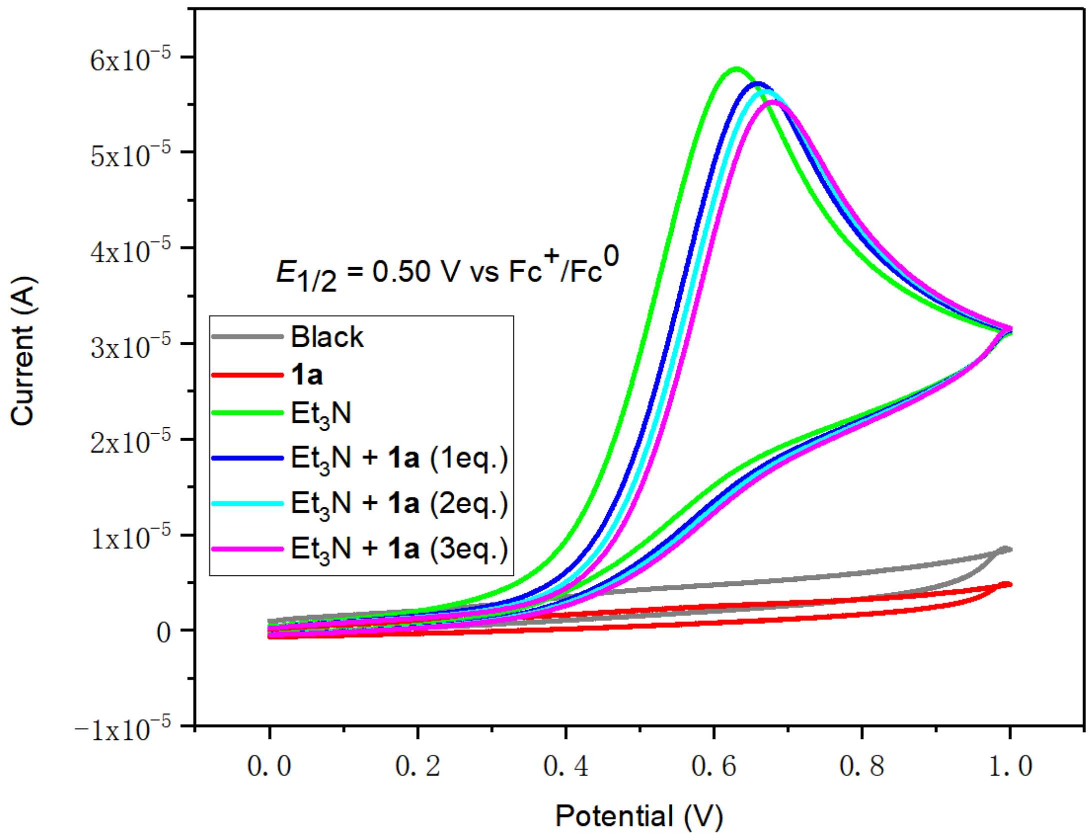

Supplementary Fig. S3 Cyclic voltammograms of black (grey line); 5 mM **1a** (red line); 5 mM Et_3N (green line); 5 mM Et_3N and 5 mM **1a** (blue line); 5 mM Et_3N and 10 mM **1a** (cyan line); 5 mM Et_3N and 15 mM **1a** (purple line); DMAc:THF = 1:45 solvent, 0.1M $^{n}Bu_4NPF_6$ supporting electrolyte, GC working electrode, 100 mV/s scan rate.

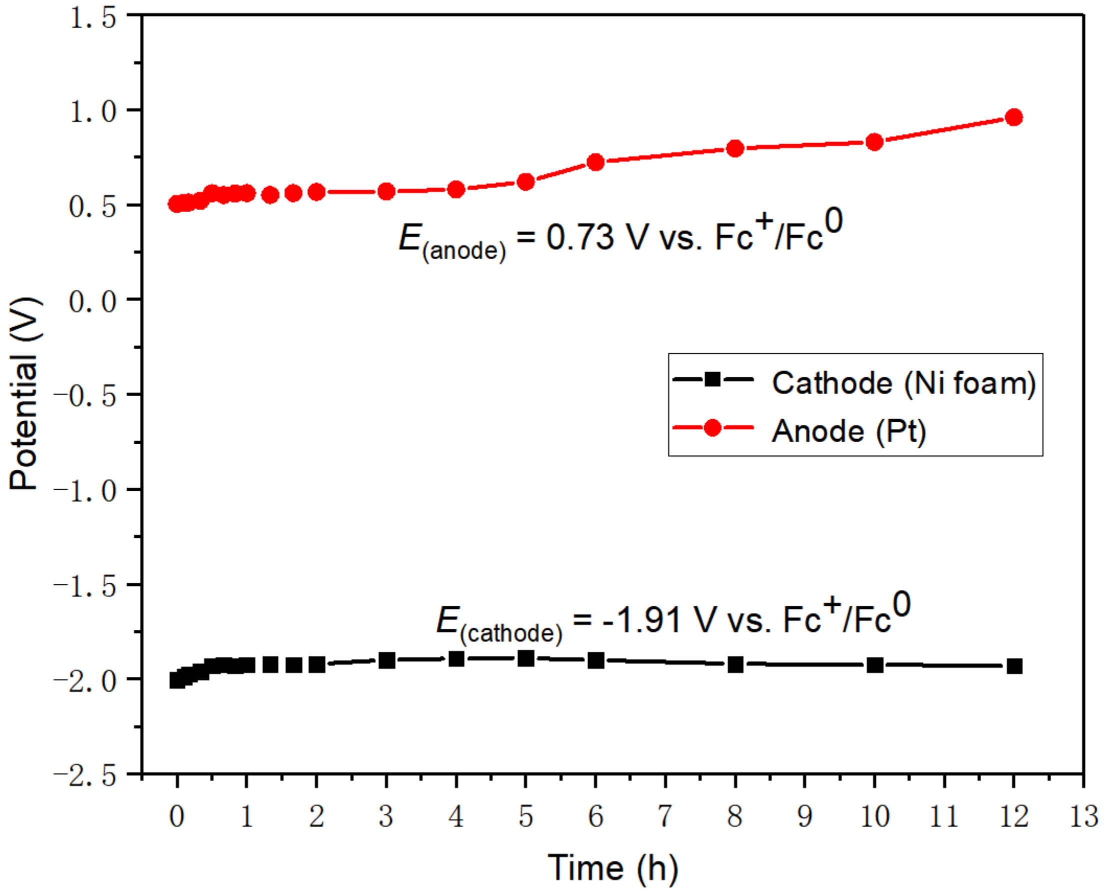

Supplementary Fig. S4 Potential profiles of Pt cathode and Ni foam anode during the electrolysis.

2.2 Optimization Details

Supplementary Table S1. Screening of Biox ligands[a,b,c]

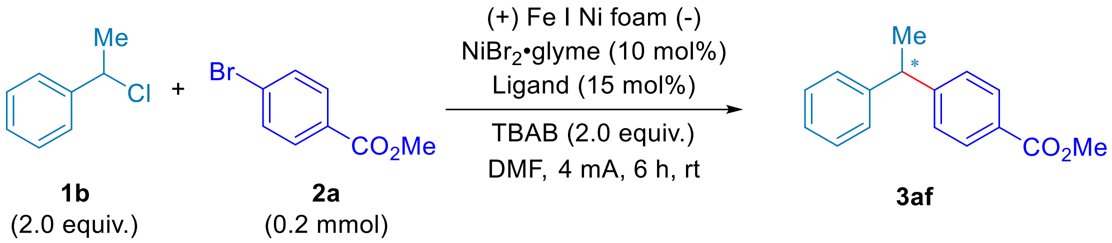

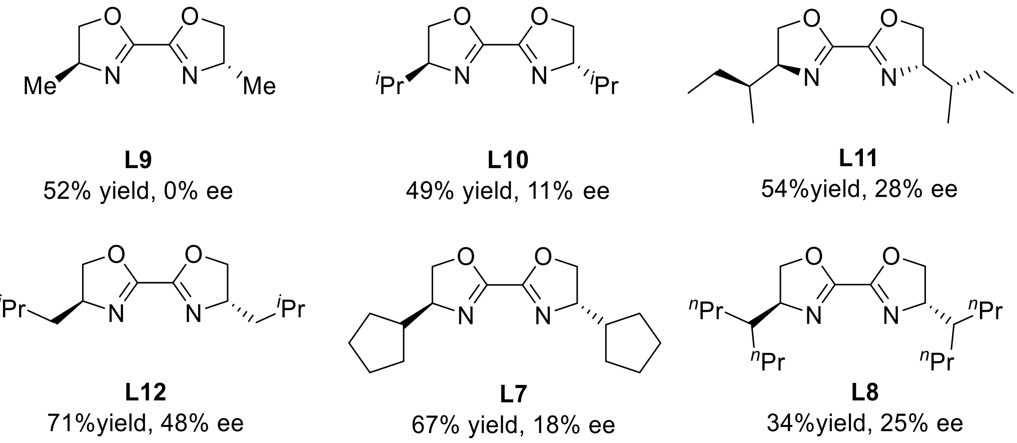

[a]Reactions were carried out with **1b** (2.0 equiv.), **2a** (0.2 mmol), $NiBr_2$•glyme (10 mol %), Ligand (15 mol %), TBAB (2.0 equiv.), DMF (2 mL), Iron (0.5 x 1.0 cm^2) as the anode. Ni form (1.0 x 2.5 cm^2) as the cathode, 4 mA, Room temperature, 6 h. [b]The yields were determined by ^1H NMR using CH_2Br_2 as an internal standard. [c]The ee values were determined by HPLC on a chiral stationary phase.

Supplementary Table S2. Screening of chiral ligand[a,b,c]

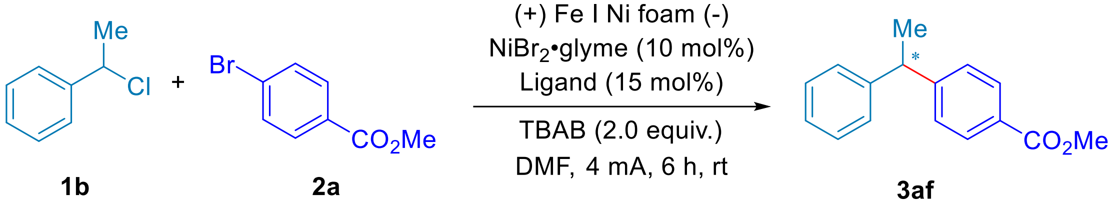

1b (2.0 equiv.) + **2a** (0.2 mmol) → **3af**

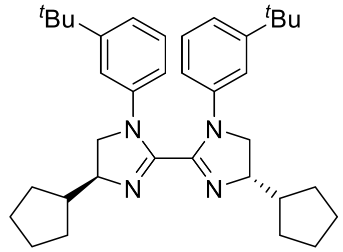

L1
95% yield, 78% ee

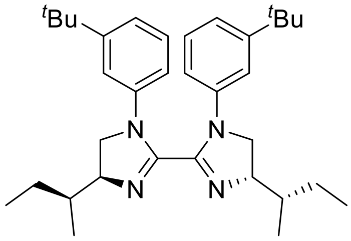

L2
54% yield, 72% ee

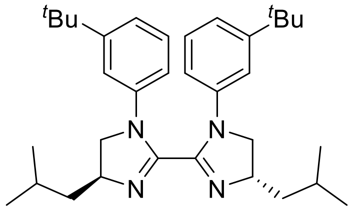

L3
45% yield, 55% ee

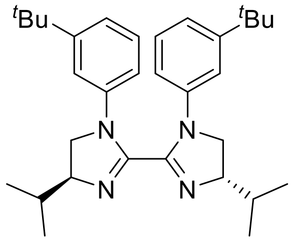

L4
72% yield, 71% ee

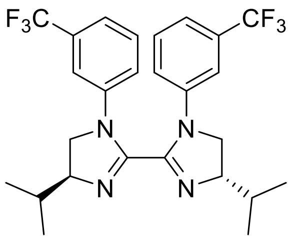

L5
51% yield, 64% ee

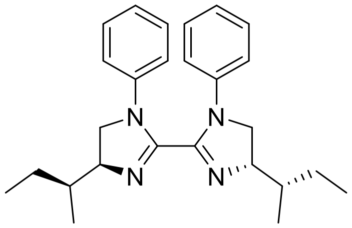

L13
54% yield, 71% ee

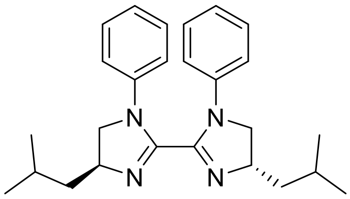

L14
44% yield, 53% ee

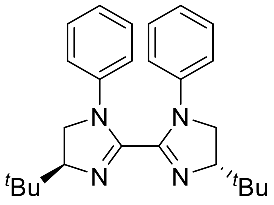

L15
39% yield, 2% ee

[a]Reactions were carried out with **1b** (2.0 equiv.), **2a** (0.2 mmol), $NiBr_2$•glyme (10 mol %), Ligand (15 mol %), TBAB (2.0 equiv.), DMF (2 mL), Iron (0.5 x 1.0 cm^2) as the anode. Ni form (1.0 x 2.5 cm^2) as the cathode, 4 mA, Room temperature, 6 h. [b]The yields were determined by 1H NMR using CH_2Br_2 as an internal standard. [c]The ee values were determined by HPLC on a chiral stationary phase.

Supplementary Table S3. Screening of catalyst[a]

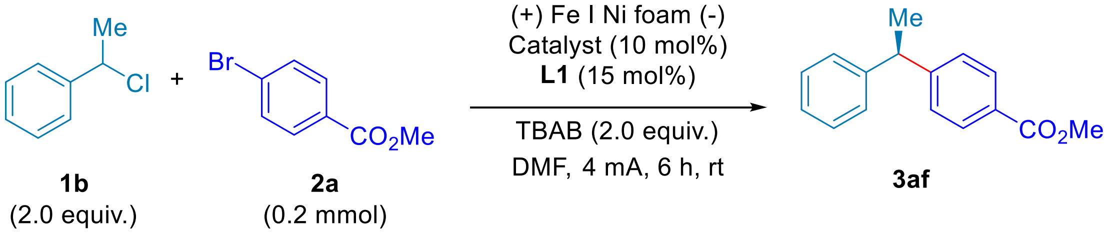

Entry	Catalyst	NMR yield (%)[b]	ee (%)[c]
1	$NiBr_2$•glyme	95	78
2	$NiCl_2$•glyme	72	74
3	NiI_2	65	76
4	$Ni(cod)_2$	90	77
5	CoI_2	32	71

[a]Reactions were carried out with **1b** (2.0 equiv.), **2a** (0.2 mmol), Catalyst (10 mol %), **L1** (15 mol %), TBAB (2.0 equiv.), DMF (2 mL), Iron (0.5 x 1.0 cm^2) as the anode. Ni form (1.0 x 2.5 cm^2) as the cathode, 4 mA, Room temperature, 6 h. [b]The yields were determined by 1H NMR using CH_2Br_2 as an internal standard. [c]The ee values were determined by HPLC on a chiral stationary phase.

Supplementary Table S4. Screening of solvent[a]

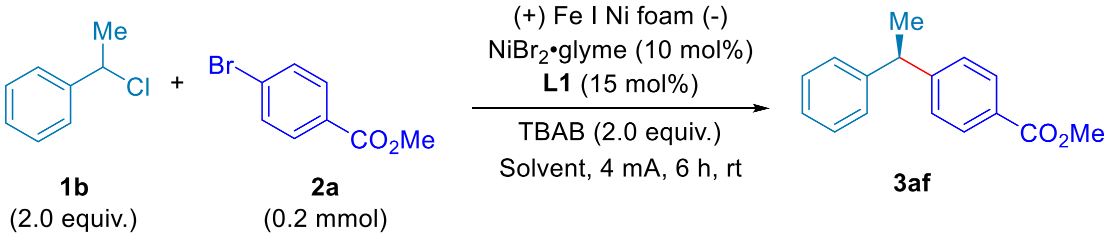

Entry	Solvent	NMR yield (%)[b]	ee (%)[c]
1	DMF	95	78
2	DMF:THF = 1:1	79	81
3	DMF:THF = 1:2	92	76

[a]Reactions were carried out with **1b** (2.0 equiv.), **2a** (0.2 mmol), $NiBr_2$•glyme (10 mol %), **L1** (15 mol %), TBAB (2.0 equiv.), Solvent (2 mL), Iron (0.5 x 1.0 cm^2) as the anode. Ni form (1.0 x 2.5 cm^2) as the cathode, 4 mA, Room temperature, 6 h. [b]Yields were determined by 1H NMR using CH_2Br_2 as an internal standard. [c]The ee values were determined by HPLC on a chiral stationary phase.

Supplementary Table S5. Screening of sacrificial agent[a]

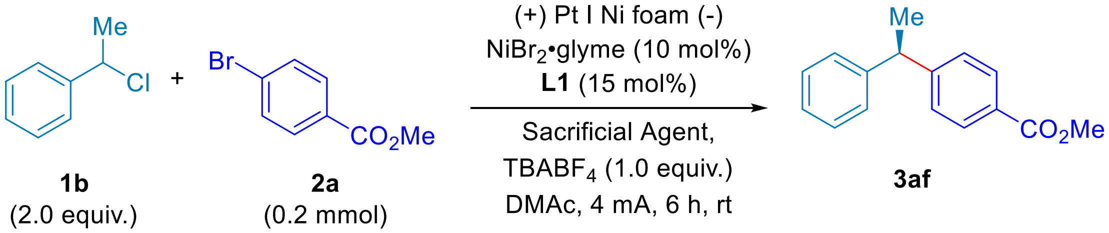

Entry	Sacrificial Agent	NMR yield (%)[b]	ee (%)[c]
1	TTMSS (3 eq.) + 2,6-lutidine (3 eq.)	50	78
2	TTMSS (3 eq.) + DBU (3 eq.)	trace	-
3	TTMSS (3 eq.) + Cs_2CO_3 (3 eq.)	19	74
4	TTMSS (3 eq.) + Et_3N (3 eq.)	60	80
5	Et_3N (3 equiv.)	52	79
6	Et_3N (1.5 equiv.)	31	80
**7**	**Et_3N (4.5 equiv.)**	**76**	**82**
8	Et_3N (6 equiv.)	64	80
9	Et_3N (7.5 equiv.)	45	80

[a]Reactions were carried out with **1b** (2.0 equiv.), **2a** (0.2 mmol), $NiBr_2$•glyme (10 mol %), **L1** (15 mol %), Sacrificial agent, $TBABF_4$ (1 equiv.), DMAc (2 mL), Pt (1.0 x 1.0 cm^2) as the anode. Ni form (1.0 x 2.5 cm^2) as the cathode, 4 mA, Room temperature, 6 h. [b]The yields were determined by 1H NMR using CH_2Br_2 as an internal standard. [c]The ee values were determined by HPLC on a chiral stationary phase.

Supplementary Table S6. Screening of solvent[a]

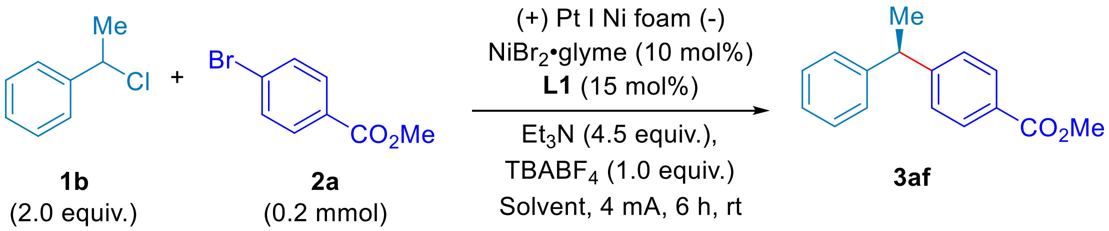

Entry	Solvent	NMR yield (%)[b]	ee (%)[c]
**1**	**DMAc:THF = 1:1**	**57**	**83**
2	DMAc:2-MTHF = 1:1	28	83
3	DMAc:Dioxane = 1:1	37	83

4	DMAc:MTBE = 1:1	40	83
5	DMAc:Toluene = 1:1	4	83
6	DMF:THF = 1:1	16	81

[a]Reactions were carried out with **1b** (2.0 equiv.), **2a** (0.2 mmol), $NiBr_2$•glyme (10 mol %), **L1** (15 mol %), Et_3N (4.5 equiv.), $TBABF_4$ (1 equiv.), Solvent (2 mL), Pt (1.0 x 1.0 cm^2) as the anode. Ni form (1.0 x 2.5 cm^2) as the cathode, 4 mA, Room temperature, 6 h. [b]The yields were determined by 1H NMR using CH_2Br_2 as an internal standard. [c]The ee values were determined by HPLC on a chiral stationary phase.

Supplementary Table S7. Increase substrate steric hindrance---methyl to ethyl[a]

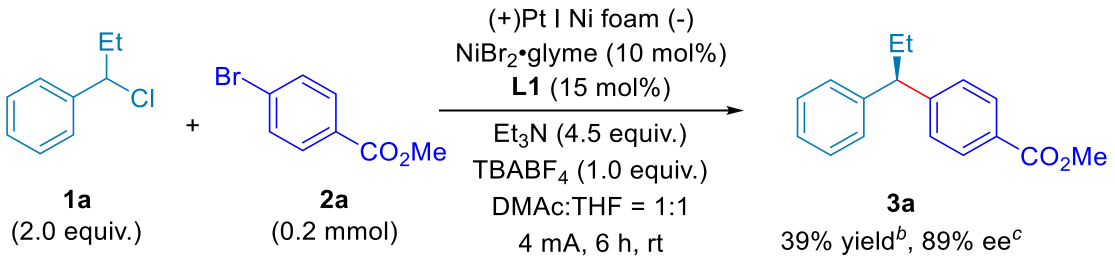

[a]Reactions were carried out with **1a** (2.0 equiv.), **2a** (0.2 mmol), $NiBr_2$•glyme (10 mol %), **L1** (15 mol %), Et_3N (4.5 equiv.), $TBABF_4$ (1 equiv.), DMAc:THF = 1:1 (2 mL), Pt (1.0 x 1.0 cm^2) as the anode. Ni form (1.0 x 2.5 cm^2) as the cathode, 4 mA, Room temperature, 6 h. [b]The yields were determined by 1H NMR using CH_2Br_2 as an internal standard. [c]The ee values were determined by HPLC on a chiral stationary phase.

Supplementary Table S8. Add 4 Å molecular sieves[a]

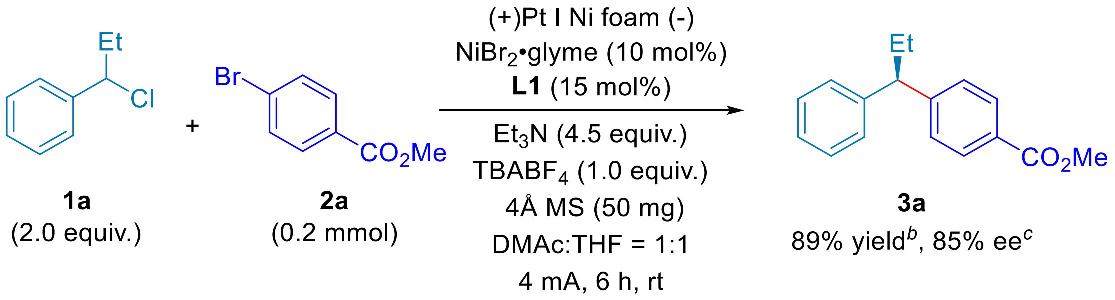

[a]Reactions were carried out with **1a** (2.0 equiv.), **2a** (0.2 mmol), $NiBr_2$•glyme (10 mol %), **L1** (15 mol %), Et_3N (4.5 equiv.), $TBABF_4$ (1 equiv.), 4Å MS (50 mg), DMAc:THF = 1:1 (2 mL), Pt (1.0 x 1.0 cm^2) as the anode. Ni form (1.0 x 2.5 cm^2) as the cathode, 4 mA, Room temperature, 6 h. [b]The yields were determined by 1H NMR using CH_2Br_2 as an internal standard. [c]The ee values were determined by HPLC on a chiral stationary phase.

Supplementary Table S9. Screening of solvent[a]

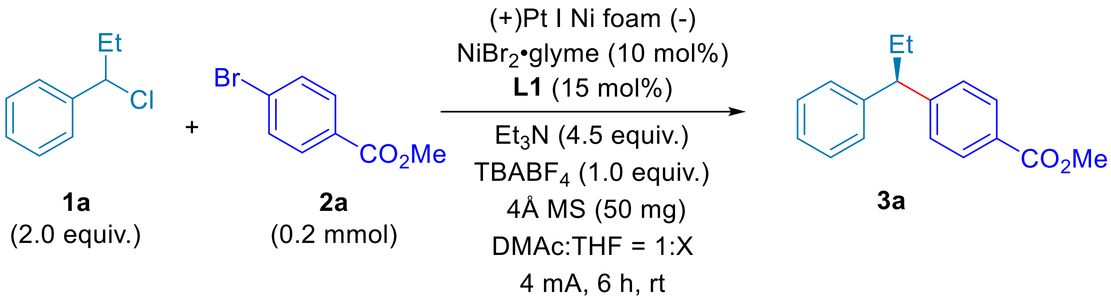

Entry	Solvent	NMR yield (%)[b]	ee (%)[c]
1	DMAc:THF = 1:1	89	85
2	DMAc:THF = 1:5	50	89
3	DMAc:THF = 1:10	20	89
4	DMAc:THF = 1:20	19	89
5	DMAc:THF = 1:30	16	89
6	DMAc:THF = 1:45	0	-
**7**	**DMAc:THF = 1:45[d]**	**89**	**90**

[a]Reactions were carried out with **1a** (2.0 equiv.), **2a** (0.2 mmol), $NiBr_2$•glyme (10 mol %), **L1** (15 mol %), Et_3N (4.5 equiv.), $TBABF_4$ (1 equiv.), 4Å MS (50 mg), Solvent (2 mL), Pt (1.0 x 1.0 cm^2) as the anode. Ni form (1.0 x 2.5 cm^2) as the cathode, 4 mA, Room temperature, 6 h. [b]Yields were determined by 1H NMR using CH_2Br_2 as an internal standard. [c]The ee values were determined by HPLC on a chiral stationary phase. [d]2 mA, Room temperature, 12 h.

Supplementary Table S10. Screening of anode[a]

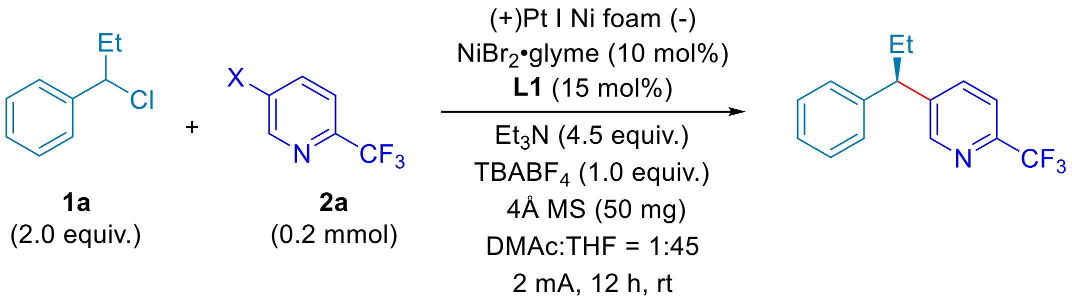

Entry	Solvent	NMR yield (%) [b]	ee (%) [c]
1 (X = Cl)	none	48	36
2 (X = Cl)	Anode Fe instead of Pt [d]	25	35

3 (X = Cl)	Anode Zn instead of Pt [d]	13	28
4 (X = Cl)	Anode Mg instead of Pt [d]	<5	29
4 (X = Br)	none	0	-
4 (X = Br)	Anode Fe instead of Pt [d]	0	-

[a]Reactions were carried out with **1a** (2.0 equiv.), **2a** (0.2 mmol), $NiBr_2$•glyme (10 mol %), **L1** (15 mol %), Et_3N (4.5 equiv.), $TBABF_4$ (1 equiv.), 4Å MS (50 mg), DMAc:THF = 1:45 (2 mL), Pt (1.0 x 1.0 cm^2) as the anode. Ni form (1.0 x 2.5 cm^2) as the cathode, 4 mA, Room temperature, 6 h. [b]The yields were determined by 1H NMR using CH_2Br_2 as an internal standard. [c]The ee values were determined by HPLC on a chiral stationary phase. [d] Without Et_3N.

Supplementary Table S11. Screening of additives[a]

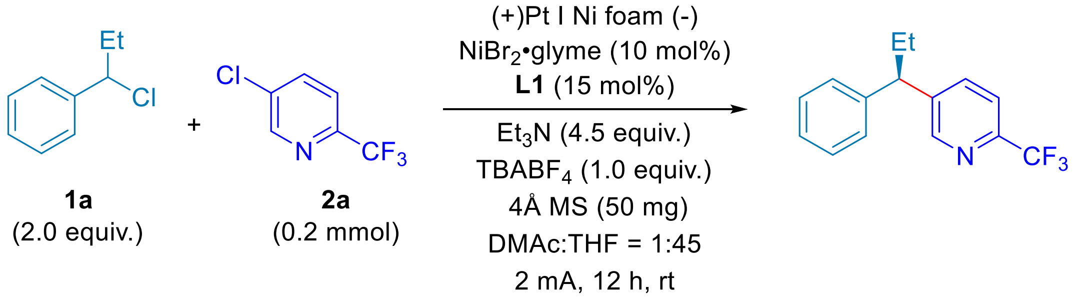

Entry	Additives	NMR yield (%)[b]	ee (%)[c]
1	none	48	36
2	$ZnBr_2$[d]	<5	20
3	$FeBr_2$[d]	0	-
4	$MgBr_2$[d]	0	-

[a]Reactions were carried out with **1a** (2.0 equiv.), **2a** (0.2 mmol), $NiBr_2$•glyme (10 mol %), **L1** (15 mol %), Et_3N (4.5 equiv.), $TBABF_4$ (1 equiv.), 4Å MS (50 mg), DMAc:THF = 1:45 (2 mL), Pt (1.0 x 1.0 cm^2) as the anode. Ni form (1.0 x 2.5 cm^2) as the cathode, 4 mA, Room temperature, 6 h. [b]The yields were determined by 1H NMR using CH_2Br_2 as an internal standard. [c]The ee values were determined by HPLC on a chiral stationary phase. [d]3 equiv.

2.3 Large-Scale Synthesis and Mechanistic Studies

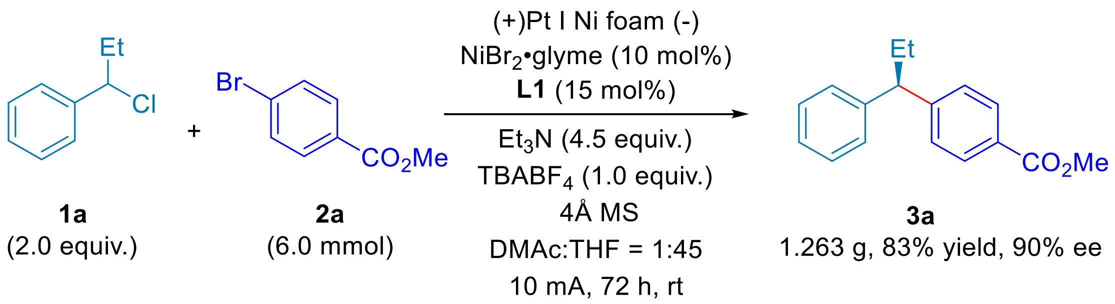

Supplementary Fig. S5. Large-scale synthesis of **3a**

In Glovebox, an oven-dried electrochemical cell with a stir bar was charged with **1a** (12 mmol, 2 equiv.) and **2a** (6 mmol, 1 equiv.), $NiBr_2$·glyme (0.6 mmol, 10 mol%), **L1** (0.9 mmol, 15 mol%), Et_3N (27 mmol, 4.5 equiv.), $TBABF_4$ (6 mmol, 1 equiv.), 4Å MS (3 g), 60 mL of DMAc:THF = 1:45. The tube was installed an Ni foam as the cathode and Pt as the anode. The mixture was stirred at room temperature for 30 min. The reaction mixture was electrolyzed under the 10 mA at RT until the complete consumption of the starting materials as monitored by TLC (72 hours). The resulting mixture was filtered with silica gel short columns, and the crude product was purified by automated silica gel column chromatography (EtOAc/hexanes). Affording the desired product **3a** in 83% (1.263 g) isolated yield and 90% ee.

Supplementary Table S12. Control electrode potential experiments[a]

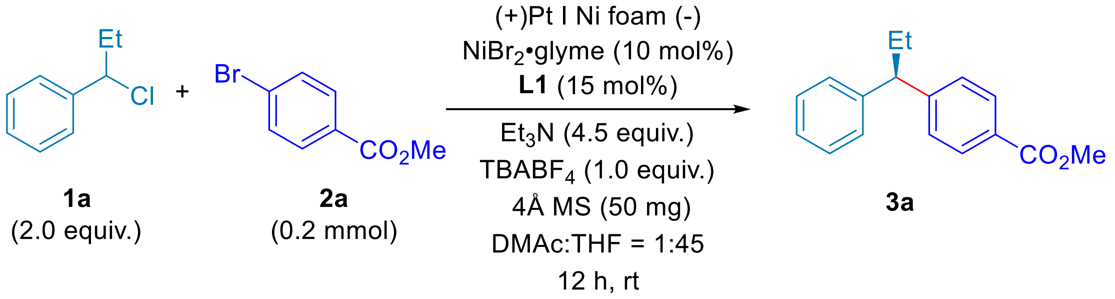

Entry	Constant Potential (V)	NMR yield (%)[b]	ee (%)[c]
1	-1.2	0	-
2	-2.0	90	89

[a]Reactions were carried out with **1a** (2.0 equiv.), **2a** (0.2 mmol), $NiBr_2$•glyme (10 mol %), **L1** (15 mol %), Et_3N (4.5 equiv.), $TBABF_4$ (1 equiv.), 4Å MS (50 mg), DMAc:THF = 1:45 (2 mL), Pt (1.0

x 1.0 cm^2) as the anode. Ni form (1.0 x 2.5 cm^2) as the cathode, rt, 12 h. [b]Yields were determined by 1H NMR using CH_2Br_2 as an internal standard. [c]The ee values were determined by HPLC on a chiral stationary phase.

Supplementary Table S13. Free radical capture experiments[a]

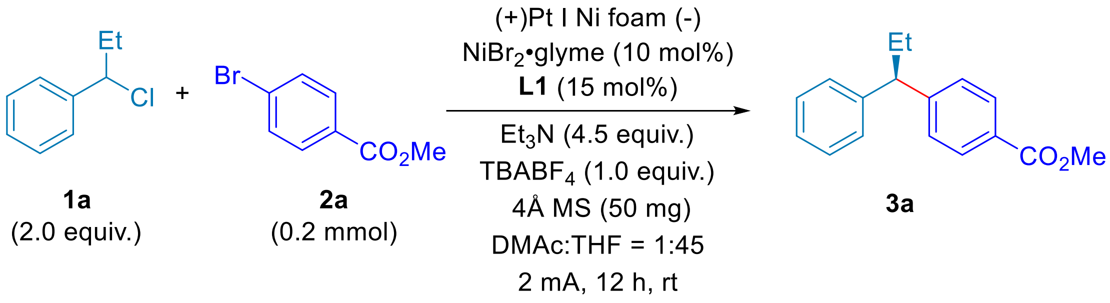

Entry	Radical trap	NMR yield (%)[b]	ee (%)[c]
1	TEMPO	trace	-
2	BHT	trace	-

[a]Reactions were carried out with **1a** (2.0 equiv.), **2a** (0.2 mmol), $NiBr_2$•glyme (10 mol %), **L1** (15 mol %), Et_3N (4.5 equiv.), $TBABF_4$ (1 equiv.), 4Å MS (50 mg), DMAc:THF = 1:45 (2 mL), Radical trap (3.0 equiv.), Pt (1.0 x 1.0 cm^2) as the anode. Ni form (1.0 x 2.5 cm^2) as the cathode, rt, 2 mA, 12 h. [b]Yields were determined by 1H NMR using CH_2Br_2 as an internal standard. [c]The ee values were determined by HPLC on a chiral stationary phase.

Supplementary Table S14. Different reduction conditions (Bromobenzene)

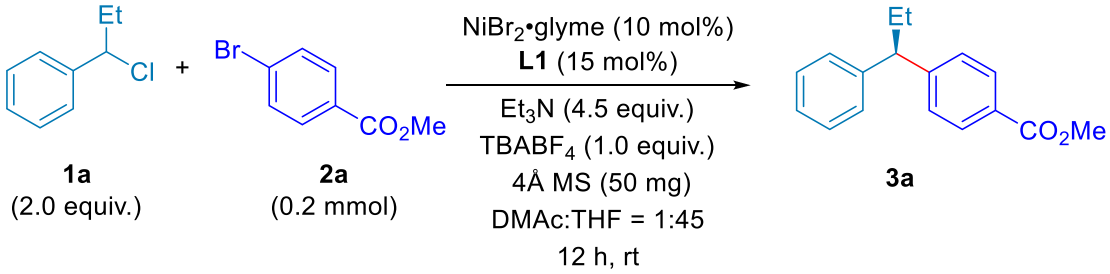

Entry	Reducing reagents	NMR yield (%)[b]	ee (%)[c]
1	Electrochemisty (Standard Conditions)	89	90
2	Mn[a]	36	88
3	Zn[a]	trace	-

4	TDAE[a]	trace	-

[a]Reactions were carried out with **1a** (2.0 equiv.), **2a** (0.2 mmol), $NiBr_2$•glyme (10 mol %), **L1** (15 mol %), Et_3N (4.5 equiv.), $TBABF_4$ (1 equiv.), 4Å MS (50 mg), DMAc:THF = 1:45 (2 mL), [Mn or Zn (3.0 equiv.), TMSCl (50 mol%)] or TDAE (3.0 equiv.), rt, 12 h. [b]The yields were determined by 1H NMR using CH_2Br_2 as an internal standard. [c]The ee values were determined by HPLC on a chiral stationary phase.

Supplementary Table S15. Different reduction conditions (Chlorobenzene)

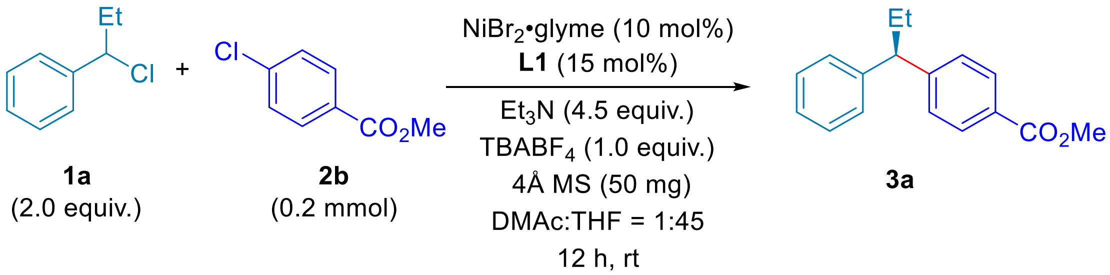

Entry	Reducing reagents	NMR yield (%)[b]	ee (%)[c]
1	Electrochemistry (Standard Conditions)	75	87
2	Mn (12 h)[a]	trace	-
3	Zn (12 h)[a]	trace	-
4	TDAE (12 h)[a]	trace	-
5	Mn (Ref. Resiman's report)	10	-
6	Photochemistry (Ref. Lu's report)	<5	-

[a]Reactions were carried out with **1a** (2.0 equiv.), **2b** (0.2 mmol), $NiBr_2$•glyme (10 mol %), **L1** (15 mol %), Et_3N (4.5 equiv.), $TBABF_4$ (1 equiv.), 4Å MS (50 mg), DMAc:THF = 1:45 (2 mL), [Mn or Zn (3.0 equiv.), TMSCl (50 mol%)] or TDAE (3.0 equiv.), Room temperature. [b]Yields were determined by 1H NMR using CH_2Br_2 as an internal standard. [c]The ee values were determined by HPLC on a chiral stationary phase. **Entry 5**: **1a** (1.2 equiv.), **2b** (0.2 mmol), $NiBr_2$•glyme (10 mol %), **L1** (20 mol %), Mn (3.0 equiv.), TMSCl (0.75 equiv.), 1,4-dioxane, rt, 18 h. **Entry 6**: **1a** (0.2 mmol), **2b** (2.0 equiv.), $NiCl_2$•glyme (10 mol %), **L1** (20 mol %), $[Ir(dFCF_3ppy)_2(dtbbpy)]Cl$ (2 mol%), HEH (1.5 equiv.), dioxane 6 W blue LEDs, 24 h

Supplementary Table S16. Different reduction conditions (Aryl triflate)[a]

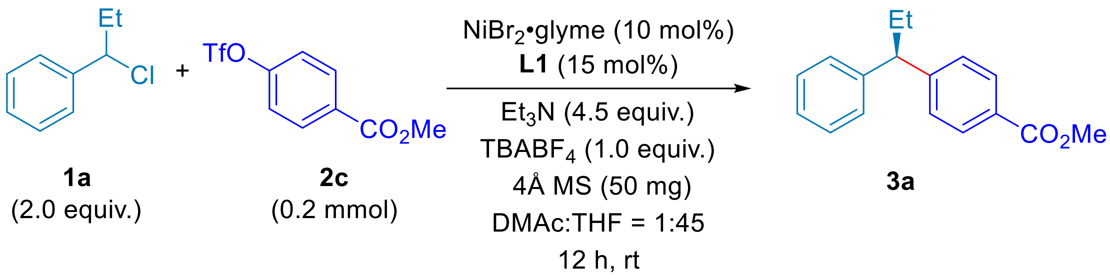

1a (2.0 equiv.) **2c** (0.2 mmol) **3a**

Entry	Reducing reagents	NMR yield (%)[b]	ee (%)[c]
1	Electrochemistry (Standard Conditions)	67	82
2	Mn (Ref. Resiman' report)	0	-
3	Photochemistry (Ref. Lu' report)	0	-

[a]Reactions were carried out with **Entry 2**: **1a** (1.2 equiv.), **2b** (0.2 mmol), NiBr$_2$•glyme (10 mol %), **L1** (20 mol %), Mn (3.0 equiv.), TMSCl (0.75 equiv.), 1,4-dioxane, rt, 18 h. **Entry 3**: **1a** (0.2 mmol), **2b** (2.0 equiv.), NiCl$_2$•glyme (10 mol %), **L1** (20 mol %), [Ir(dFCF$_3$ppy)$_2$(dtbbpy)]Cl (2 mol%), HEH (1.5 equiv.), dioxane 6 W blue LEDs, 24 h. [b]Yields were determined by 1H NMR using CH_2Br_2 as an internal standard. [c]The ee values were determined by HPLC on a chiral stationary phase.

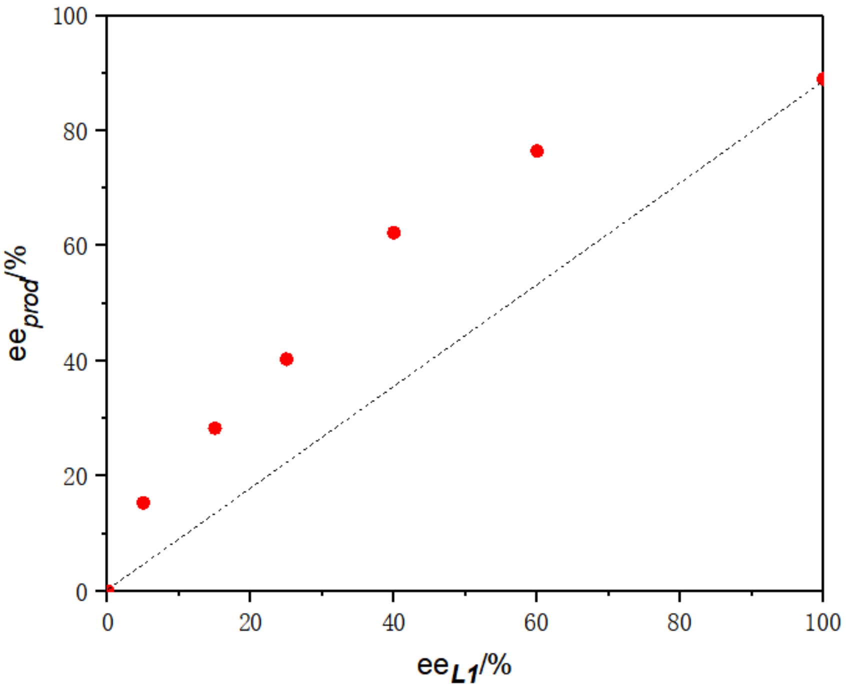

Supplementary Fig. S6 Nonlinear effects of Ni-catalyzed eRCC reactions

Supplementary Table S17. Unsuccessful substrates

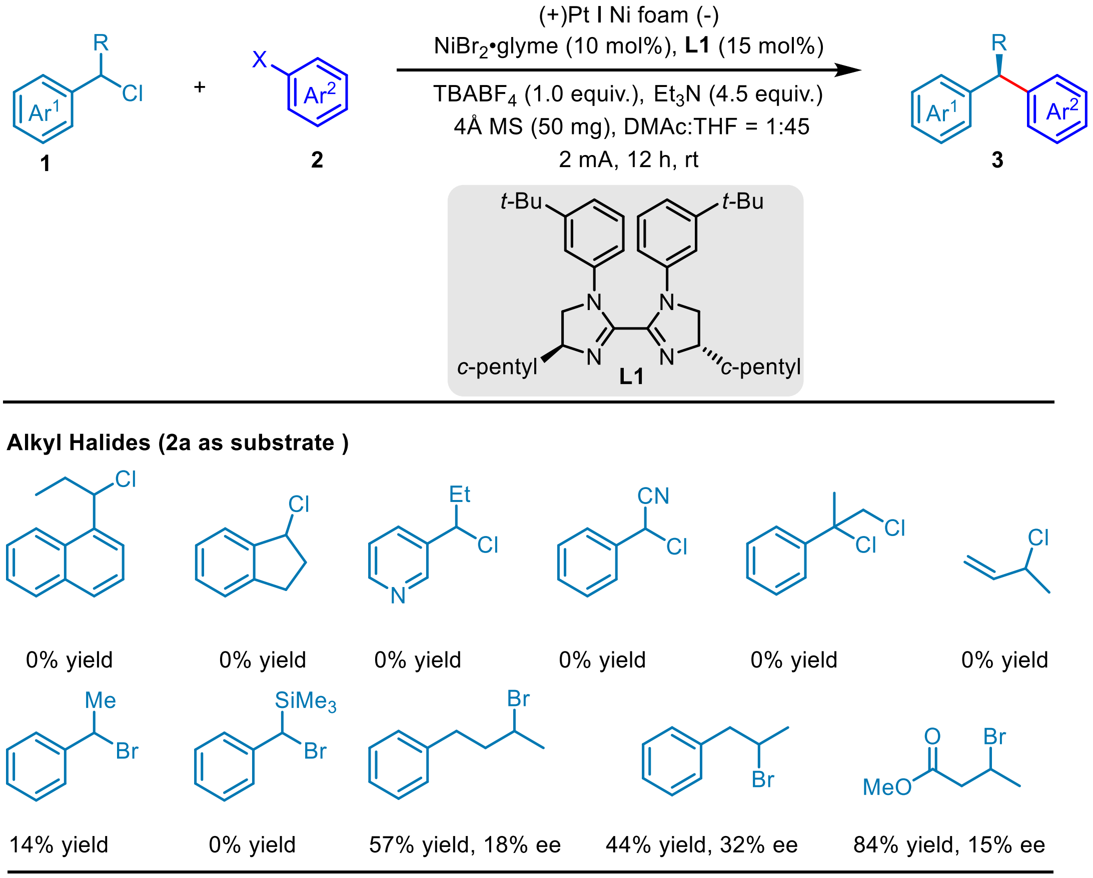

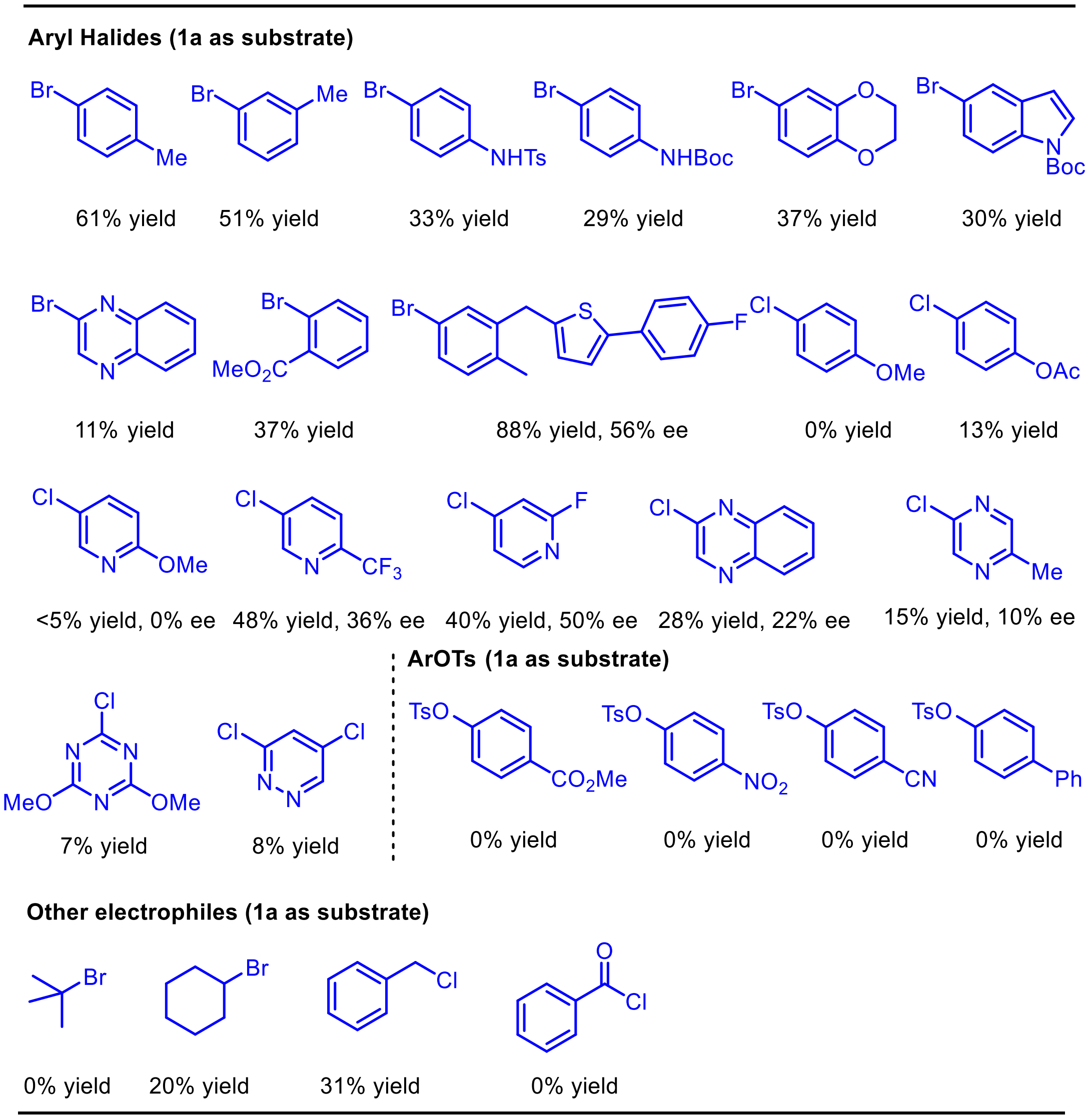
Aryl Halides (1a as substrate)
61% yield
51% yield
33% yield
29% yield
37% yield
30% yield
11% yield
37% yield
88% yield, 56% ee
0% yield
13% yield
<5% yield, 0% ee
48% yield, 36% ee
40% yield, 50% ee
28% yield, 22% ee
15% yield, 10% ee
7% yield
8% yield
ArOTs (1a as substrate)
0% yield
0% yield
0% yield
0% yield
Other electrophiles (1a as substrate)
0% yield
20% yield
31% yield
0% yield

2.4 Synthetic Procedures and Characterization of Products

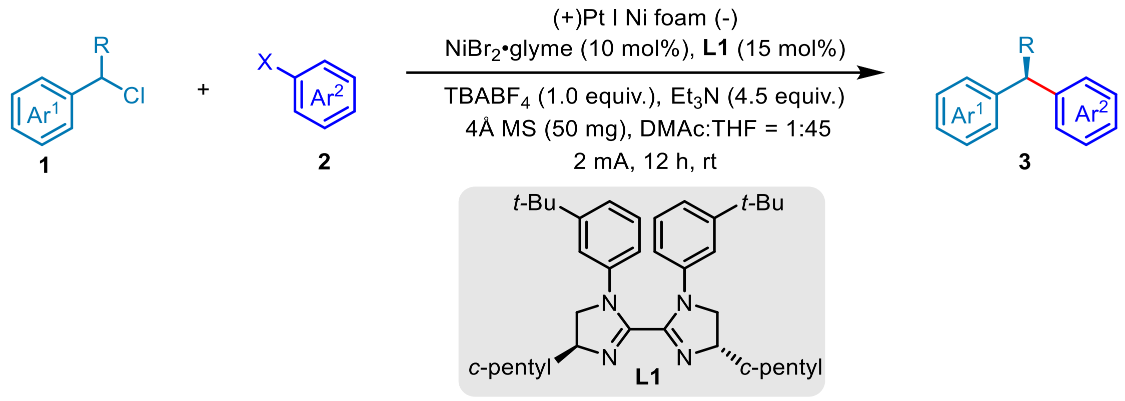

General procedure: In glovebox, an oven-dried electrochemical cell with a stir bar was charged with bromobenzene/chlorobenzene/aryl triflates (**2**, 0.2 mmol, 1 equiv.) and benzyl chloride (**1**, 0.4 mmol, 2 equiv.), $NiBr_2$·glyme (0.02 mmol, 10 mol%), ligand **L1** (0.03 mmol, 15 mol%), $TBABF_4$ (0.2 mmol, 1 equiv.), Et_3N (0.9 mmol, 4.5 equiv.), 4Å MS (50 mg), 2 mL of DMAc:THF = 1:45. The tube was installed an Ni foam as the cathode and Pt as the anode. The mixture was stirred at room temperature for 30 min. The reaction mixture was electrolyzed under a 2 mA at RT. After 12 h, EtOAc (50 mL) was added to the resulting solution, which was then washed with brine (50 mL x 3). The organic layer was dried over anhydrous Mg_2SO_4, filtered and concentrated to give the crude product. The crude product was purified by automated silica gel column chromatography (EtOAc/hexanes). Affording the desired product **3**.

Cleaning and Reuse of Pt electrode: After the reaction is finished, put the Pt electrode into a solution of concentrated hydrochloric acid: ethanol = 1:100 and ultrasonicate for 10 minutes, then wash it with ethanol and acetone and dry it, then reuse it in the next reaction.

Photographic Guide for Electrochemical coupling

Easily hand-made electrochemical cell

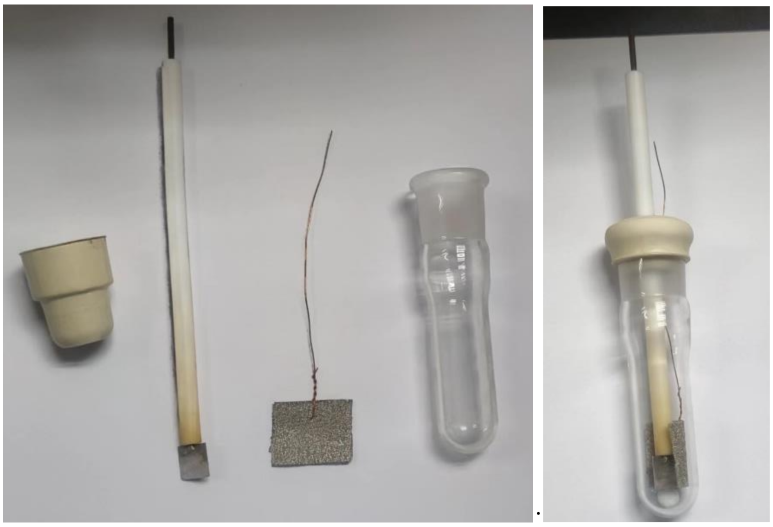

Isolated Yields and Characterization of Products

methyl (*R*)-4-(1-phenylpropyl)benzoate (3a)[1]

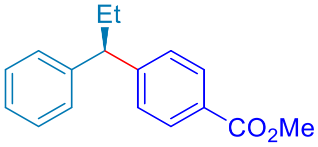

Prepared according to the general procedure **1** from (1-chloropropyl)benzene (0.4 mmol, 2 equiv.) and **2** from methyl 4-bromobenzoate (0.2 mmol, 1 equiv.). The title compound was isolated (gradient 0–5% EtOAc/hexanes) as a colorless oil (43.2 mg, 85% yield, 90% ee).

^{1}H NMR (400 MHz, $CDCl_3$) δ 7.87 (d, *J* = 8.4 Hz, 2H), 7.30 – 7.06 (m, 7H), 3.79 (s, 3H), 3.76 (t, *J* = 7.6 Hz, 1H), 2.00 (p, *J* = 7.2 Hz, 2H), 0.81 (t, *J* = 7.2 Hz, 3H).

^{13}C NMR (101 **MHz, $CDCl_3$)** δ 167.1, 150.6, 144.2, 129.8, 128.5, 128.0, 128.0, 127.9, 126.4, 53.3, 52.0, 28.4, 12.7.

$[\alpha]_D^{23}$ = -2.36 (*c* = 0.2, $CHCl_3$).

Enantiomeric excess = 90%, determined by HPLC (Daicel Chiralpak OD-H Column, *n*-Hexane:*i*-PrOH = 95:5, flow rate 0.7 mL/min, T = 25 °C, λ = 214 nm): t_R = 7.679 min (minor), t_R = 8.015 min (major).

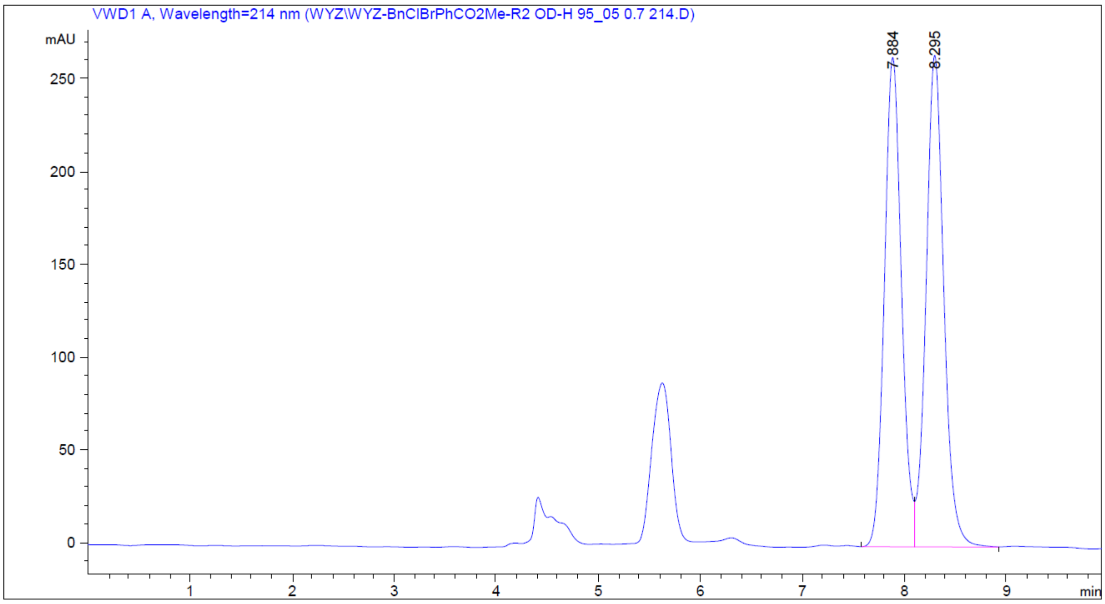

Peak NO	Ret. Time(min)	Area/%
1	7.884	48.8134
2	8.295	51.1866

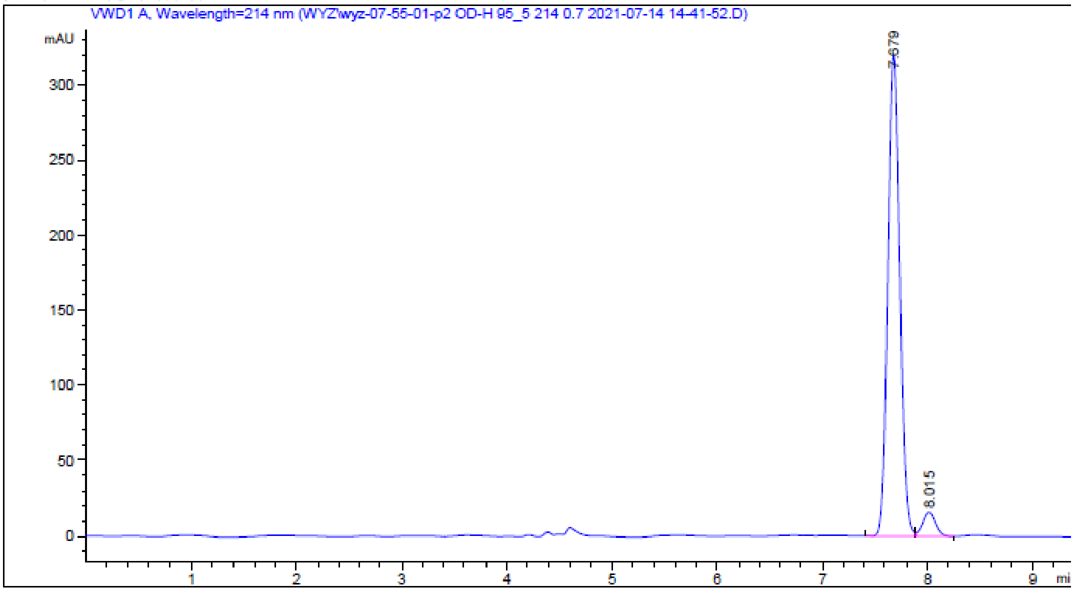

Peak NO	Ret. Time(min)	Area/%
1	7.679	94.9141
2	8.015	5.0859

methyl (*R*)-4-(1-phenylpropyl)benzoate (3a)[1]

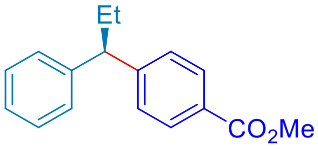

Prepared according to the general procedure **1** from (1-chloropropyl)benzene (0.4 mmol, 2 equiv.) and **2** from methyl 4-chlorobenzoate (0.2 mmol, 1 equiv.). The title compound was isolated (gradient 0–5% EtOAc/hexanes) as a colorless oil (34.5 mg, 68% yield, 87% ee).

^{1}H NMR (400 MHz, CDCl$_3$) δ 7.95 (d, *J* = 8.4 Hz, 2H), 7.36 – 7.15 (m, 7H), 3.90 – 3.81 (m, 4H), 2.17 – 1.98 (m, 2H), 0.90 (t, *J* = 7.2 Hz, 3H).

^{13}C NMR (101 **MHz, CDCl$_3$)** δ 167.1, 150.2, 144.2, 129.7, 128.5, 128.0, 127.9, 127.9, 126.3, 53.2, 52.0, 28.4, 12.7.

$[\alpha]_D$ ^{23} = -3.65 (*c* = 0.4, CHCl$_3$).

Enantiomeric excess = 87%, determined by HPLC (Daicel Chiralpak OD-H Column, *n*-Hexane:*i*-PrOH = 95:5, flow rate 0.7 mL/min, T = 25 °C, λ = 214 nm): t_R = 7.863 min (minor), t_R = 8.258 min (major).

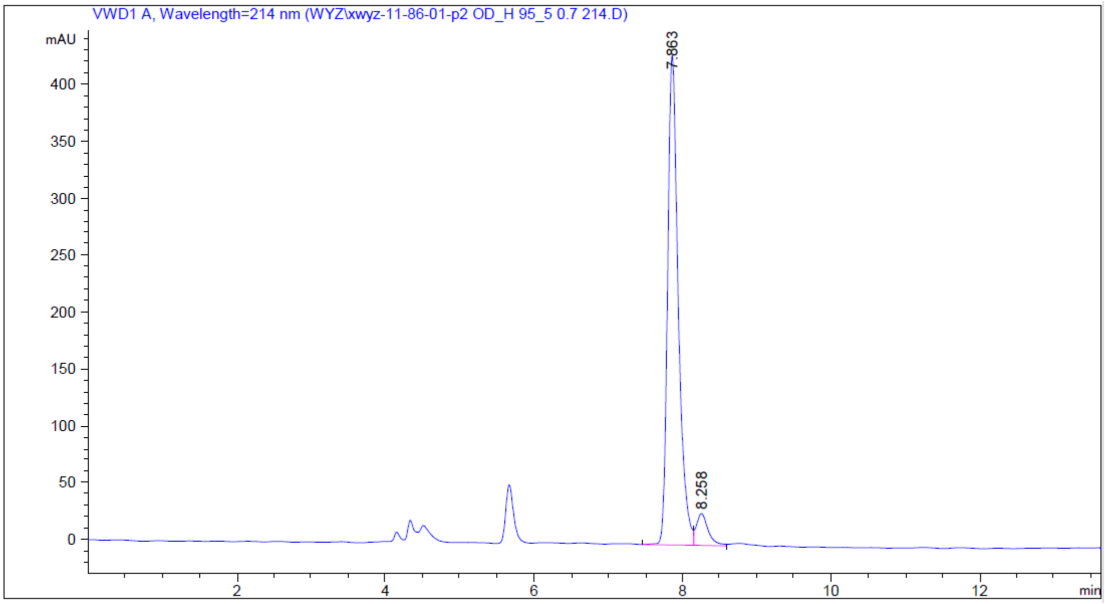

Peak NO	Ret. Time(min)	Area/%
1	7.863	93.3099
2	8.258	6.6901

methyl (*R*)-4-(1-phenylpropyl)benzoate (3a)[1]

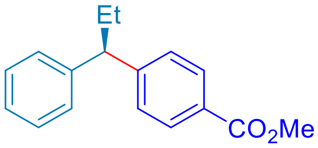

Prepared according to the general procedure **1** from (1-chloropropyl)benzene (0.4 mmol, 2 equiv.) and **2** from methyl 4-(((trifluoromethyl)sulfonyl)oxy)benzoate (0.2 mmol, 1 equiv.). The title compound was isolated (gradient 0–5% EtOAc/hexanes) as

a colorless oil (32.0 mg, 63% yield, 82% ee).

^{1}H NMR (400 MHz, CDCl$_3$) δ 7.98 (d, *J* = 8.4 Hz, 2H), 7.37 – 7.29 (m, 4H), 7.28 – 7.18 (m, 3H), 3.91 (s, 3H), 3.88 (t, *J* = 7.6 Hz, 1H), 2.19 – 2.05 (m, 2H), 0.93 (t, *J* = 7.2 Hz, 3H).

^{13}C NMR (101 MHz, CDCl$_3$) δ 167.1, 150.6, 144.2, 129.8, 128.5, 128.0, 128.0, 127.9, 126.4, 53.3, 52.0, 28.4, 12.7.

Enantiomeric excess = 82%, determined by HPLC (Daicel Chiralpak OD-H Column, *n*-Hexane:*i*-PrOH = 95:5, flow rate 0.7 mL/min, T = 25 °C, λ = 214 nm): t_R = 7.898 min (minor), t_R = 8.296 min (major).

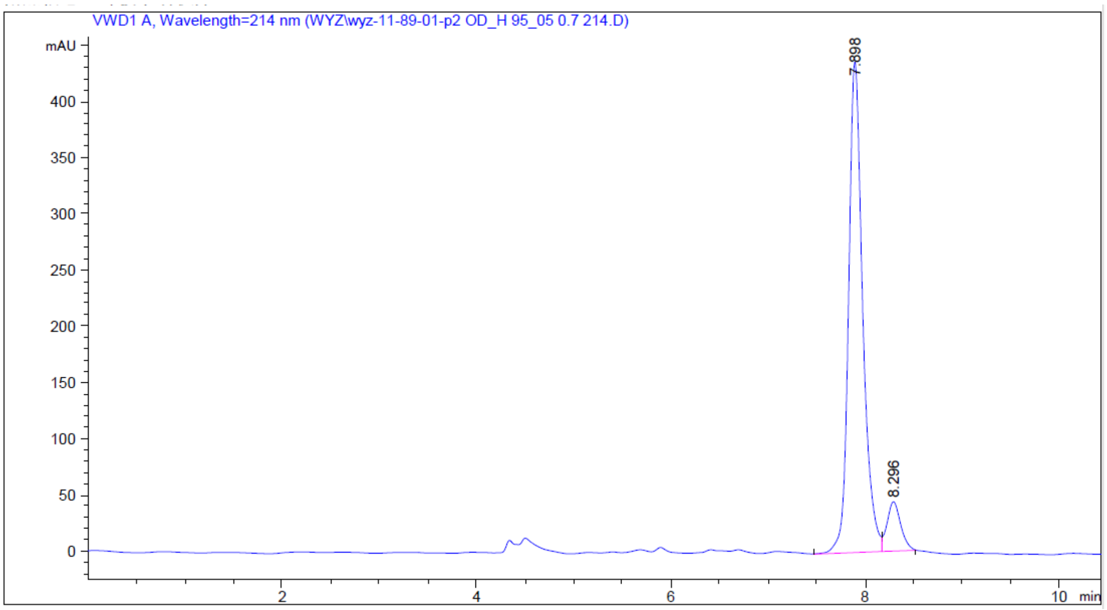

Peak NO	Ret. Time(min)	Area/%
1	7.898	91.1115
2	8.296	8.8885

ethyl (*R*)-4-(1-phenylpropyl)benzoate (3b) [1]

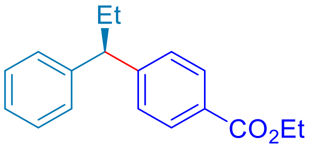

Prepared according to the general procedure **1** from (1-chloropropyl)benzene (0.4 mmol, 2 equiv.) and **2** from ethyl 4-bromobenzoate (0.2 mmol, 1 equiv.). The title compound was isolated (gradient 0–5% EtOAc/hexanes) as a yellow oil (45.5 mg, 85% yield, 89% ee).

^{1}H NMR (400 MHz, CDCl$_3$) δ 7.99 (d, *J* = 8.4 Hz, 2H), 7.44 – 7.17 (m, 7H), 4.38 (q, *J* = 7.2 Hz, 2H), 3.88 (t, *J* = 7.6 Hz, 1H), 2.12 (p, *J* = 7.2 Hz, 2H), 1.40 (t, *J* = 7.2 Hz,

3H), 0.93 (t, J = 7.2 Hz, 3H).

^{13}C NMR (101 MHz, $CDCl_3$) δ 166.6, 150.4, 144.3, 129.7, 128.5,128.4, 127.9, 127.9, 126.3, 60.8, 53.2, 28.4, 14.4, 12.7.

$[\alpha]_D$ ^{23} = -4.25 (c = 0.2, $CHCl_3$).

Enantiomeric excess = 89%, determined by HPLC (Daicel Chiralpak OJ-H Column, n-Hexane:i-PrOH = 95:5, flow rate 1.0 mL/min, T = 25 °C, λ = 214 nm): t_R = 6.520 min (minor), t_R = 7.238 min (major).

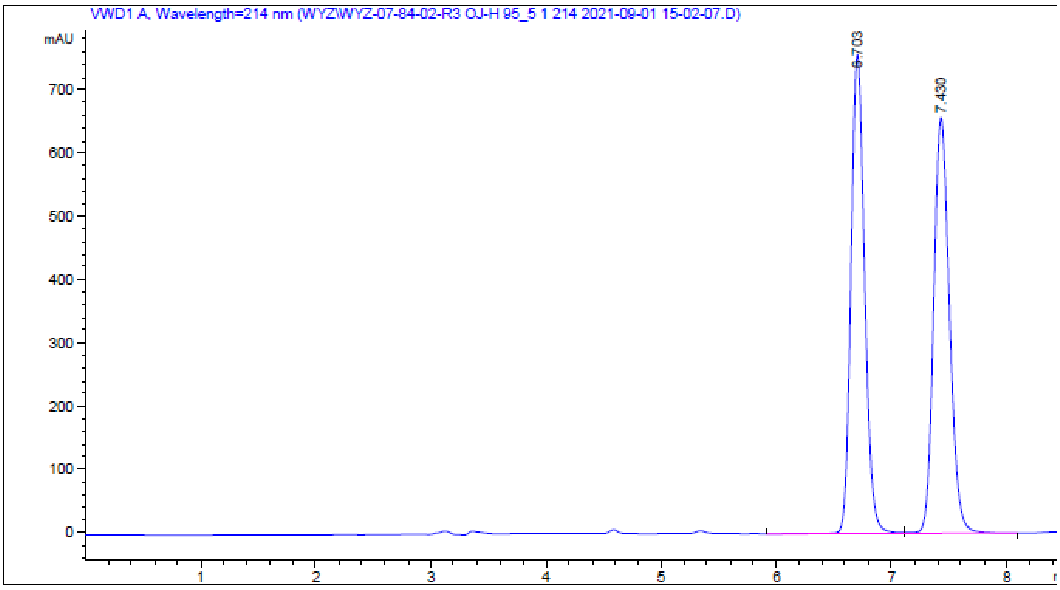

Peak NO	Ret. Time(min)	Area/%
1	6.703	49.8960
2	7.430	50.1040

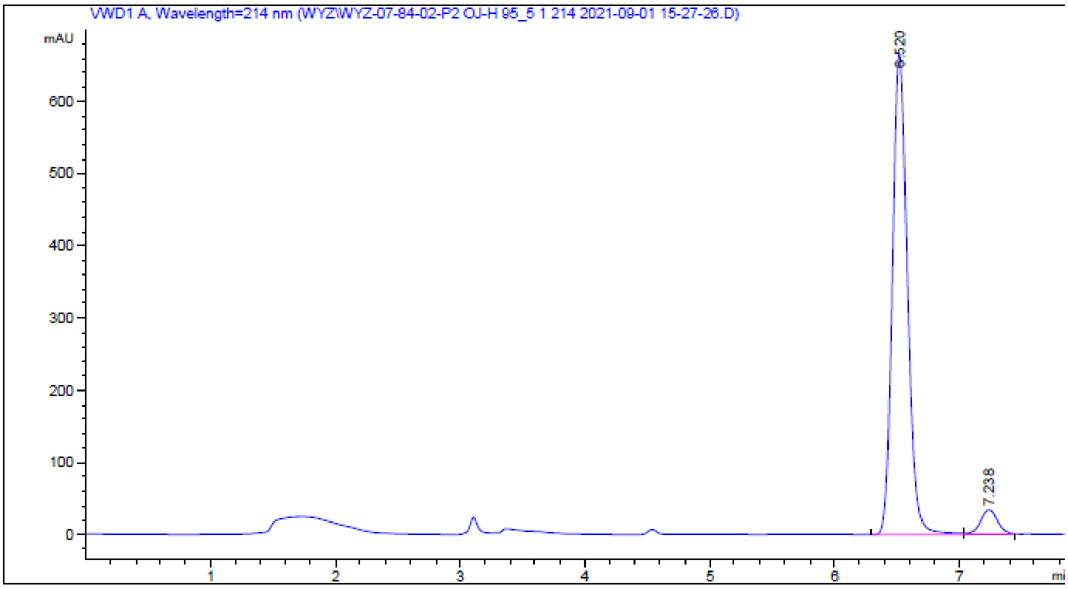

Peak NO	Ret. Time(min)	Area/%

1	6.520	94.3116
2	7.238	5.6884

ethyl (*R*)-4-(1-phenylpropyl)benzoate (3b)[1]

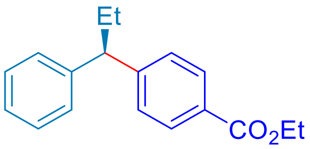

Prepared according to the general procedure **1** from (1-chloropropyl)benzene (0.4 mmol, 2 equiv.) and **2** from ethyl 4-chlorobenzoate (0.2 mmol, 1 equiv.). The title compound was isolated (gradient 0–5% EtOAc/hexanes) as a yellow oil (30.6 mg, 57% yield, 88% ee).

^{1}H NMR (400 MHz, $CDCl_3$) δ 7.95 (d, *J* = 8.4 Hz, 2H), 7.33 – 7.11 (m, 7H), 4.34 (q, *J* = 7.2 Hz, 2H), 3.84 (t, *J* = 7.6 Hz, 1H), 2.14 – 2.03 (m, 2H), 1.36 (t, *J* = 7.2 Hz, 3H), 0.90 (t, *J* = 7.2 Hz, 3H).

^{13}C NMR (101 MHz, $CDCl_3$) δ 166.6, 150.4, 144.2, 129.7, 128.5, 128.3, 127.9, 127.9, 126.3, 60.8, 53.2, 28.3, 14.3, 12.7.

Enantiomeric excess = 88%, determined by HPLC (Daicel Chiralpak OJ-H Column, *n*-Hexane:*i*-PrOH = 95:5, flow rate 1.0 mL/min, T = 25 °C, λ = 214 nm): t_R = 6.788 min (minor), t_R = 7.559 min (major).

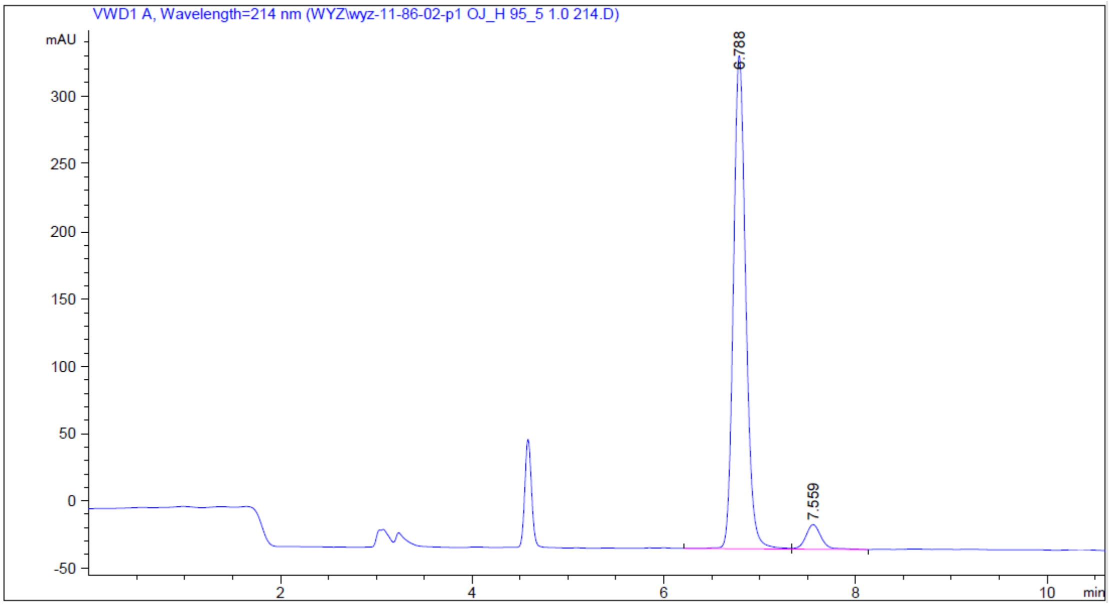

Peak NO	Ret. Time(min)	Area/%
1	6.788	94.1948
2	7.559	5.8052

(*R*)-1-(methylsulfonyl)-4-(1-phenylpropyl)benzene (3c)

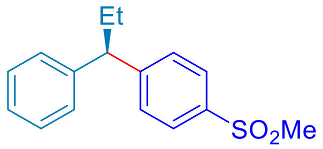

Prepared according to the general procedure 1 from (1-chloropropyl)benzene (0.4 mmol, 2 equiv.) and 2 from 1-bromo-4-(methylsulfonyl)benzene (0.2 mmol, 1 equiv.). The title compound was isolated (gradient 0–5% EtOAc/hexanes) as a yellow oil (41.0 mg, 75% yield, 89% ee).

^{1}H NMR (400 MHz, $CDCl_3$) δ 7.76 (d, *J* = 8.4 Hz, 2H), 7.35 (d, *J* = 8.0 Hz, 2H), 7.26 – 7.19 (m, 2H), 7.19 – 7.08 (m, 3H), 3.81 (t, *J* = 7.6 Hz, 1H), 2.93 (s, 3H), 2.08 – 1.95 (m, 2H), 0.83 (t, *J* = 7.2 Hz, 3H).

^{13}C NMR (101 MHz, $CDCl_3$) δ 151.8, 143.5, 138.2, 128.9, 128.7, 127.9, 127.6, 126.6, 53.2, 44.6, 28.3, 12.6.

IR (neat): 3727, 2925, 1457, 1306, 1149, 1091, 957, 798, 683, 613 cm^{-1}

HRMS (EI) calcd for $C_{16}H_{18}O_2S$ $[M]^+$:274.1024; found: 274.1022.

$[\alpha]_D$ ^{24} = -26.59 (*c* = 0.2, $CHCl_3$).

Enantiomeric excess = 89%, determined by HPLC (Daicel Chiralpak OD-H Column, *n*-Hexane:*i*-PrOH = 95:5, flow rate 1.0 mL/min, T = 25 °C, λ = 214 nm): t_R = 39.325 min (minor), t_R = 42.569 min (major).

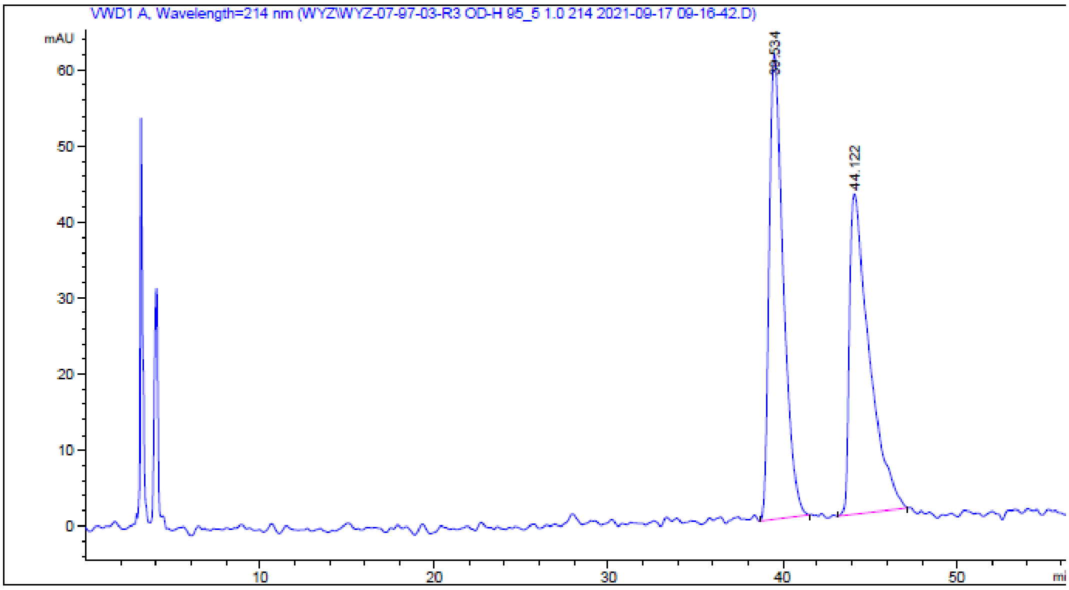

Peak NO	Ret. Time(min)	Area/%
1	39.534	50.7762
2	44.122	49.2238

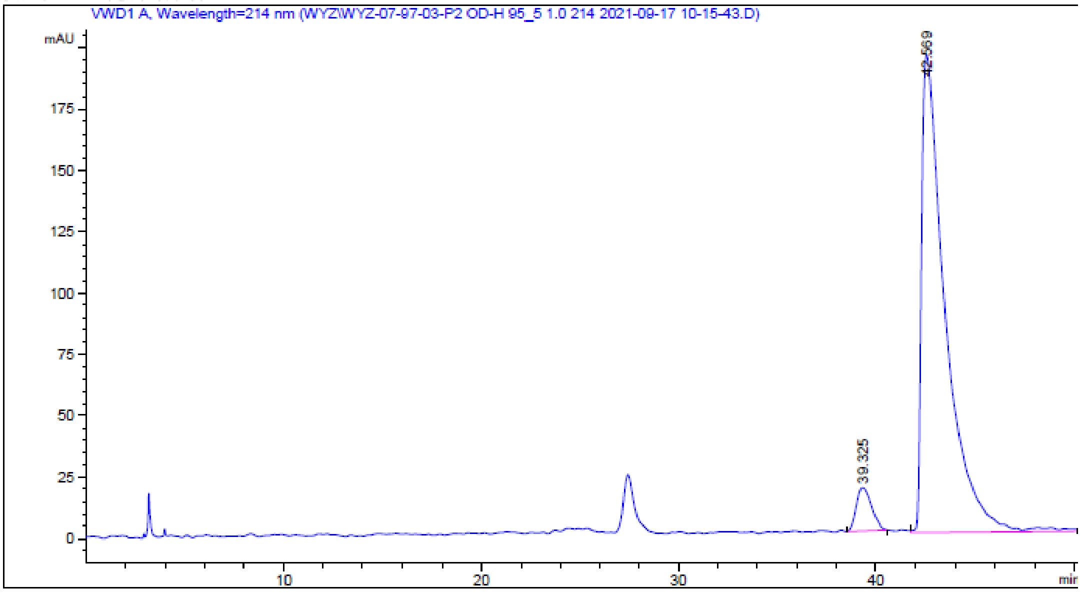

Peak NO	Ret. Time(min)	Area/%
1	39.325	5.4806
2	42.569	94.5194

(*R*)-1-(methylsulfonyl)-4-(1-phenylpropyl)benzene (3c)

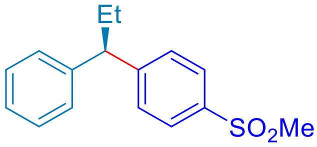

Prepared according to the general procedure 1 from (1-chloropropyl)benzene (0.4 mmol, 2 equiv.) and 2 from 1-chloro-4-(methylsulfonyl)benzene (0.2 mmol, 1 equiv.). The title compound was isolated (gradient 0–5% EtOAc/hexanes) as a yellow oil (27.9 mg, 51% yield, 90% ee).

^{1}H NMR (400 MHz, CDCl$_3$) δ 7.83 (d, *J* = 8.0 Hz, 2H), 7.42 (d, *J* = 8.0 Hz, 2H), 7.32 – 7.25 (m, 2H), 7.25 – 7.13 (m, 3H), 3.88 (t, *J* = 7.6 Hz, 1H), 3.01 (s, 3H), 2.21 – 2.00 (m, 2H), 0.90 (t, *J* = 7.2 Hz, 3H).

^{13}C NMR (101 MHz, CDCl$_3$) δ 151.7, 143.5, 138.2, 128.8, 128.6, 127.8, 127.5, 126.6, 53.2, 44.5, 28.3, 12.6.

Enantiomeric excess = 90%, determined by HPLC (Daicel Chiralpak OD-H Column, *n*-Hexane:*i*-PrOH = 95:5, flow rate 1.0 mL/min, T = 25 °C, λ = 214 nm): t_R = 40.774 min (minor), t_R = 44.806 min (major).

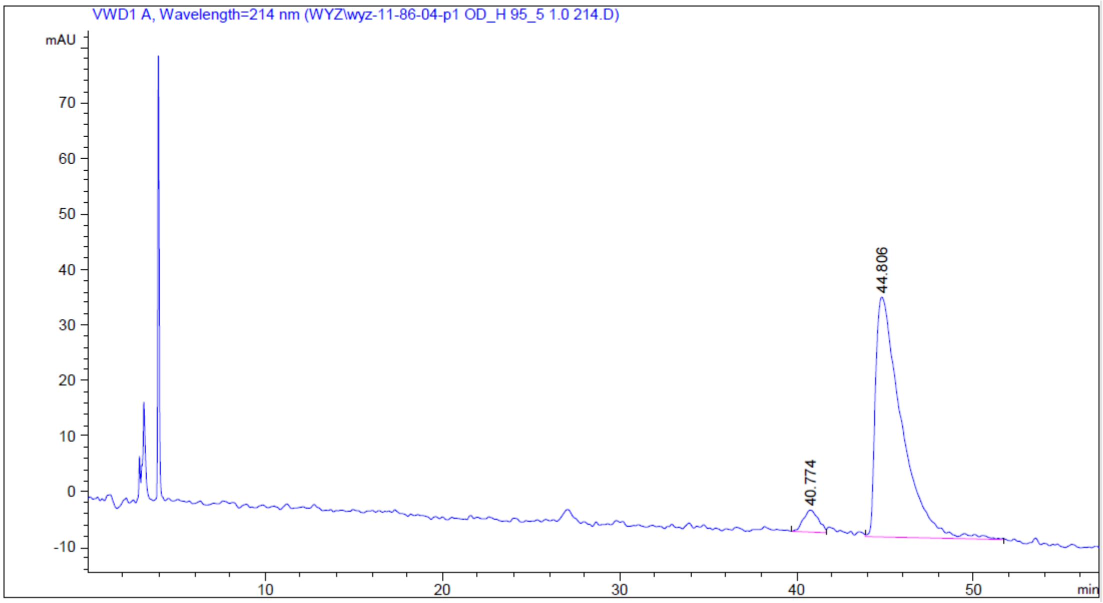

Peak NO	Ret. Time(min)	Area/%
1	40.774	5.0537
2	44.806	94.9463

(*R*)-4-(1-phenylpropyl)benzonitrile(3d)[2]

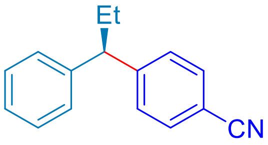

Prepared according to the general procedure 1 from (1-chloropropyl)benzene (0.4 mmol, 2 equiv.) and 2 from 4-bromobenzonitrile (0.2 mmol, 1 equiv.). The title compound was isolated (gradient 0–5% EtOAc/hexanes) as a colorless oil (33.2 mg, 75% yield, 82% ee).

^{1}H NMR (400 MHz, $CDCl_3$) δ 7.56 (d, *J* = 8.4 Hz, 2H), 7.33 (d, *J* = 8.4 Hz, 2H), 7.29 (d, *J* = 7.2 Hz, 2H), 7.24 – 7.15 (m, 3H), 3.84 (t, *J* = 7.6 Hz, 1H), 2.15 – 1.99 (m, 2H), 0.90 (t, *J* = 7.2 Hz, 3H).

^{13}C NMR (101 MHz, $CDCl_3$) δ 150.8, 143.5, 132.3, 128.7, 128.7, 127.9, 126.6, 119.0, 109.9, 53.3, 28.2, 12.6.

$[\alpha]_D$ ^{22} = -0.42 (*c* = 1.0, $CHCl_3$).

Enantiomeric excess = 82%, determined by HPLC (Daicel Chiralpak OJ-H Column, *n*-Hexane:*i*-PrOH = 90:10, flow rate 1.0 mL/min, T = 25 °C, λ = 214 nm): t_R = 9.555 min (minor), t_R = 10.579 min (major).

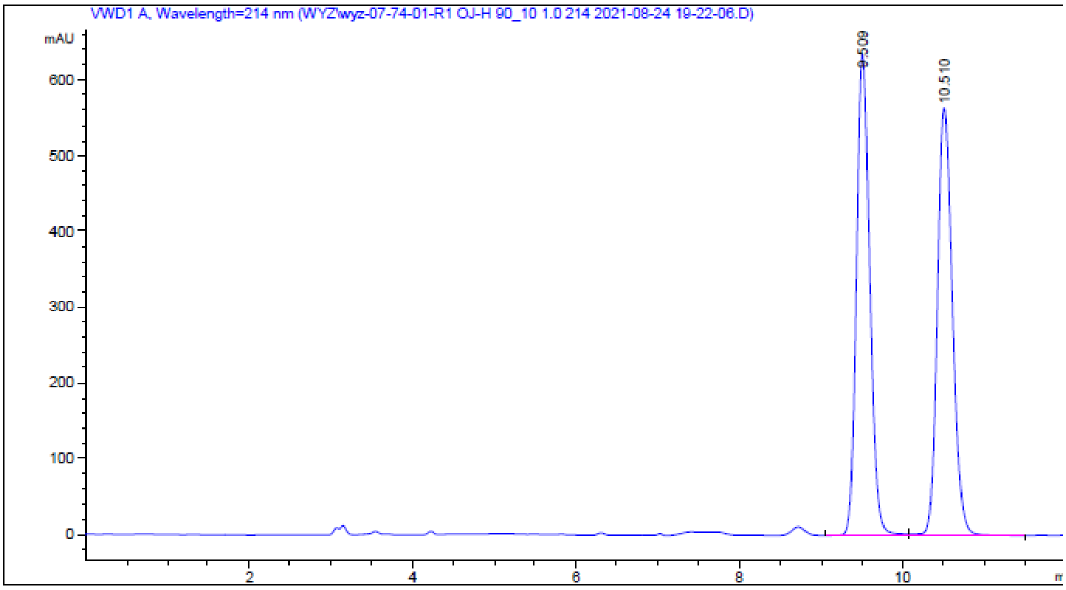

Peak NO	Ret. Time (min)	Area/%
1	9.509	49.9928
2	10.510	50.0072

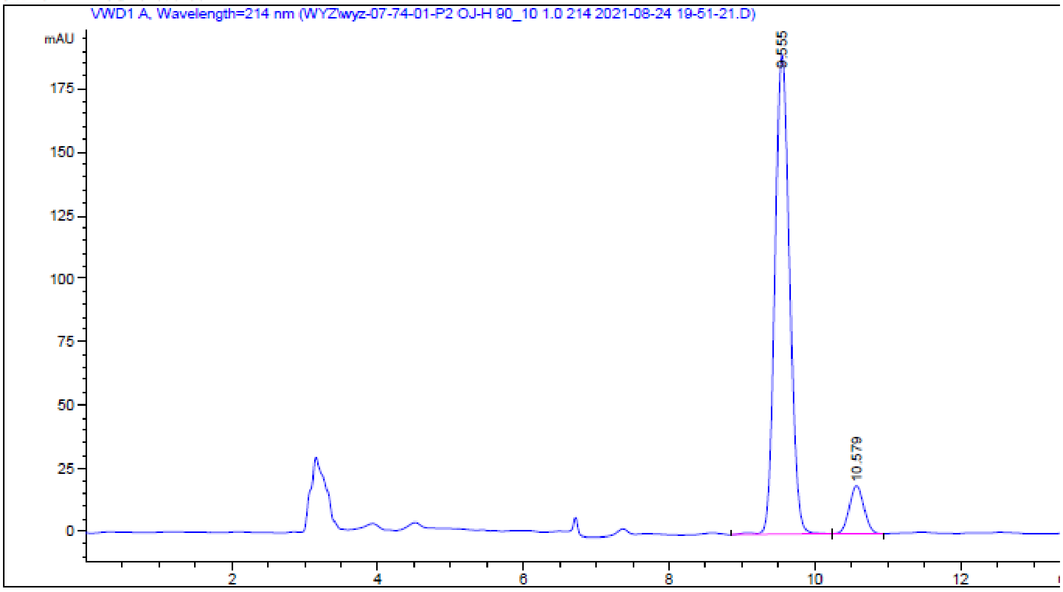

Peak NO	Ret. Time (min)	Area/%
1	9.555	90.9026
2	10.579	9.0974

(*R*)-4-(1-phenylpropyl)benzonitrile(3d)[2]

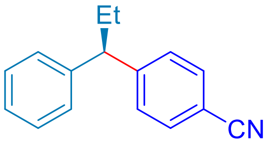

Prepared according to the general procedure 1 from (1-chloropropyl)benzene (0.4 mmol, 2 equiv.) and 2 from 4-chlorobenzonitrile (0.2 mmol, 1 equiv.). The title compound was isolated (gradient 0–5% EtOAc/hexanes) as a colorless oil (38.5 mg, 87% yield, 82% ee).

^{1}H NMR (400 MHz, CDCl$_3$) δ 7.62 – 7.50 (m, 2H), 7.33 (d, *J* = 8.4 Hz, 2H), 7.31 – 7.26 (m, 2H), 7.24 – 7.15 (m, 3H), 3.84 (t, *J* = 7.6 Hz, 1H), 2.16 – 1.98 (m, 2H), 0.90 (t, *J* = 7.2 Hz, 3H).

^{13}C NMR (101 MHz, CDCl$_3$) δ 150.8, 143.5, 132.3, 128.7, 128.7, 127.9, 126.7, 119.1, 109.9, 53.3, 28.2, 12.6.

Enantiomeric excess = 82%, determined by HPLC (Daicel Chiralpak OJ-H Column, *n*-Hexane:*i*-PrOH = 90:10, flow rate 1.0 mL/min, T = 25 °C, λ = 214 nm): t_R = 10.181 min (minor), t_R = 11.295 min (major).

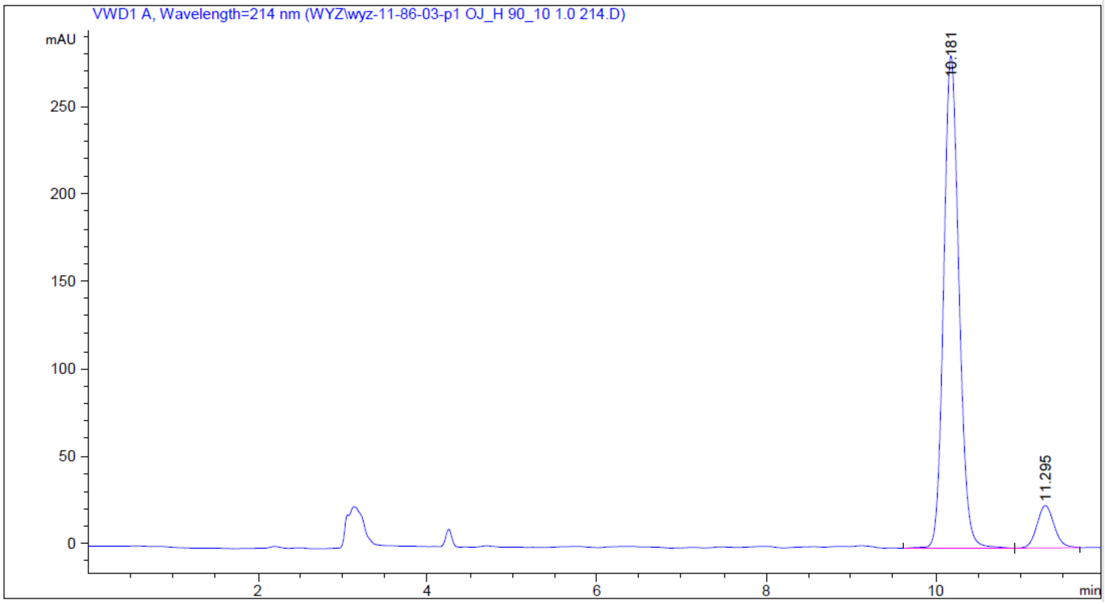

Peak NO	Ret. Time (min)	Area/%
1	10.181	91.1509
2	11.295	8.8491

(*R*)-4-(1-phenylpropyl)-1,1'-biphenyl (3e)[3]

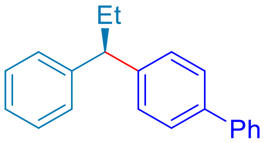

Prepared according to the general procedure **1** from (1-chloropropyl)benzene (0.4 mmol, 2 equiv.) and **2** from ethyl 4-bromo-1,1'-biphenyl (0.2 mmol, 1 equiv.). The title compound was isolated (gradient 0–5% EtOAc/hexanes) as a white soild (38.6 mg, 71% yield, 91% ee).

^{1}H NMR (400 MHz, CDCl$_3$) δ 7.61 (d, *J* = 7.6 Hz, 2H), 7.56 (d, *J* = 8.0 Hz, 2H), 7.46 (t, *J* = 7.6 Hz, 2H), 7.41 – 7.29 (m, 7H), 7.27 – 7.20 (m, 1H), 3.89 (t, *J* = 7.6 Hz, 1H), 2.17 (p, *J* = 7.2 Hz, 2H), 0.99 (t, *J* = 7.2 Hz, 3H).

^{13}C NMR (101 MHz, CDCl$_3$) δ 145.1, 144.4, 141.0, 138.9, 128.7, 128.5, 128.3, 128.0, 127.1, 127.1, 127.0, 126.1, 53.0, 28.7, 12.9.

$[\alpha]_D^{24}$ = +5.02 (*c* = 0.8, CHCl$_3$).

Enantiomeric excess = 91%, determined by HPLC (Daicel Chiralpak OJ-H Column, *n*-Hexane:*i*-PrOH = 95:5, flow rate 0.7 mL/min, T = 25 °C, λ = 214 nm): t_R = 12.866 min (minor), t_R = 14.553 min (major).

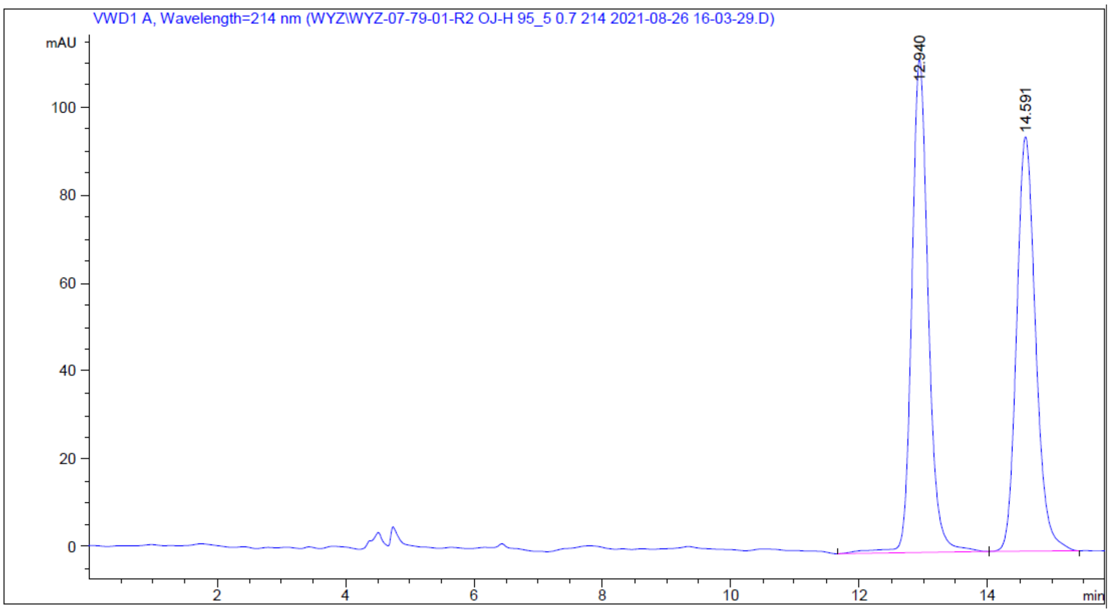

Peak NO	Ret. Time (min)	Area/%
1	12.940	50.8548
2	14.591	49.1452

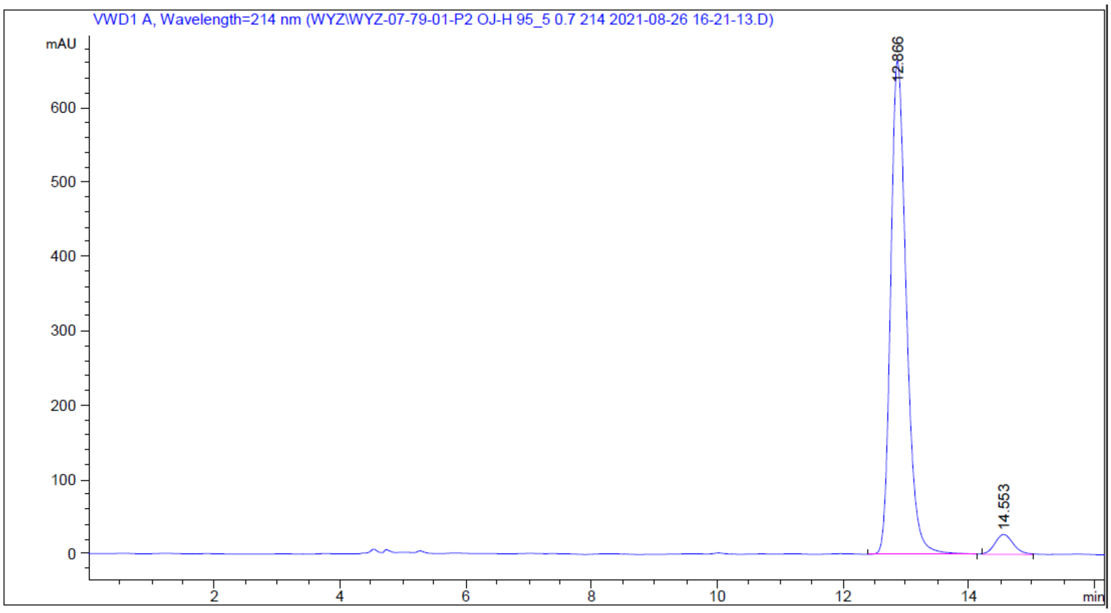

Peak NO	Ret. Time (min)	Area/%
1	12.866	95.4747
2	14.553	4.5253

(*R*)-4-(1-phenylpropyl)-1,1'-biphenyl (3e)[3]

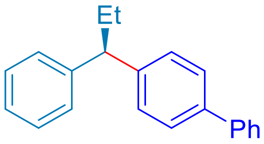

Prepared according to the general procedure **1** from (1-chloropropyl)benzene (0.4 mmol, 2 equiv.) and **2** from 4-chloro-1,1'-biphenyl (0.2 mmol, 1 equiv.). The title compound was isolated (gradient 0–5% EtOAc/hexanes) as a white soild (35.4 mg, 65% yield, 91% ee).

^{1}H NMR (400 MHz, CDCl$_3$) δ 7.57 (d, *J* = 7.2 Hz, 2H), 7.52 (d, *J* = 8.4 Hz, 2H), 7.46 – 7.39 (m, 2H), 7.37 – 7.27 (m, 7H), 7.23 – 7.16 (m, 1H), 3.85 (t, *J* = 7.6 Hz, 1H), 2.22 – 2.06 (m, 2H), 0.94 (t, *J* = 7.2 Hz, 3H).

^{13}C NMR (101 MHz, CDCl$_3$) δ 145.1, 144.3, 141.0, 138.9, 128.7, 128.4, 128.3, 128.0, 127.1, 127.0, 127.0, 126.1, 53.0, 28.6, 12.9.

Enantiomeric excess = 91%, determined by HPLC (Daicel Chiralpak OJ-H Column, *n*-Hexane:*i*-PrOH = 95:5, flow rate 0.7 mL/min, T = 25 °C, λ = 214 nm): t_R = 7.534 min (minor), t_R = 8.571 min (major).

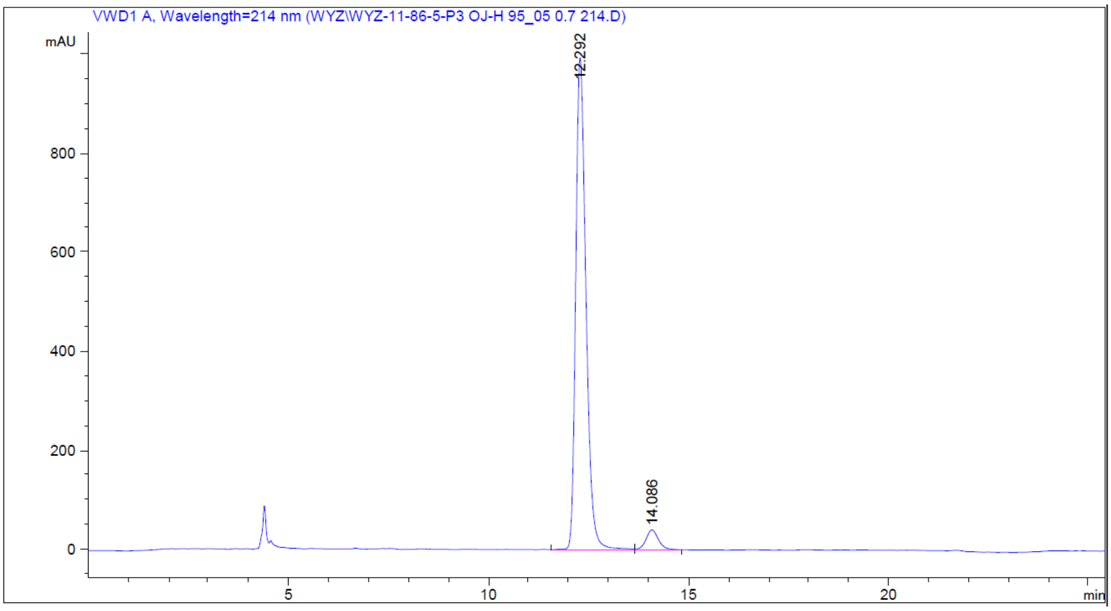

Peak NO	Ret. Time (min)	Area/%
1	12.292	95.3609
2	14.086	4.6391

(*R*)-4-(1-phenylpropyl)-1,1'-biphenyl (3e)[3]

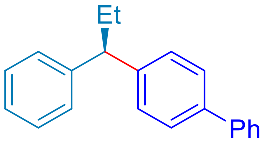

Prepared according to the general procedure **1** from (1-chloropropyl)benzene (0.4 mmol, 2 equiv.) and **2** from [1,1'-biphenyl]-4-yl trifluoromethanesulfonate (0.2 mmol, 1 equiv.). The title compound was isolated (gradient 0–5% EtOAc/hexanes) as a white soild (24.5 mg, 45% yield, 92% ee).

^{1}H NMR (400 MHz, $CDCl_3$) δ 7.55 (d, *J* = 7.2 Hz, 2H), 7.50 (d, *J* = 8.0 Hz, 2H), 7.44 – 7.37 (m, 2H), 7.33 – 7.25 (m, 7H), 7.23 – 7.13 (m, 1H), 3.93 – 3.75 (m, 1H), 2.15 – 2.05 (m, 2H), 0.93 (t, *J* = 7.2 Hz, 3H).

^{13}C NMR (101 MHz, $CDCl_3$) δ 145.0, 144.3, 141.0, 138.9, 128.6, 128.4, 128.2, 127.9, 127.0, 127.0, 126.9, 126.0, 52.9, 28.6, 12.8.

Enantiomeric excess = 92%, determined by HPLC (Daicel Chiralpak OJ-H Column, *n*-Hexane:*i*-PrOH = 95:5, flow rate 0.7 mL/min, T = 25 °C, λ = 214 nm): t_R = 7.534 min (minor), t_R = 8.571 min (major).

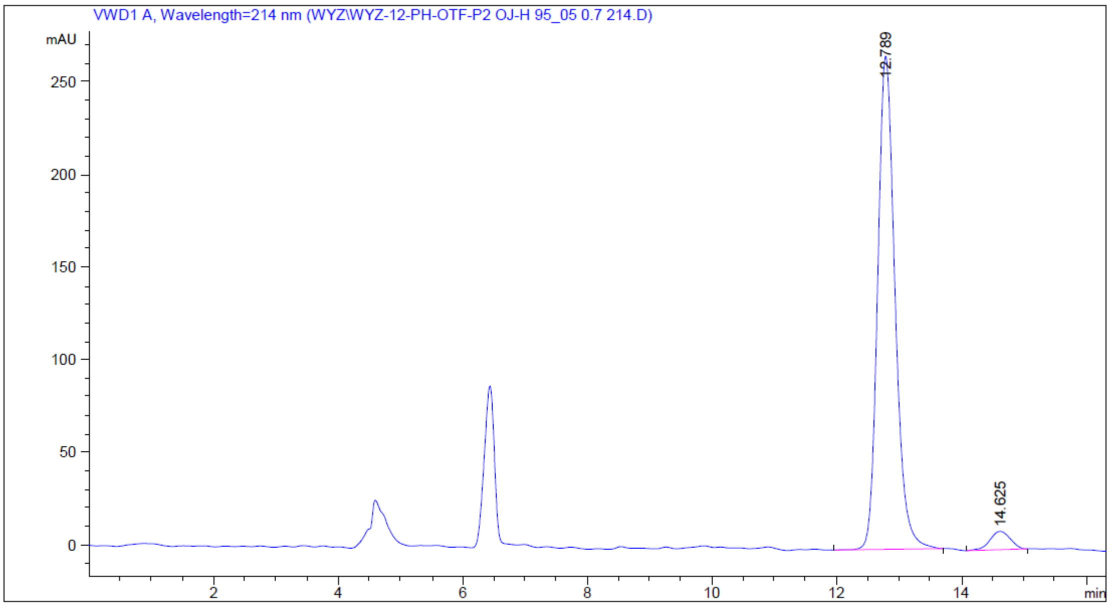

Peak NO	Ret. Time (min)	Area/%
1	12.789	95.8441
2	14.625	4.1559

methyl (*R*)-4-(1,2-diphenylethyl)benzoate (3f)[4]

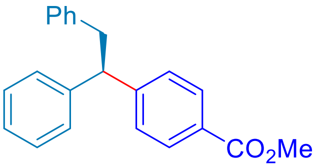

Prepared according to the general procedure **1** from (1-chloroethane-1,2-diyl)dibenzene (0.4 mmol, 2 equiv.) and **2** from methyl 4-bromobenzoate (0.2 mmol, 1 equiv.). The title compound was isolated (gradient 0–5% EtOAc/hexanes) as a white solid (51.0 mg, 81% yield, 94% ee).

M. P.: 154.1 – 155.9 °C

^{1}H NMR (400 MHz, $CDCl_3$) δ 7.97 (d, *J* = 8.4 Hz, 2H), 7.35 – 7.16 (m, 10H), 7.05 (d, *J* = 6.8 Hz, 2H), 4.35 (t, *J* = 7.6 Hz, 1H), 3.92 (s, 3H), 3.51 – 3.36 (m, 2H).

^{13}C NMR (101 MHz, $CDCl_3$) δ 167.1, 149.8, 143.7, 139.7, 129.8, 129.1, 128.6, 128.2, 128.2, 128.0, 126.6, 126.1, 53.2, 52.1, 41.9.

$[\alpha]_D^{25}$ = -52.46 (*c* = 0.8, $CHCl_3$).

Enantiomeric excess = 94%, determined by HPLC (Daicel Chiralpak OJ-H Column, *n*-Hexane:*i*-PrOH = 90:10, flow rate 1.0 mL/min, T = 25 °C, λ = 214 nm): t_R = 13.850 min (minor), t_R = 15.990 min (major).

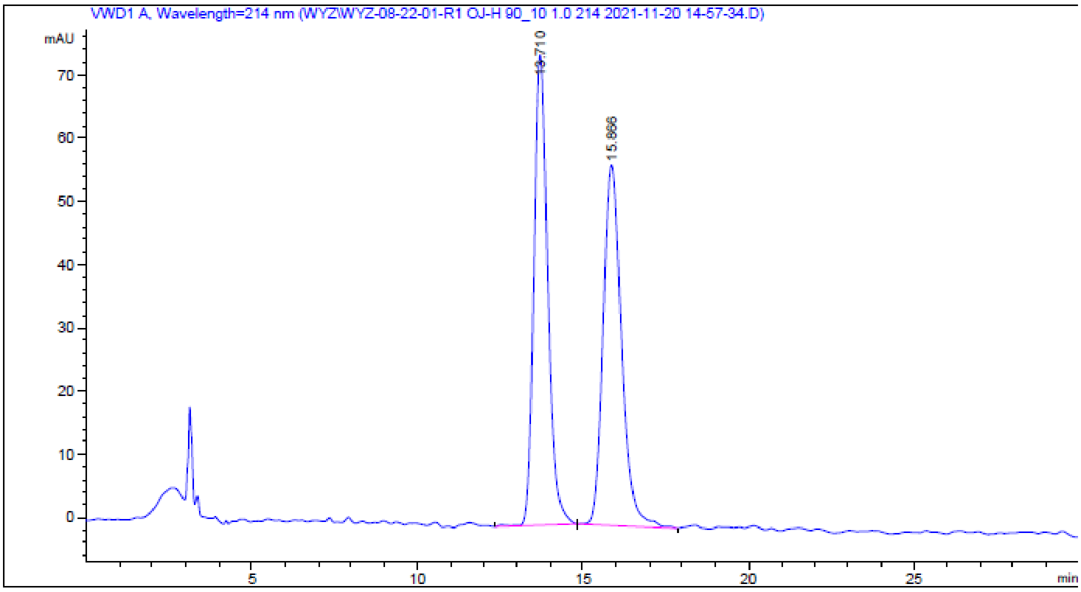

Peak NO	Ret. Time (min)	Area/%
1	13.710	49.6519
2	15.866	50.3481

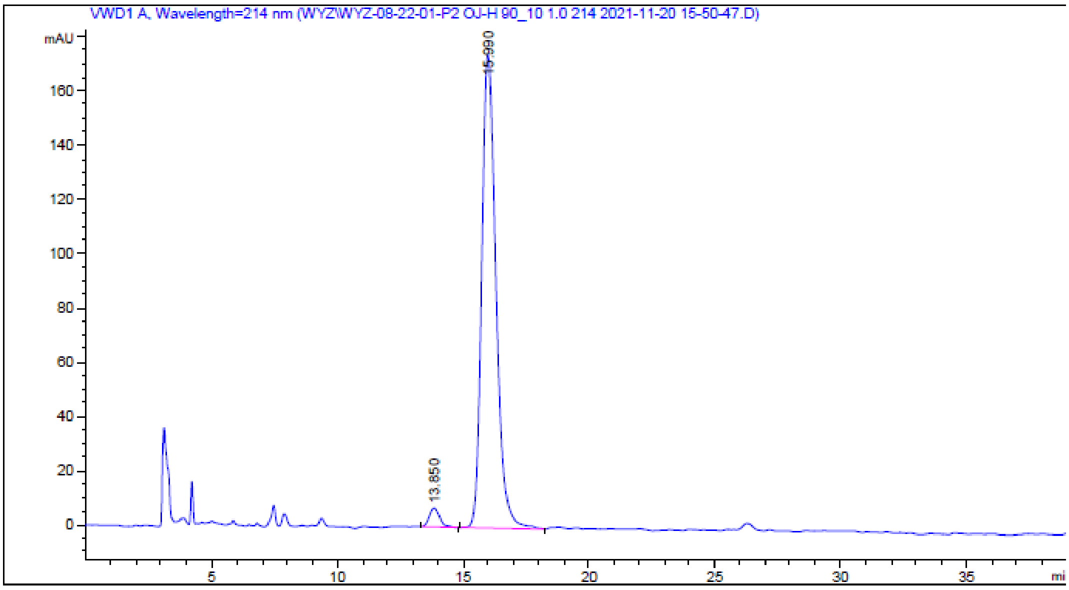

Peak NO	Ret. Time (min)	Area/%
1	13.850	2.8275
2	15.990	97.1725

methyl (*R*)-4-(1,2-diphenylethyl)benzoate (3f)[4]

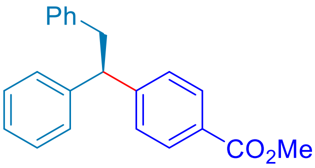

Prepared according to the general procedure **1** from (1-chloroethane-1,2-diyl)dibenzene (0.4 mmol, 2 equiv.) and **2** from methyl 4-chlorobenzoate (0.2 mmol, 1 equiv.). The title compound was isolated (gradient 0–5% EtOAc/hexanes) as a white solid (32.7 mg, 52% yield, 92% ee).

^{1}H NMR (400 MHz, CDCl$_3$) δ 7.91 (d, *J* = 8.4 Hz, 2H), 7.30 – 7.11 (m, 10H), 6.99 (d, *J* = 6.8 Hz, 2H), 4.29 (t, *J* = 7.6 Hz, 1H), 3.87 (s, 3H), 3.50 – 3.20 (m, 2H).

^{13}C NMR (101 MHz, CDCl$_3$) δ 167.0, 149.7, 143.6, 139.7, 129.7, 129.0, 128.5, 128.1, 128.0, 126.5, 126.1, 53.1, 52.0, 41.8.

Enantiomeric excess = 92%, determined by HPLC (Daicel Chiralpak OJ-H Column, *n*-Hexane:*i*-PrOH = 90:10, flow rate 1.0 mL/min, T = 25 °C, λ = 214 nm): t_R = 14.581 min (minor), t_R = 17.024 min (major).

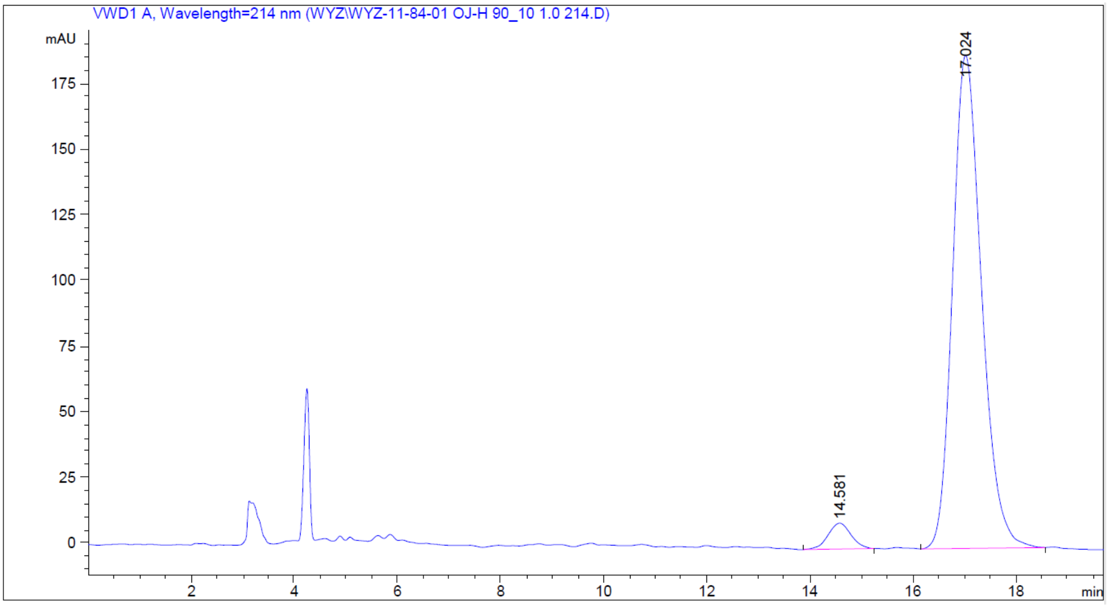

Peak NO	Ret. Time (min)	Area/%
1	14.581	3.9936
2	17.024	96.0064

***tert*-butyl (*R*)-4-(1-phenylpropyl)benzoate (3g)**

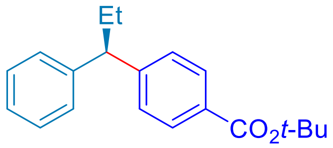

Prepared according to the general procedure **1** from (1-chloropropyl)benzene (0.4 mmol, 2 equiv.) and **2** from *tert*-butyl 4-bromobenzoate (0.2 mmol, 1 equiv.). The title compound was isolated (gradient 0–5% EtOAc/hexanes) as a white solid (44.4 mg, 75% yield, 88% ee).

M. P.: 143.2 – 146.3 °C

^{1}H NMR (400 MHz, CDCl$_3$) δ 7.93 (d, *J* = 7.6 Hz, 2H), 7.45 – 7.13 (m, 7H), 3.86 (t, *J* = 7.6 Hz, 1H), 2.18 – 2.03 (m, 2H), 1.59 (s, 9H), 0.92 (t, *J* = 7.2 Hz, 3H).
^{13}C NMR (101 MHz, CDCl$_3$) δ 165.7, 149.9, 144.3, 129.8, 129.6, 128.4, 127.8, 127.8, 126.2, 80.7, 53.1, 28.3, 28.2, 12.7. **IR** (neat): 2930, 1712, 1368, 1292, 1166, 1115, 1018, 844, 775, 735 cm^{-1}.
HRMS (EI) calcd for $C_{20}H_{24}O_2$ $[M]^+$:296.1769; found: 296.1771.
$[\alpha]_D$ ^{24} = -0.55 (*c* = 0.5, $CHCl_3$).
Enantiomeric excess = 88%, determined by HPLC (Daicel Chiralpak AD-H Column, *n*-Hexane:*i*-PrOH = 95:5, flow rate 0.7 mL/min, T = 25 °C, λ = 214 nm): t_R = 6.701 min (minor), t_R = 6.977 min (major).

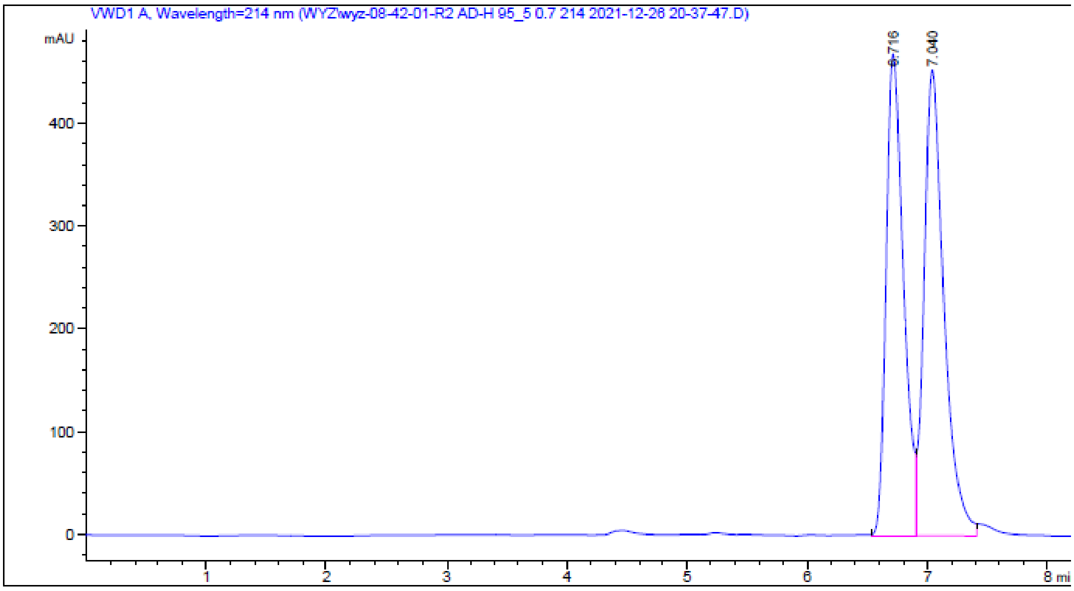

Peak NO	Ret. Time(min)	Area/%
1	6.716	47.3214
2	7.040	52.6786

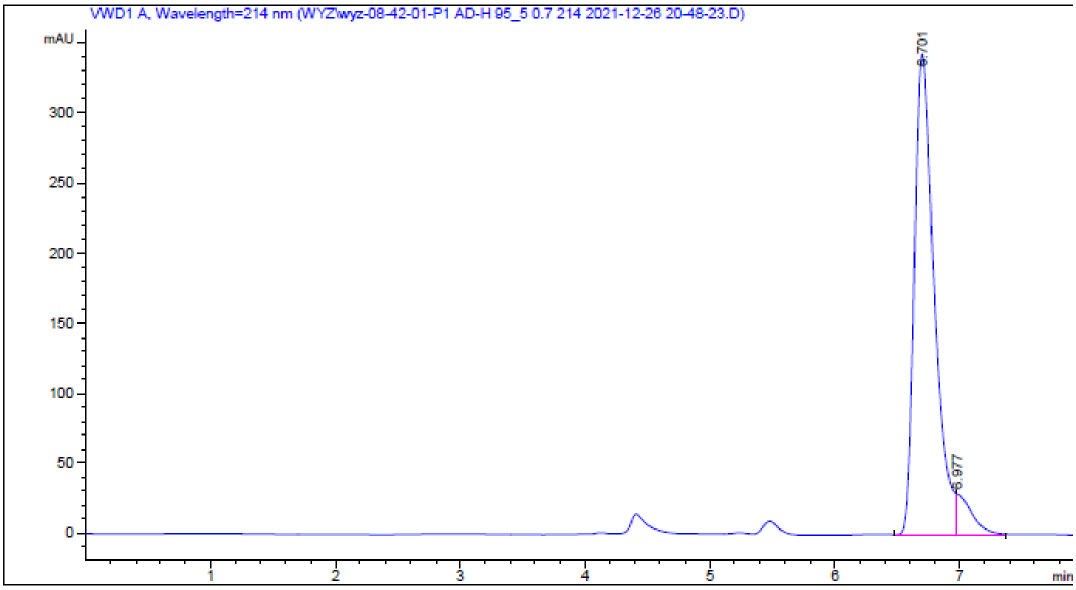

Peak NO	Ret. Time(min)	Area/%
1	6.701	93.9406
2	6.977	6.0594

(*R*)-1-methoxy-4-(1-phenylpropyl)benzene (3h)[2]

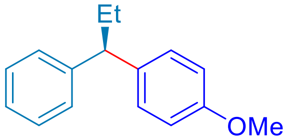

Prepared according to the general procedure **1** from (1-chloropropyl)benzene (0.4 mmol, 2 equiv.) and **2** from 1-bromo-4-methoxybenzene (0.2 mmol, 1 equiv.). The title compound was isolated (gradient 0–5% EtOAc/hexanes) as a colorless oil (31.8 mg, 70% yield, 87% ee).

^{1}H NMR (400 MHz, $CDCl_3$) δ 7.31 – 7.17 (m, 5H), 7.14 (d, *J* = 8.4 Hz, 2H), 6.87 – 6.76 (m, 2H), 3.84 – 3.65 (m, 4H), 2.04 (p, *J* = 7.6 Hz, 2H), 0.89 (t, *J* = 7.2 Hz, 3H).

^{13}C NMR (101 MHz, $CDCl_3$) δ 157.8, 145.6, 137.4, 128.8, 128.3, 127.8, 125.9, 113.7, 55.2, 52.4, 28.8, 12.8.

$[\alpha]_D$ ^{22} = -21.49 (*c* = 0.9, $CHCl_3$).

Enantiomeric excess = 87%, determined by HPLC (Daicel Chiralpak OJ-H Column, *n*-Hexane:*i*-PrOH = 90:10, flow rate 1.0 mL/min, T = 25 °C, λ = 214 nm): t_R = 11.153 min (minor), t_R = 12.145 min (major).

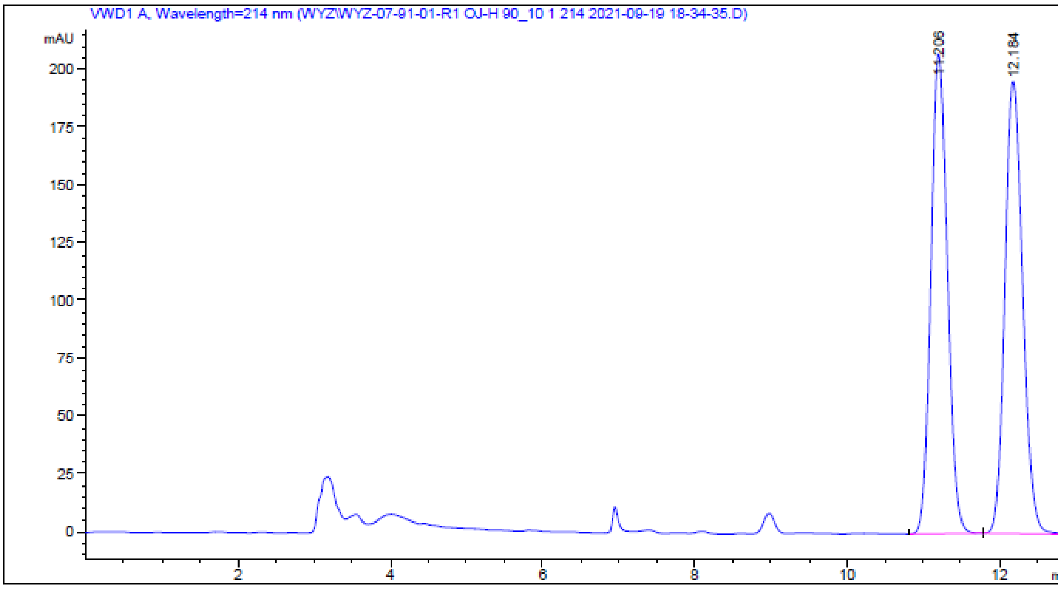

Peak NO	Ret. Time (min)	Area/%
1	11.206	49.6485
2	12.184	50.3515

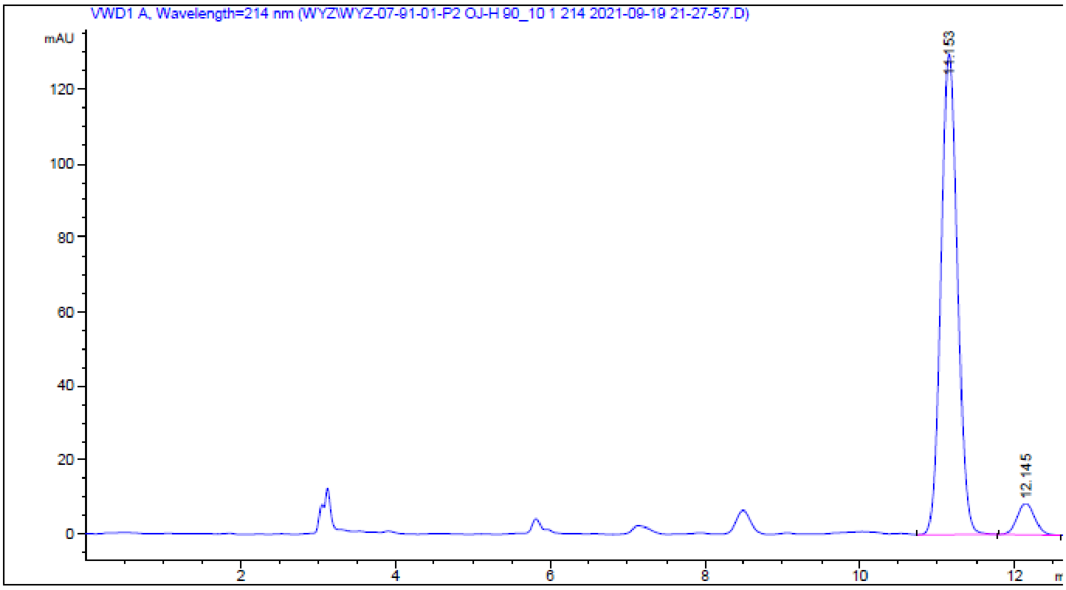

Peak NO	Ret. Time (min)	Area/%
1	11.153	93.4859
2	12.145	6.5141

(*R*)-4-(1-phenylpropyl)phenyl acetate (3i)

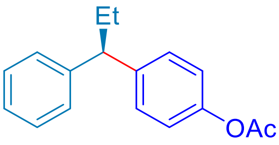

Prepared according to the general procedure **1** from (1-chloropropyl)benzene (0.4 mmol, 2 equiv.) and **2** from 4-bromophenyl acetate (0.2 mmol, 1 equiv.). The title compound was isolated (gradient 0–5% EtOAc/hexanes) as a yellow oil (36.9 mg, 73% yield, 85% ee).

^{1}H NMR (400 MHz, CDCl$_3$) δ 7.36 – 7.18 (m, 7H), 7.03 (d, J = 8.4 Hz, 2H), 3.83 (t, J = 7.6 Hz, 1H), 2.31 (s, 3H), 2.10 (p, J = 7.2 Hz, 2H), 0.94 (t, J = 7.2 Hz, 3H).

^{13}C NMR (101 MHz, CDCl$_3$) δ 169.6, 148.8, 144.8, 142.8, 128.8, 128.4, 128.0, 126.2, 121.3, 52.7, 28.7, 21.2, 12.8.

IR (neat): 2919, 1767, 1505, 1454, 1369, 1260, 1200, 1017, 910, 800 cm^{-1}.

HRMS (EI) calcd for $C_{17}H_{18}O_2$ $[M]^+$:254.1299; found: 254.1301.

$[\alpha]_D$ ^{23} = -5.47 (c = 0.1, CHCl$_3$).

Enantiomeric excess = 85%, determined by HPLC (Daicel Chiralpak OJ-H Column, n-Hexane:i-PrOH = 90:10, flow rate 1.0 mL/min, T = 25 °C, λ = 214 nm): t_R = 11.668 min (minor), t_R = 12.887 min (major).

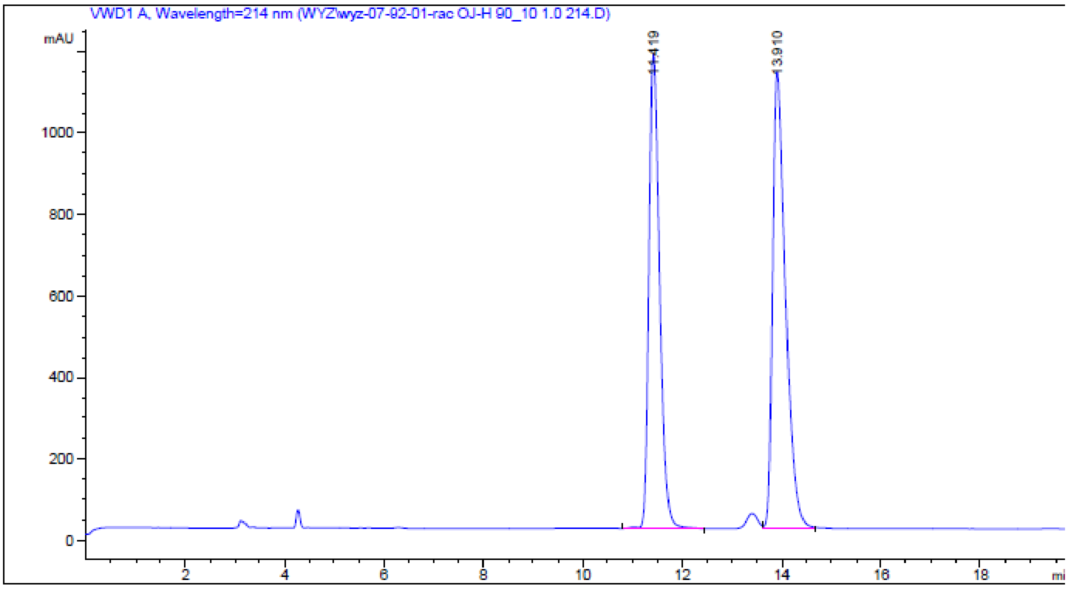

Peak NO	Ret. Time (min)	Area/%
1	11.419	45.8539
2	13.910	54.1461

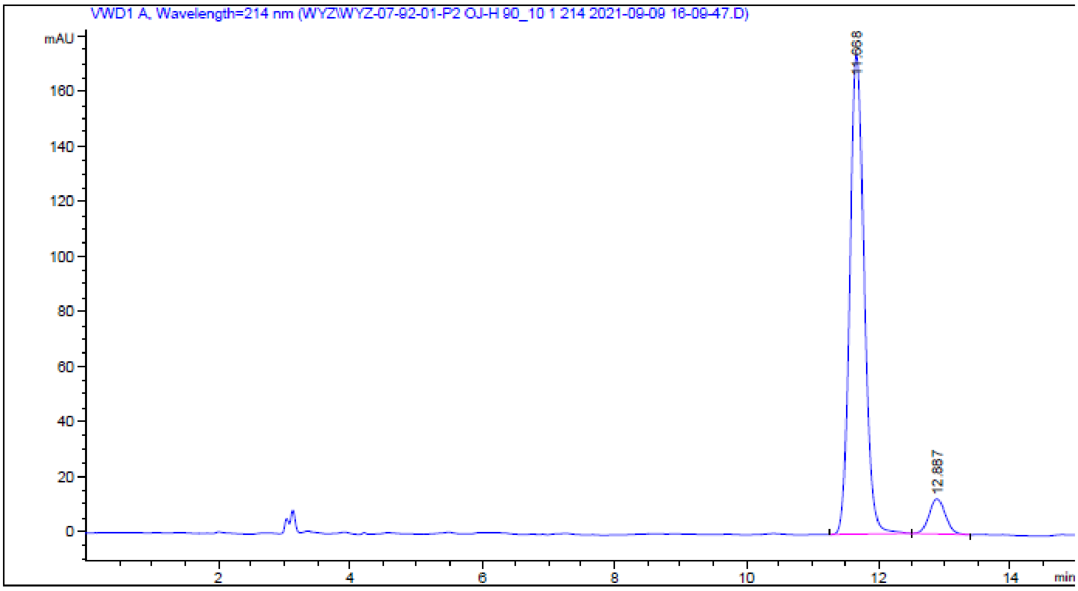

Peak NO	Ret. Time (min)	Area/%
1	11.668	92.3020
2	12.887	7.6980

(*R*)-3-(1-phenylpropyl)phenyl acetate (3j)

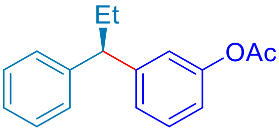

Prepared according to the general procedure **1** from (1-chloropropyl)benzene (0.4 mmol, 2 equiv.) and **2** from 3-bromophenyl acetate (0.2 mmol, 1 equiv.). The title compound was isolated (gradient 0–5% EtOAc/hexanes) as a colorless oil (45.7 mg, 90% yield, 82% ee).

^{1}H NMR (400 MHz, CDCl$_3$) δ 7.36 – 7.19 (m, 6H), 7.14 (d, J = 7.6 Hz, 1H), 7.00 (t, J = 1.6 Hz, 1H), 6.98 – 6.89 (m, 1H), 3.84 (t, J = 7.6 Hz, 1H), 2.31 (s, 3H), 2.10 (p, J = 7.2 Hz, 2H), 0.94 (t, J = 7.2 Hz, 3H).

^{13}C NMR (101 MHz, CDCl$_3$) δ 169.5, 150.7, 147.0, 144.5, 129.2, 128.5, 128.0, 126.2, 125.5, 120.9, 119.2, 52.9, 28.6, 21.2, 12.7.

IR (neat): 3501, 2953, 1767, 1587, 1261, 1142, 1014, 798, 722, 666 cm^{-1}.

HRMS (EI) calcd for $C_{17}H_{18}O_2$ $[M]^+$:254.1304; found: 254.1301.

$[\alpha]_D$ ^{26} = -3.22 (c = 0.2, $CHCl_3$).

Enantiomeric excess = 82%, determined by HPLC (Daicel Chiralpak OJ-H Column, *n*-Hexane:*i*-PrOH = 90:10, flow rate 1.0 mL/min, T = 25 °C, λ = 214 nm): t_R = 19.551 min (minor), t_R = 29.034 min (major).

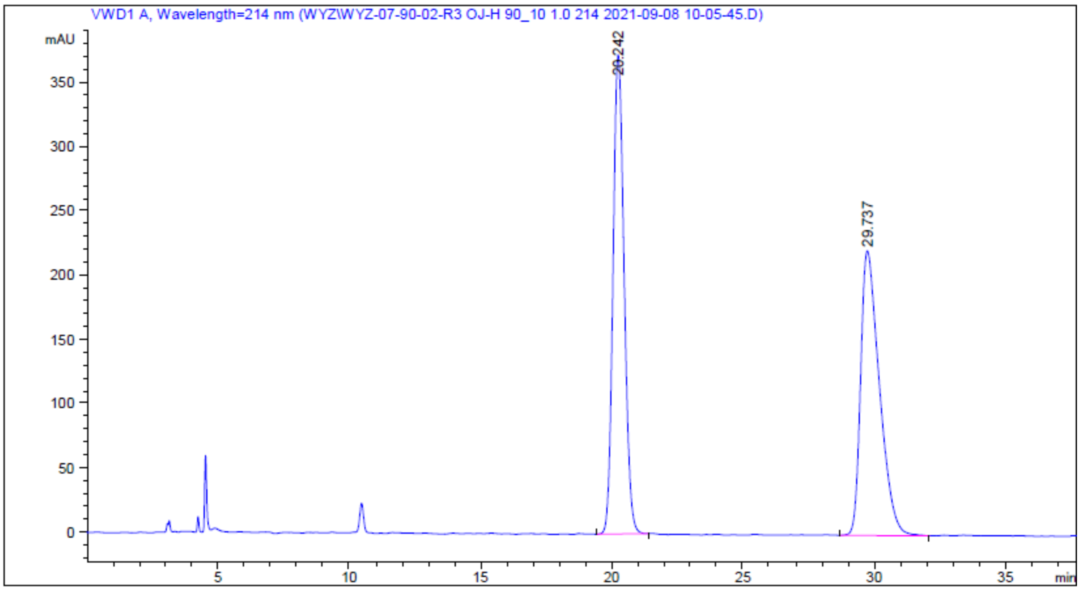

Peak NO	Ret. Time (min)	Area/%
1	20.242	50.1024
2	29.737	49.8976

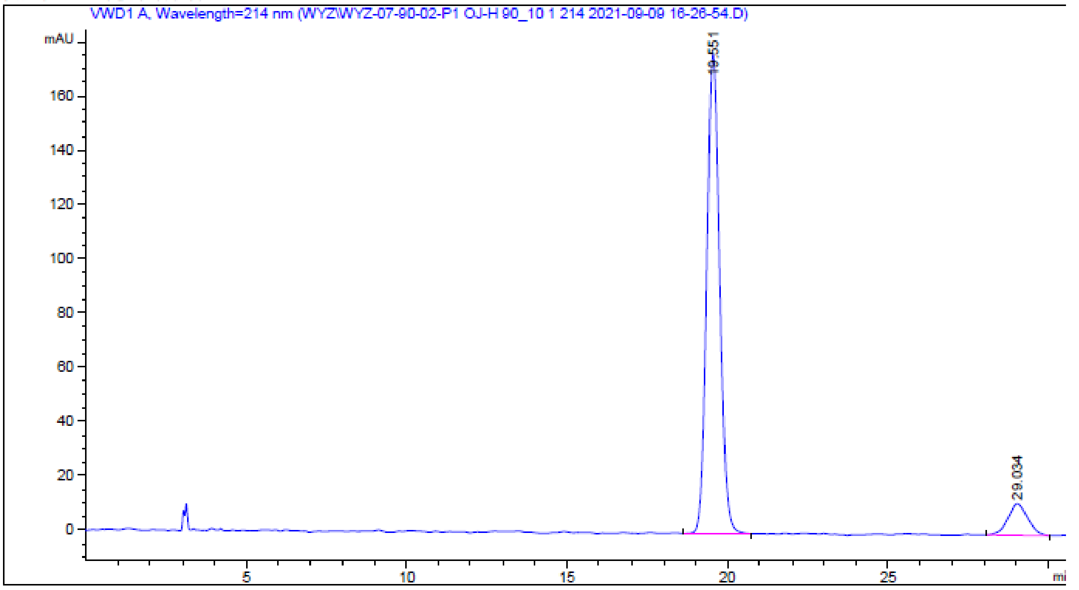

Peak NO	Ret. Time (min)	Area/%
1	19.551	90.7680
2	29.034	9.2320

ethyl (*R*)-2-(4-(1-phenylpropyl)phenyl)acetate (3k)[5]

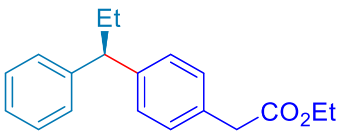

Prepared according to the general procedure **1** from (1-chloropropyl)benzene (0.4 mmol, 2 equiv.) and **2** from ethyl 2-(4-bromophenyl)acetate (0.2 mmol, 1 equiv.). The title compound was isolated (gradient 0–5% EtOAc/hexanes) as a yellow oil (45.7 mg, 81% yield, 84% ee).

^{1}H NMR (400 MHz, CDCl$_3$) δ 7.29 – 7.10 (m, 9H), 4.12 (dd, *J* = 14.0, 7.2 Hz, 2H), 3.76 (t, *J* = 7.6 Hz, 1H), 3.55 (s, 2H), 2.05 (p, *J* = 7.2 Hz, 2H), 1.23 (t, *J* = 7.2 Hz, 3H), 0.88 (t, *J* = 7.2 Hz, 3H).

^{13}C NMR (101 MHz, CDCl$_3$) δ 171.8, 145.1, 144.0, 131.8, 129.2, 128.4, 128.1, 128.0, 126.1, 60.8, 52.9, 41.0, 28.6, 14.2, 12.8.

$[\alpha]_D^{24}$ = -9.91 (*c* = 0.2, CHCl$_3$).

Enantiomeric excess = 84%, determined by HPLC (Daicel Chiralpak OJ-H Column, *n*-Hexane:*i*-PrOH = 90:10, flow rate 1.0 mL/min, T = 25 °C, λ = 214 nm): t_R = 10.462 min (minor), t_R = 11.863 min (major).

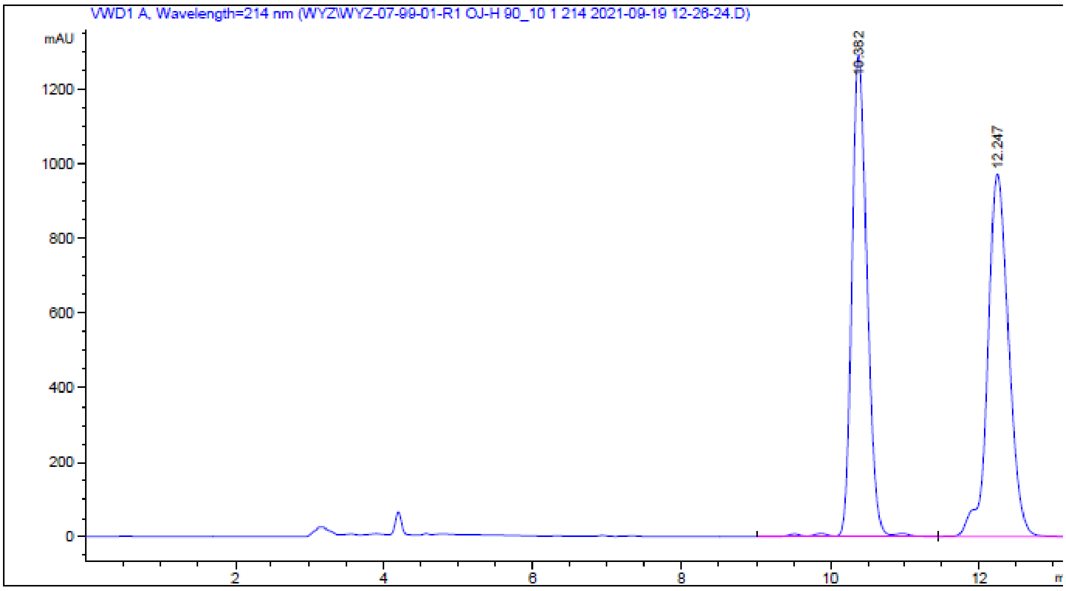

Peak NO	Ret. Time (min)	Area/%
1	10.382	49.2214
2	12.247	50.7786

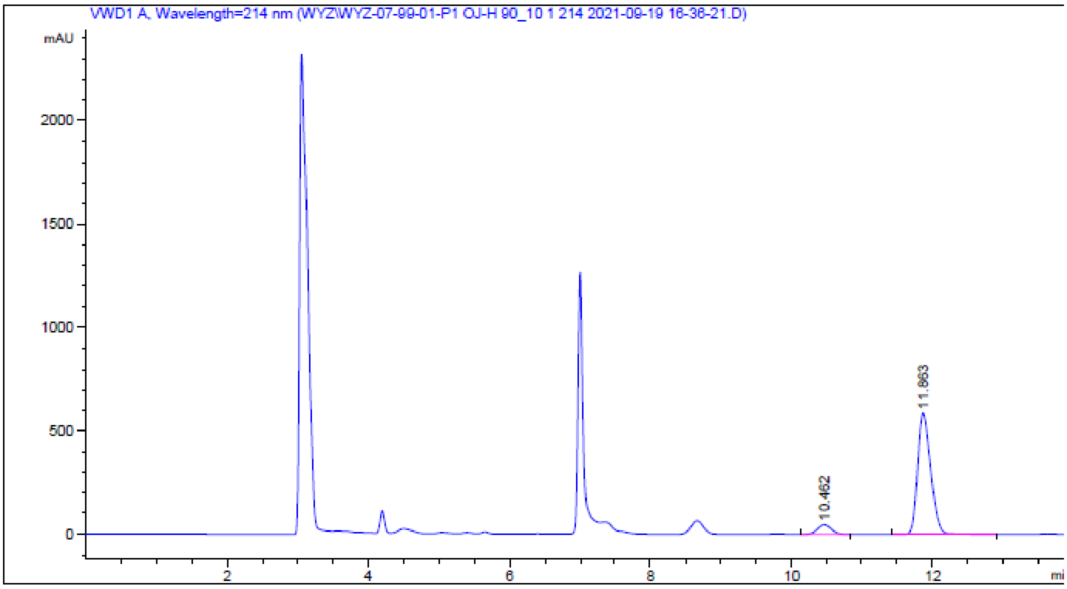

Peak NO	Ret. Time (min)	Area/%
1	10.462	7.9834
2	11.863	92.0166

methyl (*R*)-3-(1-phenylpropyl)benzoate (3l)[5]

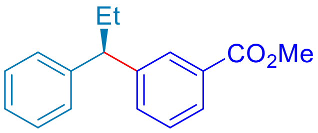

Prepared according to the general procedure **1** from (1-chloropropyl)benzene (0.4 mmol, 2 equiv.) and **2** from methyl 3-bromobenzoate (0.2 mmol, 1 equiv.). The title compound was isolated (gradient 0–5% EtOAc/hexanes) as a colorless oil (45.7 mg, 90% yield, 80% ee).

^{1}H NMR (400 MHz, $CDCl_3$) δ 7.95 (s, 1H), 7.85 (d, *J* = 7.6 Hz, 1H), 7.41 (d, *J* = 7.6 Hz, 1H), 7.34 (d, *J* = 7.6 Hz, 1H), 7.32 – 7.20 (m, 4H), 7.17 (t, *J* = 7.2 Hz, 1H), 3.88 (d, *J* = 8.0 Hz, 3H), 3.84 (t, *J* = 7.6 Hz, 1H), 2.10 (p, *J* = 7.2 Hz, 2H), 0.89 (t, *J* = 7.2 Hz, 3H).

^{13}C NMR (101 MHz, $CDCl_3$) δ 167.3, 145.6, 144.6, 132.6, 130.2, 129.0, 128.5, 128.5, 127.9, 127.4, 126.3, 53.1, 52.1, 28.5, 12.7.

$[\alpha]_D$ ^{25} = -5.36 (*c* = 0.2, $CHCl_3$).

Enantiomeric excess = 80%, determined by HPLC (Daicel Chiralpak OJ-H Column, *n*-Hexane:*i*-PrOH = 90:10, flow rate 1.0 mL/min, T = 25 °C, λ = 214 nm): t_R = 10.245 min (minor), t_R = 12.391 min (major).

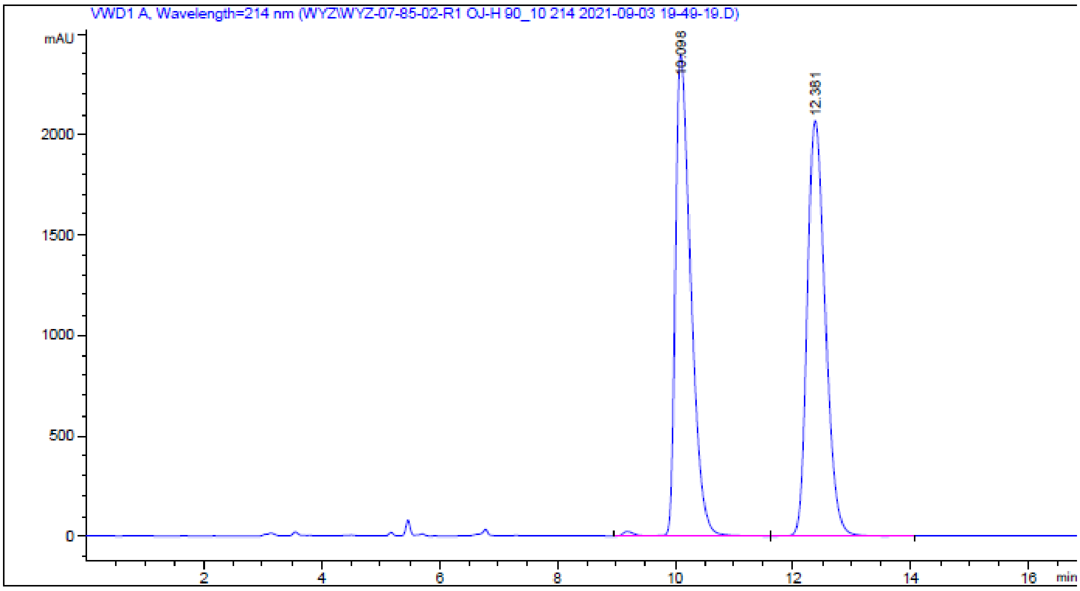

Peak NO	Ret. Time (min)	Area/%
1	10.098	49.9150
2	12.381	50.0850

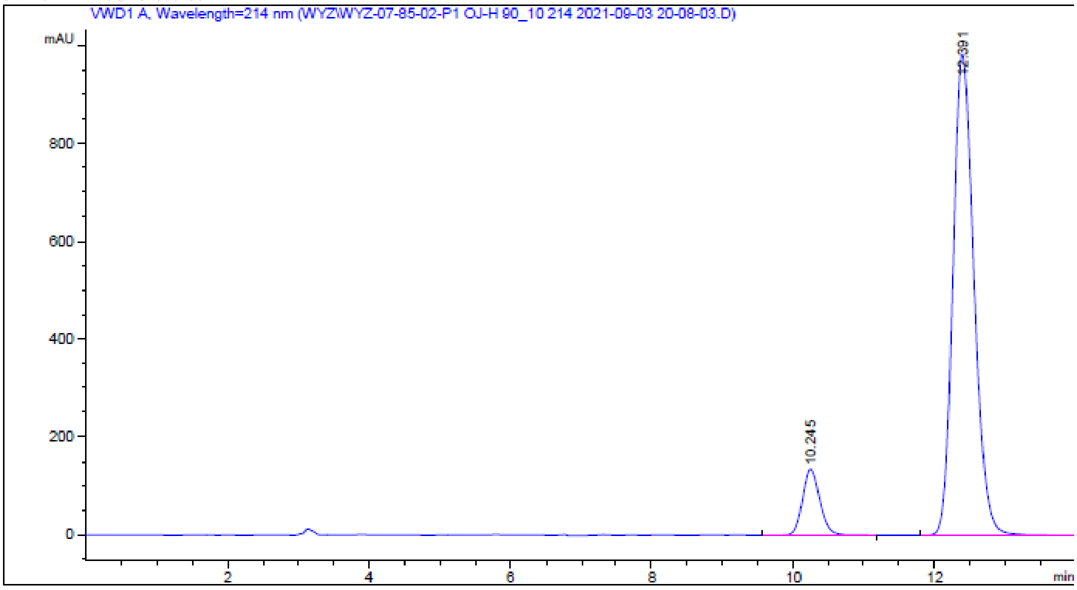

Peak NO	Ret. Time (min)	Area/%
1	10.245	10.1725
2	12.391	89.8275

(*R*)-3-(1-phenylpropyl)benzonitrile (3m)[2]

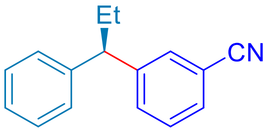

Prepared according to the general procedure **1** from (1-chloropropyl)benzene (0.4 mmol, 2 equiv.) and **2** from 3-bromobenzonitrile (0.2 mmol, 1 equiv.). The title compound was isolated (gradient 0–5% EtOAc/hexanes) as a colorless oil (31.7 mg, 72% yield, 73% ee).

^{1}H NMR (400 MHz, $CDCl_3$) δ 7.52 (s, 1H), 7.46 (d, J = 7.6 Hz, 2H), 7.37 (d, J = 7.6 Hz, 1H), 7.32 – 7.30 (m, 2H), 7.21 – 7.19 (m, 3H), 3.90 – 3.75 (m, 1H), 2.17 – 1.96 (m, 2H), 0.90 (t, J = 7.2 Hz, 3H).

^{13}C NMR (101 MHz, $CDCl_3$) δ 146.7, 143.6, 132.6, 131.5, 129.9, 129.2, 128.7, 127.8, 126.6, 119.1, 112.4, 52.8, 28.3, 12.6.

$[\alpha]_D^{23}$ = -5.11 (c = 0.3, $CHCl_3$).

Enantiomeric excess = 73%, determined by HPLC (Daicel Chiralpak OJ-H Column, n-Hexane:i-PrOH = 90:10, flow rate 1.0 mL/min, T = 25 °C, λ = 214 nm): t_R = 11.864 min (minor), t_R = 13.456 min (major).

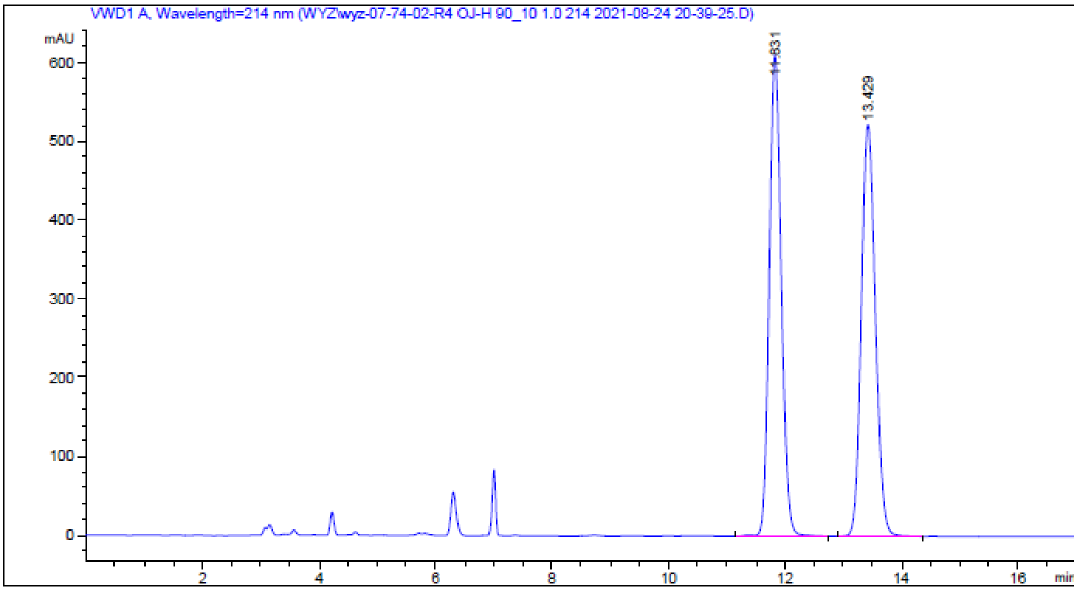

Peak NO	Ret. Time (min)	Area/%
1	11.831	50.0578
2	13.429	49.9422

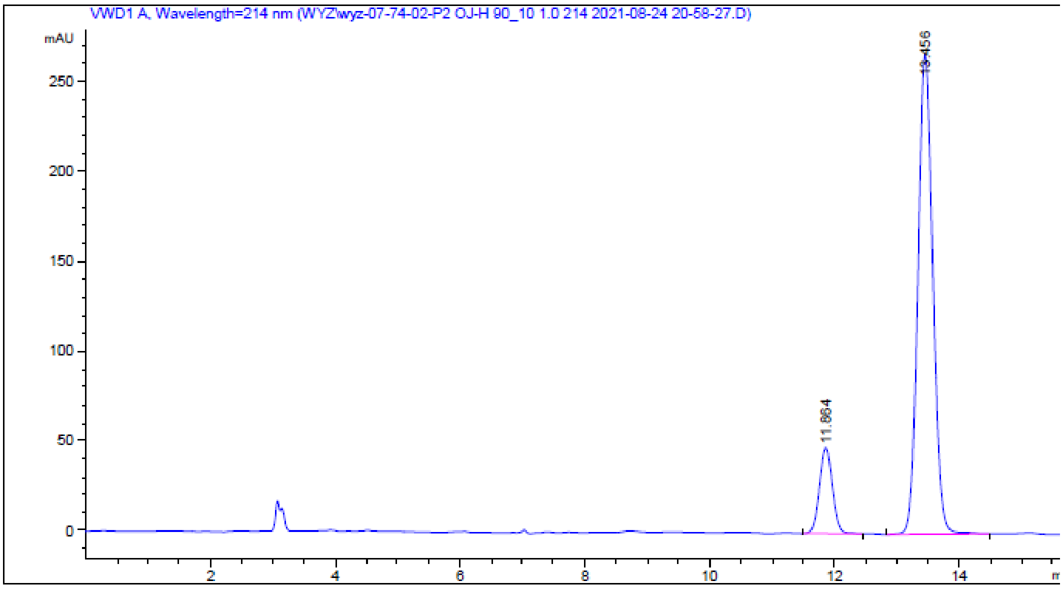

Peak NO	Ret. Time (min)	Area/%
1	11.864	13.3654
2	13.456	86.6346

(*R*)-3-(1-phenylpropyl)-1,1'-biphenyl (3n)[3]

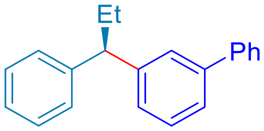

Prepared according to the general procedure **1** from (1-chloropropyl)benzene (0.4 mmol, 2 equiv.) and **2** from 3-bromo-1,1'-biphenyl (0.2 mmol, 1 equiv.). The title compound was isolated (gradient 0–5% EtOAc/hexanes) as a colorless oil (41.3 mg, 76% yield, 88% ee).

^{1}H NMR (400 MHz, $CDCl_3$) δ 7.70 – 7.60 (m, 2H), 7.55 (d, *J* = 1.6 Hz, 1H), 7.53 – 7.45 (m, 3H), 7.41 (qd, *J* = 7.2, 1.2 Hz, 2H), 7.38 – 7.33 (m, 4H), 7.32 – 7.22 (m, 2H), 3.94 (t, *J* = 7.6 Hz, 1H), 2.27 – 2.15 (m, 2H), 1.01 (td, *J* = 7.2, 1.6 Hz, 3H).

^{13}C NMR (101 MHz, $CDCl_3$) δ 145.7, 145.1, 141.5, 141.3, 128.9, 128.8, 128.5, 128.0, 127.3, 127.3, 126.9, 126.2, 125.0, 53.5, 28.7, 12.9.

$[\alpha]_D$ ^{25} = -3.41 (*c* = 0.3, $CHCl_3$).

Enantiomeric excess = 88%, determined by HPLC (Daicel Chiralpak OJ-H Column, *n*-Hexane:*i*-PrOH = 95:5, flow rate 0.7 mL/min, T = 25 °C, λ = 214 nm): t_R = 12.788 min (minor), t_R = 14.011 min (major).

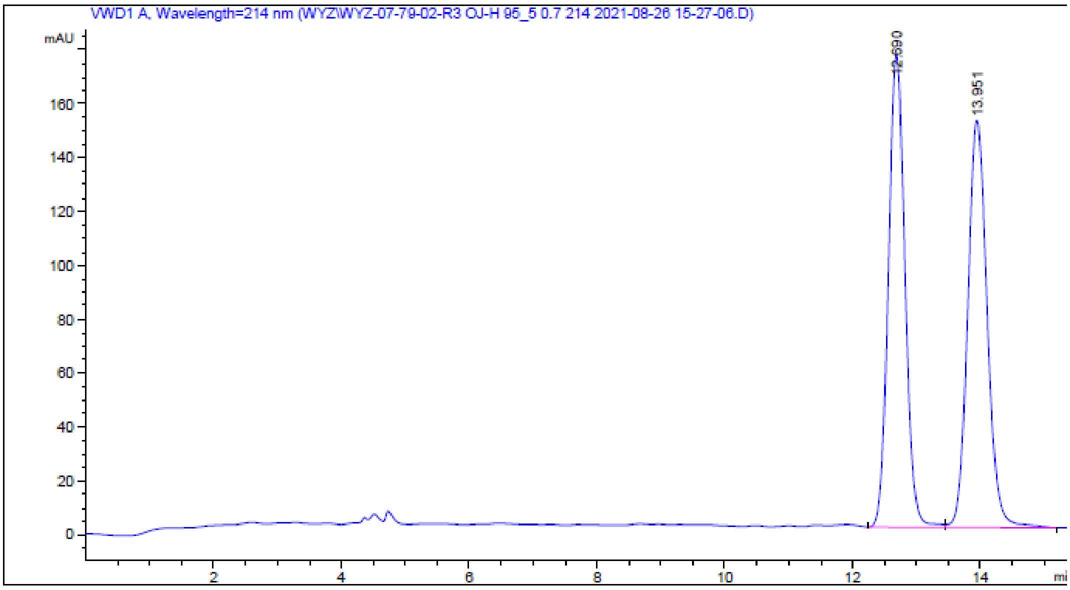

Peak NO	Ret. Time (min)	Area/%
1	12.690	49.7751
2	13.951	50.2249

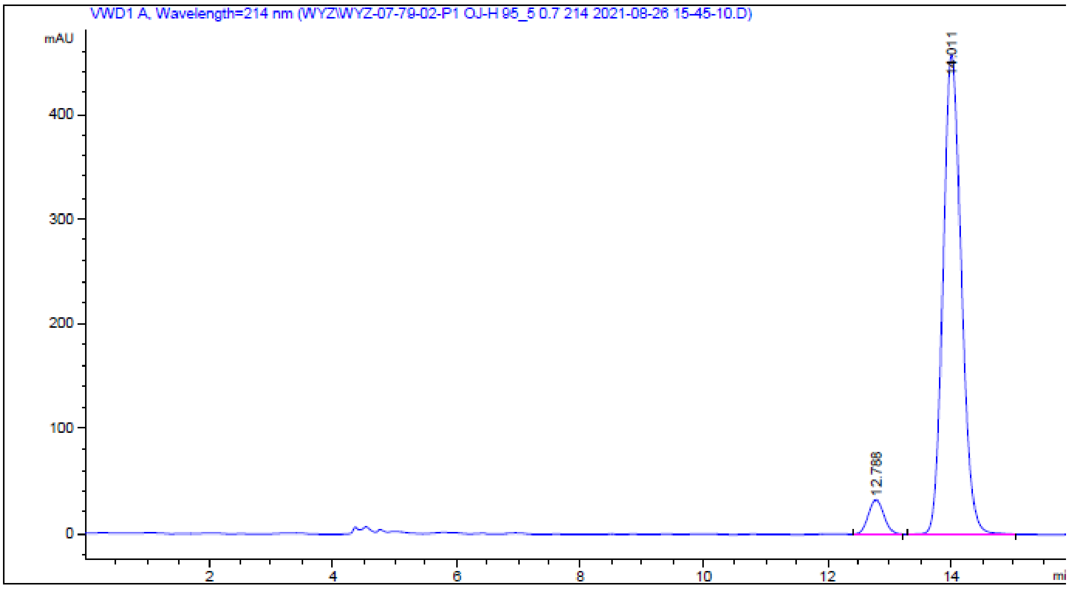

Peak NO	Ret. Time (min)	Area/%
1	12.788	5.8495
2	14.011	94.1505

(*R*)-2-(1-phenylpropyl)naphthalene (3o)[6]

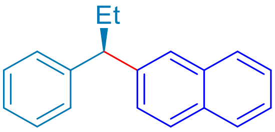

Prepared according to the general procedure **1** from (1-chloropropyl)benzene (0.4 mmol, 2 equiv.) and **2** from 2-bromonaphthalene (0.2 mmol, 1 equiv.). The title compound was isolated (gradient 0–5% EtOAc/hexanes) as a yellow oil (39.0 mg, 79% yield, 90% ee).

^{1}H NMR (400 MHz, $CDCl_3$) δ 7.93 – 7.69 (m, 4H), 7.56 – 7.43 (m, 2H), 7.39 (dt, *J* = 8.4, 2.4 Hz, 1H), 7.37 – 7.30 (m, 4H), 7.27 – 7.18 (m, 1H), 4.02 (td, *J* = 7.6, 2.8 Hz, 1H), 2.27 – 2.21 (m, 2H), 1.00 (td, *J* = 7.2, 3.2 Hz, 3H).

^{13}C NMR (101 MHz, $CDCl_3$) δ 145.1, 142.6, 133.6, 132.2, 128.4, 128.1, 128.0, 127.8, 127.6, 126.9, 126.1, 126.0, 125.9, 125.4, 53.3, 28.4, 12.9.

$[\alpha]_D^{26}$ = +5.96 (*c* = 0.2, $CHCl_3$).

Enantiomeric excess = 90%, determined by HPLC (Daicel Chiralpak AD-H Column, *n*-Hexane:*i*-PrOH = 95:5, flow rate 0.5 mL/min, T = 25 °C, λ = 214 nm): t_R = 8.482 min (minor), t_R = 8.978 min (major).

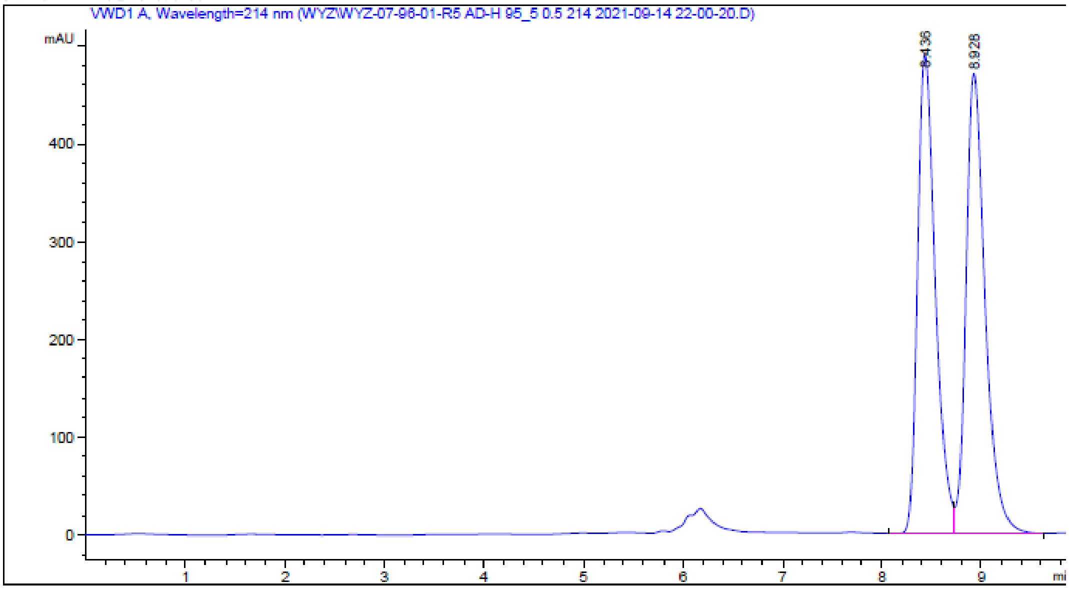

Peak NO	Ret. Time (min)	Area/%
1	8.436	49.1114
2	8.928	50.8886

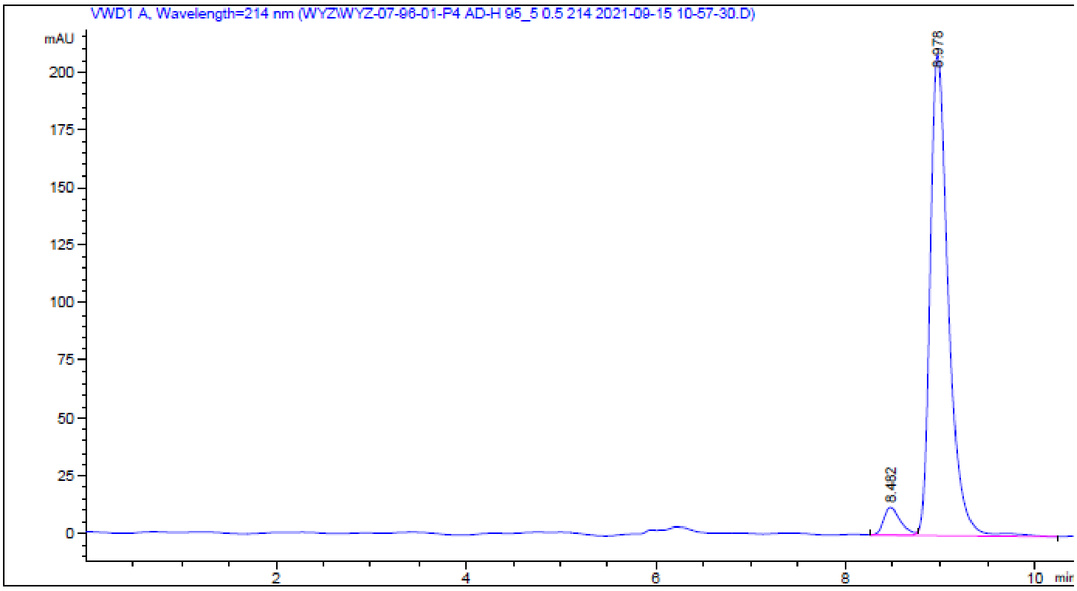

Peak NO	Ret. Time (min)	Area/%
1	8.482	4.9935
2	8.978	95.0065

(*R*)-2-methoxy-6-(1-phenylpropyl)naphthalene (3p)[5]

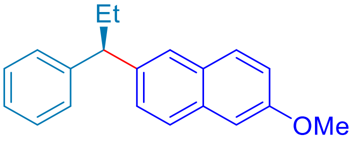

Prepared according to the general procedure **1** from (1-chloropropyl)benzene (0.4 mmol, 2 equiv.) and **2** from 2-bromo-6-methoxynaphthalene (0.2 mmol, 1 equiv.). The title compound was isolated (gradient 0–5% EtOAc/hexanes) as a white soild (49.7 mg, 90% yield, 86% ee).

M. P.: 138.1 – 142.5 °C

^{1}H NMR (400 MHz, $CDCl_3$) δ 7.68 – 7.61 (m, 3H), 7.32 – 7.19 (m, 5H), 7.19 – 7.00 (m, 3H), 3.98 – 3.75 (m, 4H), 2.19 – 2.11 (m, 2H), 0.92 (t, *J* = 7.2 Hz, 3H).

^{13}C NMR (101 MHz, $CDCl_3$) δ 157.4, 145.3, 140.4, 133.2, 129.2, 129.0, 128.4, 128.1, 127.4, 126.9, 126.1, 125.8, 118.7, 105.7, 55.3, 53.1, 28.5, 12.9.

$[\alpha]_D$ ^{26} = +1.03 (*c* = 0.6, $CHCl_3$).

Enantiomeric excess = 86%, determined by HPLC (Daicel Chiralpak OJ-H Column, *n*-Hexane:*i*-PrOH = 90:10, flow rate 1.0 mL/min, T = 25 °C, λ = 214 nm): t_R = 16.652 min (minor), t_R = 19.050 min (major).

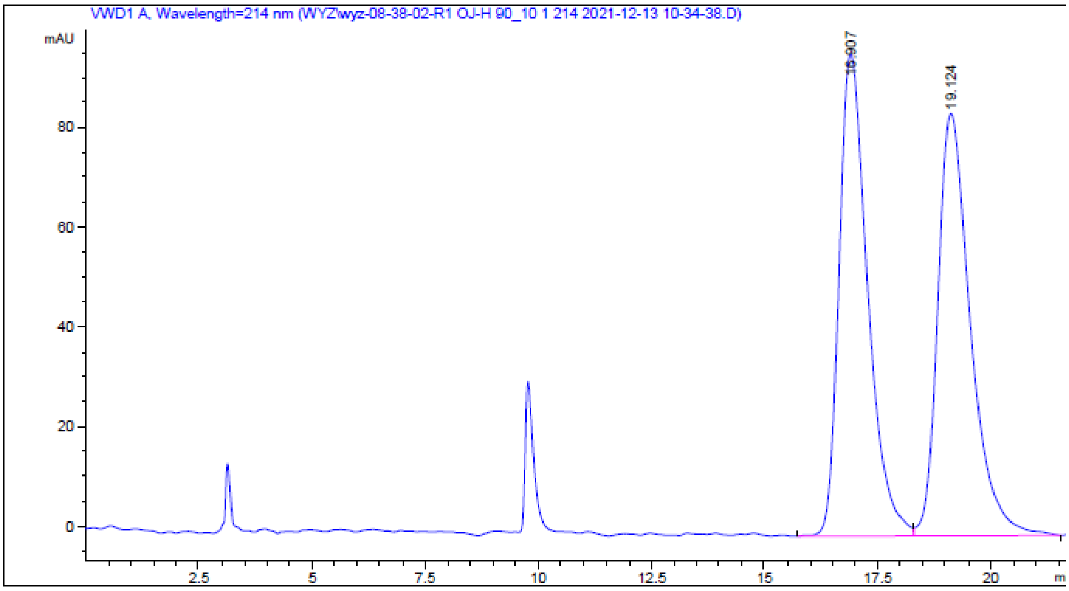

Peak NO	Ret. Time (min)	Area/%
1	16.907	49.9963
2	19.124	50.0037

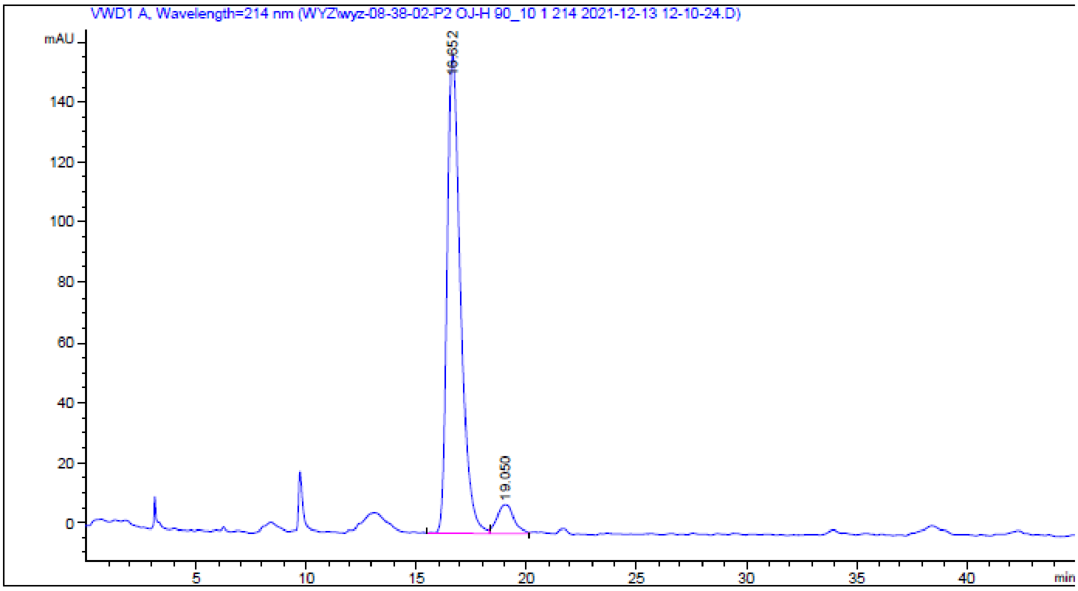

Peak NO	Ret. Time (min)	Area/%
1	16.652	93.0169
2	19.050	6.9831

(*R*)-2-(1-phenylpropyl)-9H-fluorene (3q)

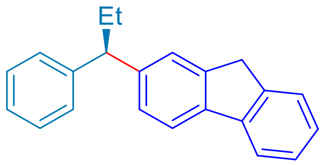

Prepared according to the general procedure **1** from (1-chloropropyl)benzene (0.4 mmol, 2 equiv.) and **2** from 2-bromo-9*H*-fluorene (0.2 mmol, 1 equiv.). The title compound was isolated (gradient 0–5% EtOAc/hexanes) as a yellow oil (42.7 mg, 75% yield, 91% ee)。

^{1}H NMR (400 MHz, $CDCl_3$) δ 7.78 (d, *J* = 7.6 Hz, 1H), 7.73 (d, *J* = 7.6 Hz, 1H), 7.55 (d, *J* = 7.2 Hz, 1H), 7.45 (s, 1H), 7.41 – 7.28 (m, 7H), 7.26 – 7.20 (m, 1H), 3.98 – 3.82 (m, 3H), 2.18 (p, *J* = 7.2 Hz, 2H), 0.98 (t, *J* = 7.2 Hz, 3H).

^{13}C NMR (101 MHz, $CDCl_3$) δ 145.4, 144.0, 143.6, 143.3, 141.7, 139.8, 128.4, 128.0, 126.7, 126.7, 126.3, 126.1, 125.0, 124.6, 119.7, 119.7, 53.4, 36.9, 28.8, 12.9.

IR (neat): 3739, 2957, 1491, 1455, 832, 797, 734, 699, 643, 624 cm^{-1}.

HRMS (EI) calcd for $C_{22}H_{20}$ $[M]^+$:284.156; found: 284.156.

$[\alpha]_D$ ^{26} = +1.89 (*c* = 0.2, $CHCl_3$).

Enantiomeric excess = 91%, determined by HPLC (Daicel Chiralpak OJ-H Column, *n*-Hexane:*i*-PrOH = 90:10, flow rate 1.0 mL/min, T = 25 °C, λ = 214 nm): t_R = 9.176 min (minor), t_R = 13.094 min (major).

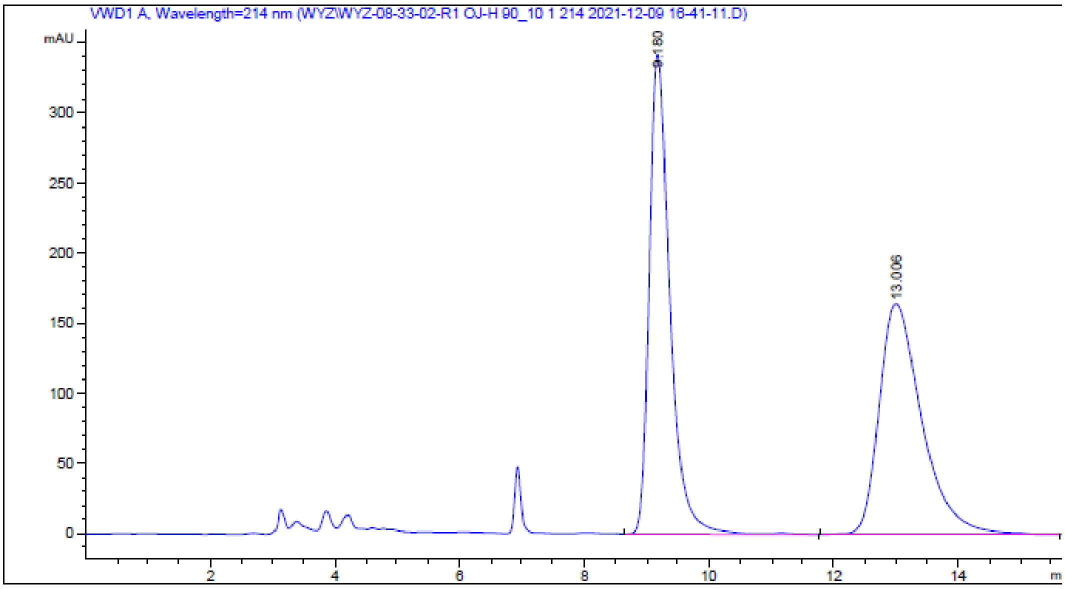

Peak NO	Ret. Time (min)	Area/%
1	9.180	50.1204
2	13.006	49.8796

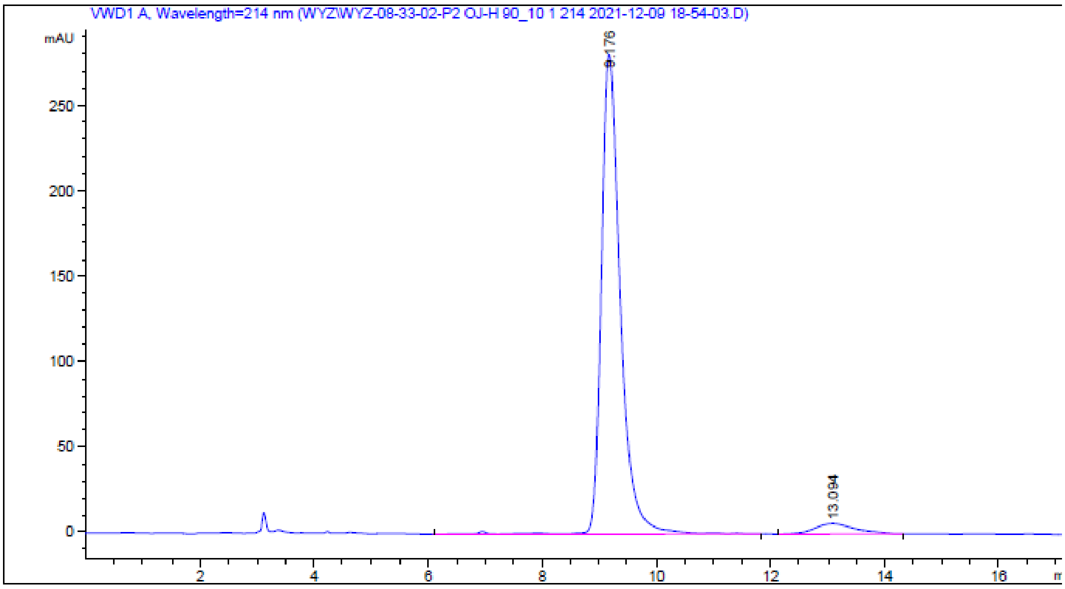

Peak NO	Ret. Time (min)	Area/%
1	9.176	95.5185
2	13.094	4.4815

(*R*)-5-(1-phenylpropyl)benzofuran (3r) [2]

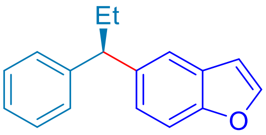

Prepared according to the general procedure **1** from (1-chloropropyl)benzene (0.4 mmol, 2 equiv.) and **2** from 5-bromobenzofuran (0.2 mmol, 1 equiv.). The title compound was isolated (gradient 0–5% EtOAc/hexanes) as a yellow oil (19.4 mg, 41% yield, 90% ee).

^{1}H NMR (400 MHz, CDCl$_3$) δ 7.57 (d, *J* = 2.0 Hz, 1H), 7.46 (d, *J* = 1.2 Hz, 1H), 7.39 (d, *J* = 8.4 Hz, 1H), 7.27 – 7.25 (m, 4H), 7.17 – 7.15 (m, 2H), 6.70 (d, *J* = 1.2 Hz, 1H), 3.89 (t, *J* = 7.6 Hz, 1H), 2.12 (p, *J* = 7.2 Hz, 2H), 0.91 (t, *J* = 7.2 Hz, 3H).

^{13}C NMR (101 MHz, CDCl$_3$) δ 153.6, 145.6, 145.1, 139.8, 128.4, 127.9, 127.5, 126.0, 124.6, 120.0, 111.1, 106.6, 53.1, 29.0, 12.9.

$[\alpha]_D$ ^{27} = -17.44 (*c* = 0.2, CHCl$_3$).

Enantiomeric excess = 90%, determined by HPLC (Daicel Chiralpak OJ-H Column, *n*-Hexane:*i*-PrOH = 90:10, flow rate 1.0 mL/min, T = 25 °C, λ = 214 nm): t_R = 13.881 min (minor), t_R = 17.509 min (major).

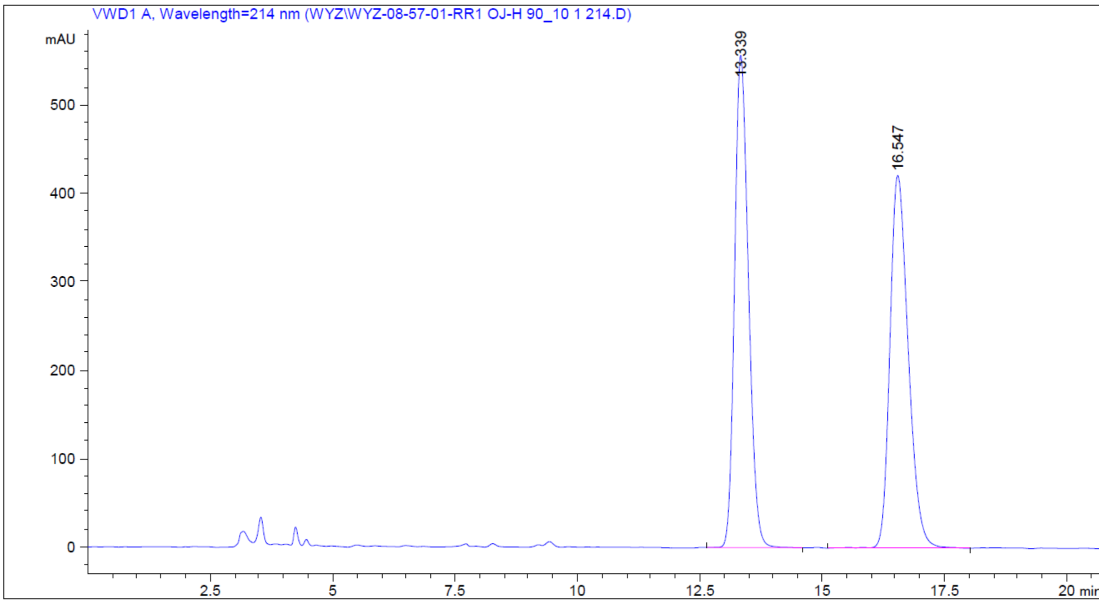

Peak NO	Ret. Time (min)	Area/%
1	13.339	49.9978
2	16.547	50.0022

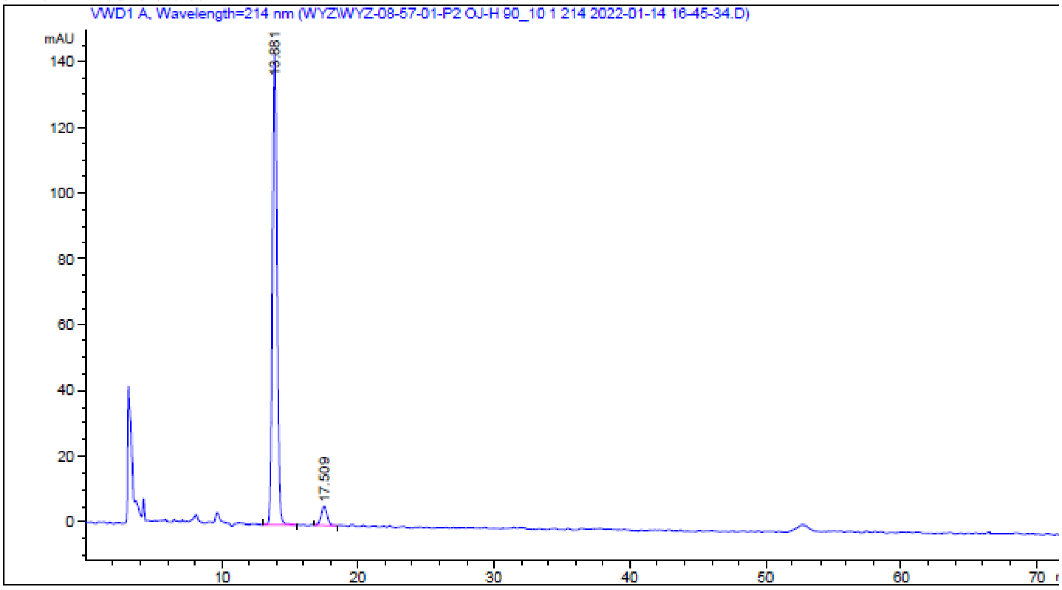

Peak NO	Ret. Time (min)	Area/%
1	13.881	94.8388
2	17.509	5.1612

(*R*)-6-(1-phenylpropyl)benzofuran (3s)

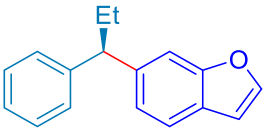

Prepared according to the general procedure **1** from (1-chloropropyl)benzene (0.4 mmol, 2 equiv.) and **2** from 6-bromobenzofuran (0.2 mmol, 1 equiv.). The title compound was isolated (gradient 0–5% EtOAc/hexanes) as a yellow oil (19.4 mg, 41% yield, 89% ee).

^{1}H NMR (400 MHz, $CDCl_3$) δ 7.55 (d, *J* = 2.0 Hz, 1H), 7.47 (d, *J* = 8.0 Hz, 1H), 7.39 (s, 1H), 7.30 – 7.23 (m, 4H), 7.20 – 7.14 (m, 1H), 7.12 (d, *J* = 8.0 Hz, 1H), 6.69 (d, *J* = 1.2 Hz, 1H), 3.90 (t, *J* = 7.6 Hz, 1H), 2.12 (p, *J* = 7.2 Hz, 2H), 0.92 (t, *J* = 7.2 Hz, 3H).

^{13}C NMR (101 MHz, $CDCl_3$) δ 155.4, 145.3, 144.7, 142.1, 128.4, 127.9, 126.1, 125.4, 123.3, 120.8, 110.4, 106.4, 53.3, 28.8, 12.8.

IR (neat): 2923, 1454, 1260, 1129, 1026, 801, 758, 733, 699, 662 cm^{-1}.

HRMS (EI) calcd for $C_{17}H_{16}O$ $[M]^+$:263.1198; found: 263.1196.

$[\alpha]_D$ ^{24} = -1.49 (*c* = 0.2, $CHCl_3$).

Enantiomeric excess = 89%, determined by HPLC (Daicel Chiralpak AD-H Column, *n*-Hexane:*i*-PrOH = 95:5, flow rate 0.7 mL/min, T = 25 °C, λ = 214 nm): t_R = 5.957 min (minor), t_R = 6.319 min (major).

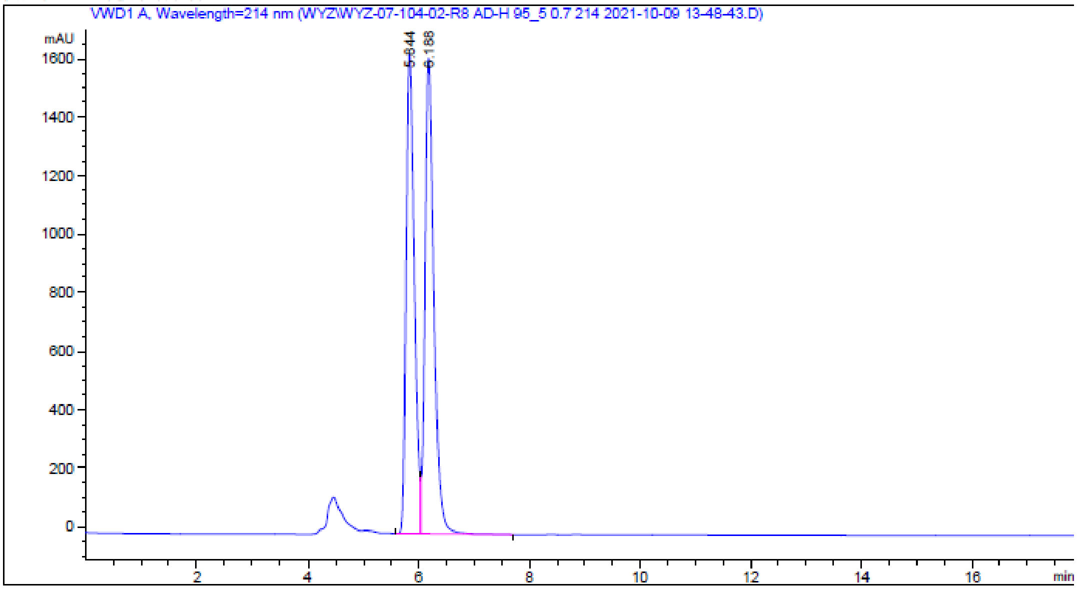

Peak NO	Ret. Time (min)	Area/%
1	5.844	47.9389
2	6.188	52.0611

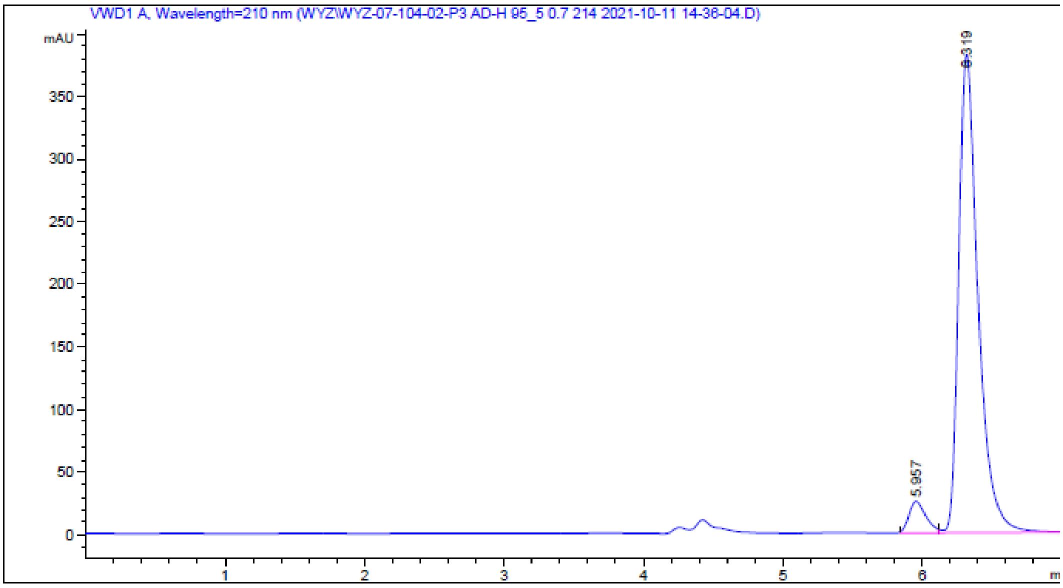

Peak NO	Ret. Time (min)	Area/%
1	5.957	5.4551
2	6.319	94.5449

(*R*)-6-(1-phenylpropyl)benzo[b]thiophene (3t)

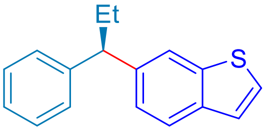

Prepared according to the general procedure **1** from (1-chloropropyl)benzene (0.4 mmol, 2 equiv.) and **2** from 6-bromobenzo[*b*]thiophene (0.2 mmol, 1 equiv.). The title compound was isolated (gradient 0–5% EtOAc/hexanes) as a yellow oil (43.4 mg, 86% yield, 86% ee).

^{1}H NMR (400 MHz, $CDCl_3$) δ 7.78 – 7.64 (m, 2H), 7.36 – 7.11 (m, 8H), 3.91 (dd, *J* = 10.4, 4.8 Hz, 1H), 2.17 – 2.05 (m, 2H), 0.91 (td, *J* = 7.2, 2.4 Hz, 3H).

^{13}C NMR (101 MHz, $CDCl_3$) δ 145.2, 141.7, 140.1, 138.0, 128.5, 128.0, 126.2, 125.7, 125.0, 123.6, 123.5, 121.3, 53.3, 28.8, 12.9.

IR (neat): 2959, 1260, 1085, 1019, 799, 752, 698 cm^{-1}.

HRMS (EI) calcd for $C_{17}H_{16}S$ $[M]^{+}$:252.0969; found: 252.0967.

$[\alpha]_D$ ^{27} = -19.82 (*c* = 0.2, $CHCl_3$).

Enantiomeric excess = 86%, determined by HPLC (Daicel Chiralpak OJ-H Column, *n*-Hexane:*i*-PrOH = 90:10, flow rate 1.0 mL/min, T = 25 °C, λ = 214 nm): t_R = 9.496 min (minor), t_R = 10.339 min (major).

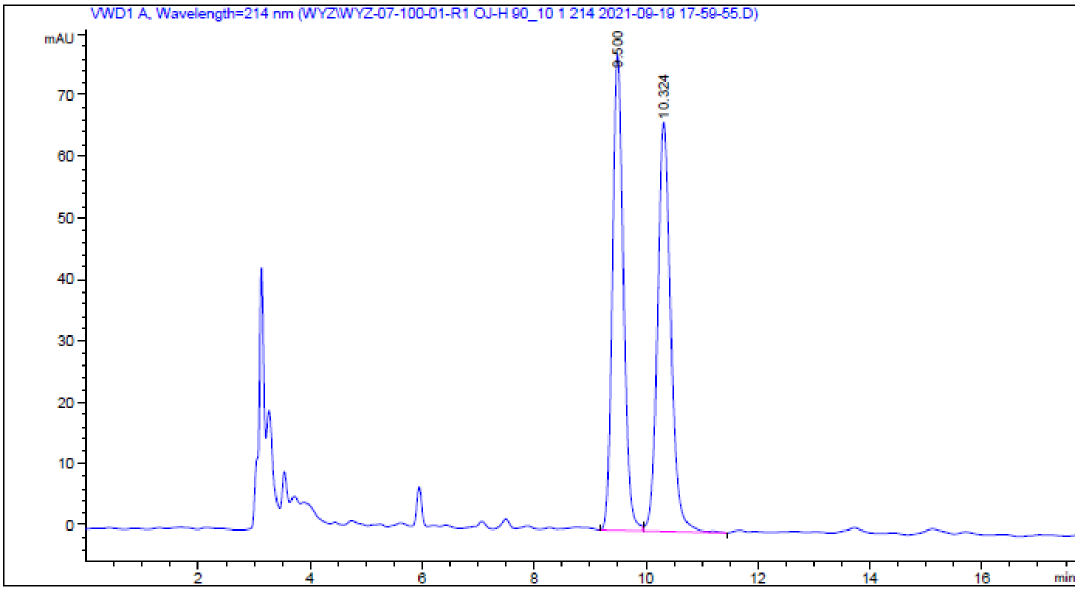

Peak NO	Ret. Time (min)	Area/%
1	9.500	49.0892
2	10.324	50.9108

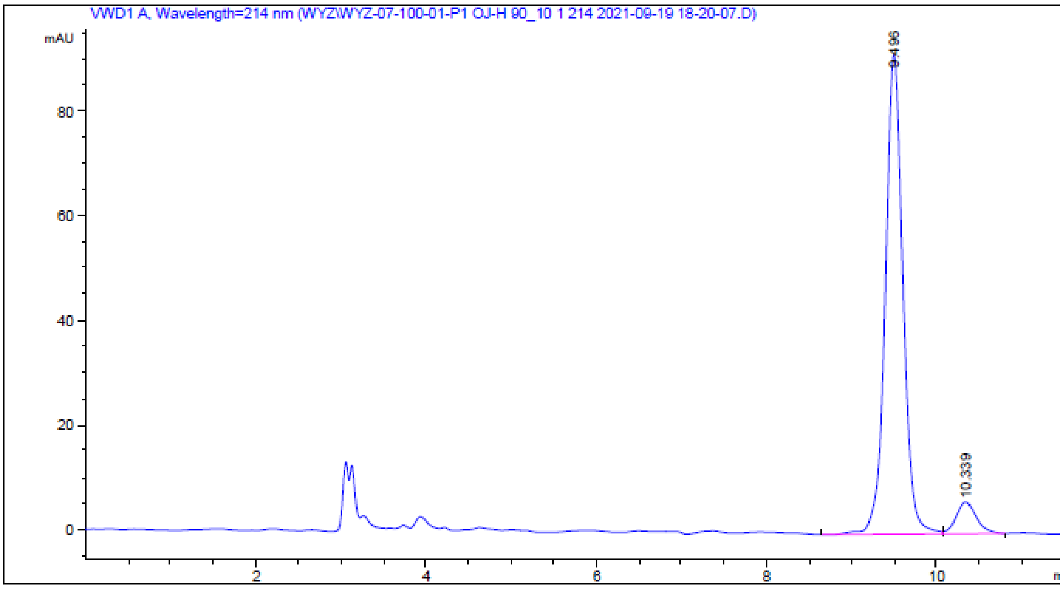

Peak NO	Ret. Time (min)	Area/%
1	9.496	92.9288
2	10.339	7.0712

tert-butyl (_R_)-6-(1-phenylpropyl)-1H-indole-1-carboxylate (3u) [2]

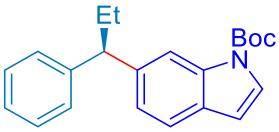

Prepared according to the general procedure **1** from (1-chloropropyl)benzene (0.4 mmol, 2 equiv.) and **2** from tert-butyl 6-bromo-1*H*-indole-1-carboxylate (0.2 mmol, 1 equiv.). The title compound was isolated (gradient 0–5% EtOAc/hexanes) as a yellow oil (28.9 mg, 43% yield, 81% ee).

^{1}H NMR (400 MHz, $CDCl_3$) δ 8.11 (s, 1H), 7.56 (d, *J* = 3.2 Hz, 1H), 7.47 (d, *J* = 8.0 Hz, 1H), 7.34 – 7.26 (m, 4H), 7.23 – 7.11 (m, 2H), 6.52 (d, *J* = 3.6 Hz, 1H), 3.95 (t, *J* = 7.6 Hz, 1H), 2.17 (p, *J* = 7.2 Hz, 2H), 1.68 (s, 9H), 0.95 (t, *J* = 7.2 Hz, 3H).

^{13}C NMR (101 MHz, $CDCl_3$) δ 158.1, 145.7, 141.7, 128.8, 128.4, 127.9, 125.9, 125.7, 123.1, 121.0, 120.7, 114.4, 107.1, 83.5, 53.7, 28.9, 28.2, 12.9.

$[\alpha]_D^{27}$ = -33.65 (*c* = 0.1, $CHCl_3$).

Enantiomeric excess = 81%, determined by HPLC (Daicel Chiralpak OJ-H Column, *n*-Hexane:*i*-PrOH = 90:10, flow rate 1.0 mL/min, T = 25 °C, λ = 214 nm): t_R = 5.806 min (minor), t_R = 8.330 min (major).

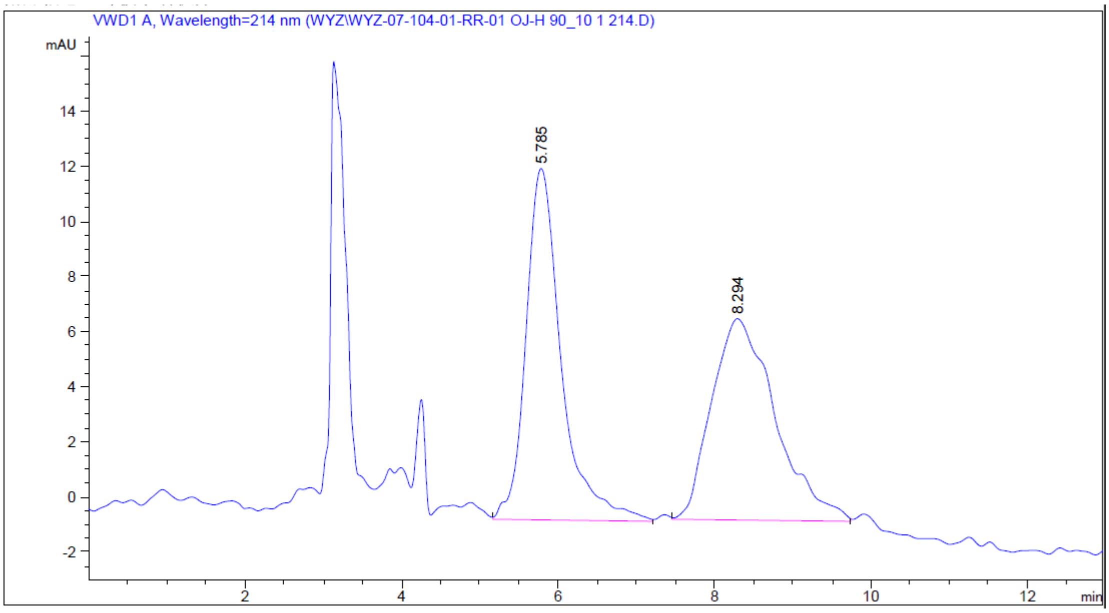

Peak NO	Ret. Time (min)	Area/%
1	5.785	49.1968
2	8.294	50.8032

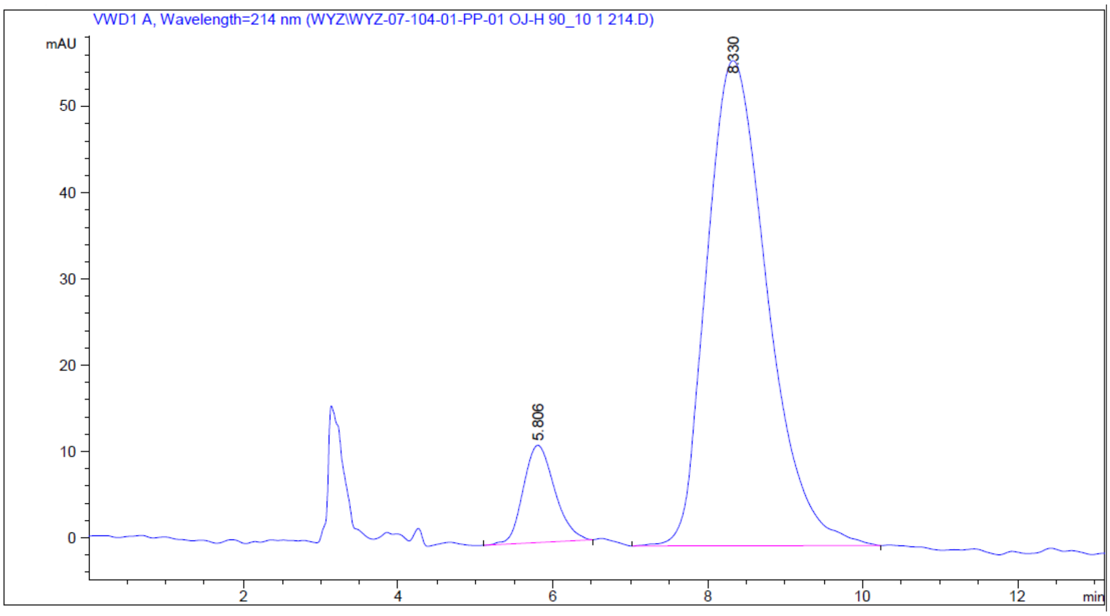

Peak NO	Ret. Time (min)	Area/%
1	5.806	9.3919
2	8.330	90.6081

(*R*)-3-(1-phenylpropyl)dibenzo[b,d]thiophene (3v)

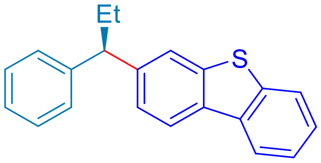

Prepared according to the general procedure **1** from (1-chloropropyl)benzene (0.4 mmol, 2 equiv.) and **2** from 3-bromodibenzo[*b*,*d*]thiophene (0.2 mmol, 1 equiv.). The title compound was isolated (gradient 0–5% EtOAc/hexanes) as a colorless oil (43.8 mg, 72% yield, 87% ee).

^{1}H NMR (400 MHz, $CDCl_3$) δ 8.15 – 8.13 (m, 1H), 8.10 (d, *J* = 8.0 Hz, 1H), 7.89 – 7.86 (m, 1H), 7.78 (s, 1H), 7.51 – 7.44 (m, 2H), 7.43 – 7.31 (m, 5H), 7.28 – 7.21 (m, 1H), 4.01 (t, *J* = 7.6 Hz, 1H), 2.22 (p, *J* = 7.2 Hz, 2H), 1.01 (t, *J* = 7.2 Hz, 3H).

^{13}C NMR (101 MHz, $CDCl_3$) δ 144.9, 144.3, 139.8, 139.4, 135.5, 133.8, 128.5, 128.0, 126.4, 126.3, 124.9, 124.4, 122.8, 121.8, 121.5, 121.4, 53.4, 28.7, 12.9.

IR (neat): 2960, 1452, 1263, 1075, 1022, 797, 736, 701 cm^{-1}.

HRMS (EI) calcd for $C_{21}H_{18}S$ $[M]^+$:302.1124; found: 302.1133.

$[\alpha]_D$ ^{27} = -8.29 (*c* = 0.5, $CHCl_3$).

Enantiomeric excess = 87%, determined by HPLC (Daicel Chiralpak OJ-H Column, *n*-Hexane:*i*-PrOH = 90:10, flow rate 1.0 mL/min, T = 25 °C, λ = 214 nm): t_R = 10.308 min (minor), t_R = 12.270 min (major).

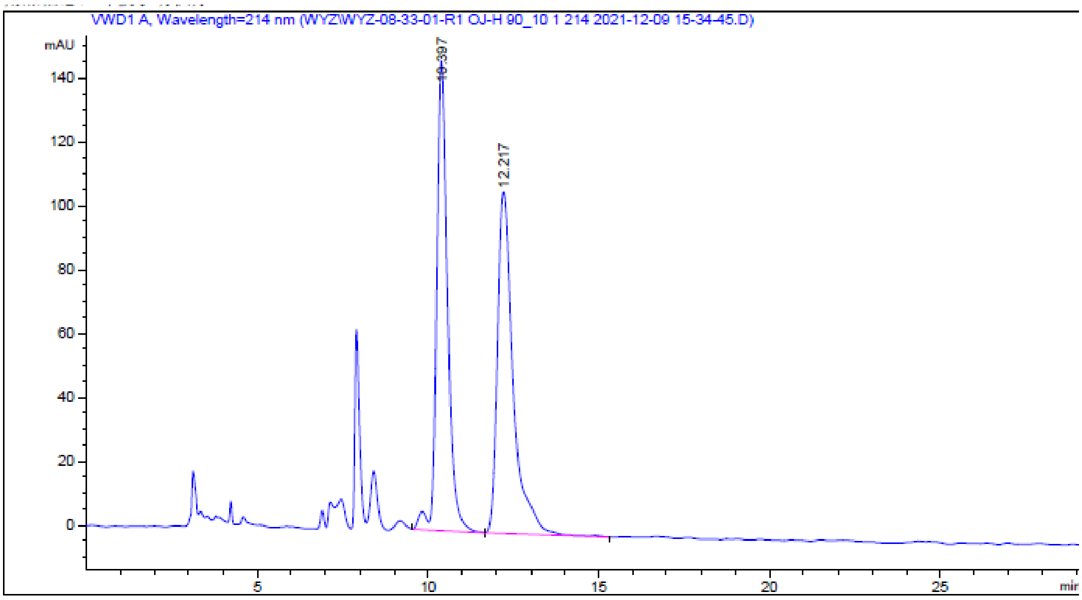

Peak NO	Ret. Time (min)	Area/%
1	10.397	49.0789
2	12.217	50.9211

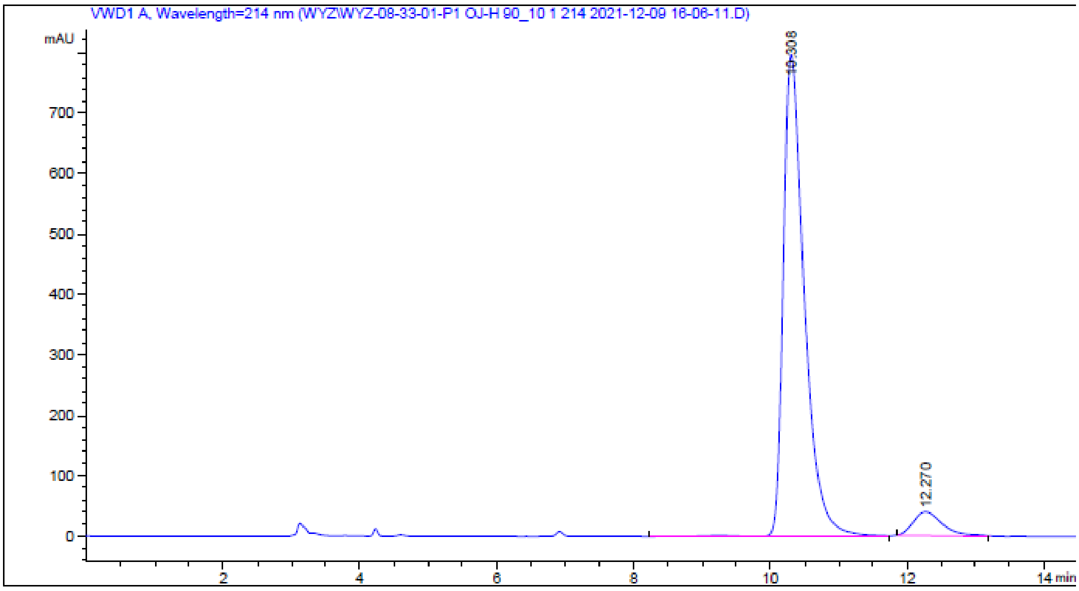

Peak NO	Ret. Time (min)	Area/%
1	10.308	93.5801
2	12.270	6.4199

(*R*)-1-(4-(1-phenylpropyl)phenyl)-1*H*-pyrrole (3w)

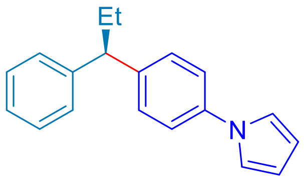

Prepared according to the general procedure **1** from (1-chloropropyl)benzene (0.4 mmol, 2 equiv.) and **2** from 1-(4-bromophenyl)-1*H*-pyrrole (0.2 mmol, 1 equiv.). The title compound was isolated (gradient 0–5% EtOAc/hexanes) as a reddish brown oil (48.1 mg, 92% yield, 87% ee).

^{1}H NMR (400 MHz, $CDCl_3$) δ 7.39 – 7.28 (m, 8H), 7.28 – 7.20 (m, 1H), 7.10 (t, *J* = 2.0 Hz, 2H), 6.52 – 6.27 (m, 2H), 3.88 (t, *J* = 7.6 Hz, 1H), 2.15 (p, *J* = 7.2 Hz, 2H), 0.98 (t, *J* = 7.2 Hz, 3H).

^{13}C NMR (101 MHz, $CDCl_3$) δ 144.9, 142.8, 138.9, 129.0, 128.5, 127.9, 126.3, 120.6, 119.4, 110.2, 52.7, 28.7, 12.8.

IR (neat): 2959, 1519, 1329, 1263, 1071, 1020, 801, 738, 701 cm^{-1}.

HRMS (EI) calcd for $C_{19}H_{19}N$ $[M]^{+}$:261.1513; found: 261.1512.

$[\alpha]_D^{26}$ = -14.32 (*c* = 0.3, $CHCl_3$).

Enantiomeric excess = 87%, determined by HPLC (Daicel Chiralpak OJ-H Column, *n*-Hexane:*i*-PrOH = 90:10, flow rate 1.0 mL/min, T = 25 °C, λ = 214 nm): t_R = 8.668 min (minor), t_R = 9.945 min (major).

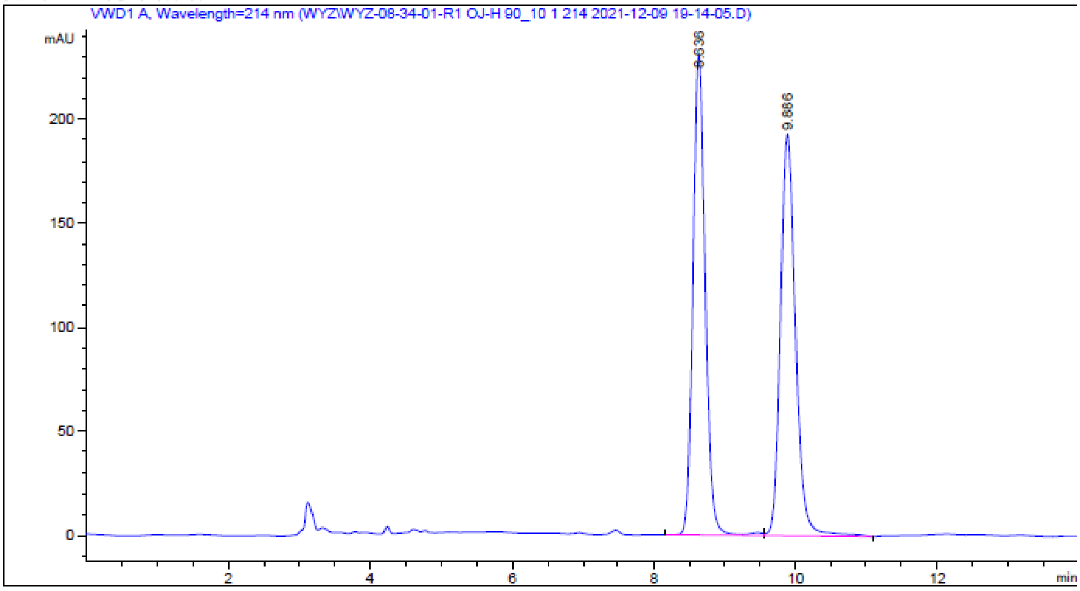

Peak NO	Ret. Time (min)	Area/%
1	8.636	49.5366
2	9.886	50.4634

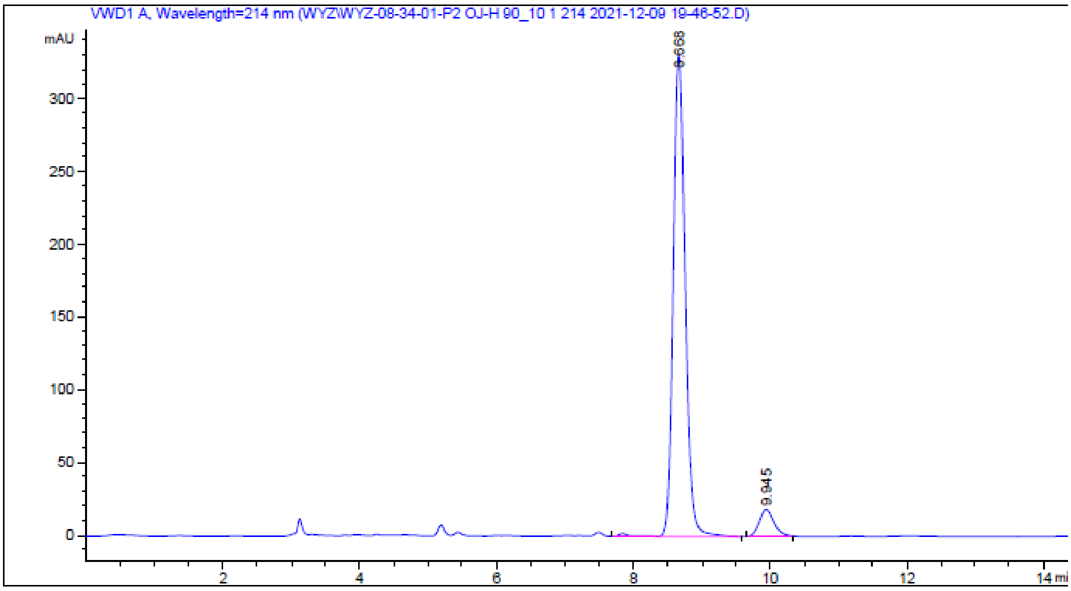

Peak NO	Ret. Time (min)	Area/%
1	8.668	93.4802
2	9.945	6.5198

(*R*)-9-(4-(1-phenylpropyl)phenyl)-9*H*-carbazole (3x)

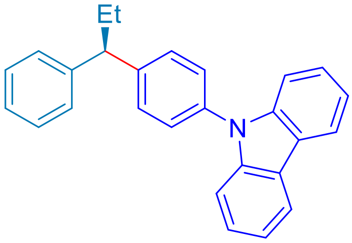

Prepared according to the general procedure **1** from (1-chloropropyl)benzene (0.4 mmol, 2 equiv.) and **2** from 9-(4-bromophenyl)-9*H*-carbazole (0.2 mmol, 1 equiv.). The title compound was isolated (gradient 0–5% EtOAc/hexanes) as a yellow oil (39.8 mg, 55% yield, 85% ee).

^{1}H NMR (400 MHz, $CDCl_3$) δ 8.19 (d, *J* = 7.6 Hz, 2H), 7.51 (s, 4H), 7.47 – 7.42 (m, 4H), 7.42 – 7.36 (m, 4H), 7.35 – 7.26 (m, 3H), 3.97 (t, *J* = 7.6 Hz, 1H), 2.22 (p, *J* = 7.2 Hz, 2H), 1.04 (t, *J* = 7.2 Hz, 3H).

^{13}C NMR (101 MHz, $CDCl_3$) δ 144.7, 144.6, 141.0, 135.5, 129.3, 128.6, 128.1, 127.0, 126.4, 125.9, 123.3, 120.3, 119.8, 109.9, 53.1, 28. 8, 12.9.

IR (neat): 2927, 1514, 1452, 1263, 1231, 1094, 1017, 801, 738, 626 cm^{-1}.

HRMS (EI) calcd for $C_{27}H_{23}N$ $[M]^+$:361.1832; found: 361.1825.

$[\alpha]_D$ ^{27} = -7.12 (*c* = 0.5, $CHCl_3$).

Enantiomeric excess = 85%, determined by HPLC (Daicel Chiralpak AD-H Column, *n*-Hexane:*i*-PrOH = 90:10, flow rate 1.0 mL/min, T = 25 °C, λ = 214 nm): t_R = 5.514

min (minor), t_R = 7.048 min (major).

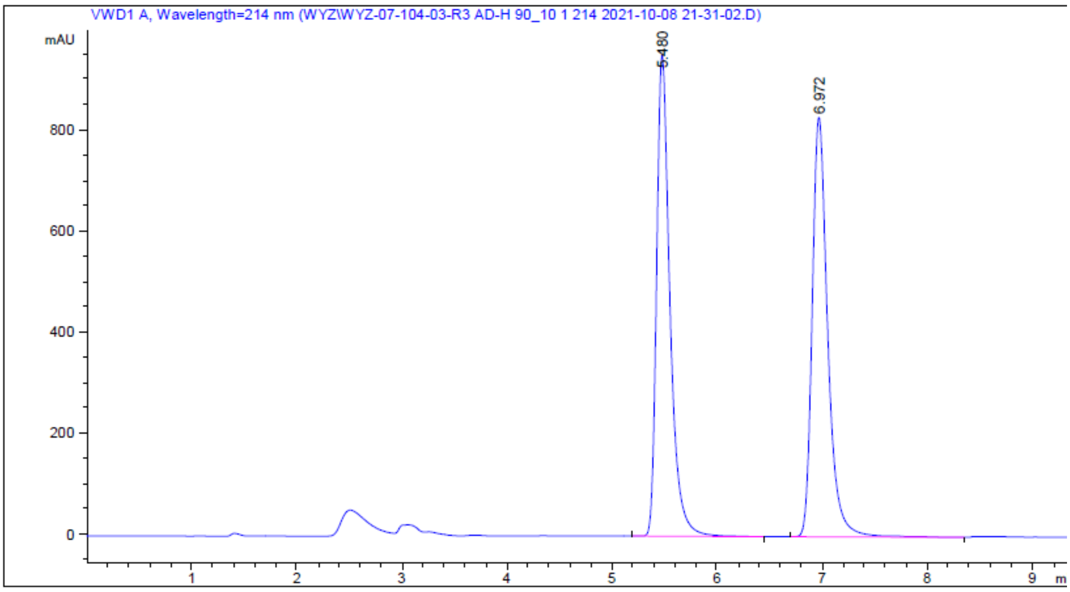

Peak NO	Ret. Time (min)	Area/%
1	5.480	49.8921
2	6.972	50.1079

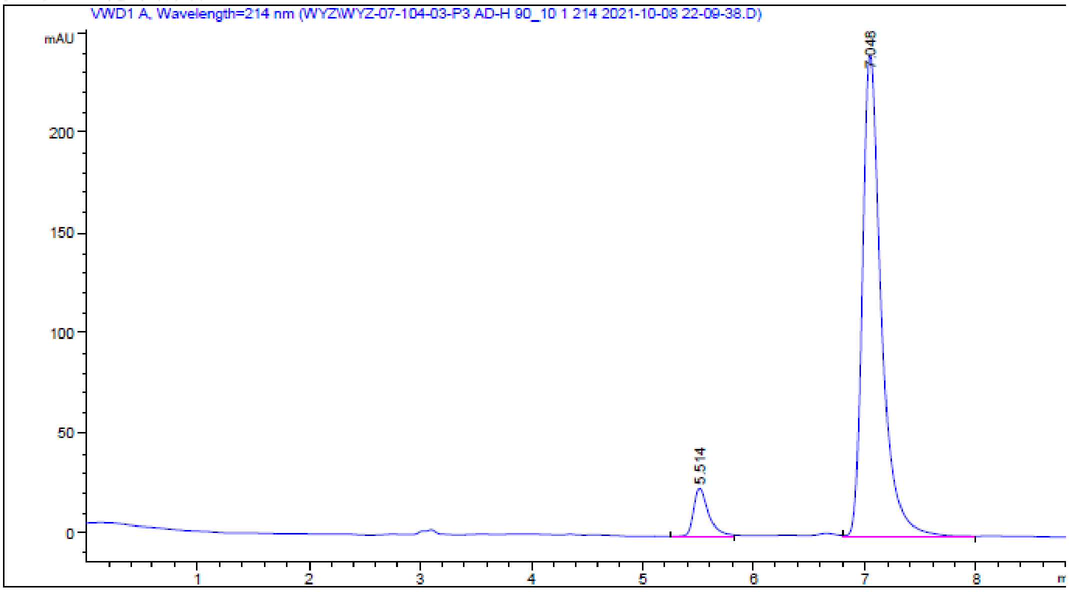

Peak NO	Ret. Time (min)	Area/%
1	5.514	7.3720
2	7.048	92.6280

***tert*-butyl (S)-4-(5-(1-phenylpropyl)pyridin-2-yl)piperazine-1-carboxylate (3y)**

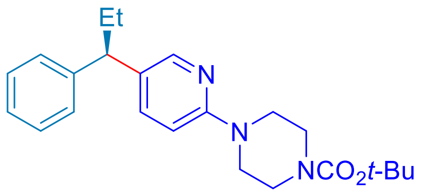

Prepared according to the general procedure **1** from (1-chloropropyl)benzene (0.4 mmol, 2 equiv.) and **2** from tert-butyl 4-(5-bromopyridin-2-yl)piperazine-1-carboxylate (0.2 mmol, 1 equiv.). The title compound was isolated (gradient 0–5% EtOAc/hexanes) as a yellow solid (41.9 mg, 55% yield, 82% ee).

M. P.: 163.1 – 163.8 °C

^{1}H NMR (400 MHz, $CDCl_3$) δ 8.13 (d, J = 2.4 Hz, 1H), 7.38 (d, J = 7.6 Hz, 1H), 7.31 – 7.27 (m, 2H), 7.26 – 7.13 (m, 3H), 6.62 (d, J = 8.8 Hz, 1H), 3.71 (t, J = 7.6 Hz, 1H), 3.68 – 3.30 (m, 8H), 2.18 – 1.93 (m, 2H), 1.50 (s, 9H), 0.92 (t, J = 7.2 Hz, 3H).

^{13}C NMR (101 MHz, $CDCl_3$) δ 154.8, 144.7, 130.2, 128.5, 127.7, 126.2, 107.5, 80.0, 49.8, 45.4 , 28.4, 12.7.

$[\alpha]_D^{24}$ = -3.49 (c = 0.3, $CHCl_3$).

Enantiomeric excess = 82%, determined by HPLC (Daicel Chiralpak AD-H Column, n-Hexane: i-PrOH = 90:10, flow rate 1.0 mL/min, T = 25 °C, λ = 214 nm): t_R = 7.592 min (minor), t_R = 10.486 min (major).

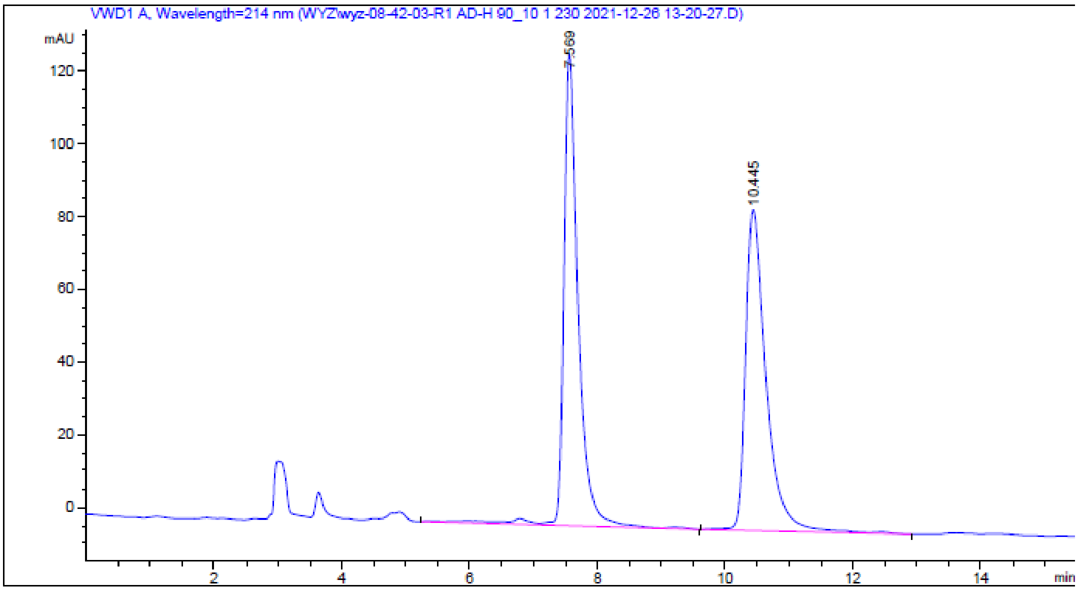

Peak NO	Ret. Time (min)	Area/%
1	7.569	51.1109
2	10.445	48.8891

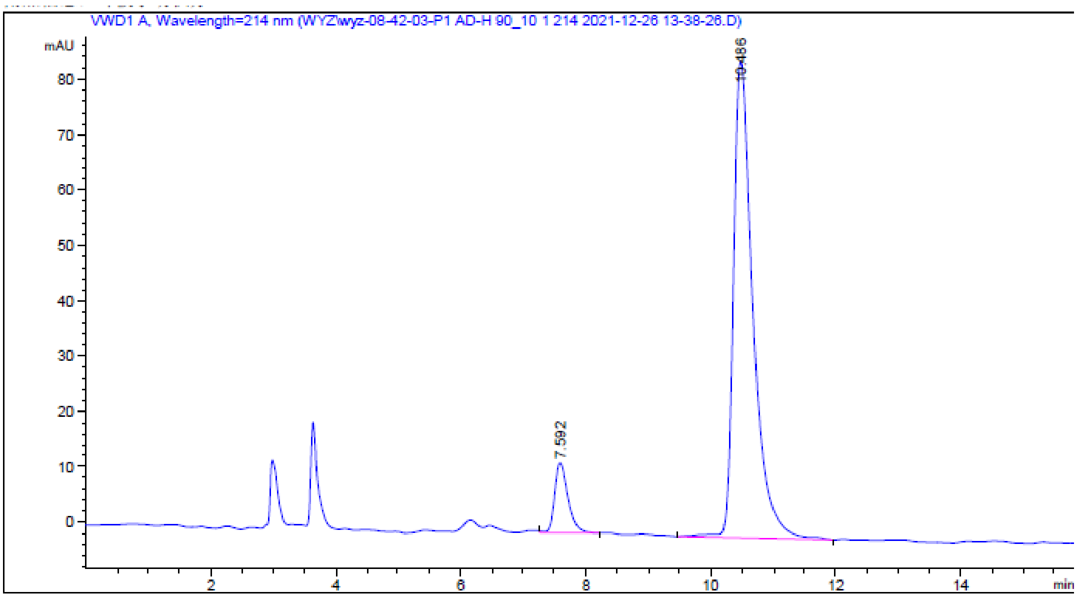

Peak NO	Ret. Time (min)	Area/%
1	7.592	9.0624
2	10.486	90.9376

(*R*)-*N*, *N*-dimethyl-5-(1-phenylpropyl)pyrimidin-2-amine (3z)[2]

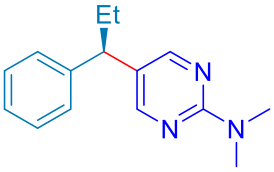

Prepared according to the general procedure **1** from (1-chloropropyl)benzene (0.4 mmol, 2 equiv.) and **2** from 5-bromo-N,N-dimethylpyrimidin-2-amine (0.2 mmol, 1 equiv.). The title compound was isolated (gradient 0–5% EtOAc/hexanes) as a yellow oil (19.5 mg, 40% yield, 61% ee).

^{1}H NMR (400 MHz, $CDCl_3$) δ 8.20 (s, 2H), 7.30 – 7.15 (m, 5H), 3.61 (t, *J* = 7.6 Hz, 1H), 3.16 (s, 6H), 2.01 (td, *J* = 14.0, 6.8 Hz, 2H), 0.90 (t, *J* = 7.2 Hz, 3H).

^{13}C NMR (101 MHz, $CDCl_3$) δ 156.9, 144.1, 141.6, 128.5, 127.6, 126.3, 124.7, 47.8, 37.2, 28.2, 12.6.

$[\alpha]_D$ ^{27} = -63.72 (*c* = 0.1, $CHCl_3$).

Enantiomeric excess = 61%, determined by HPLC (Daicel Chiralpak OJ-H Column, *n*-Hexane:*i*-PrOH = 90:10, flow rate 1.0 mL/min, T = 25 °C, λ = 214 nm): t_R = 8.953 min (minor), t_R = 12.377 min (major).

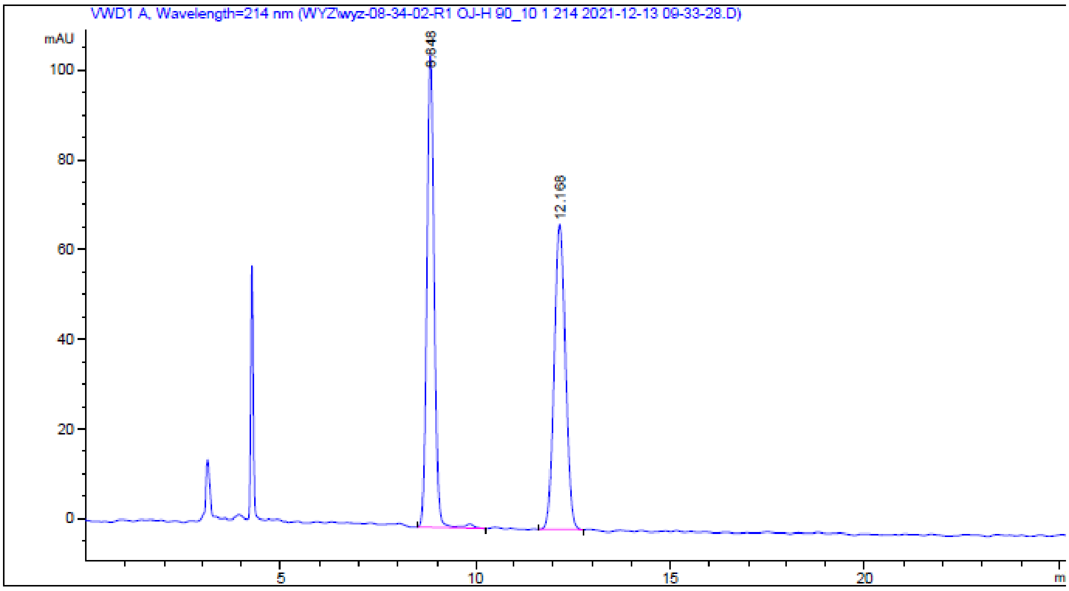

Peak NO	Ret. Time (min)	Area/%
1	8.848	50.5818
2	12.168	49.4182

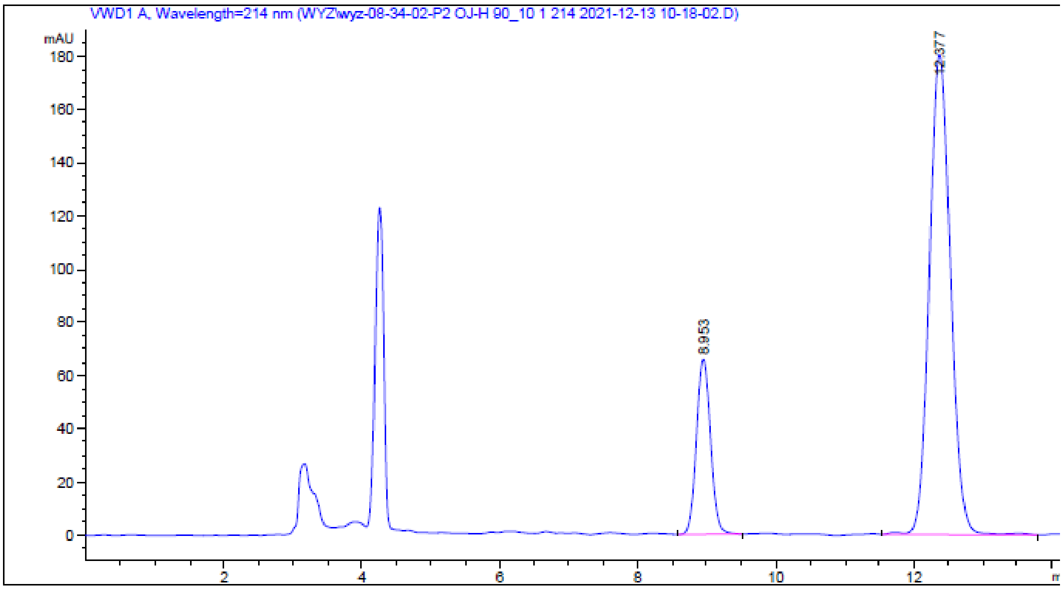

Peak NO	Ret. Time (min)	Area/%
1	8.953	19.5285
2	12.377	80.4715

methyl (*R*)-4-(1-(*p*-tolyl)propyl)benzoate (3aa)[2]

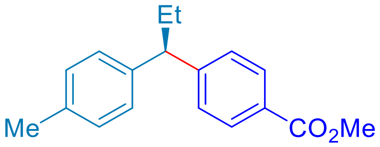

Prepared according to the general procedure **1** from 1-(1-chloropropyl)-4-methylbenzene (0.4 mmol, 2 equiv.) and **2** from methyl 4-bromobenzoate (0.2 mmol, 1 equiv.). The title compound was isolated (gradient 0–5% EtOAc/hexanes) as a colorless oil (33.3 mg, 62% yield, 84% ee)。

^{1}H NMR (400 MHz, $CDCl_3$) δ 7.94 (d, *J* = 7.2 Hz, 2H), 7.29 (d, *J* = 7.6 Hz, 2H), 7.10 (s, 4H), 3.88 (s, 3H), 3.81 (t, *J* = 7.6 Hz, 1H), 2.30 (s, 3H), 2.10 – 2.03 (m, 2H), 0.89 (t, *J* = 7.2 Hz, 3H).

^{13}C NMR (101 MHz, $CDCl_3$) δ 167.1, 150.8, 141.2, 135.8, 129.7, 129.2, 128.8, 127.9, 127.7, 52.8, 51.9, 28.4, 20.9, 12.7.

$[\alpha]_D$ ^{24} = -6.19 (*c* = 0.3, $CHCl_3$).

Enantiomeric excess = 84%, determined by HPLC (Daicel Chiralpak OJ-H Column, *n*-Hexane:*i*-PrOH = 95:5, flow rate 0.7 mL/min, T = 25 °C, λ = 214 nm): t_R = 10.448 min (minor), t_R = 11.146 min (major).

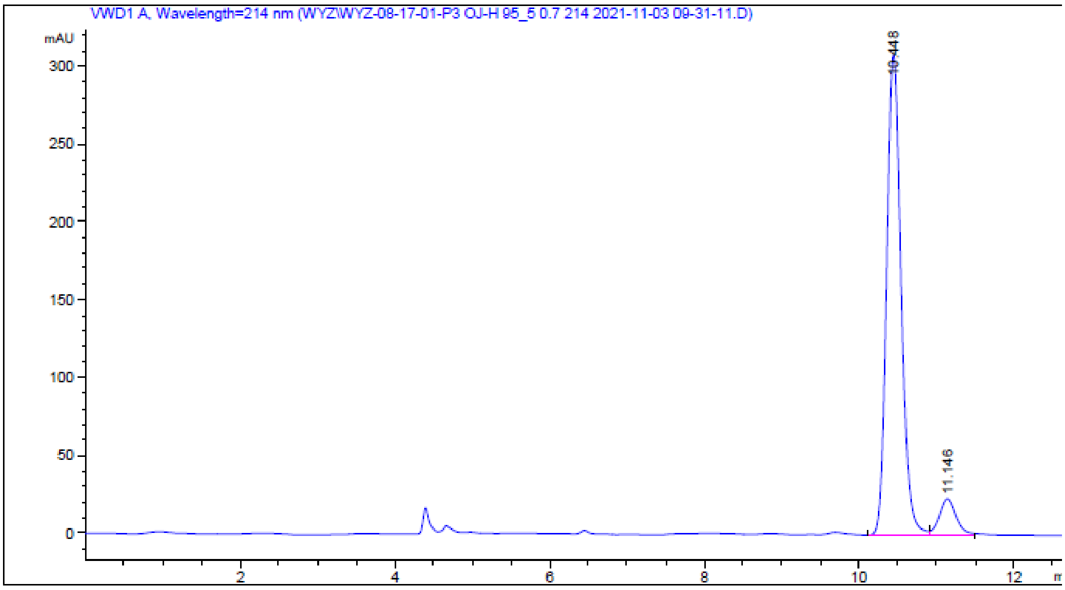

Peak NO	Ret. Time (min)	Area/%
1	10.448	91.8586
2	11.146	8.1414

methyl (*S*)-4-(1-(4-(trifluoromethyl)phenyl)propyl)benzoate (3ab)

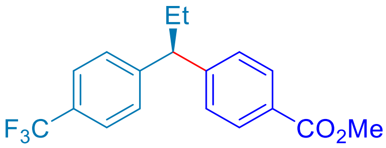

Prepared according to the general procedure **1** from (4-(1-chloropropyl)phenyl)(difluoro-l3-methyl)-l2-fluorane (0.4 mmol, 2 equiv.) and **2** from methyl 4-bromobenzoate (0.2 mmol, 1 equiv.). The title compound was isolated

(gradient 0–5% EtOAc/hexanes) as a colorless oil (54.8 mg, 85% yield, 80% ee).

^{1}H NMR (400 MHz, $CDCl_3$) δ 7.96 (d, *J* = 6.8 Hz, 2H), 7.53 (d, *J* = 7.6 Hz, 2H), 7.32 (d, *J* = 8.0 Hz, 2H), 7.28 (d, *J* = 7.2 Hz, 2H), 3.91 (d, *J* = 7.6 Hz, 1H), 3.88 (s, 3H), 2.10 (p, *J* = 7.2 Hz, 2H), 0.90 (t, *J* = 7.2 Hz, 3H).

^{13}C NMR (101 MHz, $CDCl_3$) δ 166.9, 149.4, 148.3, 130.0, 128.7 (q, *J* = 32.0 Hz), 128.5, 128.2, 127.9, 125.5 (q, *J* = 4.0 Hz), 122.9, 53.0, 52.1, 28.2, 12.5.

^{19}F NMR (377 MHz, $CDCl_3$) δ -62.45. **IR** (neat): 2924, 1724, 1610, 1326, 1280, 1165, 1068, 1018, 802, 706 cm^{-1}.

HRMS (EI) calcd for $C_{18}H_{17}F_3O_2$ $[M]^+$:322.1181; found: 322.1175.

$[\alpha]_D^{23}$ = -1.86 (*c* = 0.7, $CHCl_3$).

Enantiomeric excess = 80%, determined by HPLC (Daicel Chiralpak AD-H Column, *n*-Hexane:*i*-PrOH = 95:5, flow rate 0.7 mL/min, T = 25 °C, λ = 214 nm): t_R = 10.016 min (minor), t_R = 10.650 min (major).

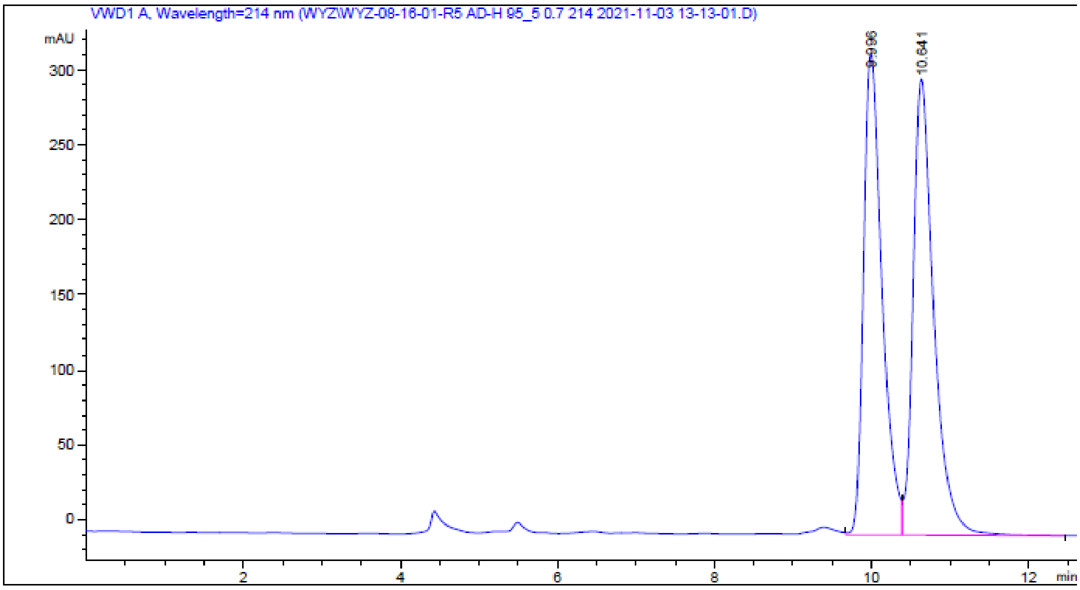

Peak NO	Ret. Time (min)	Area/%
1	9.996	48.5924
2	10.641	51.4076

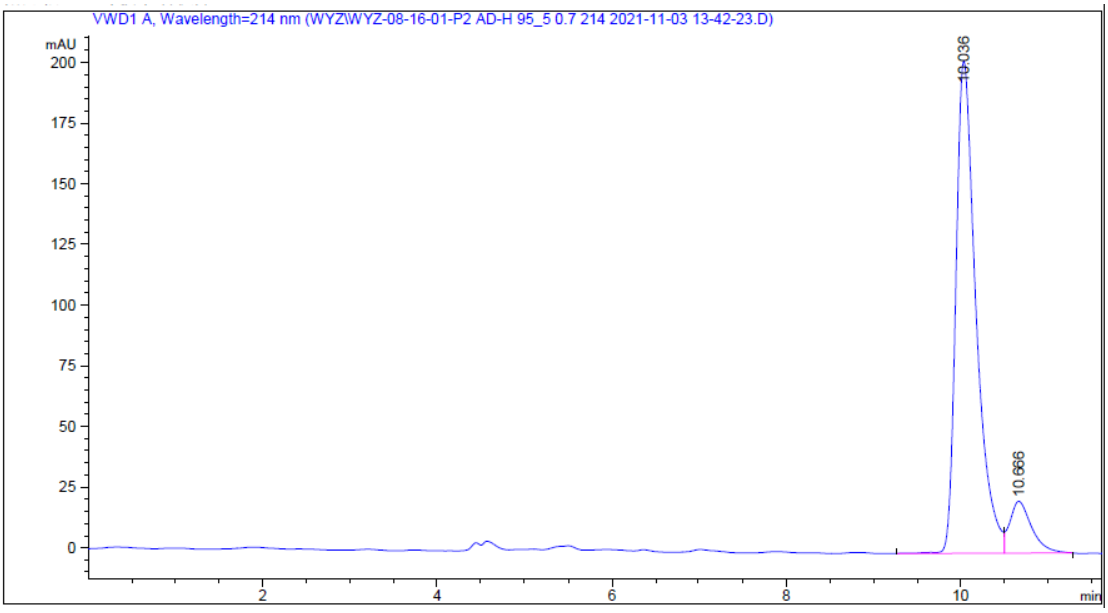

Peak NO	Ret. Time (min)	Area/%
1	10.036	89.7560
2	10.666	10.2440

methyl (*S*)-4-(1-(2-fluorophenyl)propyl)benzoate (3ac)

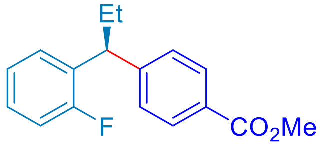

Prepared according to the general procedure **1** from 1-(1-chloropropyl)-2-fluorobenzene (0.4 mmol, 2 equiv.) and **2** from methyl 4-bromobenzoate (0.2 mmol, 1 equiv.). The title compound was isolated (gradient 0–5% EtOAc/hexanes) as a colorless oil (38.1 mg, 70% yield, 86% ee).

^{1}H NMR (400 MHz, $CDCl_3$) δ 7.97 (d, *J* = 8.0 Hz, 2H), 7.35 (d, *J* = 8.0 Hz, 2H), 7.30 – 7.24 (m, 1H), 7.23 – 7.16 (m, 1H), 7.15 – 7.06 (m, 1H), 7.06 – 6.95 (m, 1H), 4.23 (t, *J* = 7.6 Hz, 1H), 3.90 (s, 3H), 2.16 – 2.05 (m, 2H), 0.94 (t, *J* = 7.2 Hz, 3H).

^{13}C NMR (101 MHz, $CDCl_3$) δ 167.0, 160.8 (d, *J* = 244 Hz), 149.4, 131.1 (d, *J* = 14.0 Hz), 129.8 , 128.4 (d, *J* = 5.0 Hz) 128.2, 128.0, 127.9 (d, *J* = 8.0 Hz) 124.2 (d, *J* = 3.0 Hz), 115.5 (d, *J* = 23.0 Hz) , 52.0, 45.4, 27.4, 12.5.

^{19}F NMR (377 MHz, $CDCl_3$) δ -117.68.

IR (neat): 2926, 1722, 1610, 1489, 1280, 1112, 1019, 863, 756, 739 cm^{-1}.

HRMS (EI) calcd for $C_{17}H_{17}F_1O_2$ $[M]^+$:272.1214; found: 272.1207.

$[\alpha]_D$ ^{24} = -2.65 (*c* = 0.2, $CHCl_3$).

Enantiomeric excess = 86%, determined by HPLC (Daicel Chiralpak OJ-H Column, *n*-Hexane:*i*-PrOH = 90:10, flow rate 1.0 mL/min, T = 25 °C, λ = 214 nm): t_R = 6.051

min (minor), t_R = 6.536 min (major).

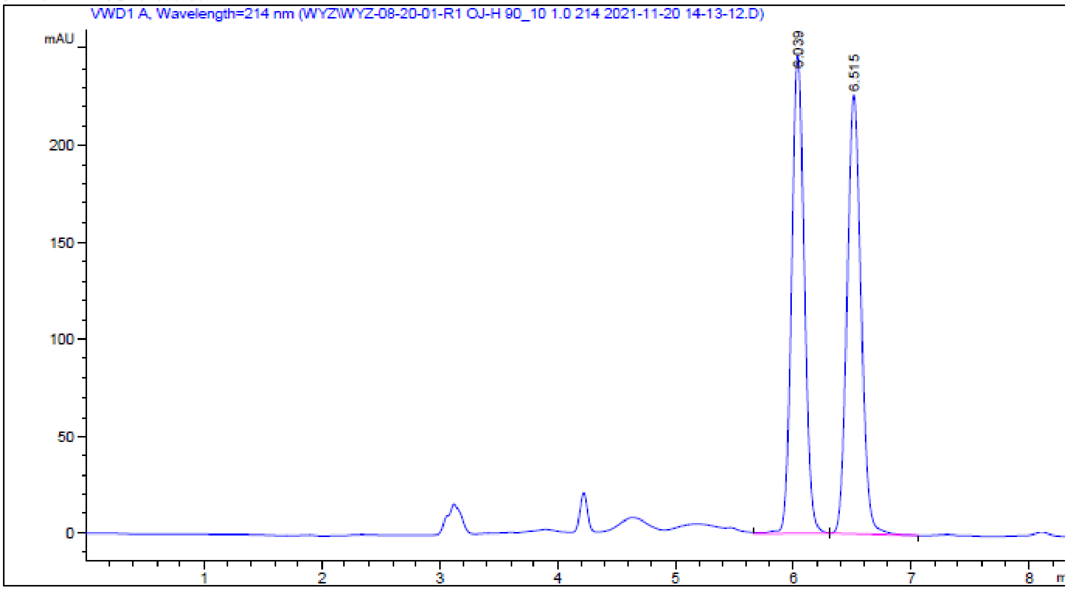

Peak NO	Ret. Time (min)	Area/%
1	6.039	49.7324
2	6.515	50.2676

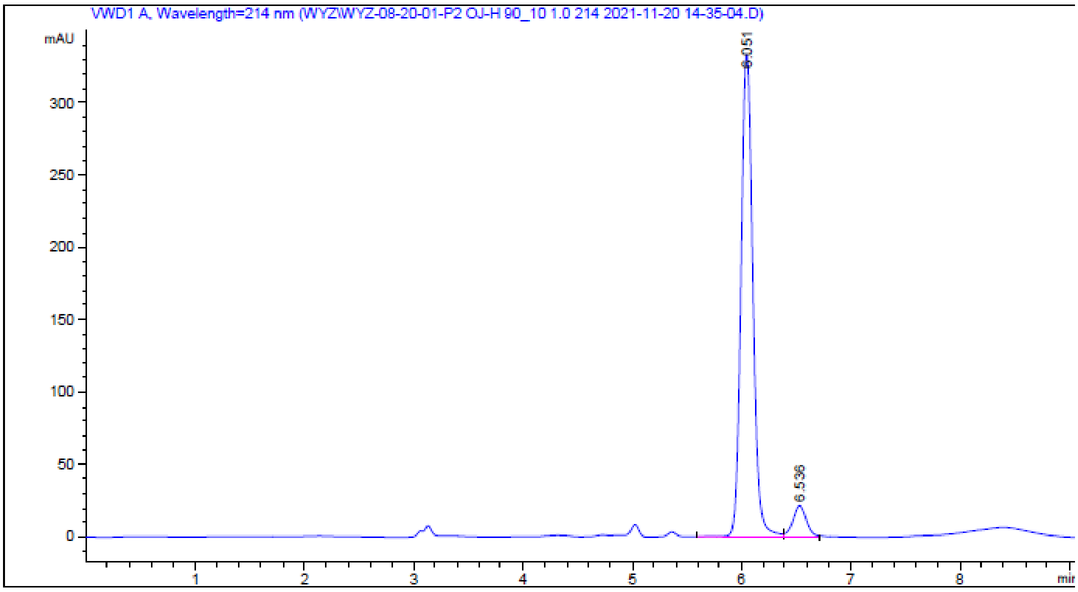

Peak NO	Ret. Time (min)	Area/%
1	6.051	92.9575
2	6.536	7.0425

methyl (*S*)-4-(1-(6-(trifluoromethyl)pyridin-3-yl)propyl)benzoate (3ad)

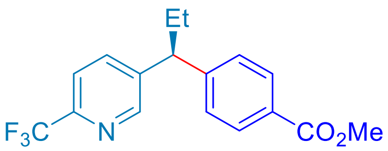

Prepared according to the general procedure **1** from 5-(1-chloropropyl)-2-(trifluoromethyl)pyridine (0.4 mmol, 2 equiv.) and **2** from methyl 4-bromobenzoate (0.2 mmol, 1 equiv.). The title compound was isolated (gradient 0–5% EtOAc/hexanes) as a colorless oil (32.3 mg, 50% yield, 73% ee).

^{1}H NMR (400 MHz, $CDCl_3$) δ 8.65 (s, 1H), 8.01 (d, *J* = 8.4 Hz, 2H), 7.69 (d, *J* = 8.0, 1H), 7.62 (d, *J* = 8.0 Hz, 1H), 7.31 (d, *J* = 8.4 Hz, 2H), 3.99 (t, *J* = 7.6 Hz, 1H), 3.92 (s, 3H), 2.28 – 2.06 (m, 2H), 0.95 (t, *J* = 7.2 Hz, 3H).

^{13}C NMR (101 MHz, $CDCl_3$) δ 166.7, 149.8, 147.9, 146.4 (q, *J* = 40.0 Hz), 143.0, 136.3, 130.2, 128.9, 127.9, 125.4, 120.4 (q, *J* = 10.0 Hz), 52.1, 50.5, 28.1, 12.4.

^{19}F NMR (377 MHz, $CDCl_3$) δ -67.82.

IR (neat): 2918, 1727, 1463, 1339, 1260, 1089, 1021, 801, 762 cm^{-1}.

HRMS (ESI) calcd for $C_{17}H_{17}NO_2F_3$ $[M+H]^+$:324.11998; found: 324.12059.

$[\alpha]_D$ ^{22} = -6.59 (*c* = 0.1, $CHCl_3$).

Enantiomeric excess = 73%, determined by HPLC (Daicel Chiralpak AD-H Column, *n*-Hexane:*i*-PrOH = 90:10, flow rate 1.0 mL/min, T = 25 °C, λ = 254 nm): t_R = 7.249 min (minor), t_R = 7.862 min (major).

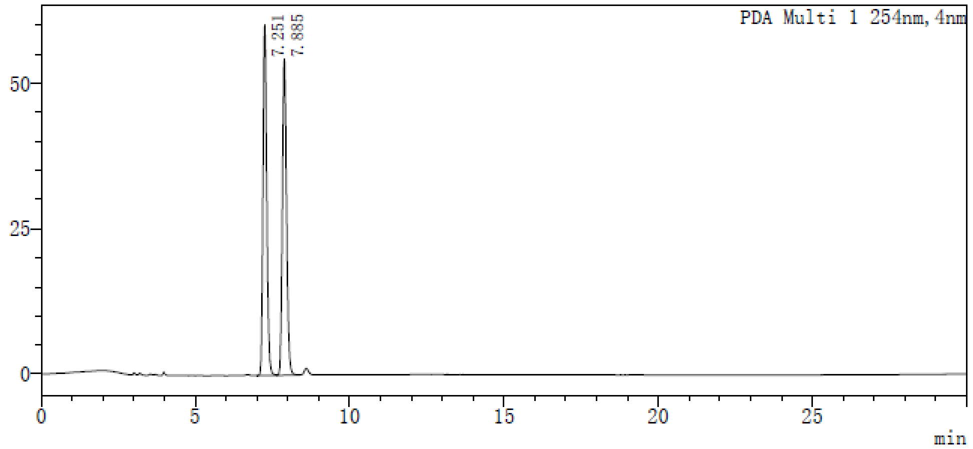

Peak NO	Ret. Time (min)	Area/%
1	7.251	50.050
2	7.885	49.950

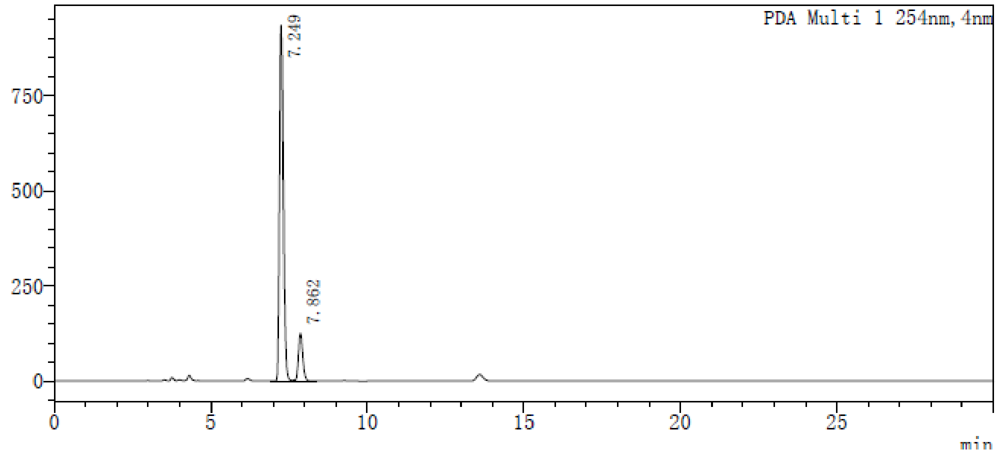

Peak NO	Ret. Time (min)	Area/%
1	7.249	86.299
2	7.862	13.701

methyl (*S*)-4-(1-(6-methoxypyridin-3-yl)propyl)benzoate (3ae)

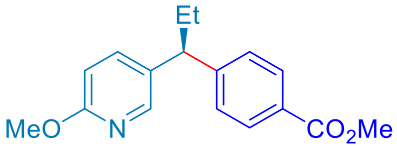

Prepared according to the general procedure **1** from (*R*)-5-(1-chloropropyl)-2-methoxypyridine (0.4 mmol, 2 equiv.) and **2** from methyl 4-bromobenzoate (0.2 mmol, 1 equiv.). The title compound was isolated (gradient 0–5% EtOAc/hexanes) as a yellow oil (40.3 mg, 71% yield, 79% ee).

^{1}H NMR (400 MHz, $CDCl_3$) δ 8.04 (d, *J* = 2.4 Hz, 1H), 7.94 (d, *J* = 8.4 Hz, 2H), 7.36 (dd, *J* = 8.4, 2.4 Hz, 1H), 7.26 (d, *J* = 8.4 Hz, 2H), 6.66 (d, *J* = 8.4 Hz, 1H), 3.89 (s, 3H), 3.87 (s, 3H), 3.78 (t, *J* = 7.6 Hz, 1H), 2.11 – 1.96 (m, 2H), 0.88 (t, *J* = 7.2 Hz, 3H).

^{13}C NMR (101 MHz, $CDCl_3$) δ 166.9, 162.8, 149.8, 145.6, 138.2, 132.2, 129.8, 128.3, 127.8, 110.8, 53.4, 52.0, 49.9, 28.1, 12.5.

$[\alpha]_D^{24}$ = -7.42 (*c* = 0.5, $CHCl_3$).

Enantiomeric excess = 79%, determined by HPLC (Daicel Chiralpak AD-H Column, *n*-Hexane:*i*-PrOH = 95:5, flow rate 1.0 mL/min, T = 25 °C, λ = 254 nm): t_R = 13.694 min (minor), t_R = 14.501 min (major).

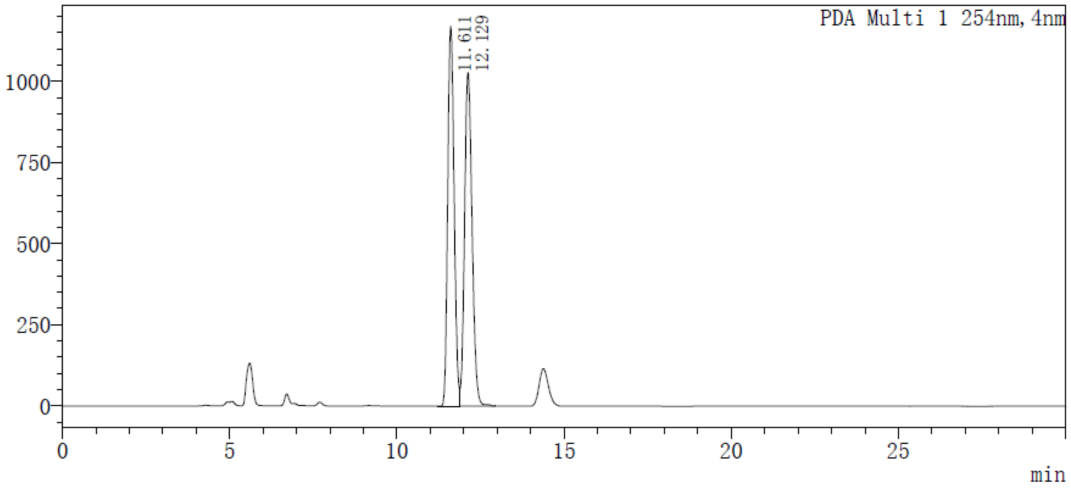

Peak NO	Ret. Time (min)	Area/%
1	11.611	49.262
2	12.129	50.738

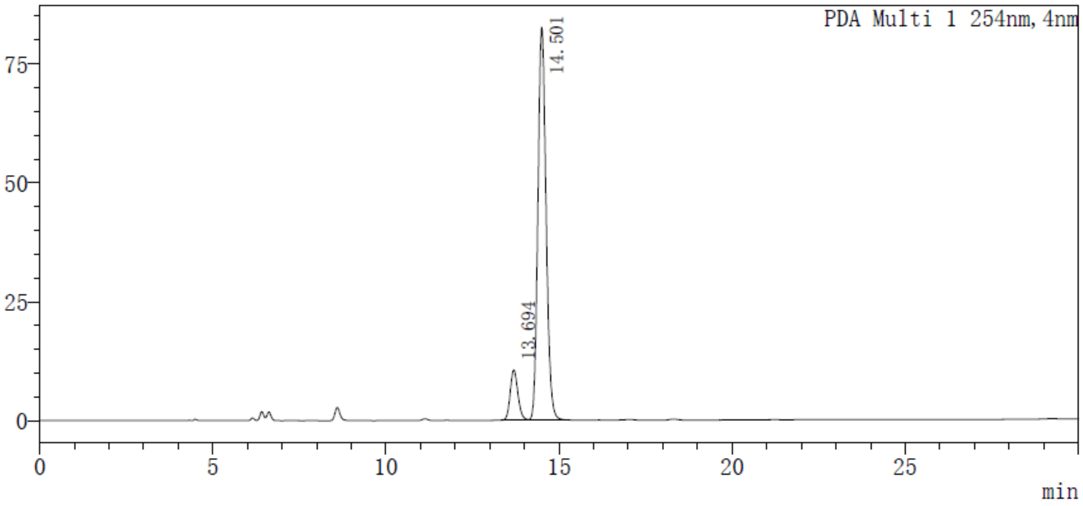

Peak NO	Ret. Time (min)	Area/%
1	13.694	10.733
2	14.501	89.267

methyl (*R*)-4-(1-phenylethyl)benzoate (3af)[7]

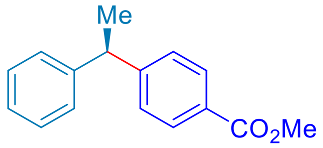

Prepared according to the general procedure **1** from (1-chloroethyl)benzene (0.4 mmol, 2 equiv.) and **2** from methyl 4-bromobenzoate (0.2 mmol, 1 equiv.). The title compound was isolated (gradient 0–5% EtOAc/hexanes) as a colorless oil (40.7 mg, 85% yield,

88% ee).

^{1}H NMR (400 MHz, CDCl$_3$) δ 7.96 (d, *J* = 8.0 Hz, 2H), 7.33 – 7.23 (m, 4H), 7.20 (d, *J* = 7.2 Hz, 3H), 4.20 (q, *J* = 7.2 Hz, 1H), 3.89 (s, 3H), 1.65 (d, *J* = 7.2 Hz, 3H).

^{13}C NMR (101 MHz, CDCl$_3$) δ 167.0, 151.7, 145.4, 129.7, 128.5, 128.0, 127.6, 127.6, 126.3, 51.9, 44.8, 21.6.

$[\alpha]_D^{21}$ = -6.01 (*c* = 1.6, CHCl$_3$).

Enantiomeric excess = 88%, determined by HPLC (Daicel Chiralpak OB-H Column, *n*-Hexane:*i*-PrOH = 90:10, flow rate 0.7 mL/min, T = 25 °C, λ = 214 nm): t_R = 12.447 min (minor), t_R = 15.650 min (major).

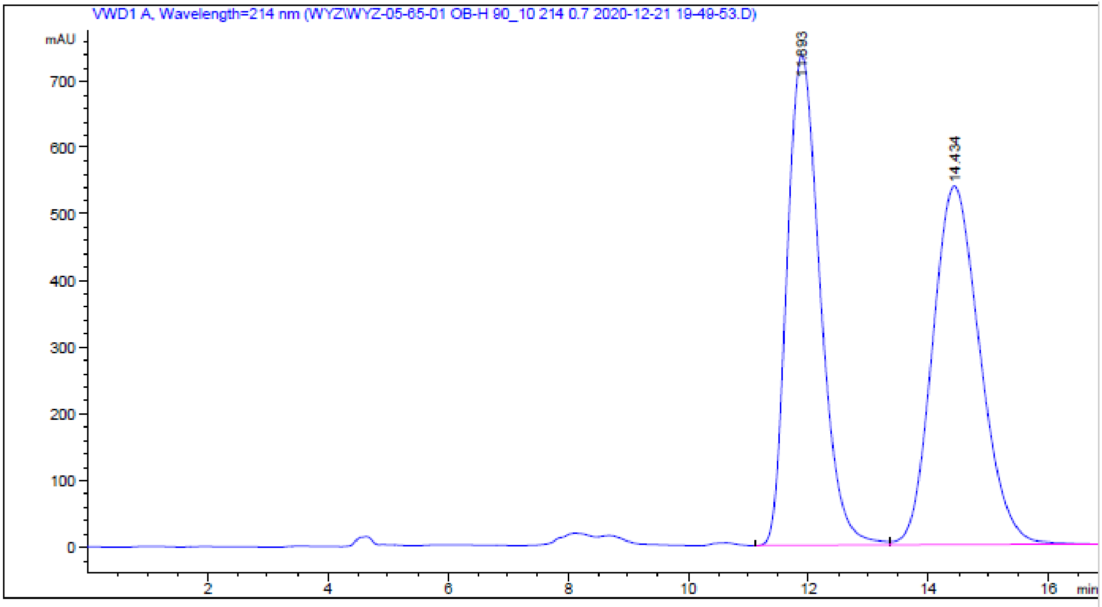

Peak NO	Ret. Time (min)	Area/%
1	11.893	48.9800
2	14.434	51.0200

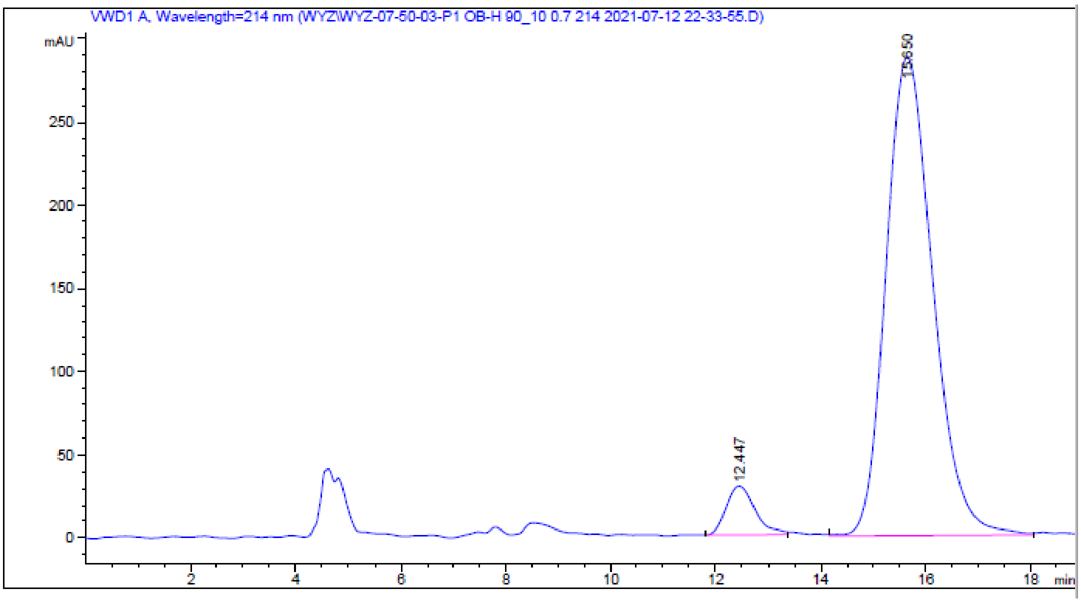

Peak NO	Ret. Time (min)	Area/%
1	12.447	5.9128
2	15.650	94.0872

methyl (*S*)-4-(1-(naphthalen-2-yl)ethyl)benzoate (3ag)[7]

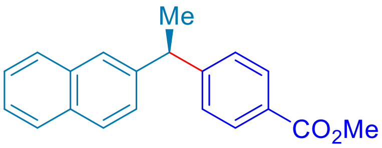

Prepared according to the general procedure **1** from 2-(1-chloroethyl)naphthalene (0.4 mmol, 2 equiv.) and **2** from methyl 4-bromobenzoate (0.2 mmol, 1 equiv.). The title compound was isolated (gradient 0–5% EtOAc/hexanes) as a yellow oil (47.5 mg, 82% yield, 63% ee).

^{1}H NMR (400 MHz, $CDCl_3$) δ 7.93 (d, *J* = 8.4 Hz, 2H), 7.76 (d, *J* = 8.0 Hz, 2H), 7.71 (d, *J* = 8.4 Hz, 1H), 7.65 (s, 1H), 7.46 – 7.37 (m, 2H), 7.29 (d, *J* = 8.0 Hz, 2H), 7.25 – 7.19 (m, 1H), 4.32 (q, *J* = 7.2 Hz, 1H), 3.85 (s, 3H), 1.71 (d, *J* = 7.2 Hz, 3H).

^{13}C NMR (101 MHz, $CDCl_3$) δ 167.0, 151.5, 142.8, 133.4, 132.1, 129.7, 128.1, 128.0, 127.8, 127.7, 127.6, 126.6, 126.1, 125.5,125.4, 52.0, 44.8, 21.5.

$[\alpha]_D^{25}$ = -21.27 (*c* = 0.6, $CHCl_3$).

Enantiomeric excess = 63%, determined by HPLC (Daicel Chiralpak OD-H Column, *n*-Hexane:*i*-PrOH = 90:10, flow rate 1.0 mL/min, T = 25 °C, λ = 214 nm): t_R = 6.486 min (minor), t_R = 7.040 min (major).

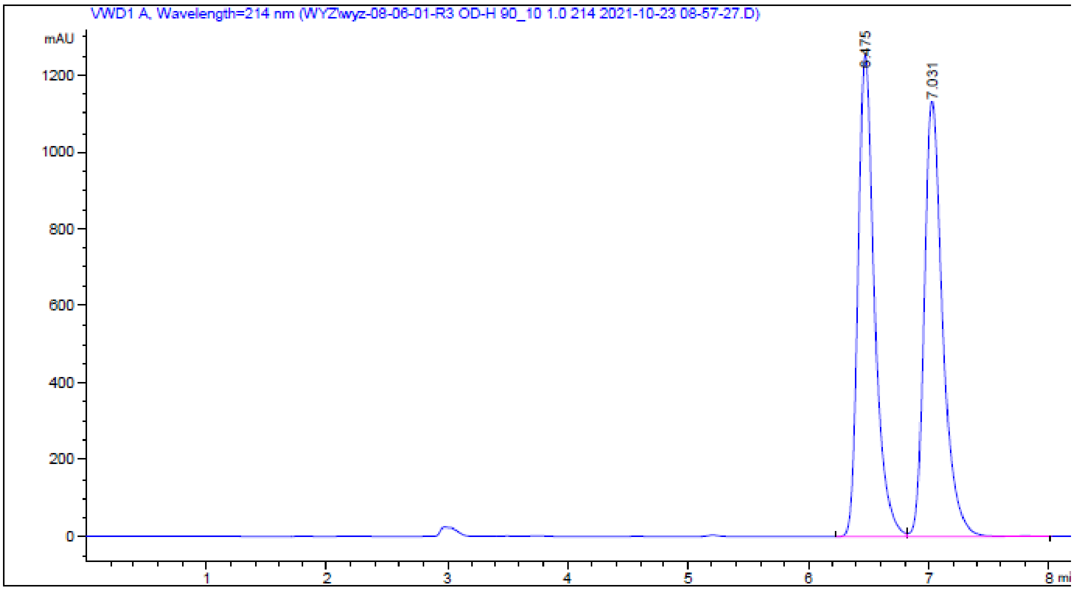

Peak NO	Ret. Time (min)	Area/%
1	6.475	49.7498
2	7.031	50.2502

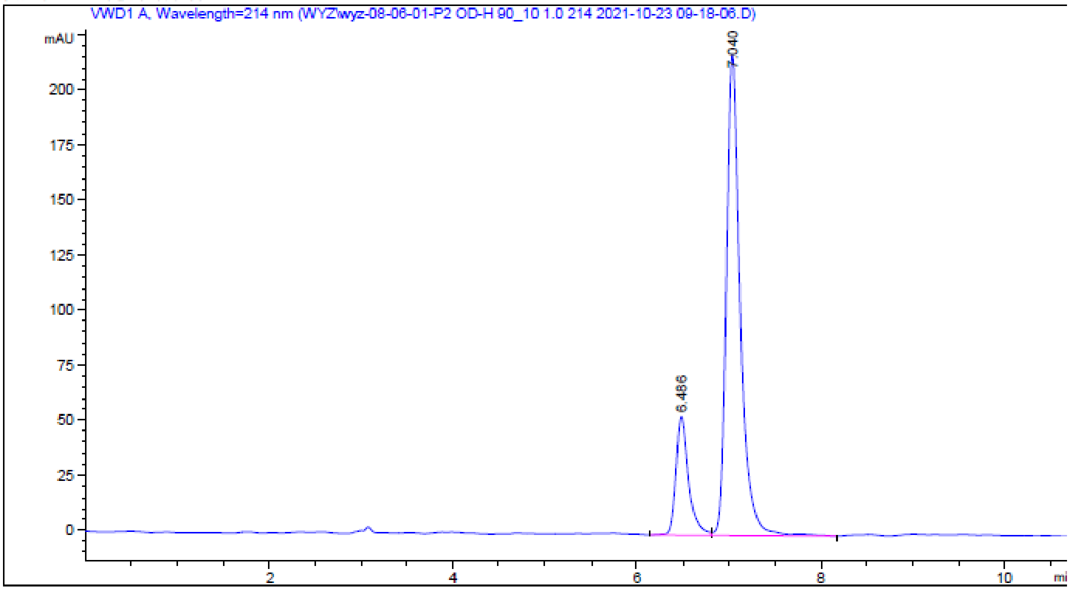

Peak NO	Ret. Time (min)	Area/%
1	6.486	18.3086
2	7.040	81.6914

methyl (*R*)-4-(1-phenylpentyl)benzoate (3ah)[4]

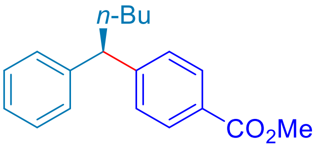

Prepared according to the general procedure **1** from (1-chloropentyl)benzene (0.4 mmol, 2 equiv.) and **2** from methyl 4-bromobenzoate (0.2 mmol, 1 equiv.). The title compound was isolated (gradient 0–5% EtOAc/hexanes) as a colorless oil (33.2 mg, 59% yield, 89% ee).

^{1}H NMR (400 MHz, $CDCl_3$) δ 7.95 (d, *J* = 7.6 Hz, 2H), 7.35 – 7.24 (m, 4H), 7.24 – 7.12 (m, 3H), 3.94 (t, *J* = 7.6 Hz, 1H), 3.88 (s, 3H), 2.08 – 2.03 (m, 2H), 1.41 – 1.29 (m, 2H), 1.28 – 1.18 (m, 2H), 0.86 (t, *J* = 7.2 Hz, 3H).

^{13}C NMR (101 MHz, $CDCl_3$) δ 167.0, 150.7, 144.4, 129.7, 128.5, 128.0, 127.9, 127.8, 126.3, 52.0, 51.4, 35.2, 30.1, 22.6, 14.0.

$[\alpha]_D^{23}$ = -3.01 (*c* = 0.1, $CHCl_3$).

Enantiomeric excess = 89%, determined by HPLC (Daicel Chiralpak OJ-H Column, *n*-Hexane:*i*-PrOH = 95:5, flow rate 0.7 mL/min, T = 25 °C, λ = 214 nm): t_R = 9.210 min (minor), t_R = 9.854 min (major).

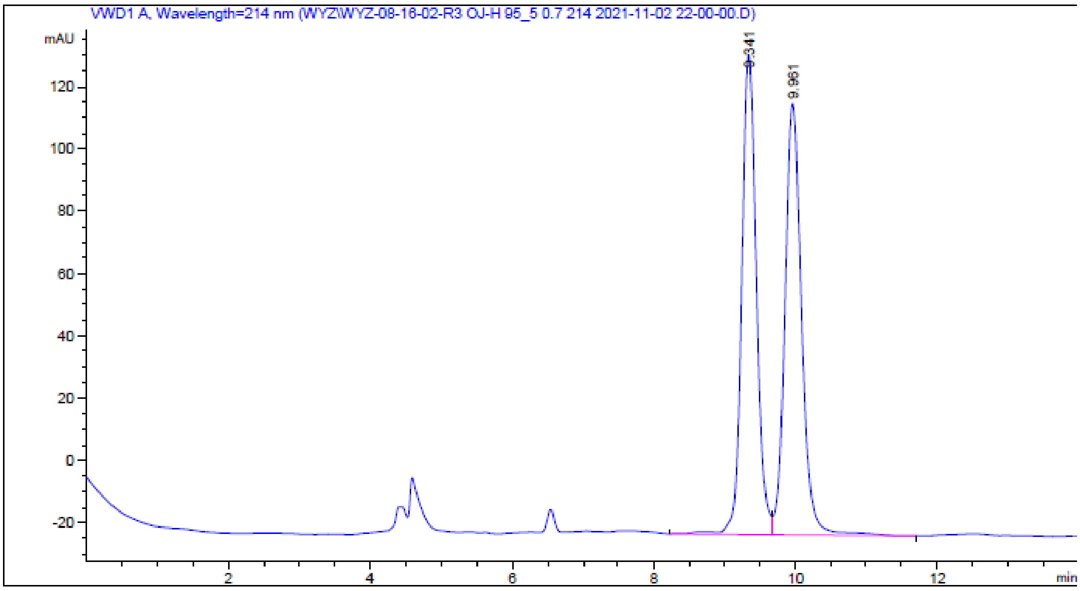

Peak NO	Ret. Time (min)	Area/%
1	9.341	49.4499
2	9.961	50.5501

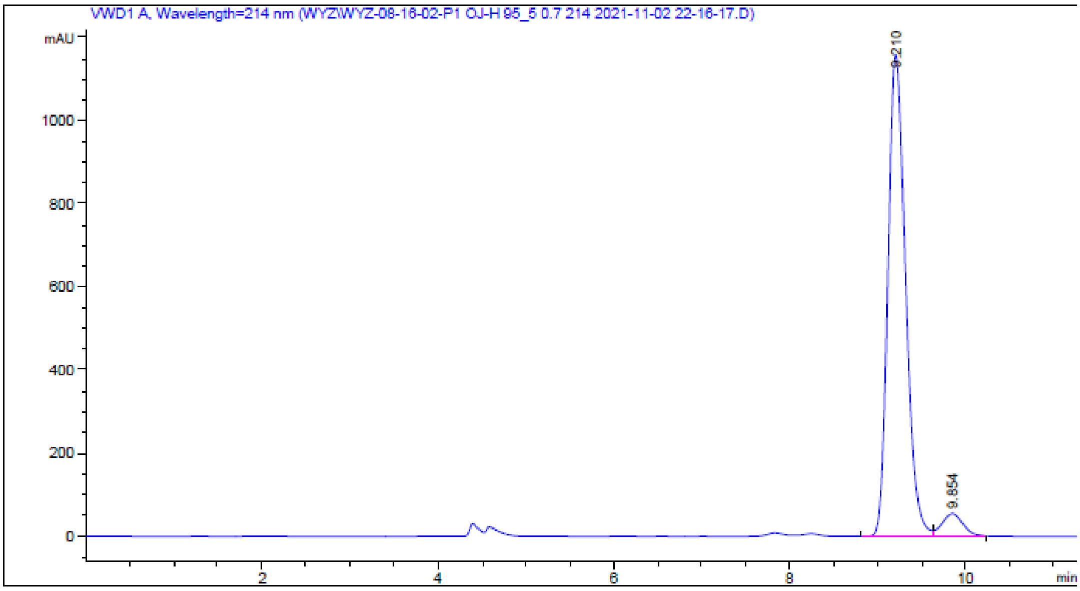

Peak NO	Ret. Time (min)	Area/%
1	9.210	94.5702
2	9.854	5.4298

methyl (*R*)-4-(3-chloro-1-phenylpropyl)benzoate (3ai)

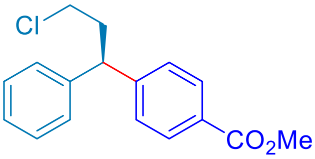

Prepared according to the general procedure **1** from (1,2-dichloroethyl)benzene (0.4 mmol, 2 equiv.) and **2** from methyl 4-bromobenzoate (0.2 mmol, 1 equiv.). The title compound was isolated (gradient 0–5% EtOAc/hexanes) as a colorless oil (47.2 mg, 70% yield, 91% ee).

^{1}H NMR (400 MHz, $CDCl_3$) δ 8.00 (d, *J* = 8.4 Hz, 2H), 7.38 – 7.31 (m, 4H), 7.30 – 7.23 (m, 3H), 4.33 (t, *J* = 7.6 Hz, 1H), 3.92 (s, 3H), 3.48 (t, *J* = 6.4 Hz, 2H), 2.54 (td, *J* = 7.6, 1.2 Hz, 2H).

^{13}C NMR (101 MHz, $CDCl_3$) δ 166.9, 148.9, 142.6, 130.0, 128.8, 128.5, 127.9, 127.9, 126.9, 52.1, 47.8, 42.9, 37.8.

IR (neat): 2922, 1720, 1436, 1279, 1260, 1103, 1018, 797, 704, 664 cm^{-1}.

HRMS (EI) calcd for $C_{17}H_{17}ClO_2$ $[M]^+$:288.0913; found: 288.0912.

$[\alpha]_D$ ^{25} = +0.14 (*c* = 0.2, $CHCl_3$).

Enantiomeric excess = 91%, determined by HPLC (Daicel Chiralpak OJ-H Column, *n*-Hexane:*i*-PrOH = 90:10, flow rate 1.0 mL/min, T = 25 °C, λ = 214 nm): t_R = 11.389 min (minor), t_R = 12.797 min (major).

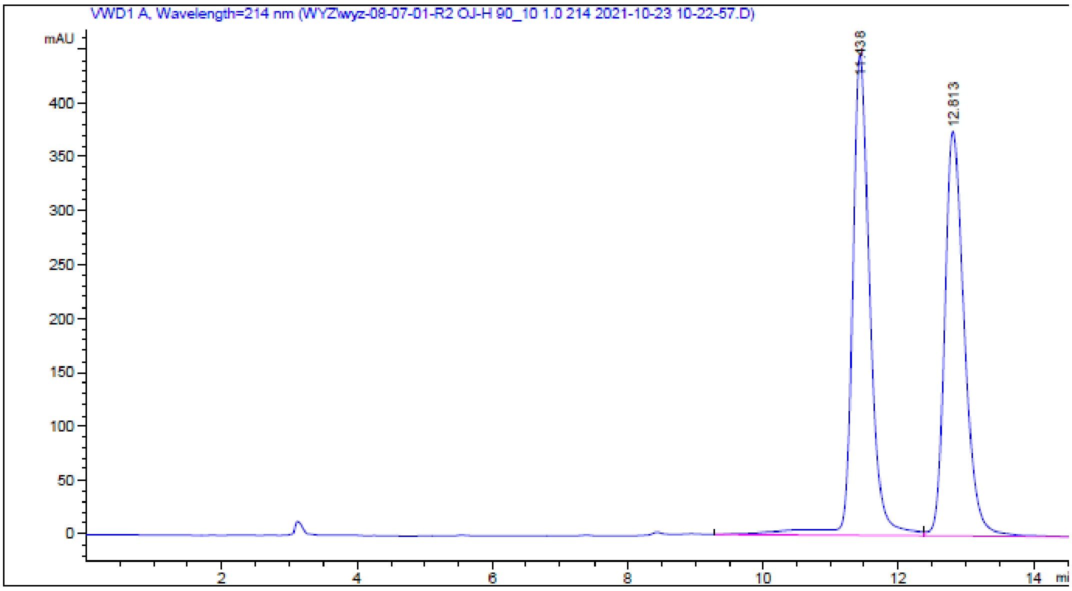

Peak NO	Ret. Time (min)	Area/%
1	11.438	51.6410
2	12.813	48.3590

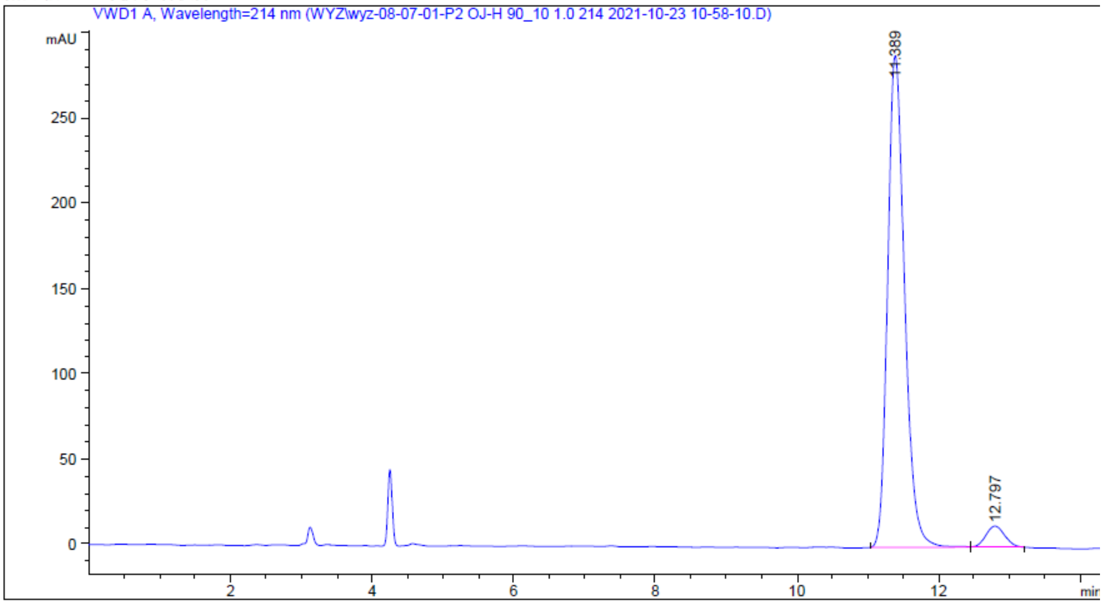

Peak NO	Ret. Time (min)	Area/%
1	11.389	95.3985
2	12.794	4.6015

methyl (*R*)-4-(1-phenylhex-5-en-1-yl)benzoate (3aj)

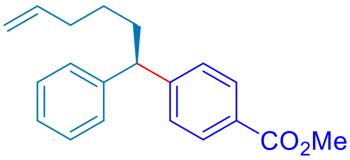

Prepared according to the general procedure **1** from (1-chlorohex-5-en-1-yl)benzene (0.4 mmol, 2 equiv.) and **2** from methyl 4-bromobenzoate (0.2 mmol, 1 equiv.). The title compound was isolated (gradient 0–5% EtOAc/hexanes) as a colorless oil (50.0 mg, 85% yield, 82% ee).

^{1}H NMR (400 MHz, CDCl$_3$) δ 7.90 (d, *J* = 8.0 Hz, 2H), 7.29 – 7.11 (m, 7H), 5.71 (dq, *J* =10.0, 6.4 Hz, 1H), 4.91 (dd, *J* = 17.2, 14.0 Hz, 2H), 3.90 (t, *J* = 7.6 Hz, 1H), 3.83 (s, 3H), 2.02 (dd, *J* = 14.6, 7.6 Hz, 4H), 1.36 – 1.26 (m, 2H).

^{13}C NMR (101 MHz, CDCl$_3$) δ 167.0, 150.5, 144.2, 138.4, 129.8, 128.5, 127.9, 127.8, 126.4, 114.7, 52.0, 51.2, 34.8, 33.6, 27.2.

IR (neat): 2957, 1722, 1609, 1436, 1279, 1181, 1111, 1019, 807, 770 cm^{-1}.

HRMS (EI) calcd for $C_{18}H_{20}O_2$ $[M]^+$:268.1459; found: 268.1458.

$[\alpha]_D$ ^{24} = -3.72 (*c* = 0.2, CHCl$_3$).

Enantiomeric excess = 82%, determined by HPLC (Daicel Chiralpak OD-H Column, *n*-Hexane:*i*-PrOH = 90:10, flow rate 1.0 mL/min, T = 25 °C, λ = 214 nm): t_R = 4.494 min (minor), t_R = 4.785 min (major).

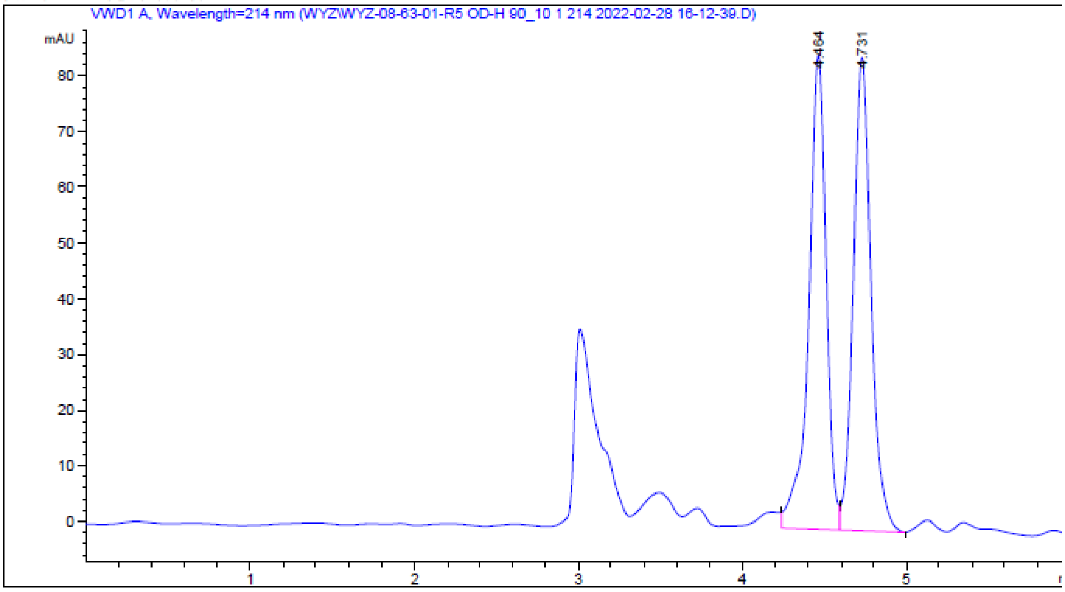

Peak NO	Ret. Time (min)	Area/%
1	4.464	49.7062
2	4.731	50.2938

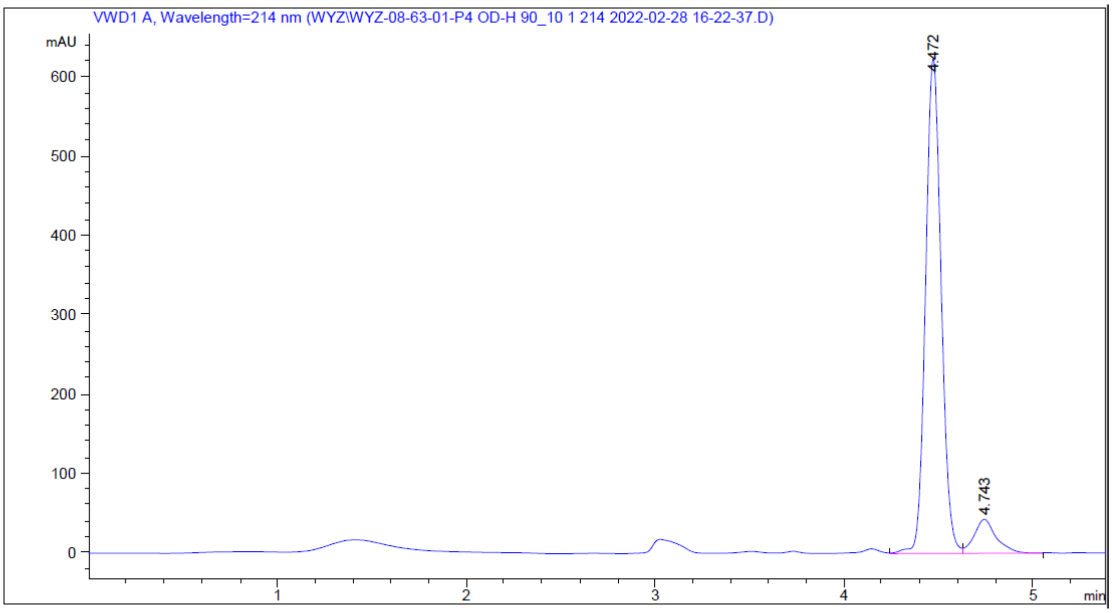

Peak NO	Ret. Time (min)	Area/%
1	4.472	91.1342
2	4.743	8.8658

methyl (*R*)-4-(phenyl(trimethylsilyl)methyl)benzoate (3ak) [8]

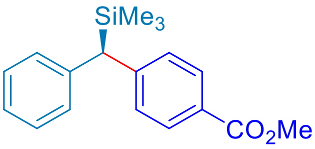

Prepared according to the general procedure **1** from (S)-(chloro(phenyl)methyl)trimethylsilane (0.4 mmol, 2 equiv.) and **2** from methyl 4-bromobenzoate (0.2 mmol, 1 equiv.). The title compound was isolated (gradient 0–5% EtOAc/hexanes) as a colorless oil (34.6 mg, 58% yield, 92% ee).

^{1}H NMR (400 MHz, $CDCl_3$) δ 7.92 (d, *J* = 8.4 Hz, 2H), 7.26 (ddd, *J* = 12.8, 8.0, 6.4 Hz, 6H), 7.19 – 7.13 (m, 1H), 3.87 (s, 3H), 3.59 (s, 1H), 0.03 (s, 9H).

^{13}C NMR (101 MHz, $CDCl_3$) δ 167.1, 148.7, 141.8, 129.6, 128.9, 128.4, 128.3, 126.9, 125.4, 51.9, 46.6, -1.8.

$[\alpha]_D^{24}$ = -9.34 (*c* = 0.5, $CHCl_3$).

Enantiomeric excess = 92%, determined by HPLC (Daicel Chiralpak AD-H Column, *n*-Hexane:*i*-PrOH = 95:5, flow rate 0.7 mL/min, T = 25 °C, λ = 214 nm): t_R = 7.511 min (minor), t_R = 8.089 min (major).

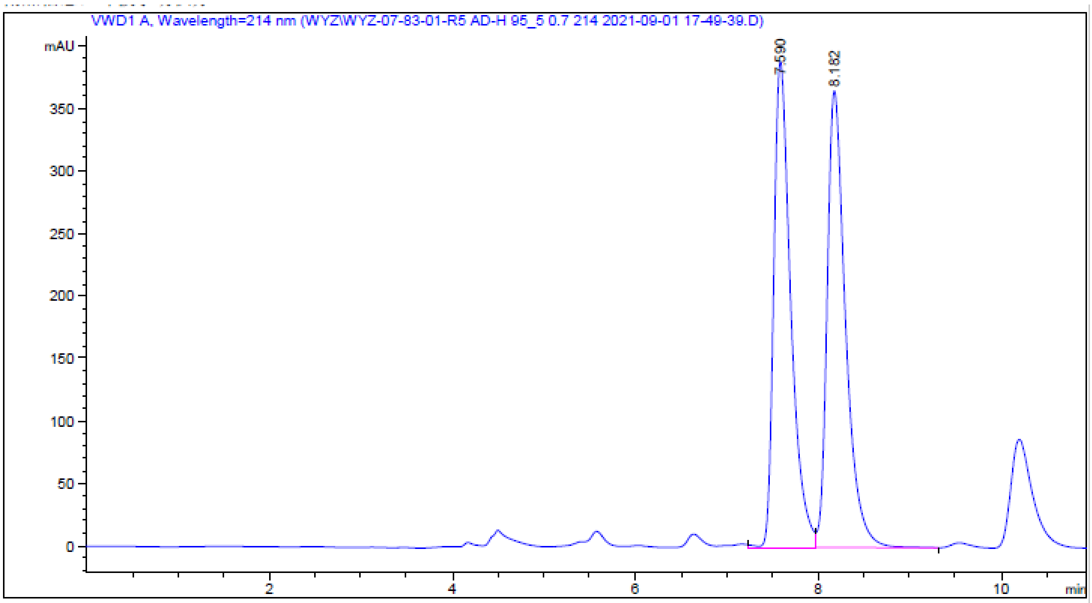

Peak NO	Ret. Time (min)	Area/%
1	7.590	49.5761
2	8.182	50.4239

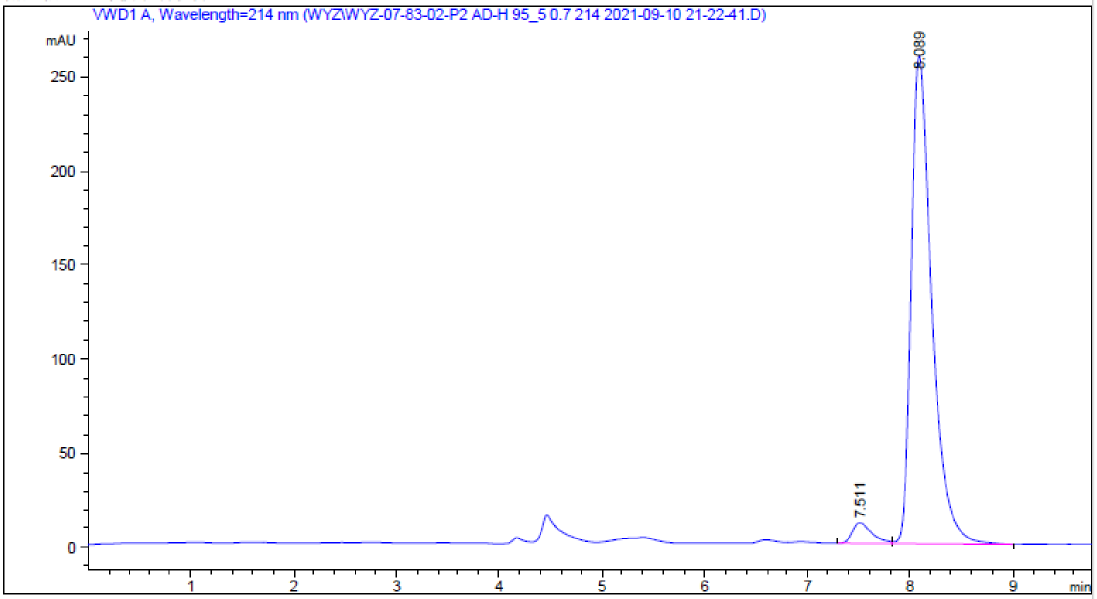

Peak NO	Ret. Time (min)	Area/%
1	7.511	3.8111
2	8.089	96.1889

methyl (*R*)-4-((dimethyl(phenyl)silyl)(phenyl)methyl)benzoate (3al)[8]

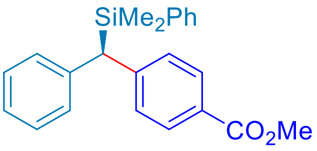

Prepared according to the general procedure **1** from (*S*)-(chloro(phenyl)methyl)dimethyl(phenyl)silane (0.4 mmol, 2 equiv.) and **2** from methyl 4-bromobenzoate (0.2 mmol, 1 equiv.). The title compound was isolated (gradient 0–5% EtOAc/hexanes) as a colorless oil (40.3 mg, 56% yield, 93% ee).

^{1}H NMR (400 MHz, $CDCl_3$) δ 7.86 (d, *J* = 8.0 Hz, 2H), 7.37 – 7.33 (m, 1H), 7.32 – 7.20 (m, 7H), 7.15 (dd, *J* = 7.2, 3.6 Hz, 4H), 3.88 (s, 3H), 3.81 (s, 1H), 0.29 (s, 6H).

^{13}C NMR (101 MHz, $CDCl_3$) δ 167.2, 148.1, 141.3, 136.9, 134.4, 129.5, 129.3, 129.1, 128.6, 128.4, 127.7, 125.6, 110.0, 51.9, 46.4, -3.1, -3.4.

$[\alpha]_D^{25}$ = -6.32 (*c* = 0.3, $CHCl_3$).

Enantiomeric excess = 93%, determined by HPLC (Daicel Chiralpak AD-H Column, *n*-Hexane:*i*-PrOH = 95:5, flow rate 0.7 mL/min, T = 25 °C, λ = 254 nm): t_R = 5.228 min (minor), t_R = 6.414 min (major).

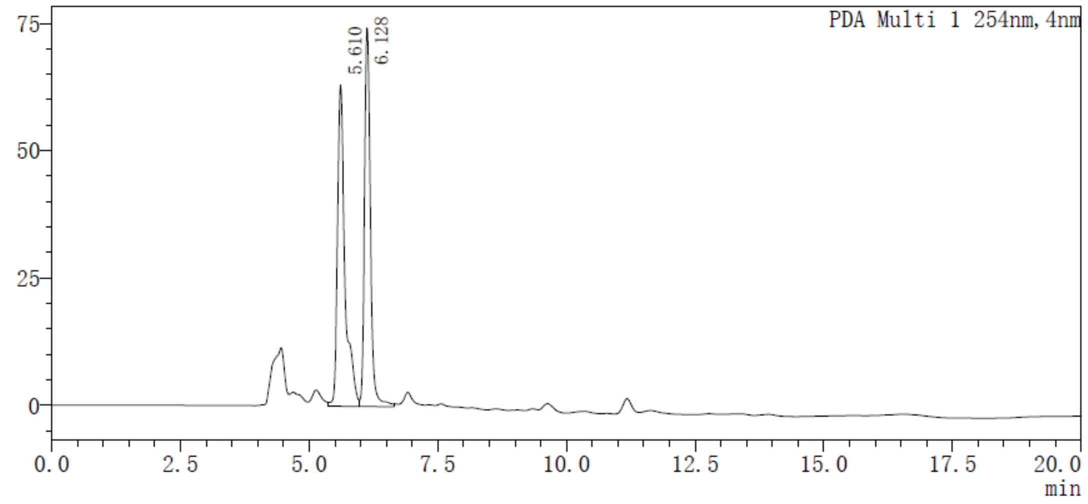

Peak NO	Ret. Time (min)	Area/%
1	5.610	51.920
2	6.128	48.080

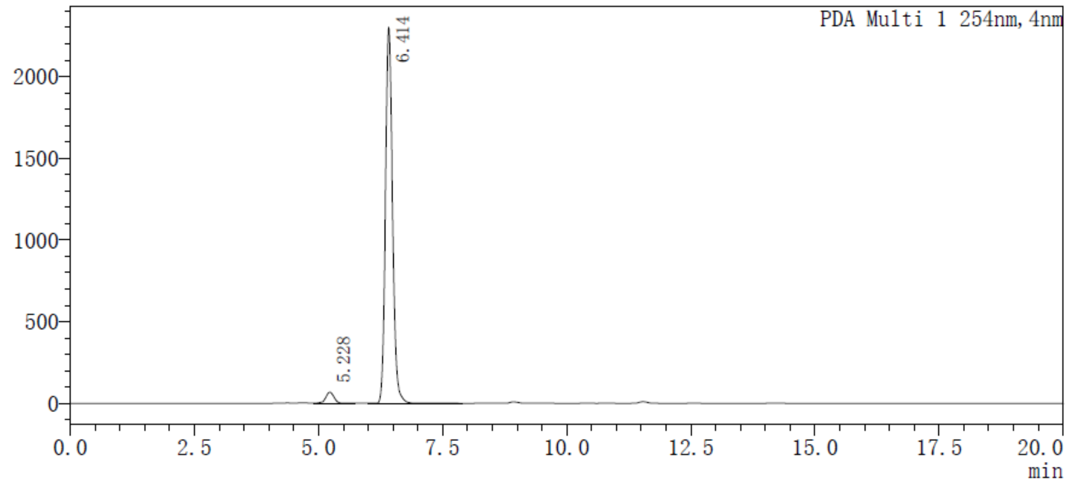

Peak NO	Ret. Time (min)	Area/%
1	5.228	3.504
2	6.414	96.496

(*E*)-3,7-dimethylocta-2,6-dien-1-yl (*R*)-4-(1-phenylpropyl)benzoate (3am)

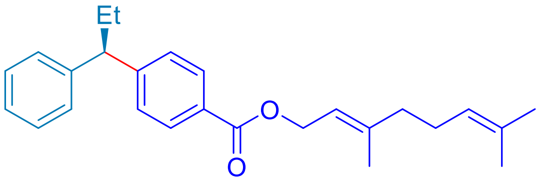

Prepared according to the general procedure **1** from (1-chloropropyl)benzene (0.4 mmol, 2 equiv.) and **2** from (*E*)-3,7-dimethylocta-2,6-dien-1-yl 4-bromobenzoate (0.2 mmol, 1 equiv.). The title compound was isolated (gradient 0–5% EtOAc/hexanes) as a colorless oil (59.1 mg, 79% yield, 90% ee).

^{1}H NMR (400 MHz, $CDCl_3$) δ 7.98 (d, *J* = 8.4 Hz, 2H), 7.34 – 7.26 (m, 4H), 7.25 – 7.15 (m, 3H), 5.46 (dd, *J* = 7.2, 6.0 Hz, 1H), 5.11 (t, *J* = 6.4 Hz, 1H), 4.83 (d, *J* = 7.2 Hz, 2H), 3.86 (t, *J* = 7.6 Hz, 1H), 2.10 (dt, *J* = 10.8, 6.8 Hz, 6H), 1.77 (s, 3H), 1.69 (s, 3H), 1.62 (s, 3H), 0.91 (t, *J* = 7.2 Hz, 3H).

^{13}C NMR (101 MHz, $CDCl_3$) δ 166.6, 150.4, 144.2, 142.2, 131.8, 129.7, 128.5, 128.4, 127.9, 126.3, 123.7, 118.4, 61.7, 53.2, 39.5, 28.3, 26.3, 25.7, 17.7, 16.5, 12.6.

IR (neat): 2961, 1716, 1609, 1453, 1378, 1269, 1180, 1101, 1018, 800, 756 cm^{-1}.

$[\alpha]_D$ ^{26} = +0.53 (*c* = 0.9, $CHCl_3$).

Enantiomeric excess = 90%, SFC analysis (OJ-3, 2% MeOH in CO_2, 1.0 mL/min, T = 25 °C, λ = 214 nm): t_R = 13.064 min (minor), t_R = 14.760 min (major).

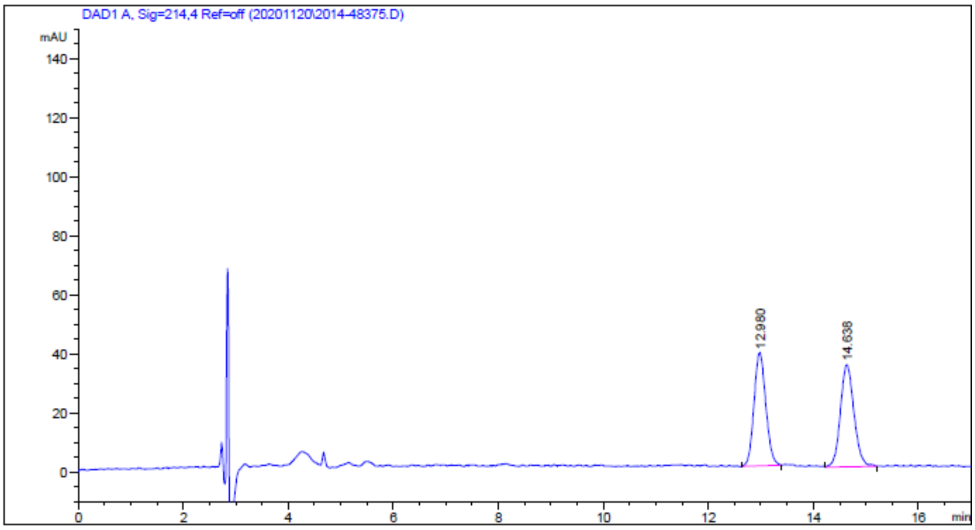

Peak NO	Ret. Time (min)	Area/%
1	12.980	49.0633
2	14.638	50.9367

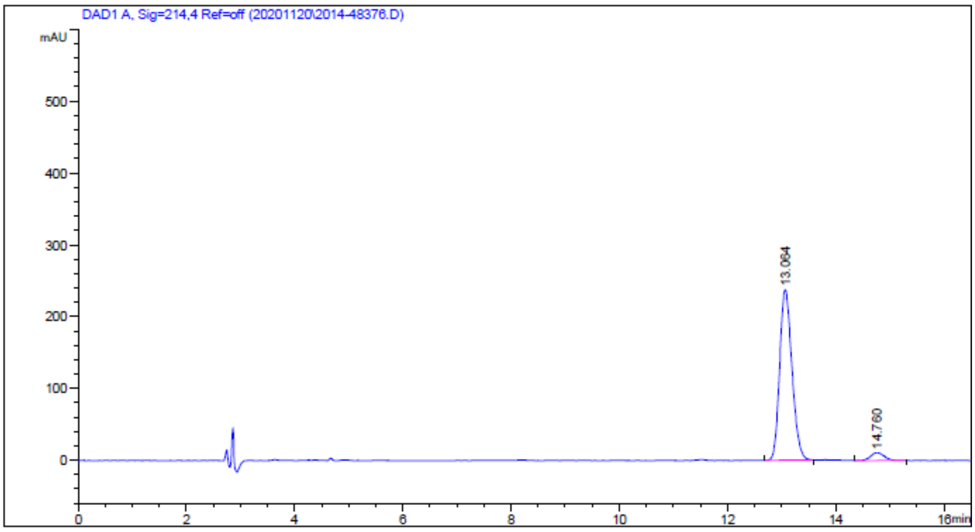

Peak NO	Ret. Time (min)	Area/%
1	13.064	95.0575
2	14.760	4.9425

((3*S*,5*S*,7*S*)-adamantan-1-yl)methyl 4-((*R*)-1-phenylpropyl)benzoate (3an)

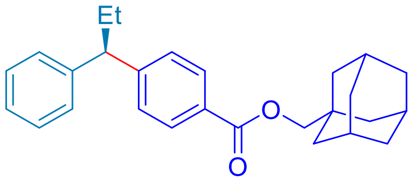

Prepared according to the general procedure **1** from (1-chloropropyl)benzene (0.4 mmol, 2 equiv.) and **2** from ((*3r*,*5r*,*7r*)-adamantan-1-yl)methyl 4-bromobenzoate (0.2 mmol, 1 equiv.). The title compound was isolated (gradient 0–5% EtOAc/hexanes) as a colorless oil (54.1 mg, 70% yield, 87% ee).

^{1}H NMR (400 MHz, CDCl$_3$) δ 7.99 (d, *J* = 8.4 Hz, 2H), 7.33 (d, *J* = 8.0 Hz, 2H), 7.31 – 7.27 (m, 2H), 7.25 – 7.18 (m, 3H), 3.91 (s, 2H), 3.87 (t, *J* = 7.6 Hz, 1H), 2.11 (p, *J* = 7.2 Hz, 2H), 2.02 (s, 3H), 1.78 – 1.67 (m, 7H), 1.64 (d, *J* = 2.0 Hz, 5H), 0.92 (t, *J* = 7.2 Hz, 3H).

^{13}C NMR (101 MHz, CDCl$_3$) δ 166.7, 150.5, 144.3, 129.8, 128.5, 128.5, 128.0, 127.9, 126.4, 74.3, 53.3, 39.5, 37.0, 33.5, 28.4, 28.1, 12.7.

IR (neat): 2911, 1718, 1261, 1098, 1018, 864, 799, 757, 740, 703 cm^{-1}.

HRMS (EI) calcd for $C_{27}H_{32}O_2$ $[M]^+$:388.2407; found: 388.2397.

$[\alpha]_D$ ^{23} = +2.00 (*c* = 0.2, CHCl$_3$).

Enantiomeric excess = 87%, determined by HPLC (Daicel Chiralpak OJ-H Column, *n*-Hexane:*i*-PrOH = 90:10, flow rate 1.0 mL/min, T = 25 °C, λ = 254 nm): t_R = 4.804

min (minor), t_R = 5.528 min (major).

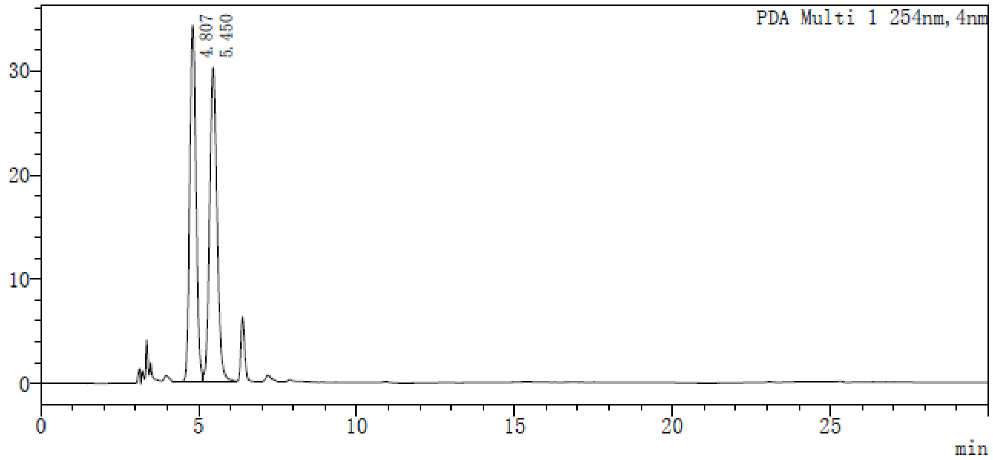

Peak NO	Ret. Time (min)	Area/%
1	4.807	49.150
2	5.450	50.850

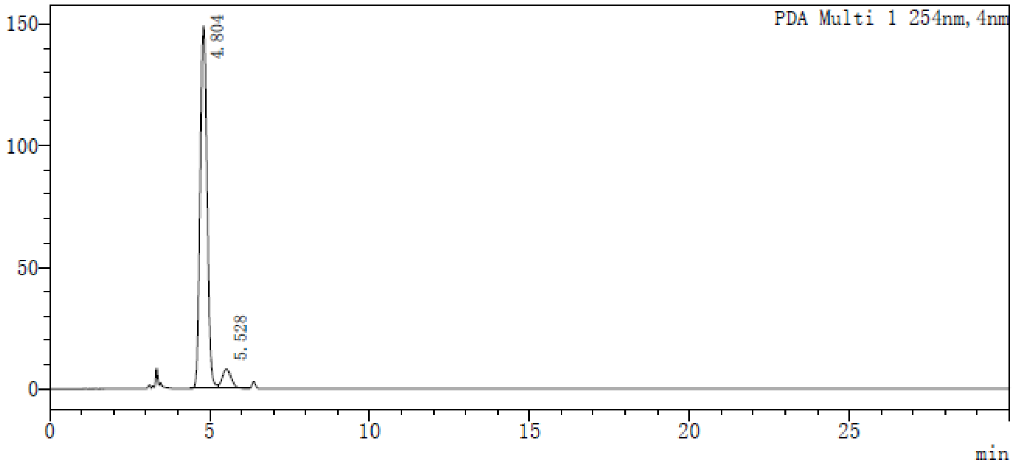

Peak NO	Ret. Time (min)	Area/%
1	4.804	93.297
2	5.528	6.703

(*R*)-2-(1-(3,4,5-trimethoxyphenyl)ethyl)naphthalene (3ao)[1]

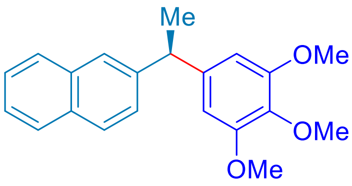

Prepared according to the general procedure **1** from 2-(1-chloroethyl)naphthalene (0.4 mmol, 2 equiv.) and **2** from 5-bromo-1,2,3-trimethoxybenzene (0.2 mmol, 1 equiv.). The title compound was isolated (gradient 0–5% EtOAc/hexanes) as a white solid (31.0

mg, 48% yield, 57% ee).

^{1}H NMR (400 MHz, CDCl$_3$) δ 7.84 – 7.78 (m, 2H), 7.76 (d, J = 8.4 Hz, 1H), 7.69 (s, 1H), 7.50 – 7.40 (m, 2H), 7.33 (dd, J = 8.4, 1.6 Hz, 1H), 6.48 (s, 2H), 4.25 (q, J = 7.2 Hz, 1H), 3.83 (s, 3H), 3.80 (s, 6H), 1.72 (d, J = 7.2 Hz, 3H).

^{13}C NMR (101 MHz, CDCl$_3$) δ 153.1, 143.6, 141.9, 136.3, 133.5, 132.1, 128.0, 127.8, 127.6, 126.7, 126.0, 125.4, 125.3, 104.9, 60.8, 56.1, 45.1, 21.9.

$[\alpha]_D$ ^{27} = -10.3 (c = 0.8, CHCl$_3$).

Enantiomeric excess = 57%, determined by HPLC (Daicel Chiralpak OB-H Column, n-Hexane:i-PrOH = 85:15, flow rate 1.0 mL/min, T = 25 °C, λ = 214 nm): t_R = 7.680 min (minor), t_R = 8.425 min (major).

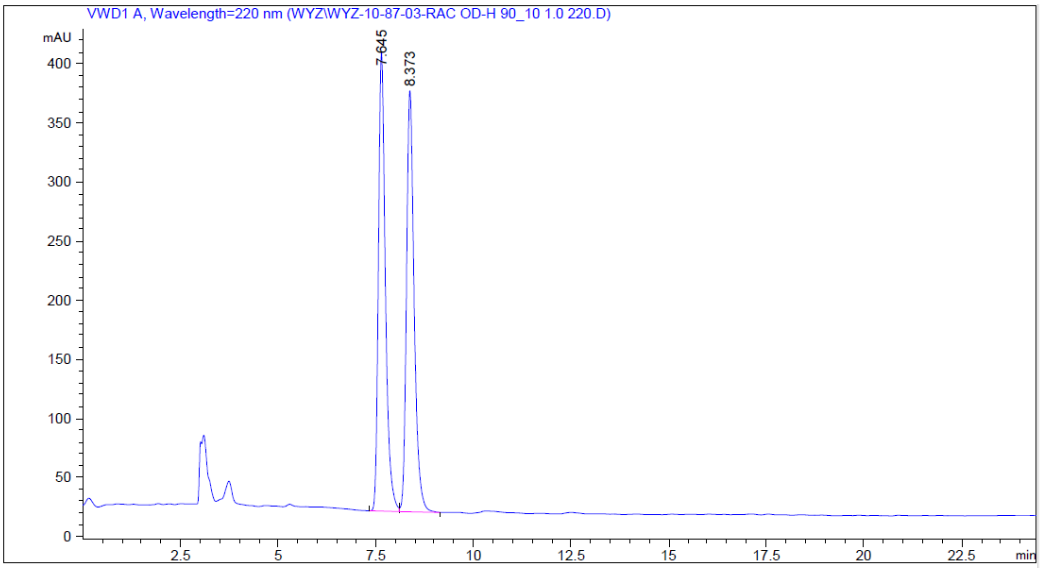

Peak NO	Ret. Time (min)	Area/%
1	7.645	49.9084
2	8.373	50.0916

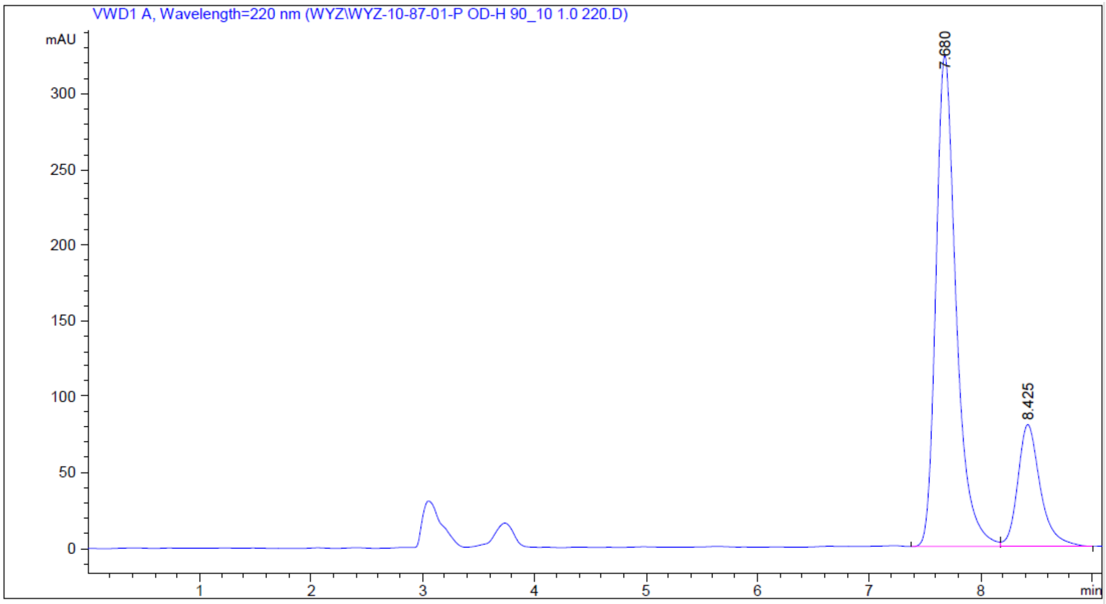

Peak NO	Ret. Time (min)	Area/%
1	7.680	78.3792
2	8.425	21.6208

(*R*)-4-(3,4-dichlorophenyl)-3,4-dihydronaphthalen-1(2*H*)-one (3ap)[9]

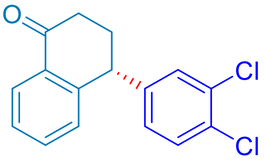

Prepared according to the general procedure **1** from 4-chloro-3,4-dihydronaphthalen-1(2H)-one (0.4 mmol, 2 equiv.) and **2** from 4-bromo-1,2-dichlorobenzene (0.2 mmol, 1 equiv.). The title compound was isolated (gradient 0–5% EtOAc/hexanes) as a white solid (25.1 mg, 43% yield, 87% ee).

^{1}H NMR (400 MHz, $CDCl_3$) δ 8.14 (dd, *J* = 7.6, 1.2 Hz, 1H), 7.49 (td, *J* = 7.6, 1.4 Hz, 1H), 7.40 (t, *J* = 7.6 Hz, 2H), 7.25 (d, *J* = 2.0 Hz, 1H), 6.97 (d, *J* = 8.0 Hz, 2H), 4.30 (dd, *J* = 8.0, 4.4 Hz, 1H), 2.80 – 2.58 (m, 2H), 2.48 (tt, *J* = 8.0, 4.4 Hz, 1H), 2.33 – 2.21 (m, 1H).

^{13}C NMR (101 MHz, $CDCl_3$) δ 197.4, 144.9, 144.1, 133.9, 132.8, 132.7, 131.0, 130.6, 130.6, 129.3, 128.0, 127.6, 127.4, 44.6, 36.6, 31.7.

$[\alpha]_D$ ^{26} = -32.8 (*c* = 0.6, $CHCl_3$).

Enantiomeric excess = 87%, determined by HPLC (Daicel Chiralpak OD-H Column, *n*-Hexane:*i*-PrOH = 98:2, flow rate 0.5 mL/min, T = 25 °C, λ = 254 nm): t_R = 24.282 min (minor), t_R = 27.110 min (major).

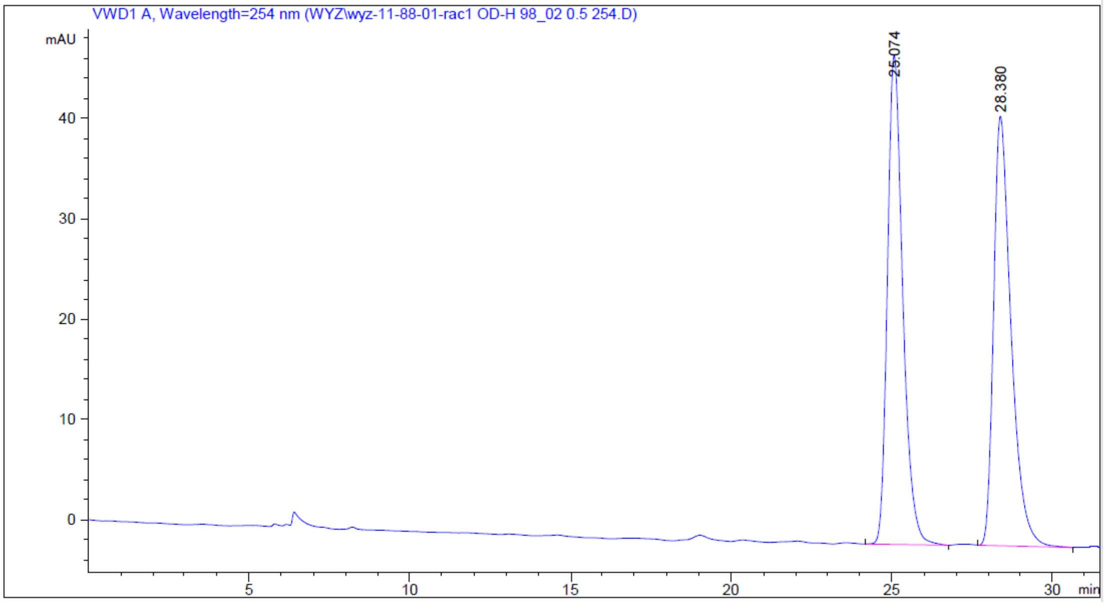

Peak NO	Ret. Time (min)	Area/%
1	25.074	49.9444
2	28.380	50.0556

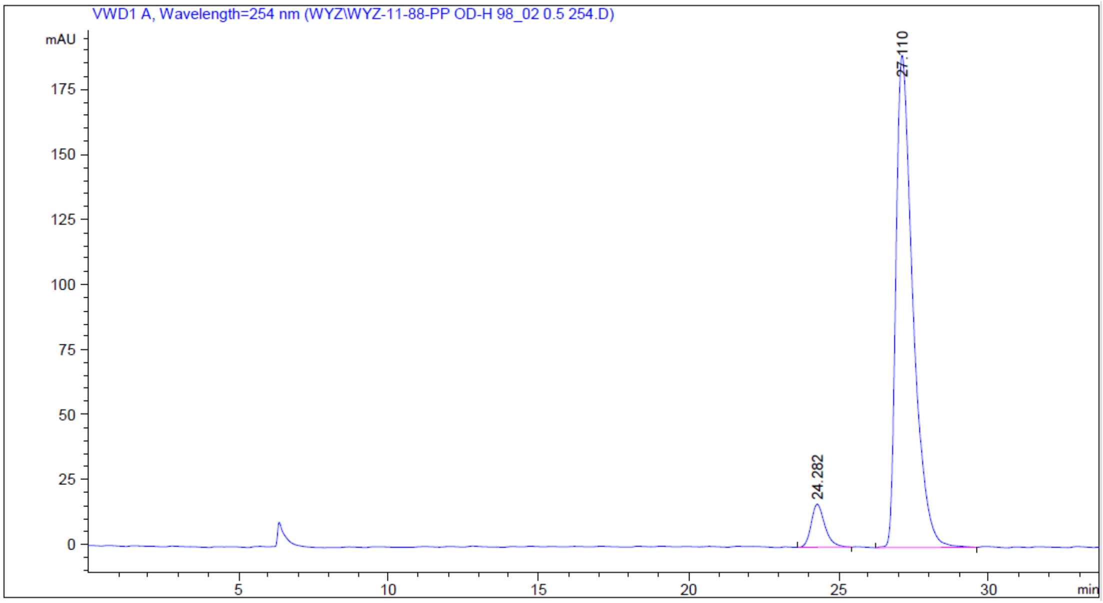

Peak NO	Ret. Time (min)	Area/%
1	24.282	6.5902
2	27.110	93.4098

(3*S*,8*R*,9*S*,10*S*,13*R*,14*S*,17R)-10,13-dimethyl-17-((*R*)-5-methylhexan-2-yl)hexadecahydro-1H-cyclopenta[a]phenanthren-3-yl 4-((*R*)-1-phenylpropyl)benzoate (3aq)

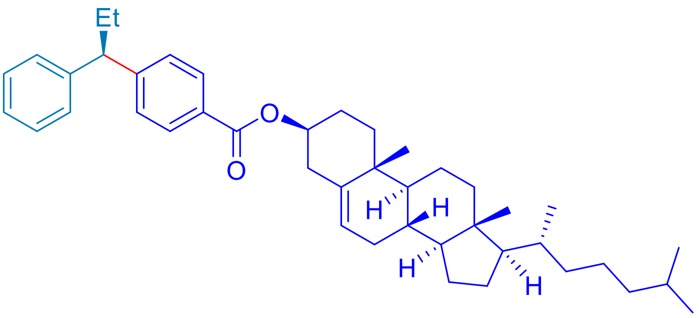

Prepared according to the general procedure **1** from (1-chloropropyl)benzene (0.4 mmol, 2 equiv.) and **2** from (*3S*,*8S*,*9S*,*10R*,*13R*,*14S*,*17R*)-10,13-dimethyl-17-((R)-6-methylheptan-2-yl)-2,3,4,7,8,9,10,11,12,13,14,15,16,17-tetradecahydro-1*H*-cyclopenta[a]phenanthren-3-yl 4-bromobenzoate (0.2 mmol, 1 equiv.). The title compound was isolated (gradient 0–5% EtOAc/hexanes) as a colorless oil (57.0 mg, 48% yield, 89% de).

^{1}H NMR (400 MHz, $CDCl_3$) δ 7.95 (d, *J* = 8.0 Hz, 2H), 7.30 – 7.25 (m, 4H), 7.21 – 7.15 (m, 3H), 5.40 (d, *J* = 4.0 Hz, 1H), 4.86 – 4.80 (m, 1H), 3.84 (t, *J* = 7.8 Hz, 1H), 2.43 (d, *J* = 8.0 Hz, 2H), 2.10 – 2.07 (m, 2H), 1.98 – 1.00 (m, 29H), 0.93 – 0.84 (m, 12H), 0.69 (s, 3H).

^{13}C NMR (101 MHz, $CDCl_3$) δ 165.9, 150.3, 144.3, 139.7, 129.7, 128.7, 128.5, 127.9, 126.3, 122.7, 74.4, 56.7, 56.2, 53.2, 50.0, 42.3, 39.8, 39.5, 38.3, 37.1, 36.7, 36.2, 35.8, 31.9, 28.3, 27.9, 24.3, 23.9, 22.9, 22.6, 21.1, 19.4, 18.7, 12.7, 11.9.

IR (neat): 2954, 1713, 1463, 1272, 1180, 1112, 1017, 799, 757, 739, 704 cm^{-1}.

$[\alpha]_D^{25}$ = -3.64 (*c* = 0.5, $CHCl_3$).

Diastereomeric excess = 89%, SFC analysis (OJ-3, 2% MeOH in CO_2, 1.0 mL/min, T = 25 °C, λ = 214 nm): t_R = 6.207 min (minor), t_R = 6.588 min (major).

Additional Info : Peak(s) manually integrated

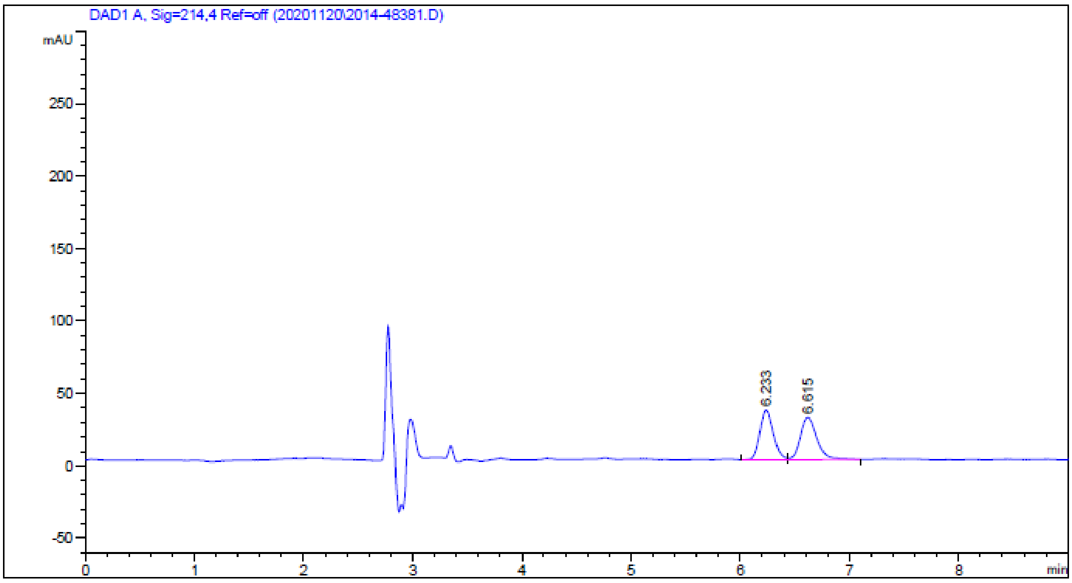

Peak NO	Ret. Time (min)	Area/%
1	6.233	49.1582
2	6.615	50.8418

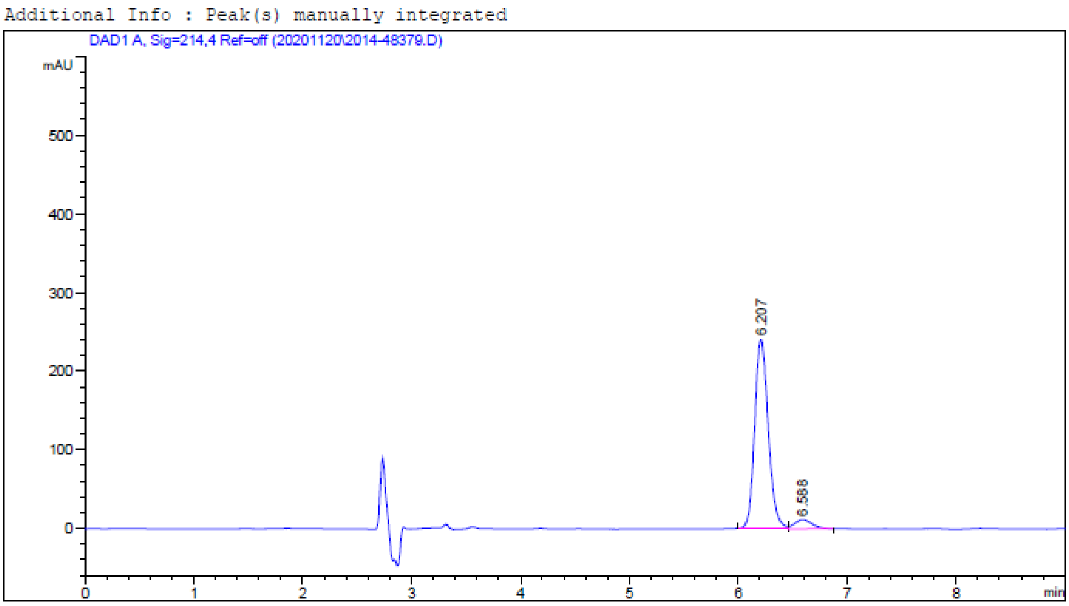

Peak NO	Ret. Time (min)	Area/%
1	6.207	94.3956
2	6.588	5.6044

(*1R,2S,5R*)-2,5-diisopropylcyclohexyl 4-((*R*)-1-phenylpropyl)benzoate (3ar)

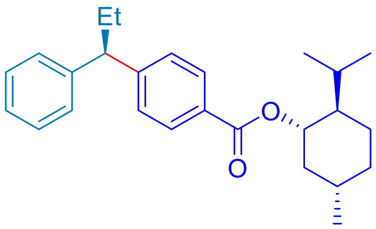

Prepared according to the general procedure **1** from (1-chloropropyl)benzene (0.4 mmol, 2 equiv.) and **2** from (*1S*,*2R*,*5S*)-2-isopropyl-5-methylcyclohexyl 4-bromobenzoate (0.2 mmol, 1 equiv.). The title compound was isolated (gradient 0–5% EtOAc/hexanes) as a colorless oil (52.8 mg, 65% yield, 92% de).

^{1}H NMR (400 MHz, $CDCl_3$) δ 7.98 (d, *J* = 8.4 Hz, 2H), 7.38 – 7.26 (m, 4H), 7.26 – 7.15 (m, 3H), 4.92 (td, *J* = 10.8, 4.4 Hz, 1H), 3.87 (t, *J* = 7.6 Hz, 1H), 2.17 – 2.06 (m, 3H), 1.96 (tt, *J* = 9.6, 3.6 Hz, 1H), 1.73 (d, *J* = 11.6 Hz, 2H), 1.63 – 1.49 (m, 2H), 1.18 (s, 2H), 0.93 (t, *J* = 6.4 Hz, 9H), 0.79 (d, *J* = 6.8 Hz, 3H).

^{13}C NMR (101 MHz, $CDCl_3$) δ 166.1, 150.3, 144.3, 129.8, 128.7, 128.5, 127.9 (d, *J* = 2.8 Hz), 126.3, 74.6, 53.3, 47.3, 41.0, 34.4, 31.5, 28.3, 26.5, 23.6, 22.1, 20.8, 16.5, 12.7.

IR (neat): 2957, 1712, 1609, 1453, 1272, 1178, 1110, 1018, 798, 755, 703 cm^{-1}.

HRMS (MALDI) calcd for $C_{28}H_{42}O_2N$ $[M+NH_4]^+$:424.3208; found: 424.3210.

$[\alpha]_D$ ^{26} = +62.05 (*c* = 0.8, $CHCl_3$).

Diastereomeric excess = 92%, determined by HPLC (Daicel Chiralpak OB-H Column, *n*-Hexane:*i*-PrOH = 85:15, flow rate 1.0 mL/min, T = 25 °C, λ = 214 nm): t_R = 7.116 min (minor), t_R = 7.658 min (major).

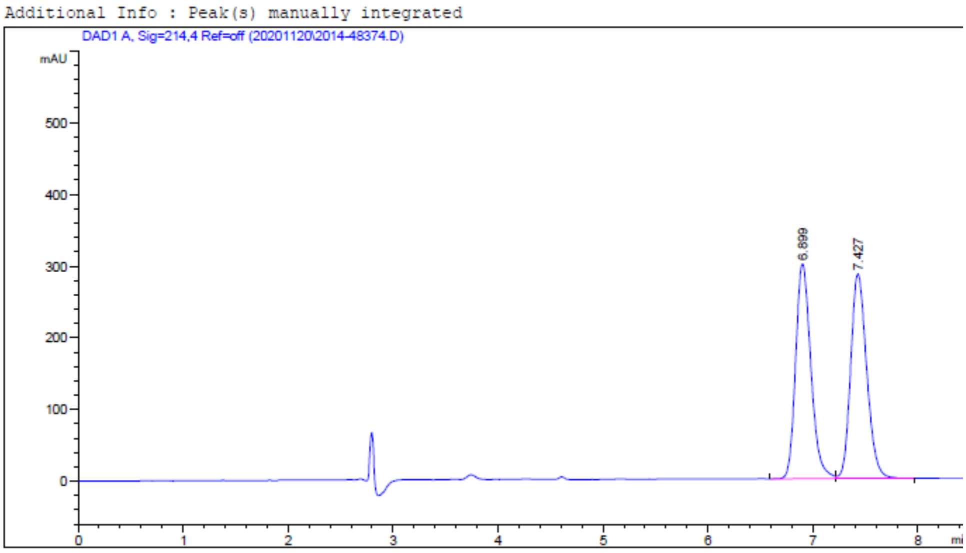

Peak NO	Ret. Time (min)	Area/%
1	6.899	49.9192
2	7.427	50.0808

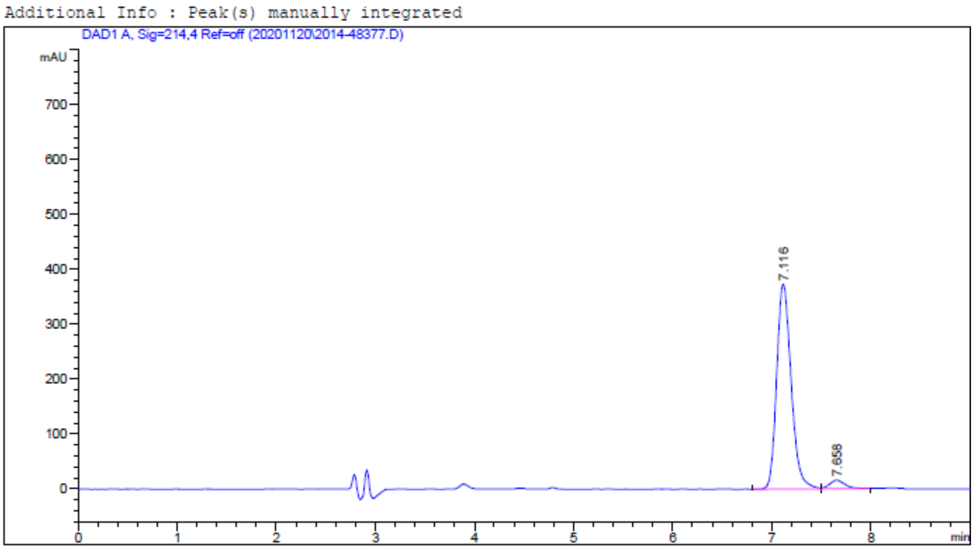

Peak NO	Ret. Time (min)	Area/%
1	7.116	95.9062
2	7.658	4.0938

((3a*R*,5*R*,5a*S*,8a*S*,8b*R*)-2,2,7,7-tetramethyltetrahydro-5*H*-bis([1,3]dioxolo)[4,5-

b:4',5'-d]pyran-5-yl)methyl 4-((*R*)-1-phenylpropyl)benzoate (3as)

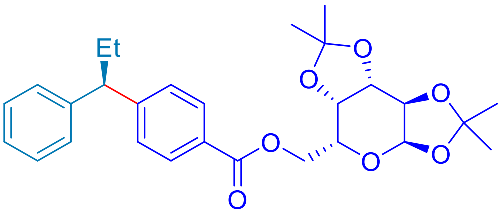

Prepared according to the general procedure **1** from (1-chloropropyl)benzene (0.4 mmol, 2 equiv.) and **2** from ((*3aR*,*5R*,*5aS*,*8aS*,*8bR*)-2,2,7,7-tetramethyltetrahydro-5*H*-bis([1,3]dioxolo)[4,5-b:4',5'-*d*]pyran-5-yl)methyl 4-bromobenzoate (0.2 mmol, 1 equiv.). The title compound was isolated (gradient 0–5% EtOAc/hexanes) as a colorless oil (73.3 mg, 76% yield, 90% de).

^{1}H NMR (400 MHz, CDCl$_3$) δ 7.96 (d, *J* = 8.0 Hz, 2H), 7.31 – 7.26 (m, 4H), 7.24 – 7.14 (m, 3H), 5.55 (d, *J* = 4.8 Hz, 1H), 4.65 – 4.64 (m, 1H), 4.49 (m, 1H), 4.48 – 4.41 (m, 1H), 4.35 – 4.28 (m, 2H), 4.17 – 4.16 (m, 1H), 3.84 (t, *J* = 7.6 Hz, 1H), 2.12 – 2.05 (m, 2H), 1.49 (d, *J* = 13.6 Hz, 6H), 1.34 (d, *J* = 8.8 Hz, 6H), 0.90 (t, *J* = 7.2 Hz, 3H).

^{13}C NMR (101 MHz, CDCl$_3$) δ 166.4, 150.7, 144.2, 129.9, 128.5, 128.4, 128.0, 127.9, 127.9, 126.3, 109.7, 108.8, 96.3, 71.1, 70.7, 70.6, 66.1, 63.7, 53.2, 28.3, 26.0, 25.0, 24.5, 12.7.

IR (neat): 2958, 1720, 1457, 1378, 1259, 1102, 1011, 796, 706, 638 cm^{-1}.

HRMS (ESI) calcd for $C_{28}H_{34}O_7Na$ $[M+Na]^+$:505.21927; found: 505.21967.

$[\alpha]_D$ ^{22} = -46.93 (*c* = 0.4, CHCl$_3$).

Diastereomeric excess = 90%, determined by HPLC (Daicel Chiralpak AD-H Column, *n*-Hexane:*i*-PrOH = 90:10, flow rate 1.0 mL/min, T = 25 °C, λ = 254 nm): t_R = 8.171 min (minor), t_R = 9.577 min (major).

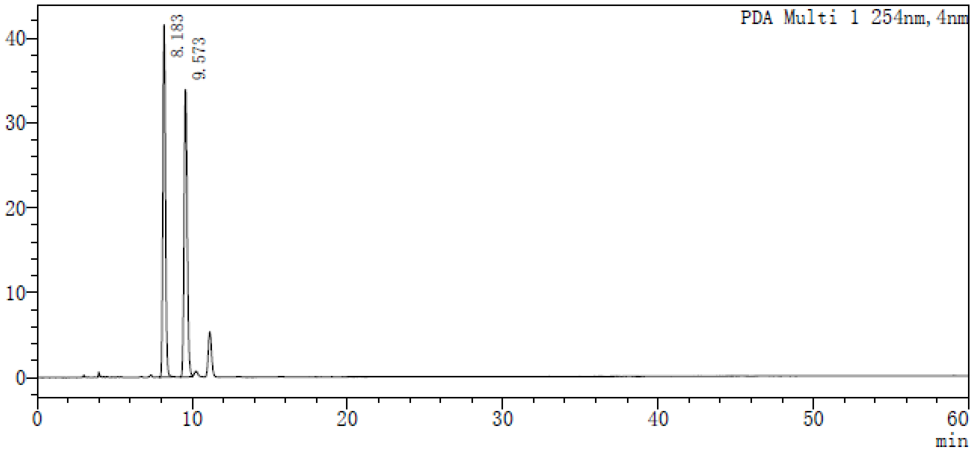

Peak NO	Ret. Time (min)	Area/%
1	8.183	50.047

2	9.573	49.953

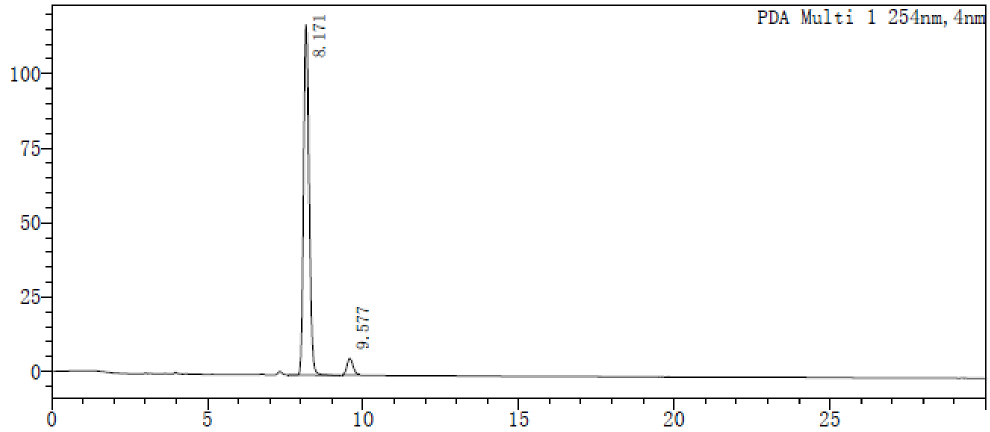

Peak NO	Ret. Time (min)	Area/%
1	8.171	94.809
2	9.577	5.191

3. Supplementary Figures

3.1. X-Ray Crystal Structures

X-Ray Crystal Structure of 3g (CCDC 2312564)

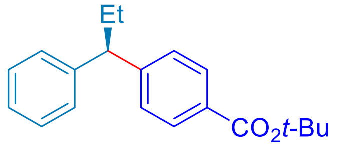

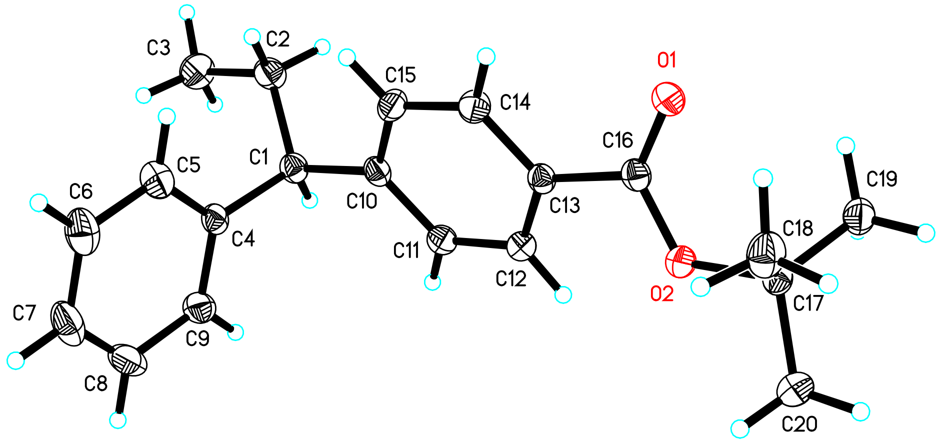

Table. Crystal data and structure refinement for **3g**.

Identification code	mo_d8v23545_0m	
Empirical formula	C20 H24 O2	
Formula weight	296.39	
Temperature	213(2) K	
Wavelength	0.71073 Å	
Crystal system	Monoclinic	
Space group	P 21	
Unit cell dimensions	a = 11.4238(4) Å	= 90°.
	b = 7.0832(2) Å	=
116.7450(10)°.		
	c = 11.7912(4) Å	= 90°.
Volume	852.04(5) Å^3	
Z	2	
Density (calculated)	1.155 Mg/m^3	
Absorption coefficient	0.073 mm^{-1}	

F(000)	320
Crystal size	0.200 x 0.150 x 0.120 mm^3
Theta range for data collection	1.996 to 25.995°.
Index ranges	-14<=h<=14, -8<=k<=8, -14<=l<=14
Reflections collected	12035
Independent reflections	3324 [R(int) = 0.0457]
Completeness to theta = 25.242°	99.6 %
Absorption correction	Semi-empirical from equivalents
Max. and min. transmission	0.7456 and 0.6331
Refinement method	Full-matrix least-squares on F^2
Data / restraints / parameters	3324 / 1 / 204
Goodness-of-fit on F^2	1.056
Final R indices [I>2sigma(I)]	R1 = 0.0339, wR2 = 0.0778
R indices (all data)	R1 = 0.0392, wR2 = 0.0812
Absolute structure parameter	-0.2(6)
Extinction coefficient	0.093(14)
Largest diff. peak and hole	0.111 and -0.110 e.$Å^{-3}$

3.2 ^{1}H NMR, ^{13}C NMR, ^{19}F NMR

Compound 3a ^{1}H NMR (400 MHz, $CDCl_3$) (methyl 4-bromobenzoate)

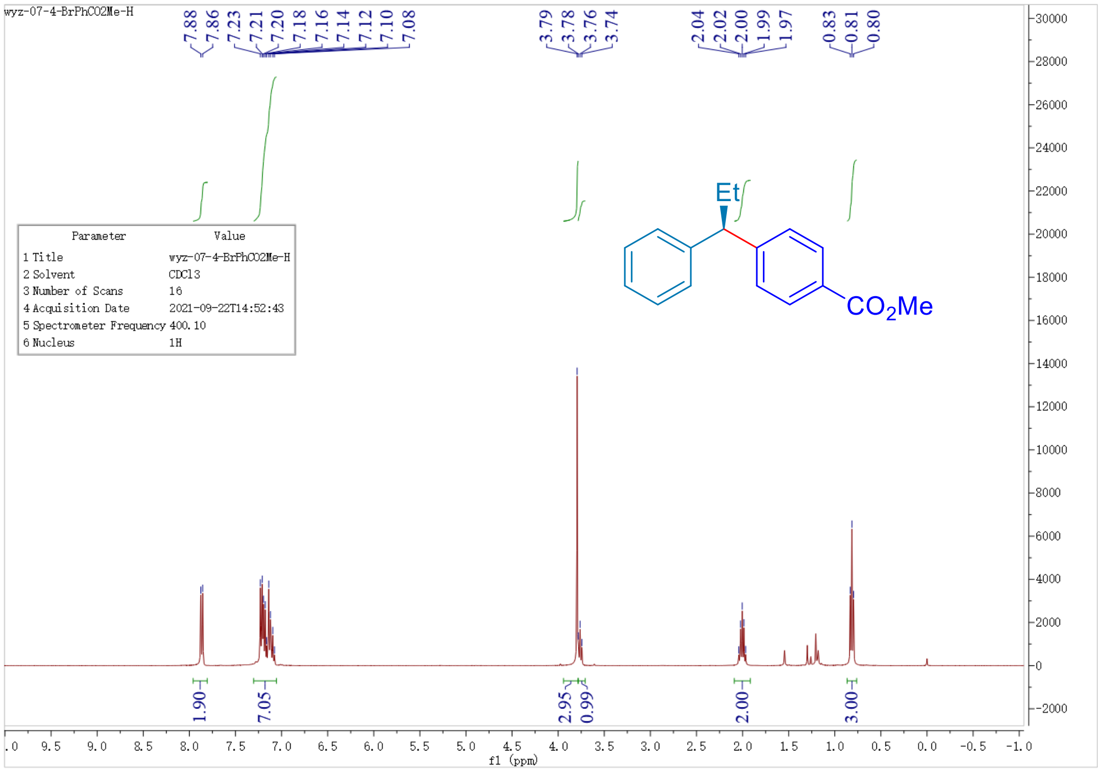

Compound 3a ^{13}C NMR (101 MHz, $CDCl_3$) (methyl 4-bromobenzoate)

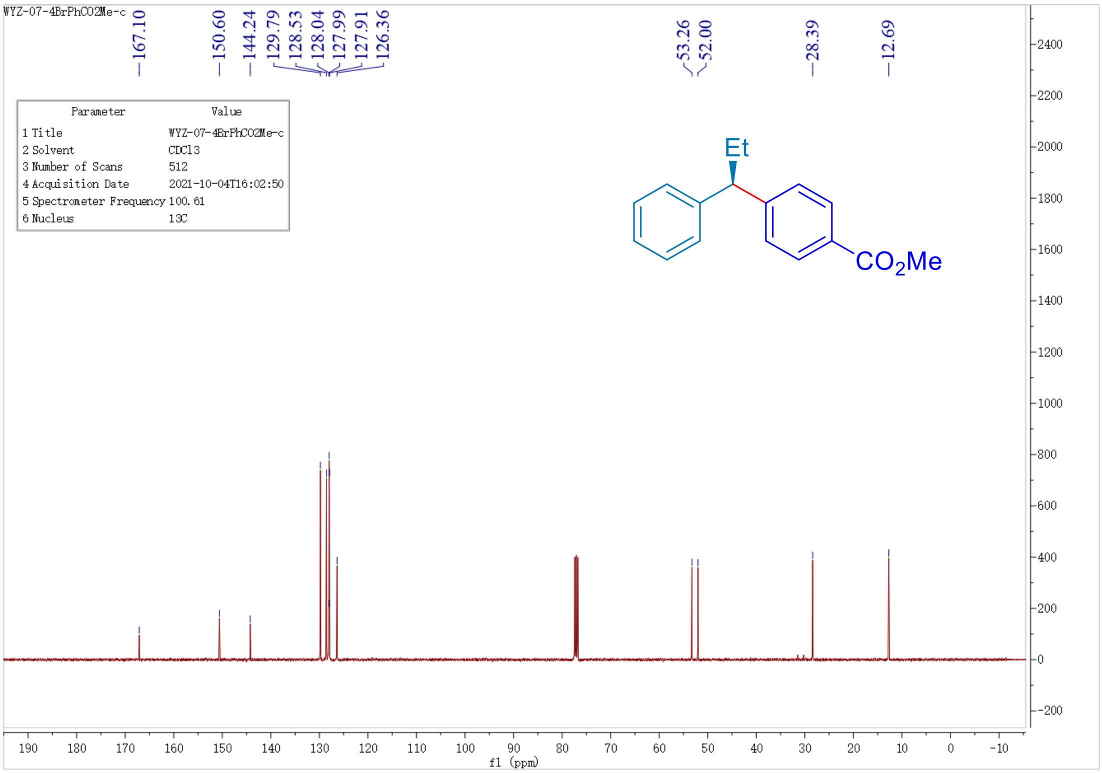

Compound 3a ^{1}H NMR (400 MHz, CDCl$_3$) (methyl 4-chlorobenzoate)

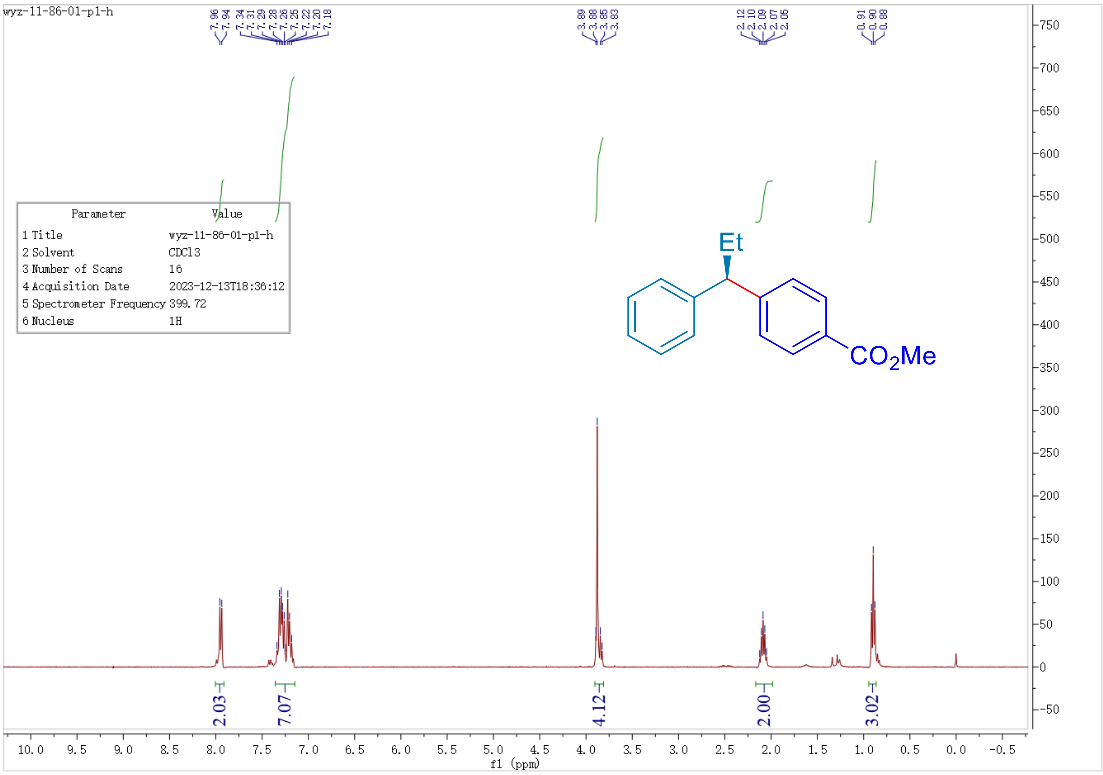

Compound 3a ^{13}C NMR (101 MHz, CDCl$_3$) (methyl 4-chlorobenzoate)

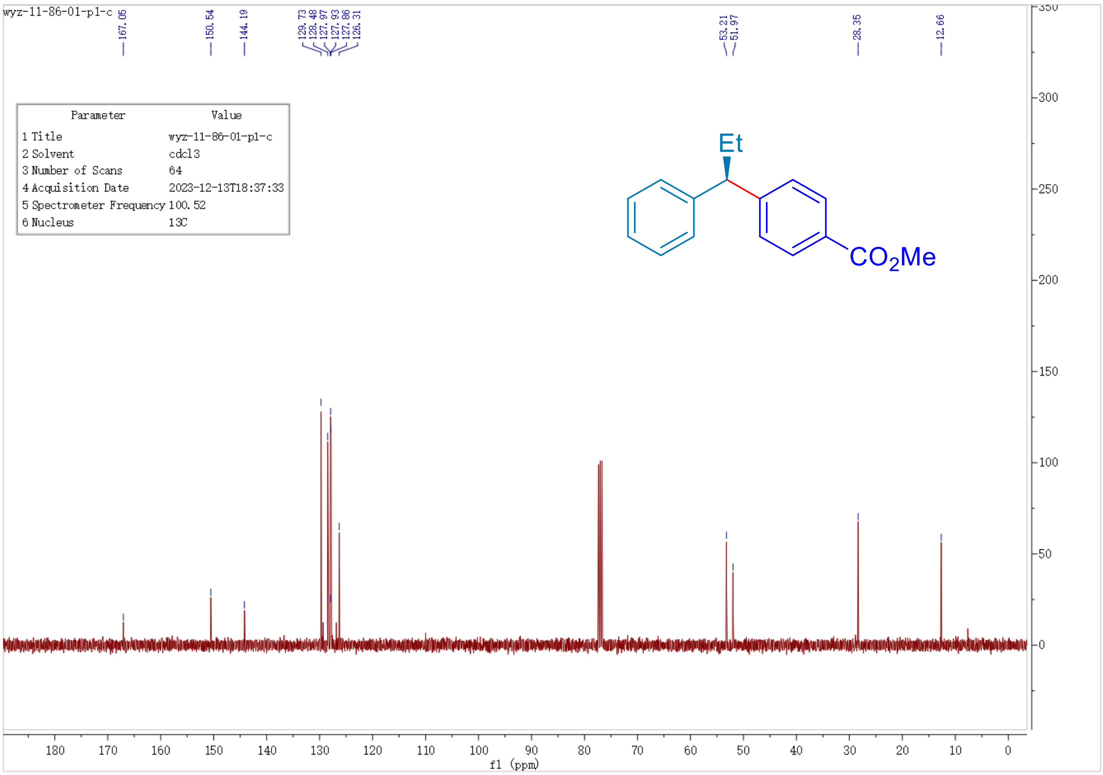

Compound 3a ^{1}H NMR (400 MHz, CDCl3) (methyl 4-(((trifluoromethyl)sulfonyl)oxy)benzoate)

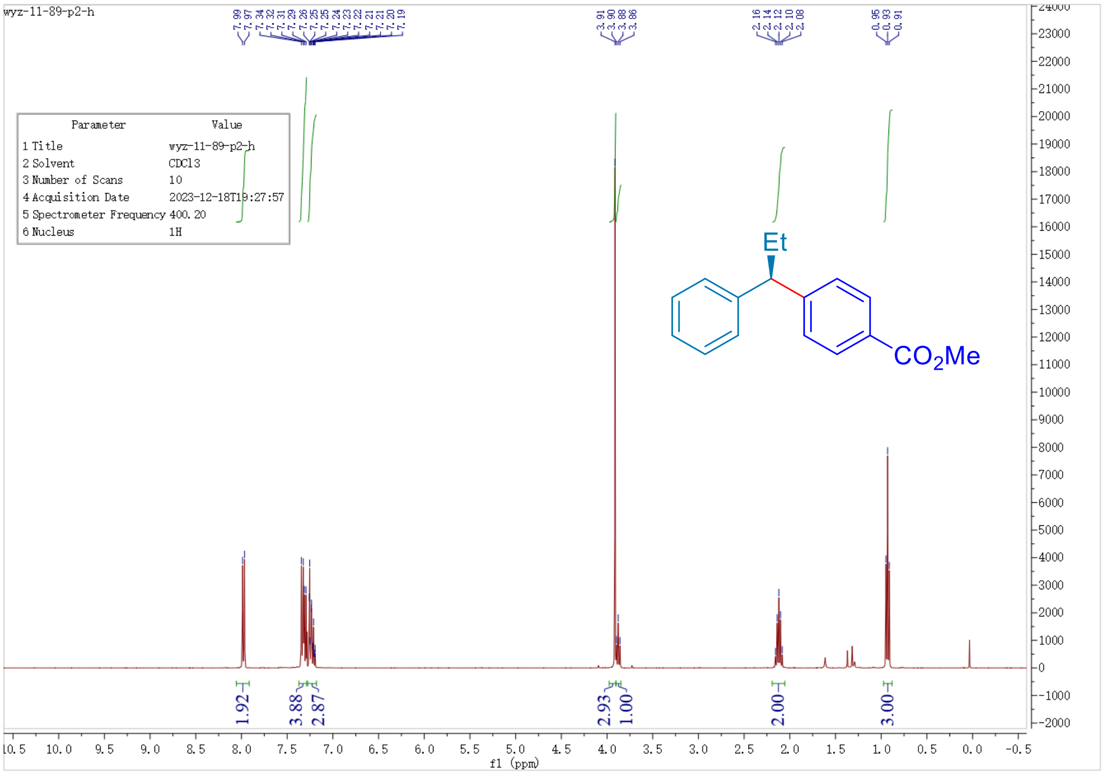

Compound 3a ^{13}C NMR (101 MHz, CDCl3) (methyl 4-(((trifluoromethyl)sulfonyl)oxy)benzoate)

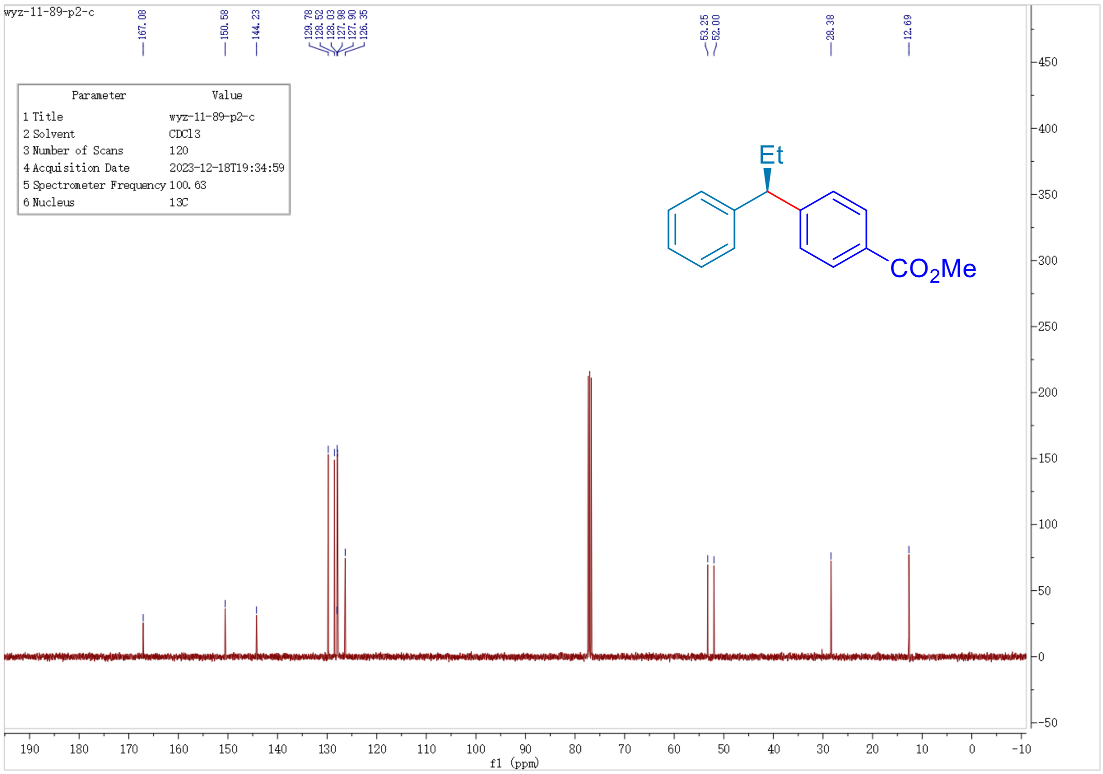

Compound 3b ^{1}H NMR (400 MHz, $CDCl_3$) (ethyl 4-bromobenzoate)

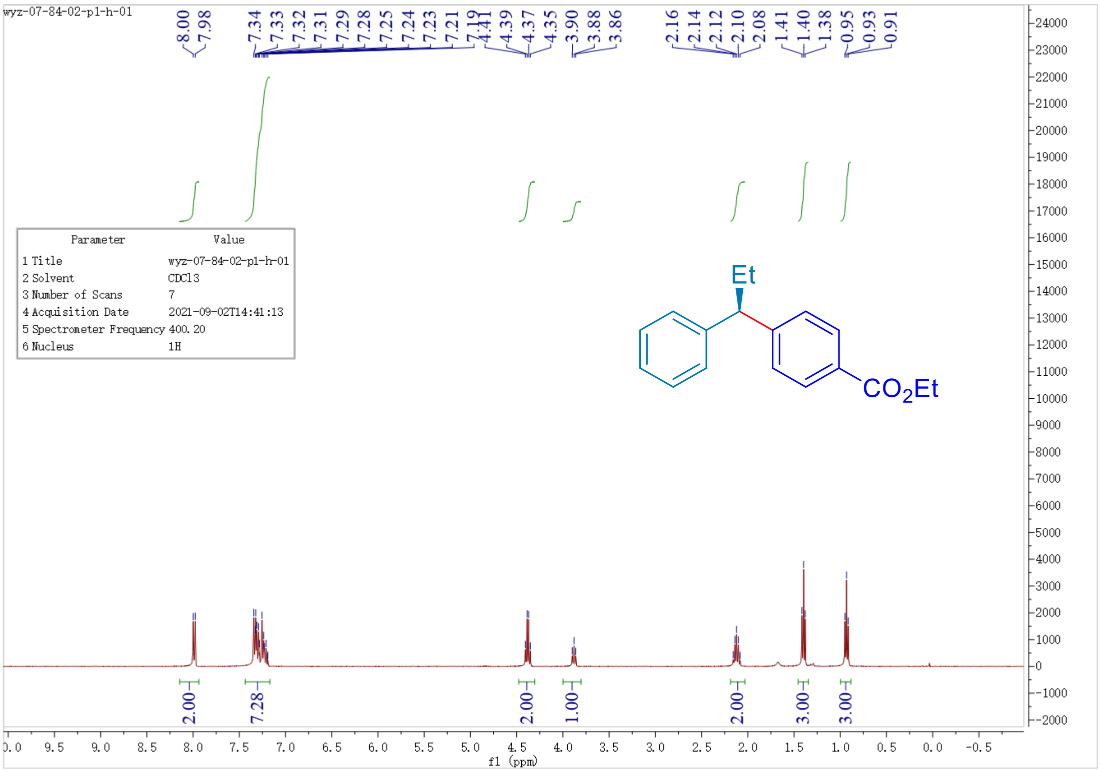

Compound 3b ^{13}C NMR (101 MHz, $CDCl_3$) (ethyl 4-bromobenzoate)

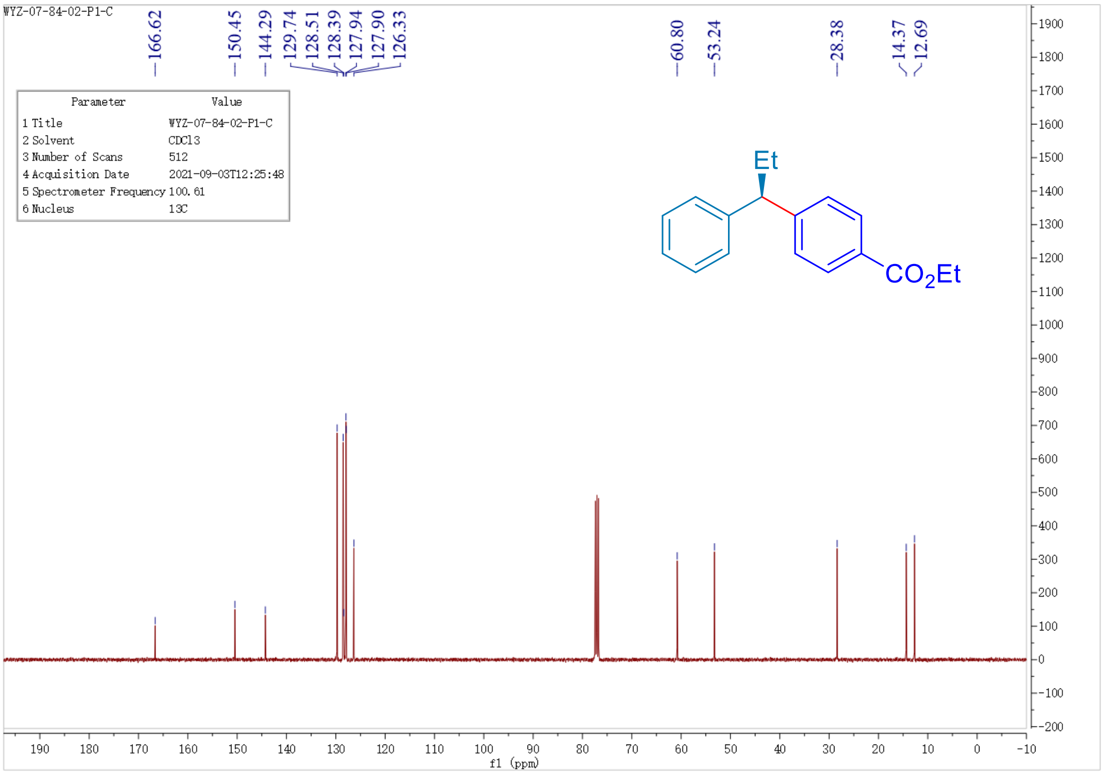

Compound 3b ^{1}H NMR (400 MHz, $CDCl_3$) (ethyl 4-chlorobenzoate)

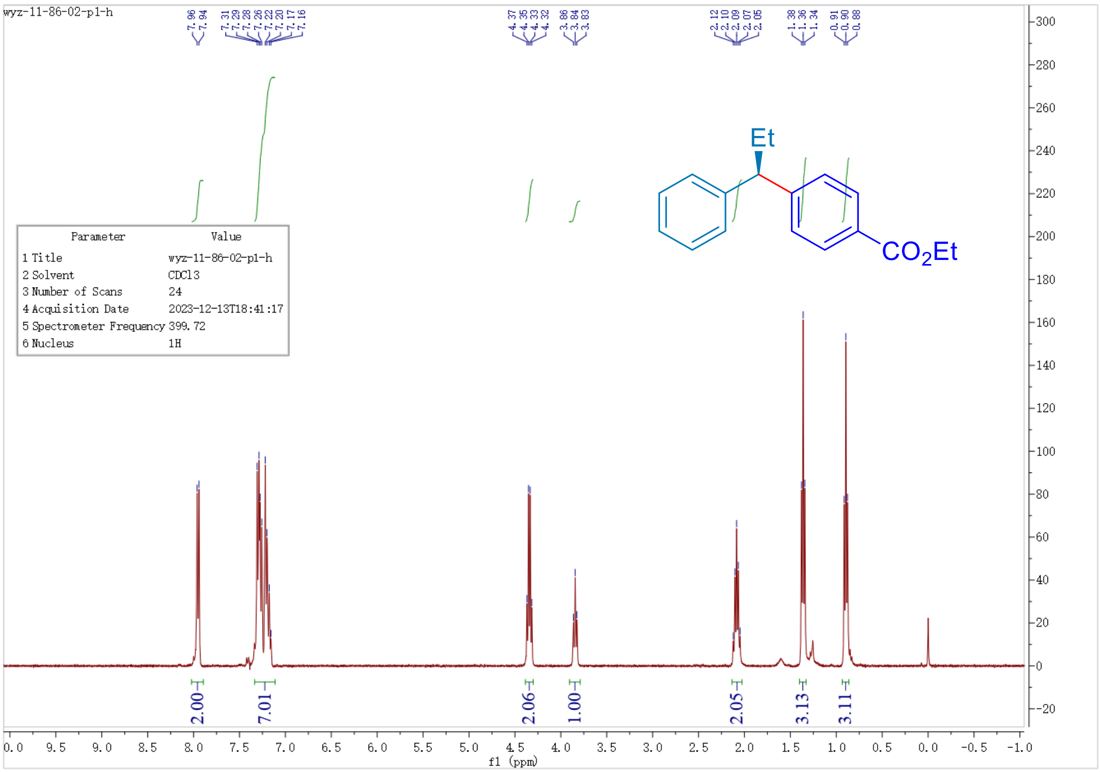

Compound 3b ^{13}C NMR (101 MHz, $CDCl_3$) (ethyl 4-chlorobenzoate)

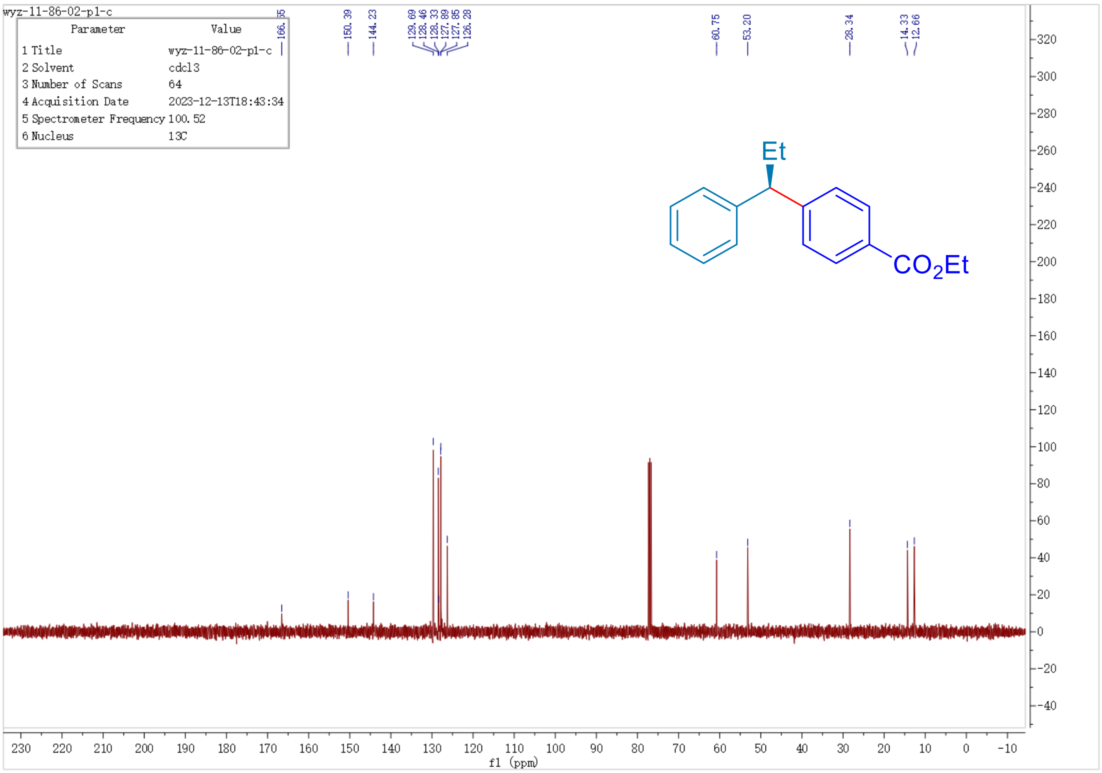

Compound 3c ^{1}H NMR (400 MHz, $CDCl_3$) (1-bromo-4-(methylsulfonyl)benzene)

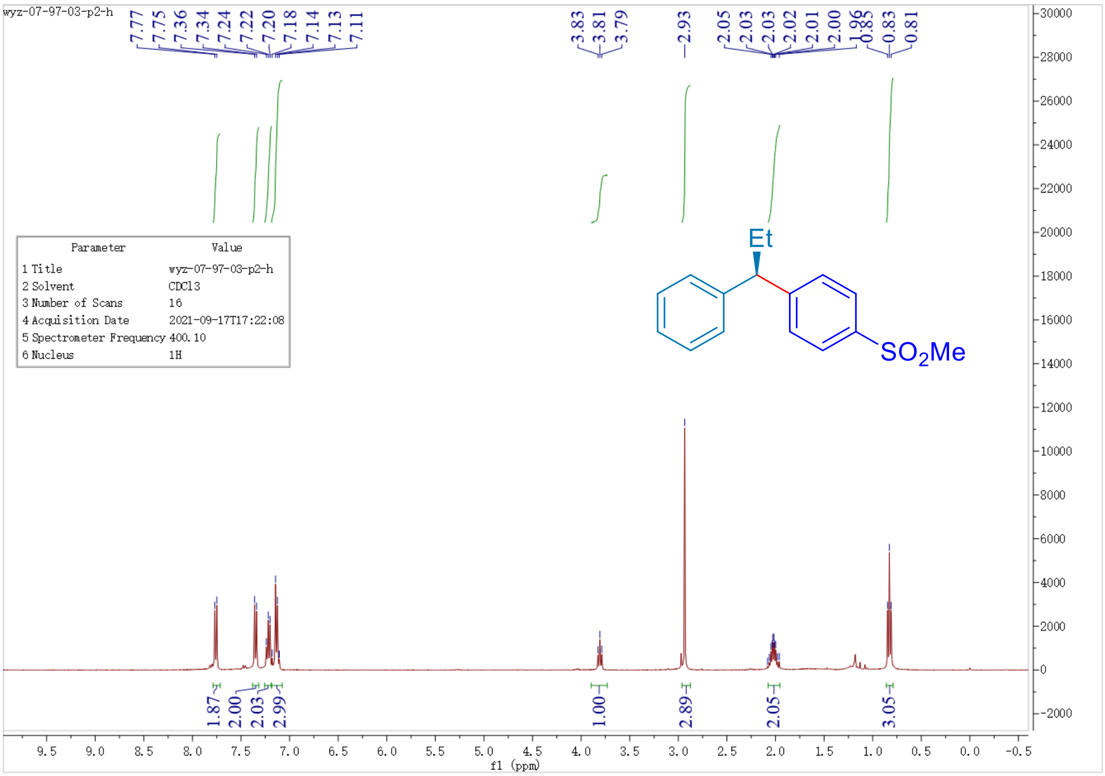

Compound 3c ^{13}C NMR (101 MHz, $CDCl_3$) (1-bromo-4-(methylsulfonyl)benzene)

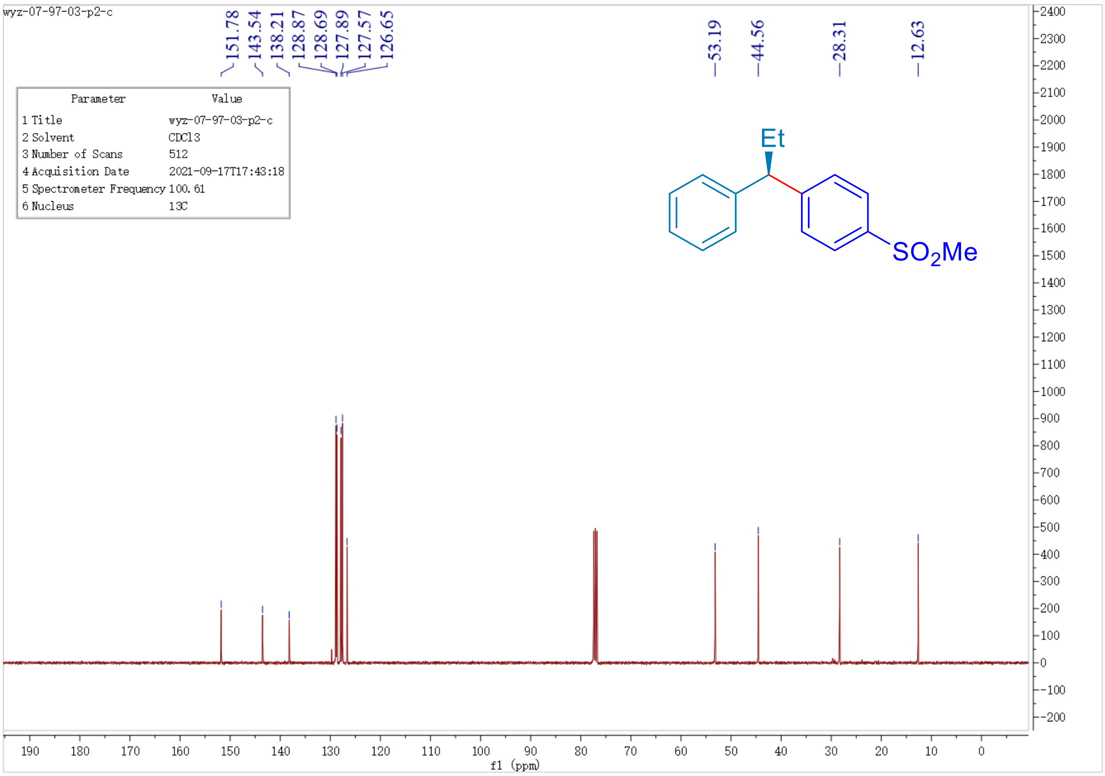

Compound 3c ^{1}H NMR (400 MHz, CDCl₃) (1-chloro-4-(methylsulfonyl)benzene)

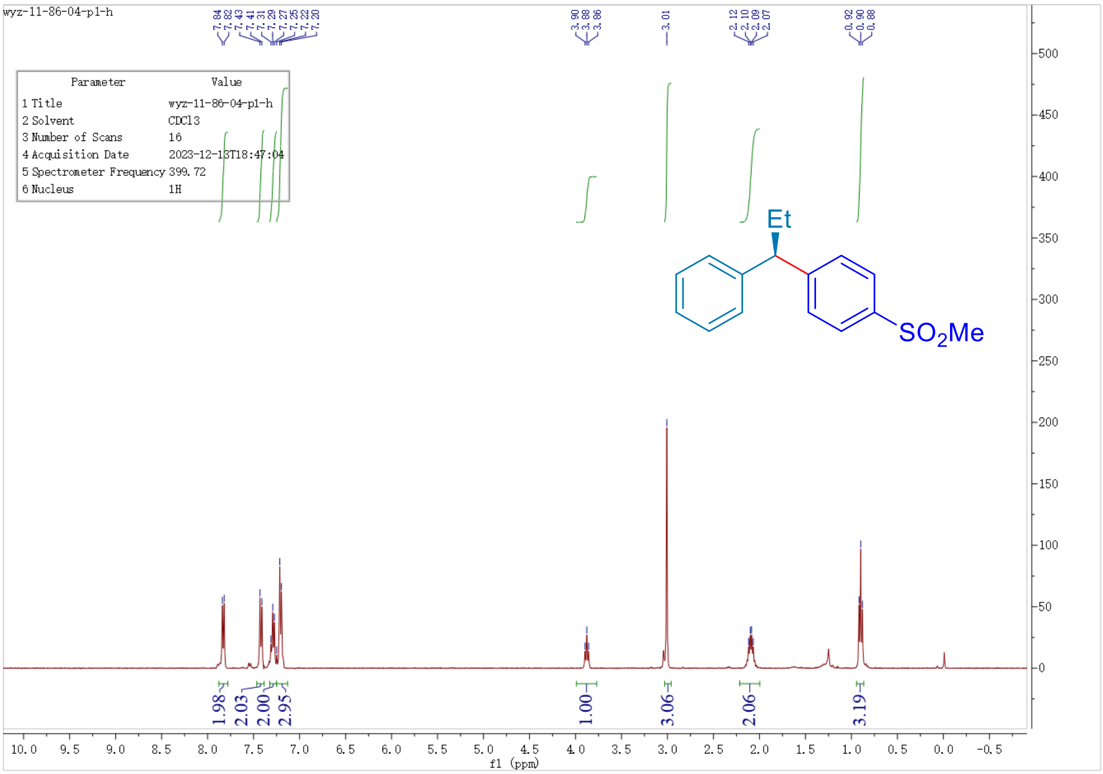

Compound 3c ^{13}C NMR (101 MHz, CDCl₃) (1-chloro-4-(methylsulfonyl)benzene)

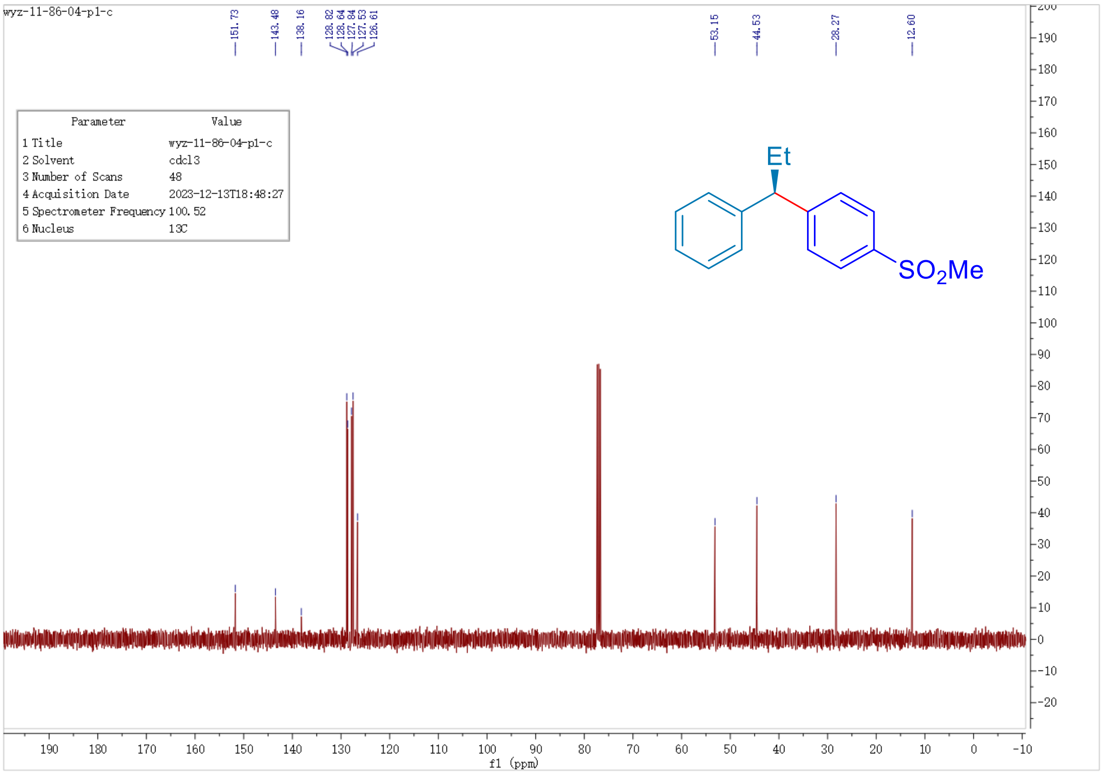

Compound 3d ^{1}H NMR (400 MHz, $CDCl_3$) (4-bromobenzonitrile)

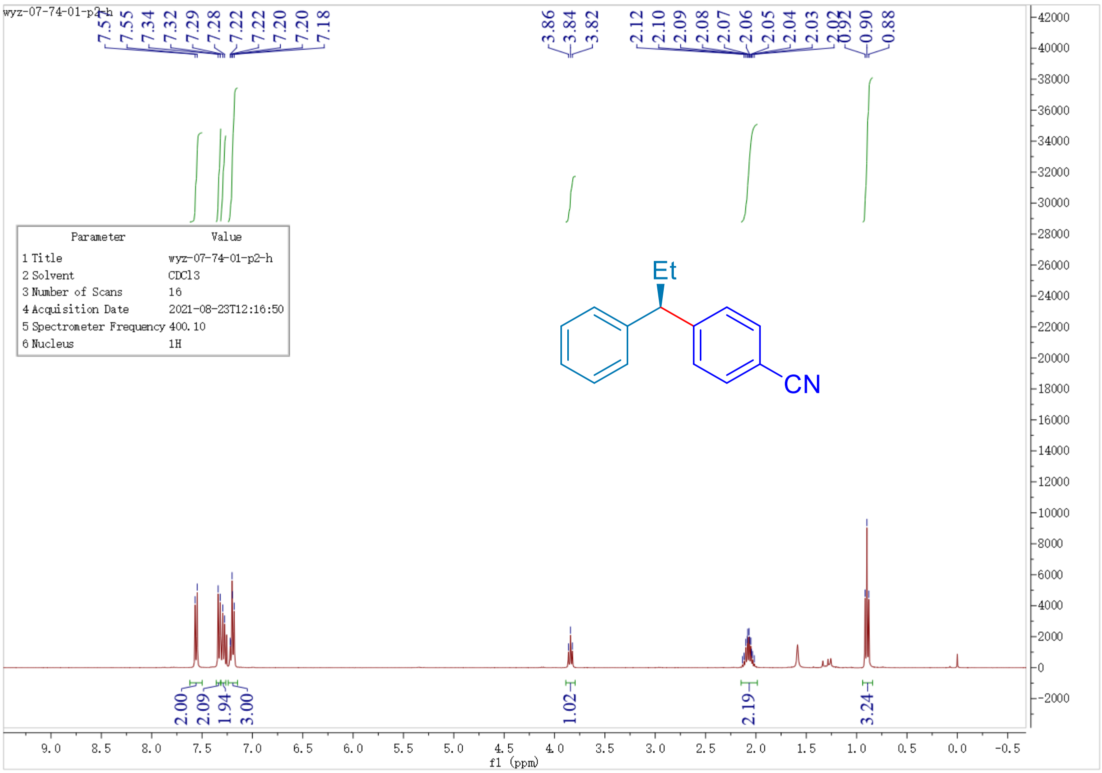

Compound 3d ^{13}C NMR (101 MHz, $CDCl_3$) (4-bromobenzonitrile)

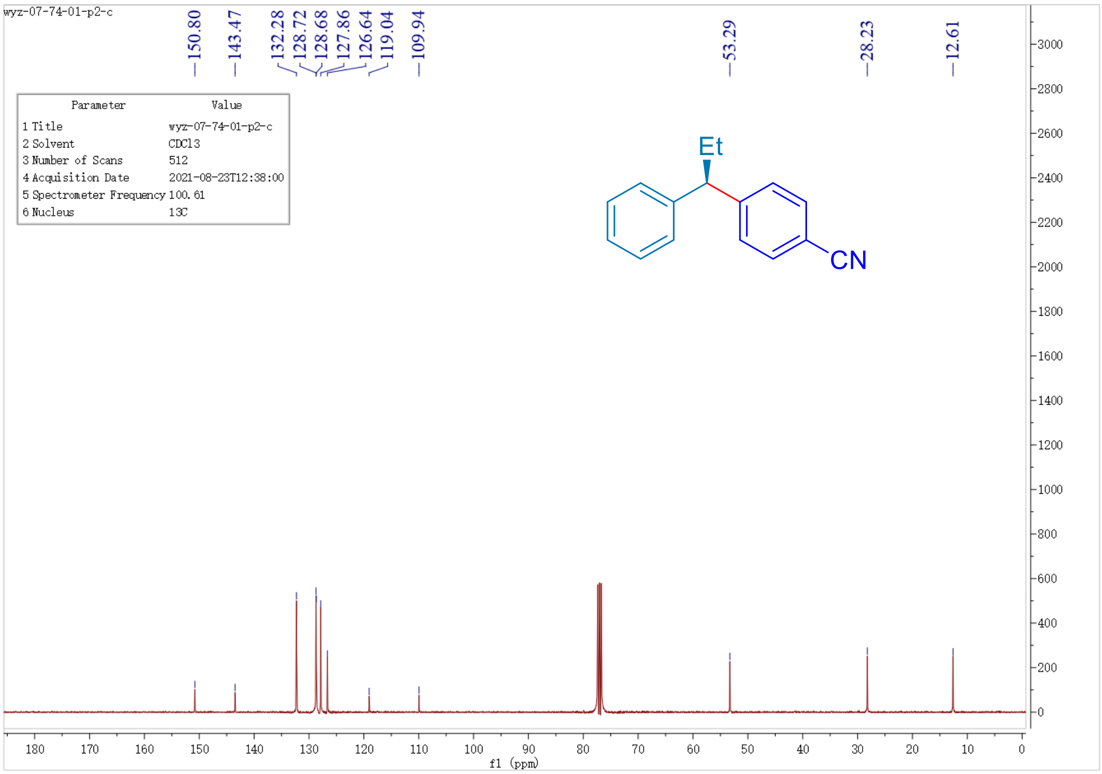

Compound 3d ^{1}H NMR (400 MHz, $CDCl_3$) (4-chlorobenzonitrile)

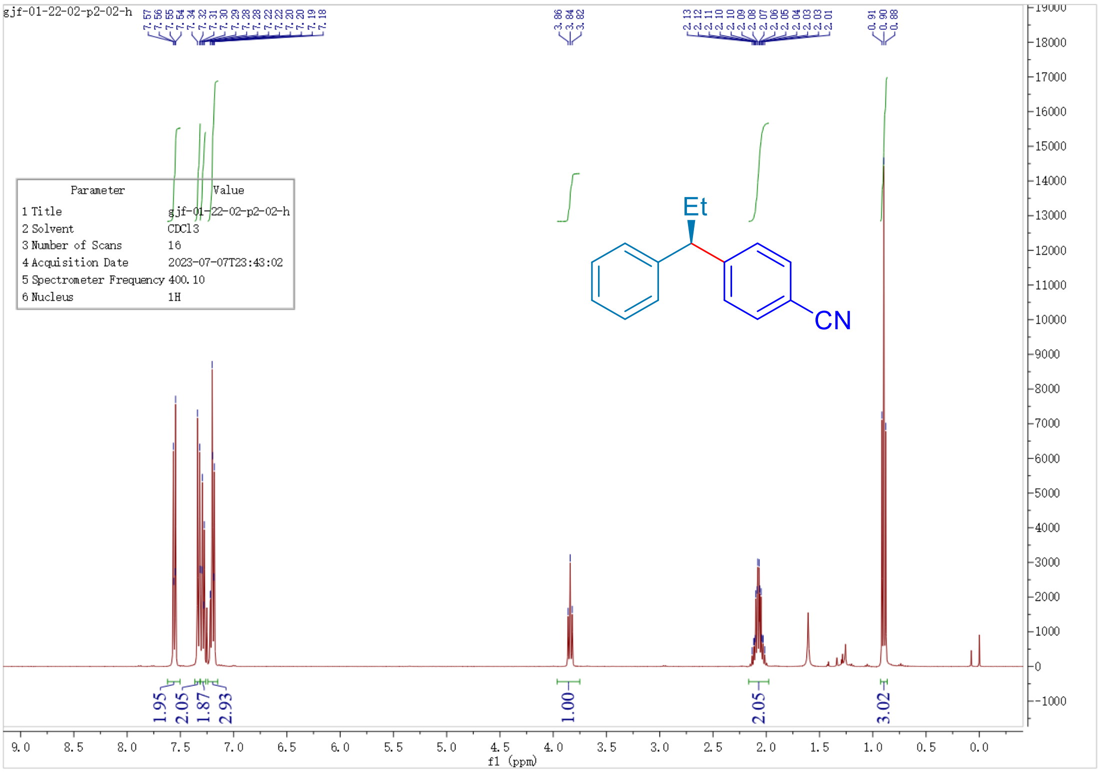

Compound 3d ^{13}C NMR (101 MHz, $CDCl_3$) (4-chlorobenzonitrile)

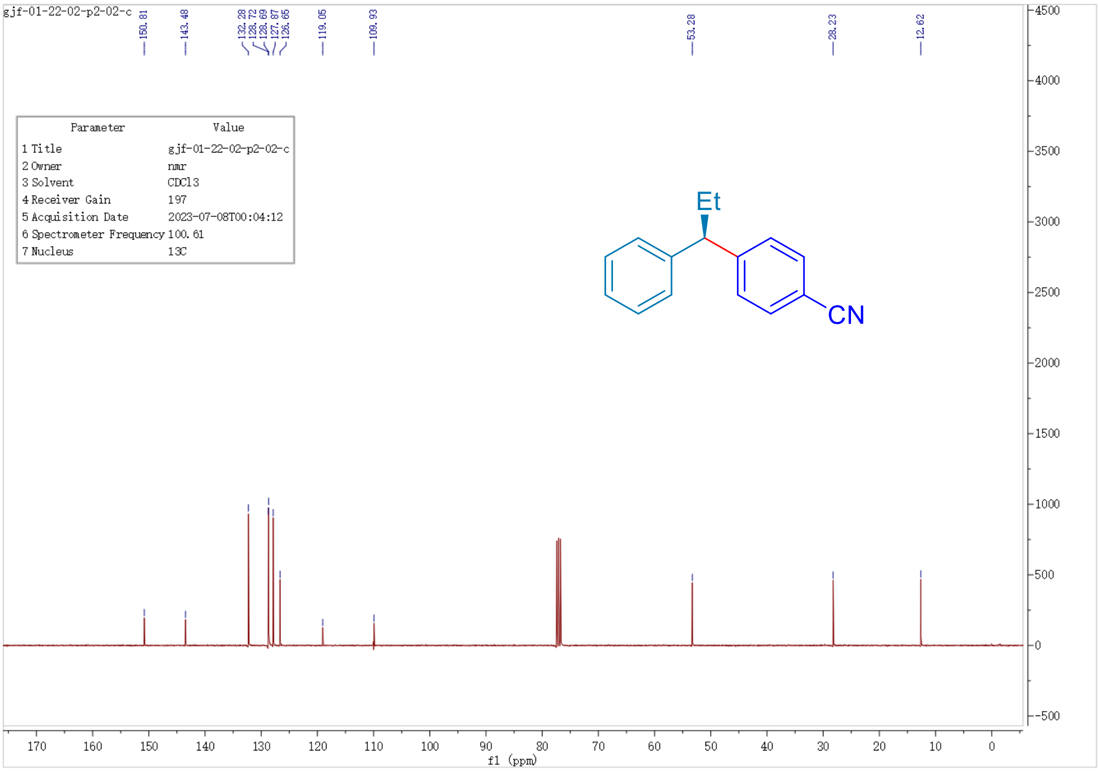

Compound 3e ^{1}H NMR (400 MHz, $CDCl_3$) (4-bromo-1,1'-biphenyl)

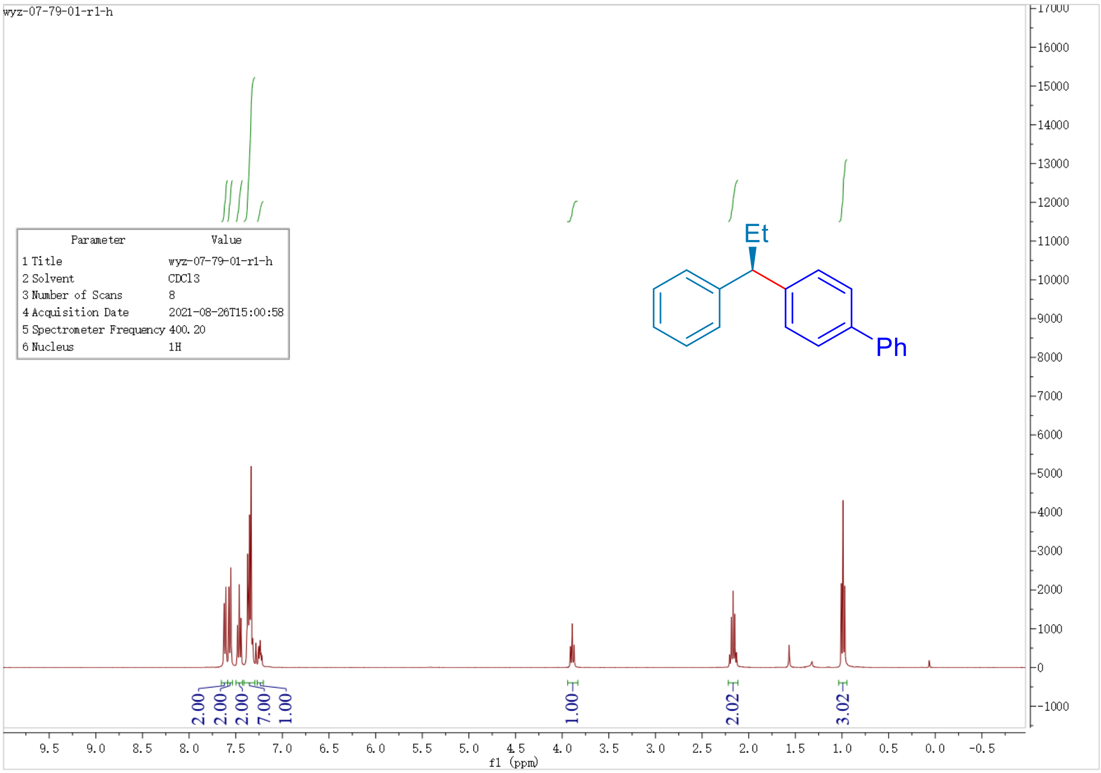

Compound 3e ^{13}C NMR (101 MHz, $CDCl_3$) (4-bromo-1,1'-biphenyl)

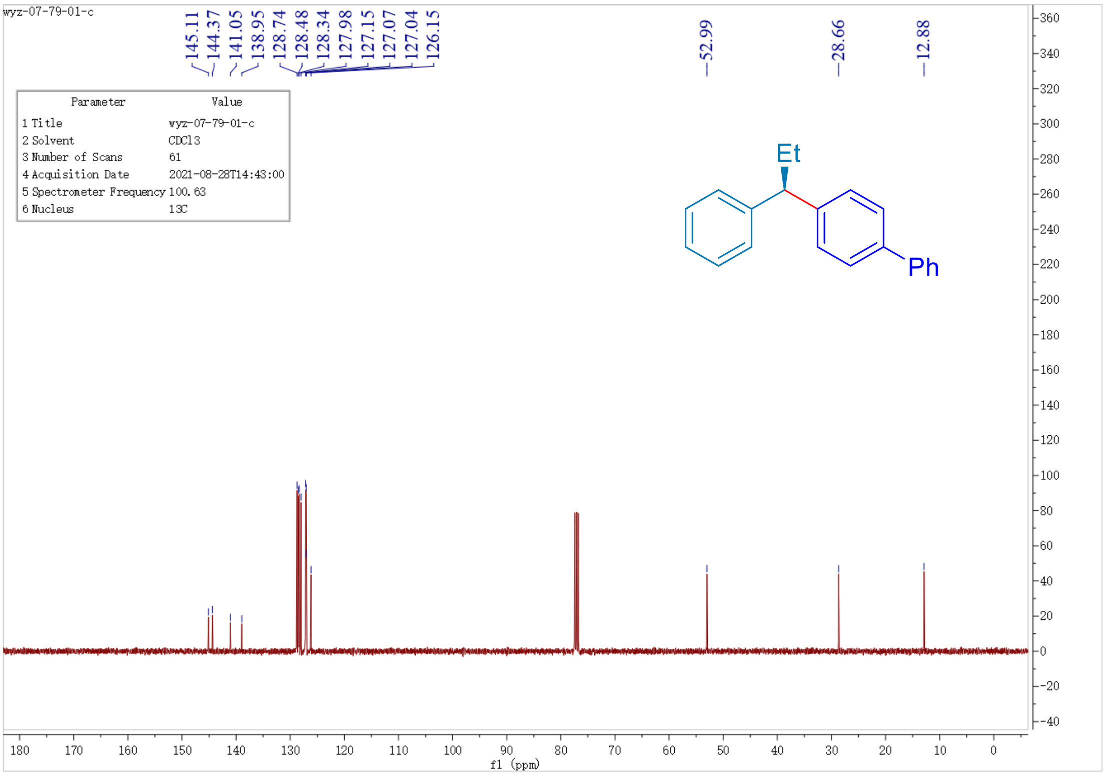

Compound 3e ^{1}H NMR (400 MHz, CDCl₃) (4-chloro-1,1'-biphenyl)

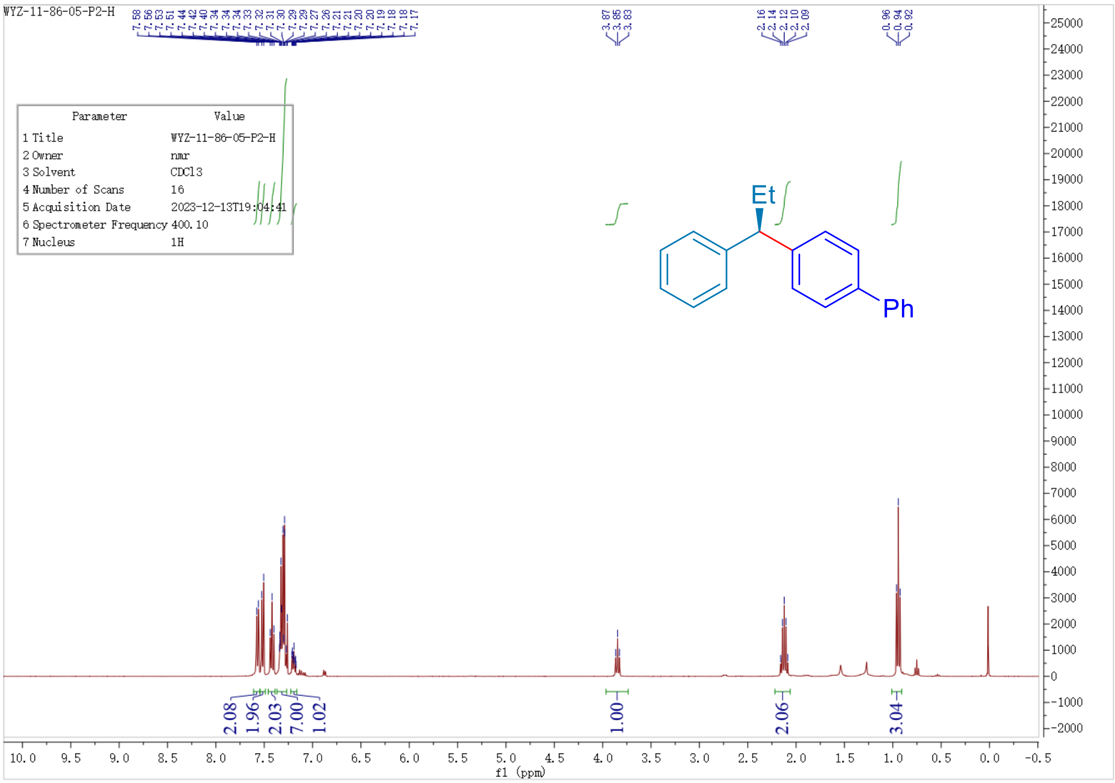

Compound 3e ^{13}C NMR (101 MHz, CDCl₃) (4-chloro-1,1'-biphenyl) [1,1'-biphenyl]-4-yl trifluoromethanesulfonate

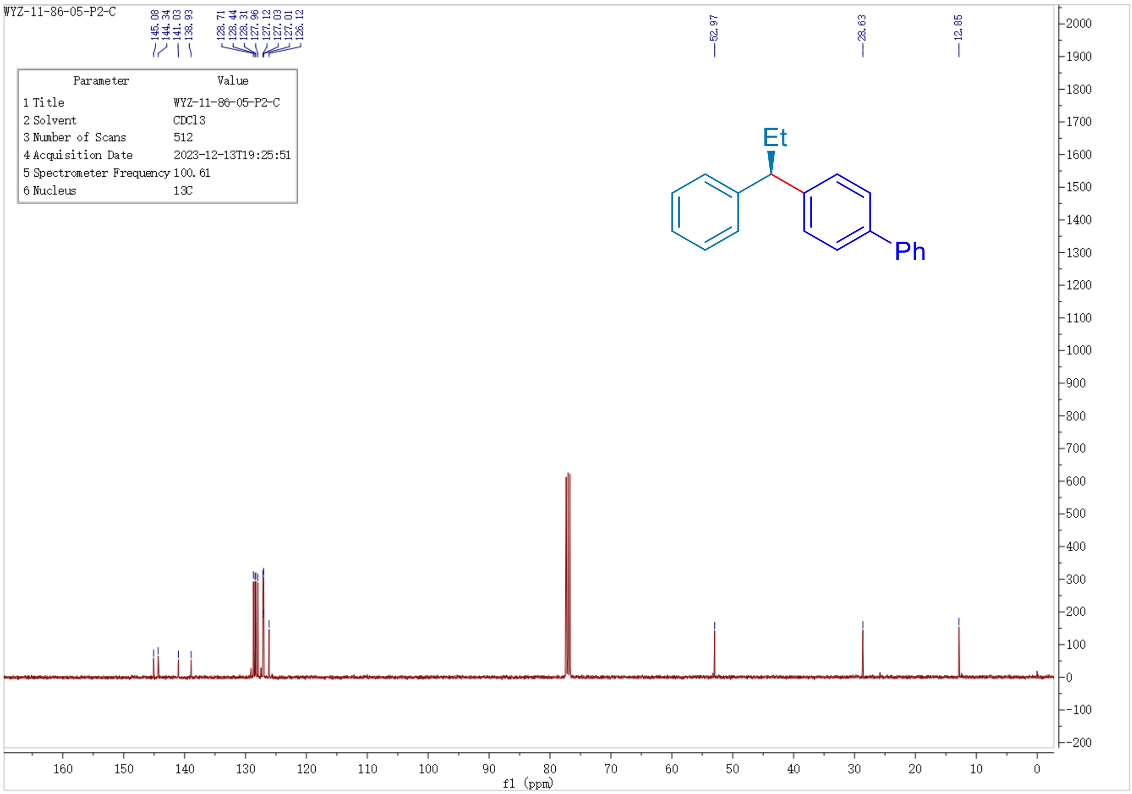

Compound 3e ^{1}H NMR (400 MHz, $CDCl_3$) ([1,1'-biphenyl]-4-yl trifluoromethanesulfonate)

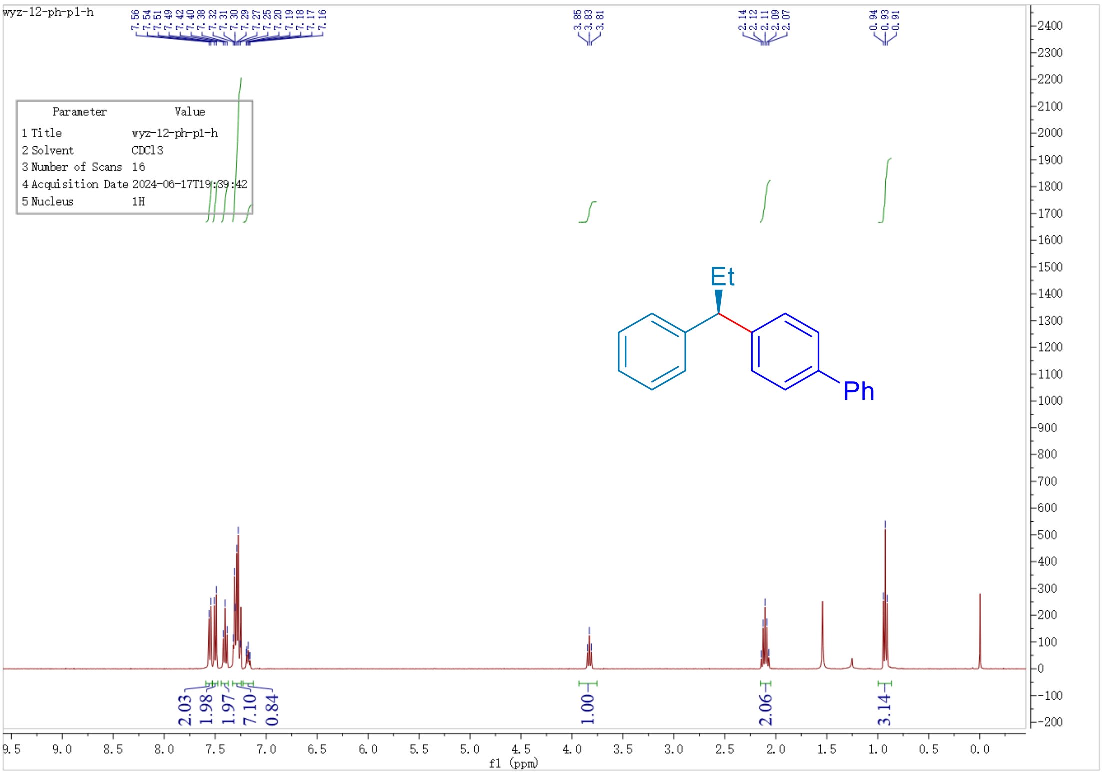

Compound 3e ^{13}C NMR (101 MHz, $CDCl_3$) ([1,1'-biphenyl]-4-yl trifluoromethanesulfonate)

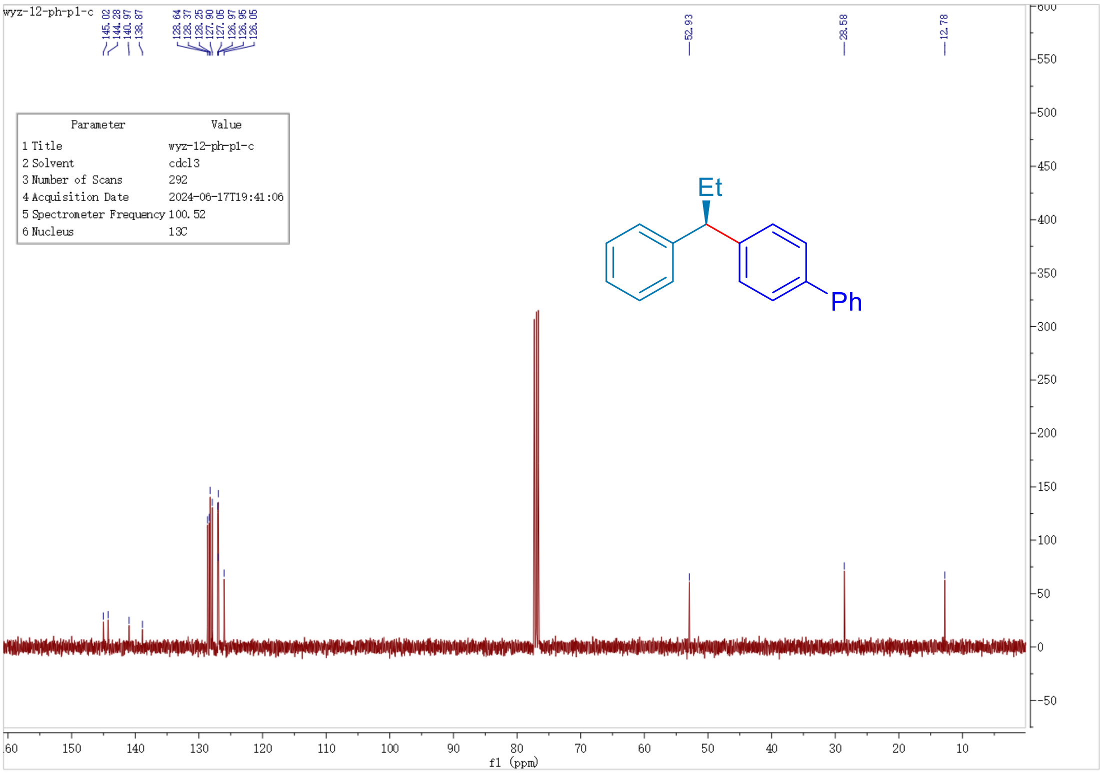

Compound 3f ^{1}H NMR (400 MHz, CDCl$_3$) (methyl 4-bromobenzoate)

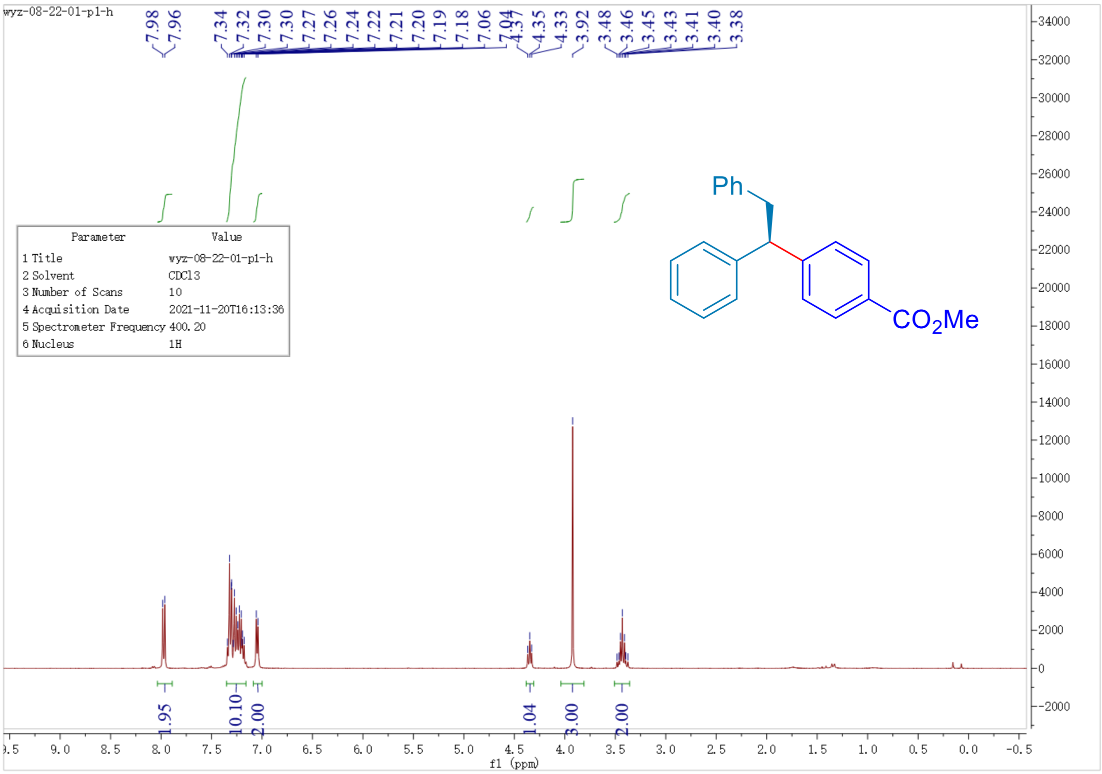

Compound 3f ^{13}C NMR (101 MHz, CDCl$_3$) (methyl 4-bromobenzoate)

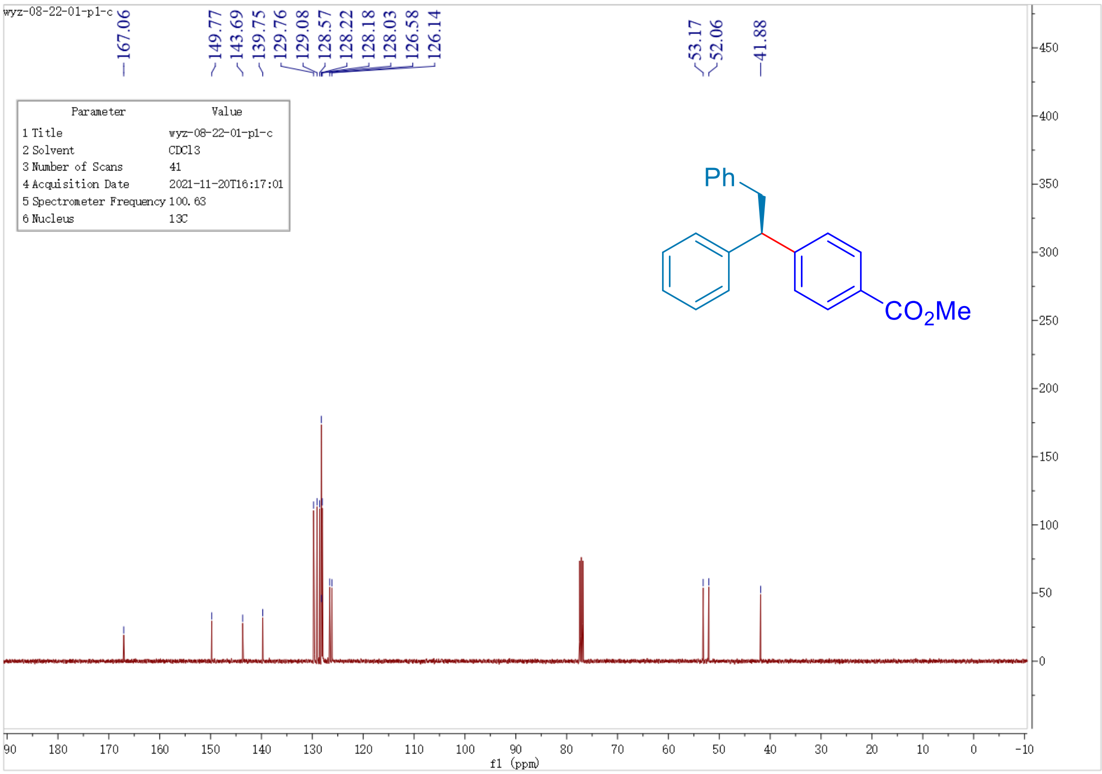

Compound 3f ^{1}H NMR (400 MHz, $CDCl_3$) (methyl 4-chlorobenzoate)

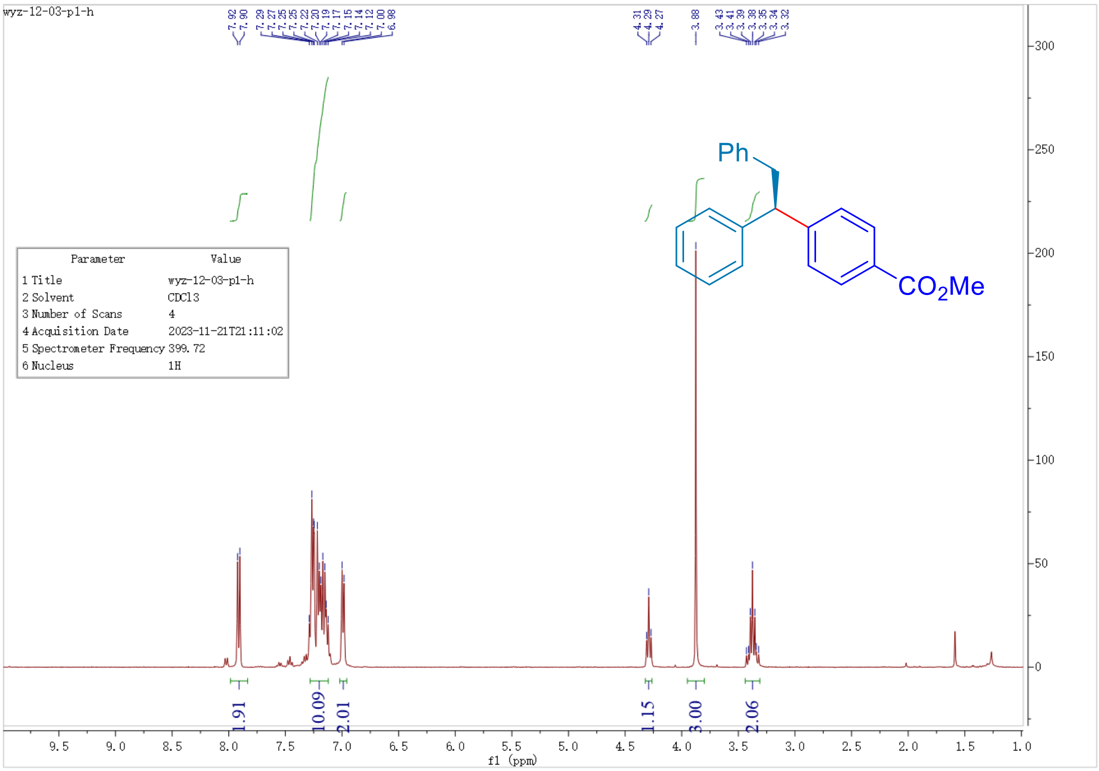

Compound 3f ^{13}C NMR (101 MHz, $CDCl_3$) (methyl 4-chlorobenzoate)

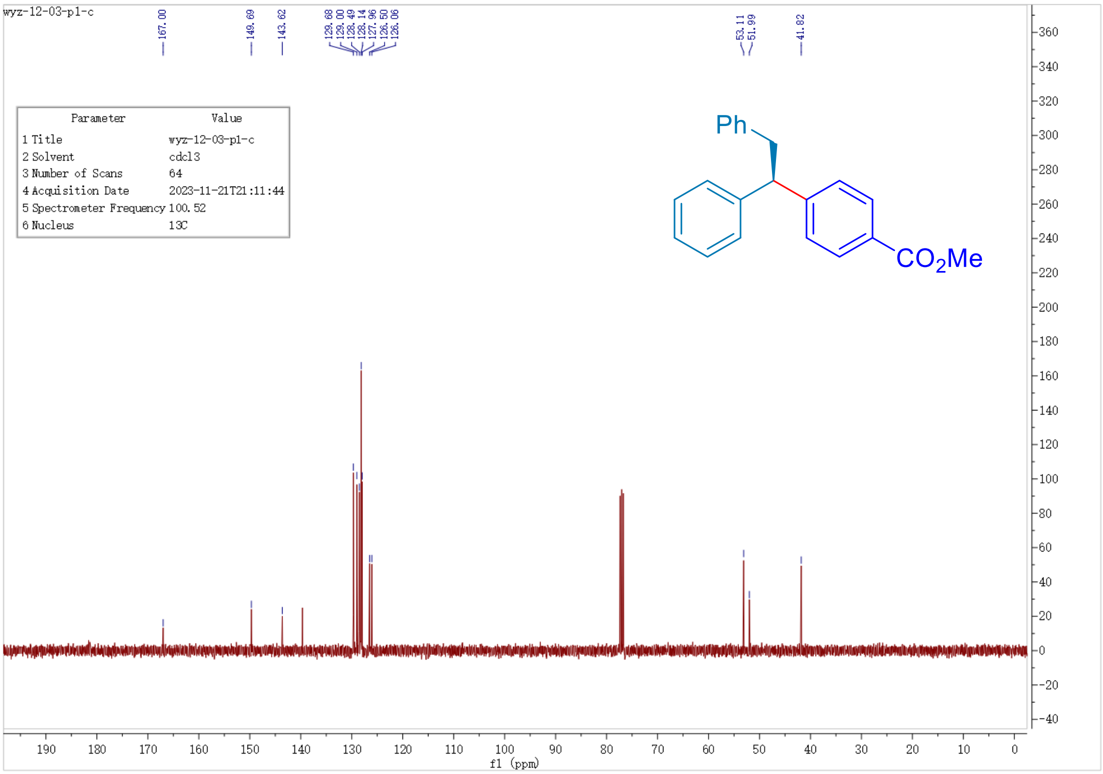

Compound 3g ^{1}H NMR (400 MHz, $CDCl_3$)

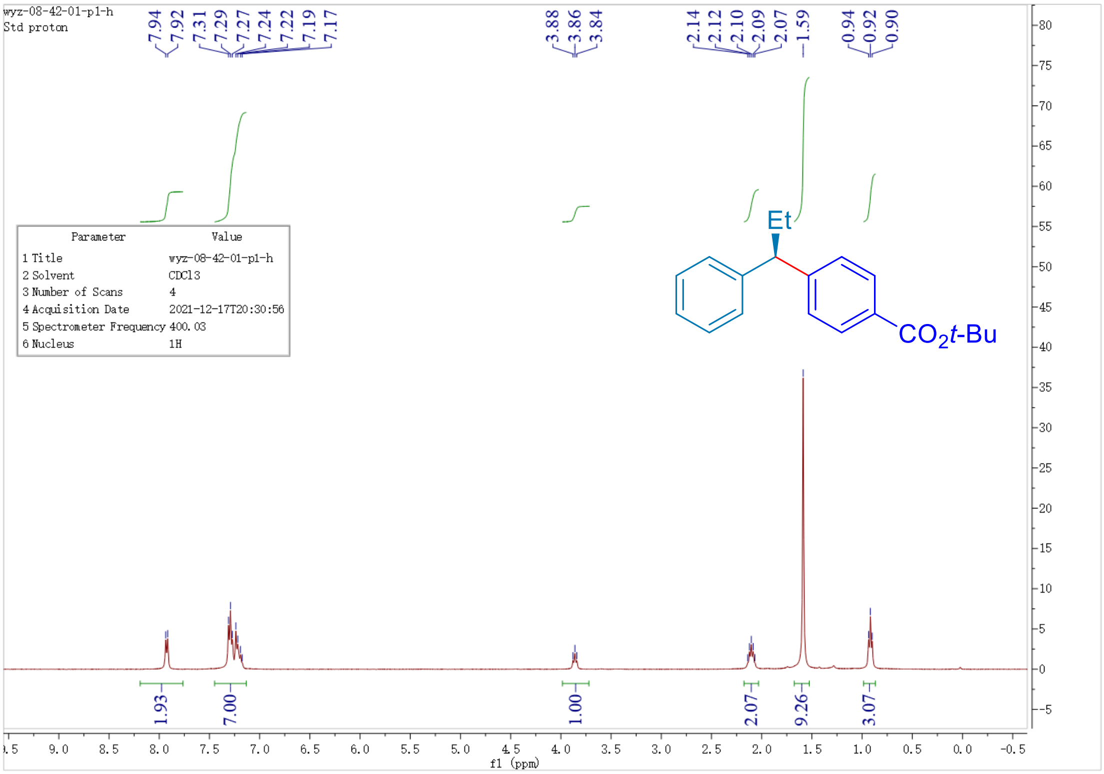

Compound 3g ^{13}C NMR (101 MHz, $CDCl_3$)

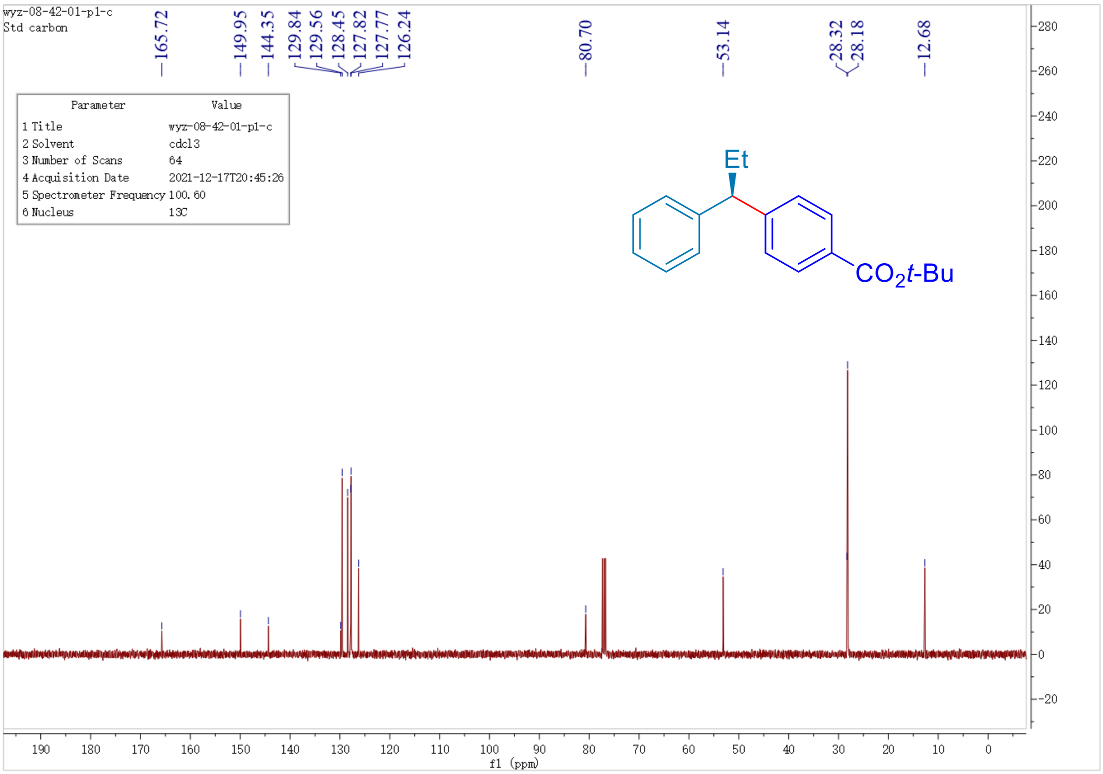

Compound 3h ^{1}H NMR (400 MHz, $CDCl_3$)

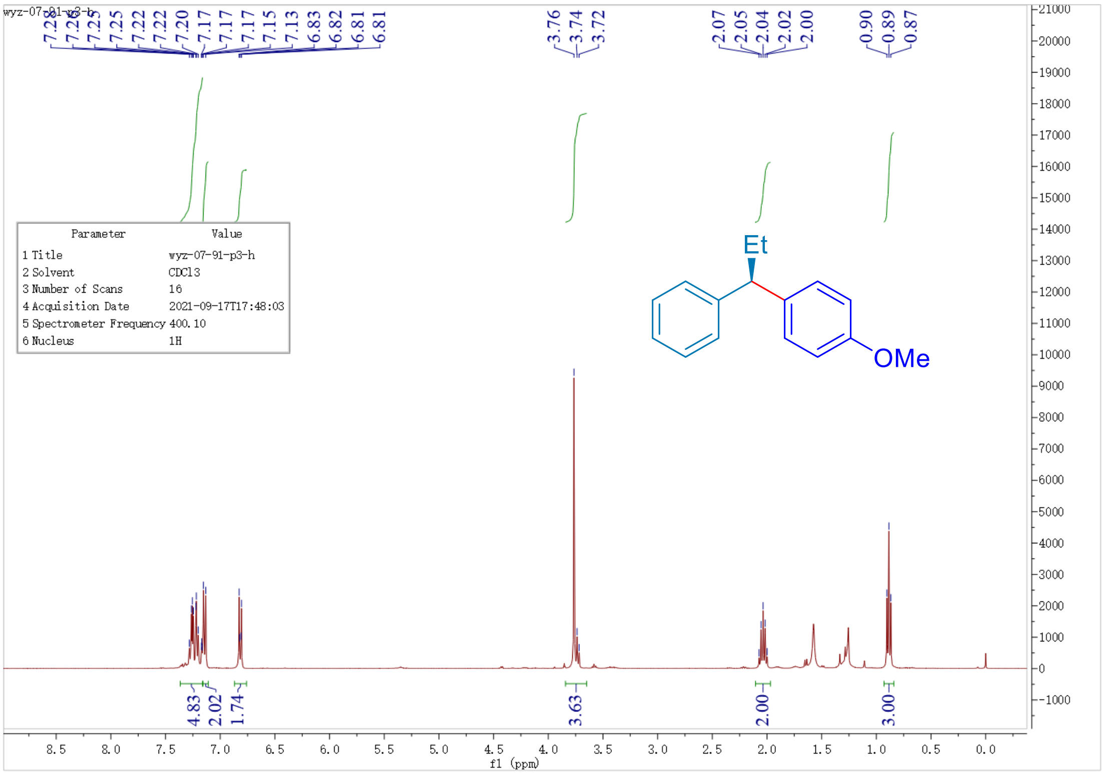

Compound 3h ^{13}C NMR (101 MHz, $CDCl_3$)

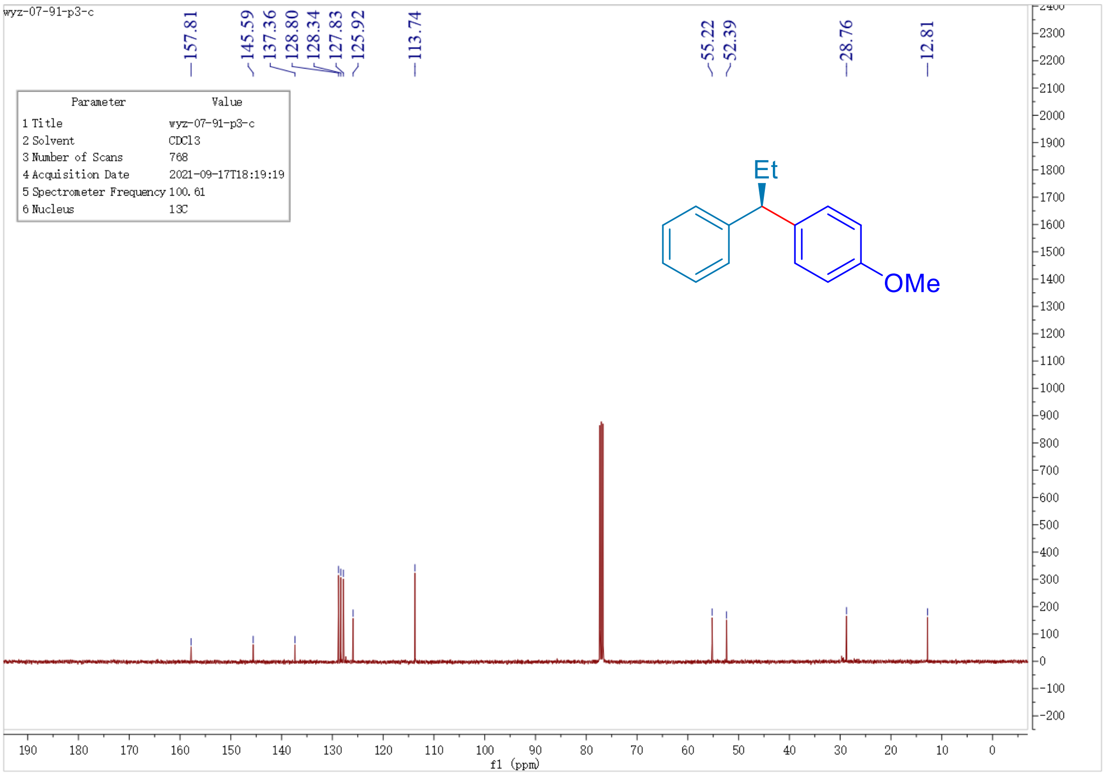

Compound 3i ^{1}H NMR (400 MHz, $CDCl_3$)

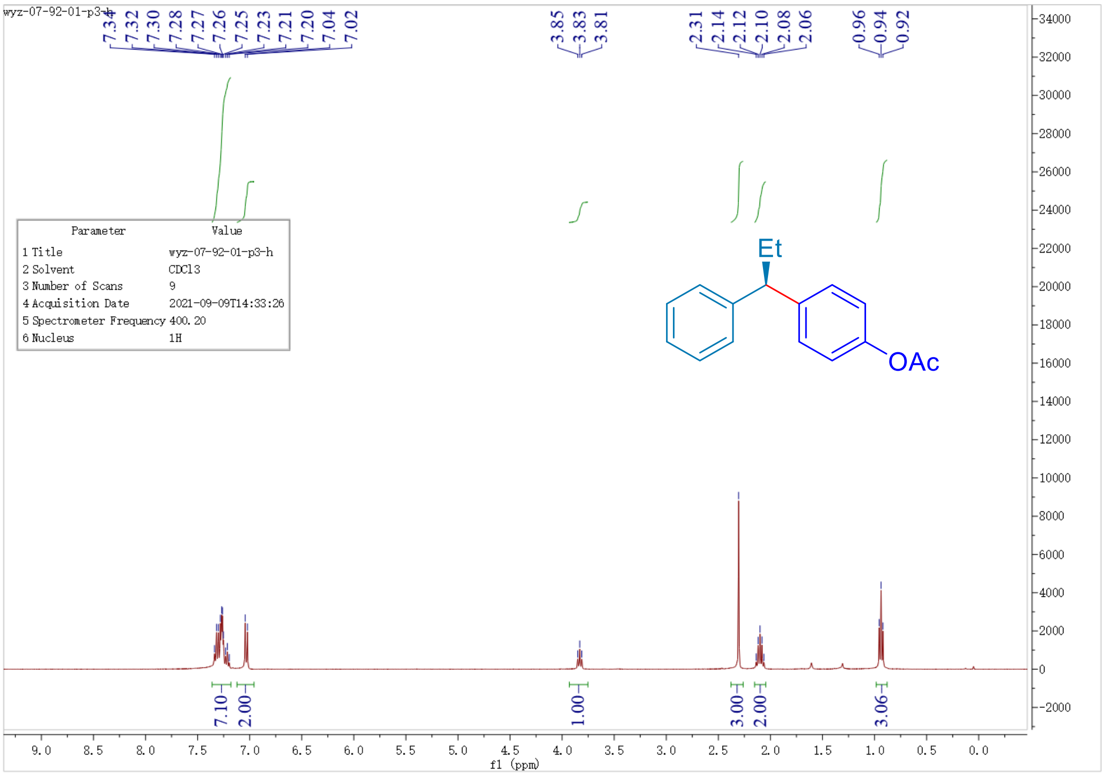

Compound 3i ^{13}C NMR (101 MHz, $CDCl_3$)

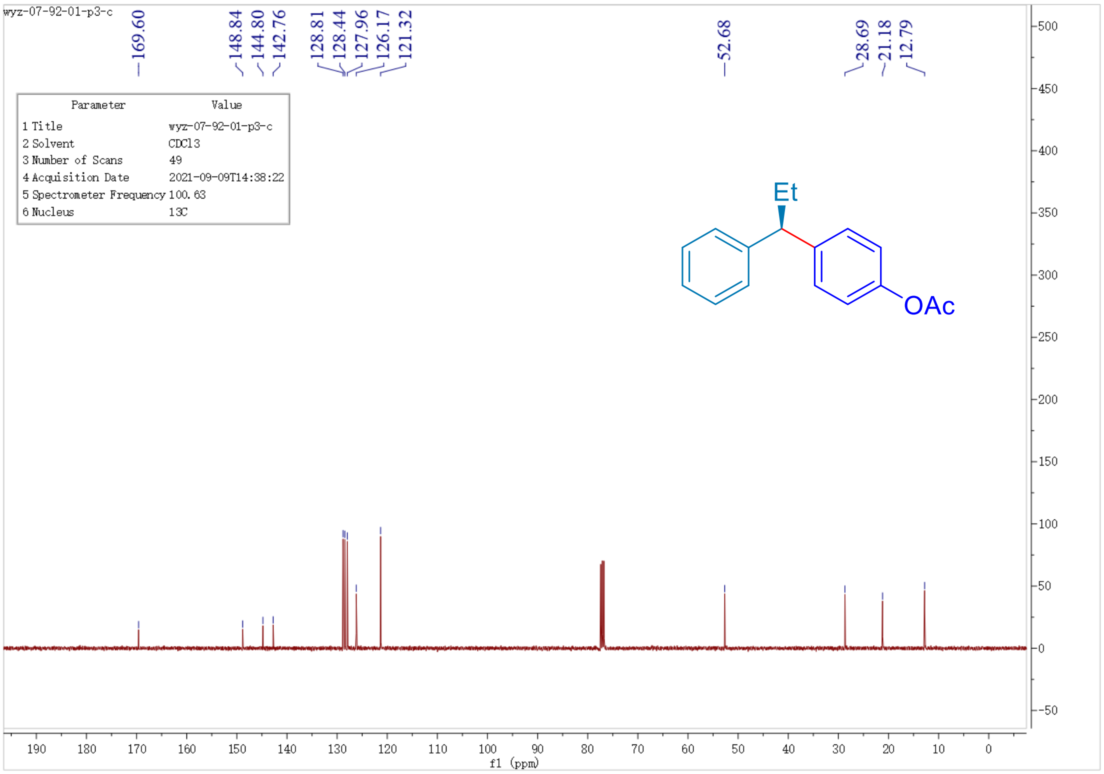

Compound 3j ^{1}H NMR (400 MHz, $CDCl_3$)

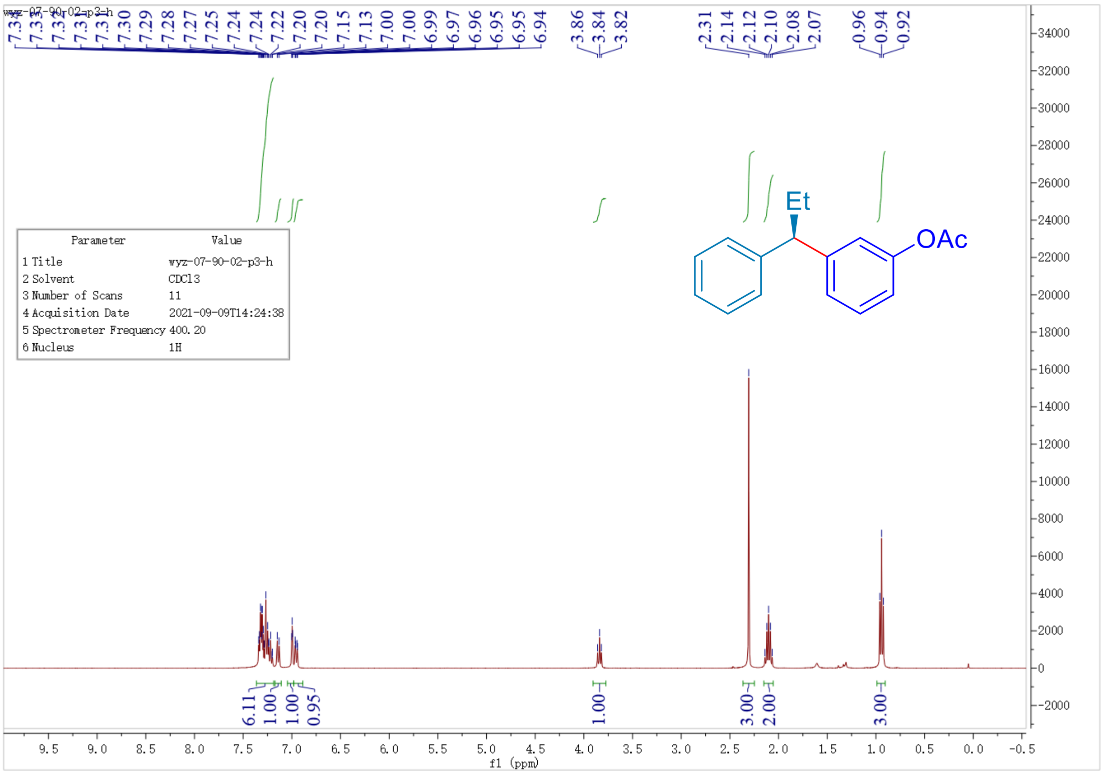

Compound 3j ^{13}C NMR (101 MHz, $CDCl_3$)

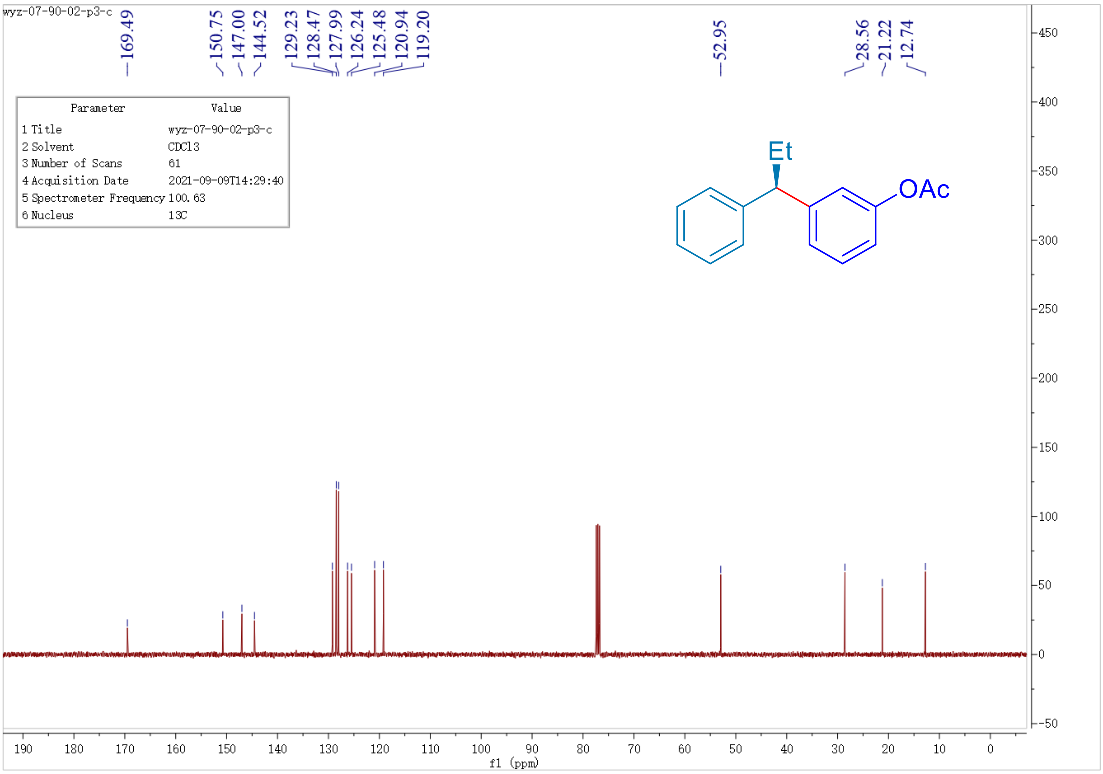

Compound 3k ^{1}H NMR (400 MHz, $CDCl_3$)

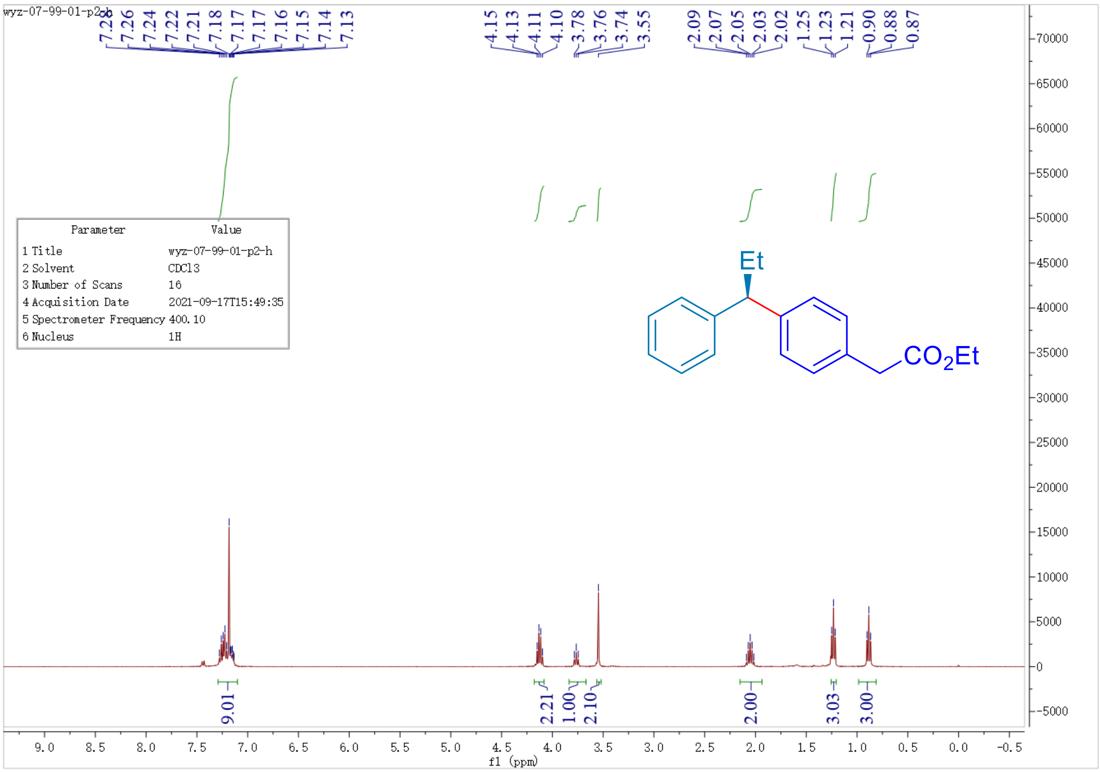

Compound 3k ^{13}C NMR (101 MHz, $CDCl_3$)

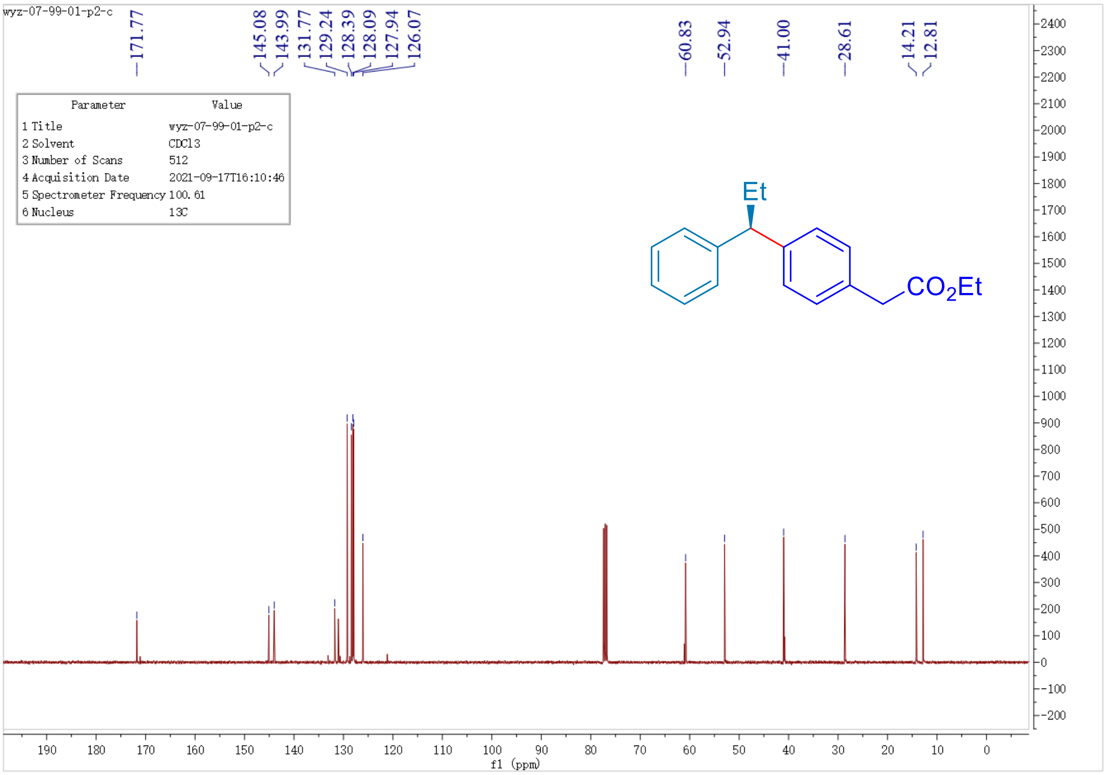

Compound 3l ^{1}H NMR (400 MHz, $CDCl_3$)

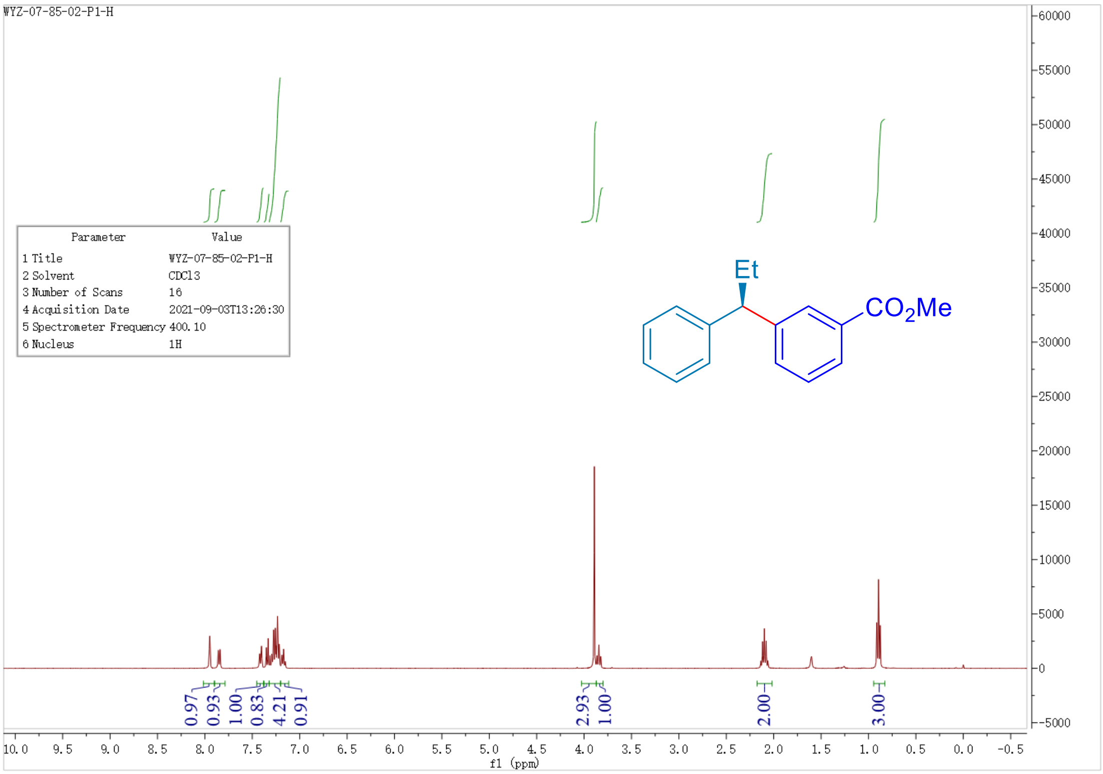

Compound 3l ^{13}C NMR (101 MHz, $CDCl_3$)

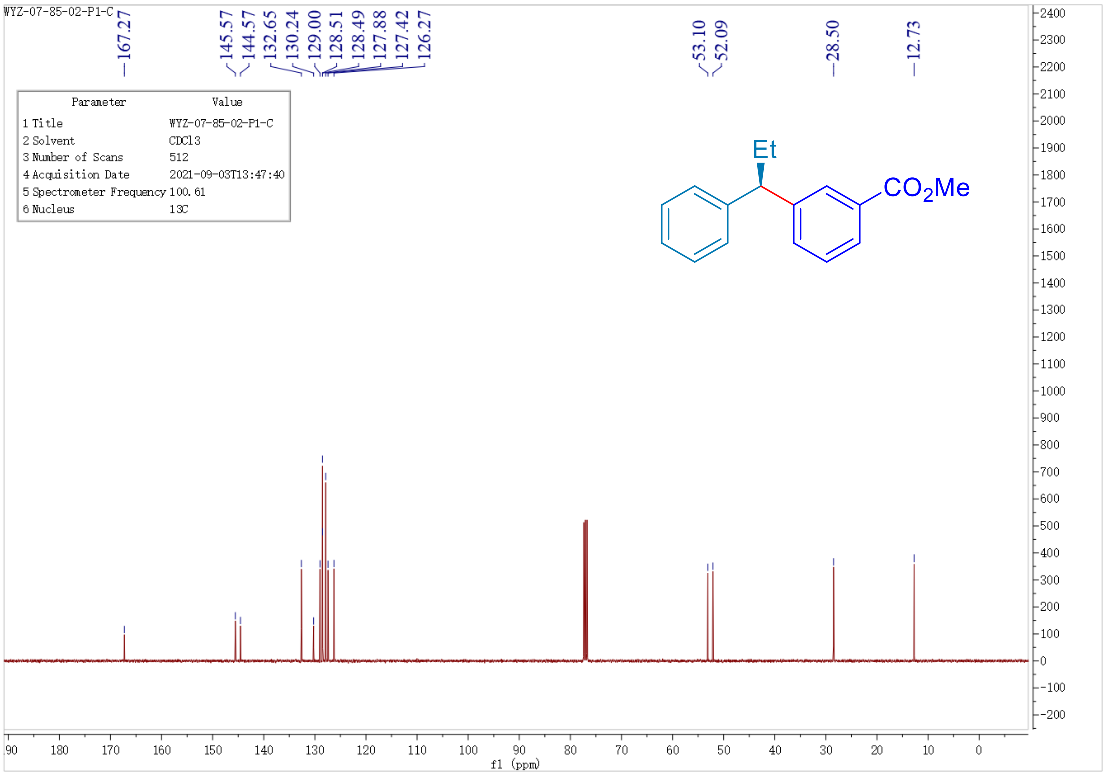

Compound 3m ^{1}H NMR (400 MHz, $CDCl_3$)

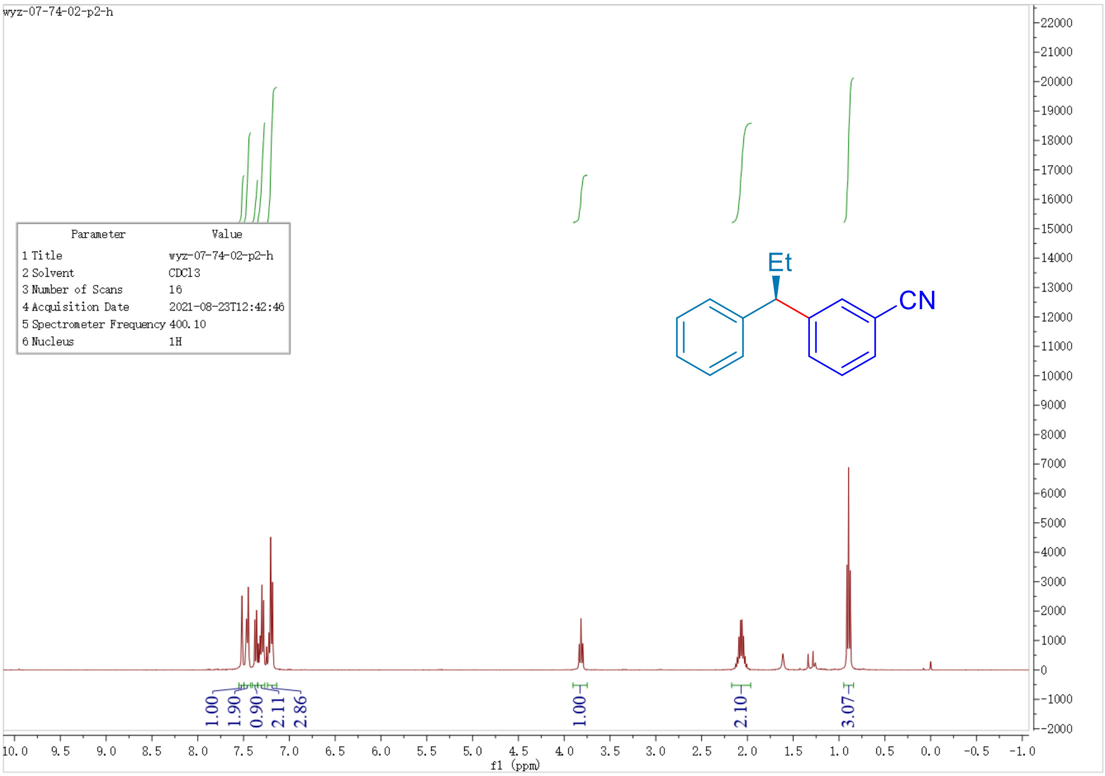

Compound 3m ^{13}C NMR (101 MHz, $CDCl_3$)

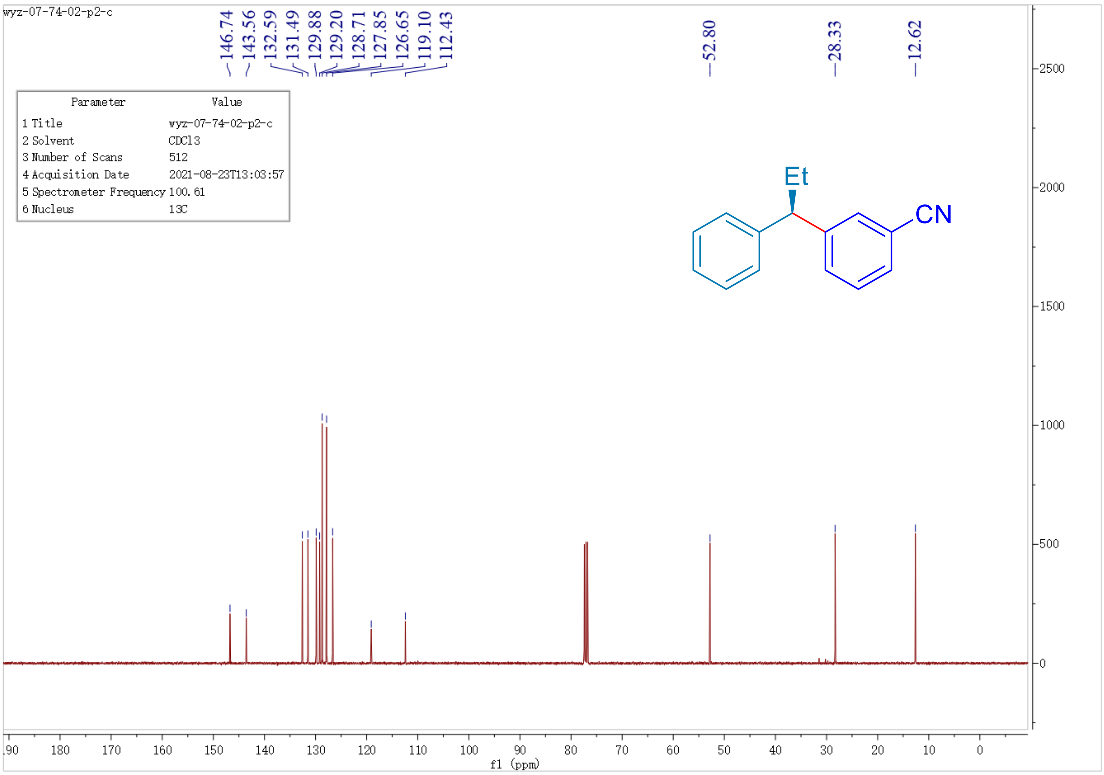

Compound 3n ¹H NMR (400 MHz, $CDCl_3$)

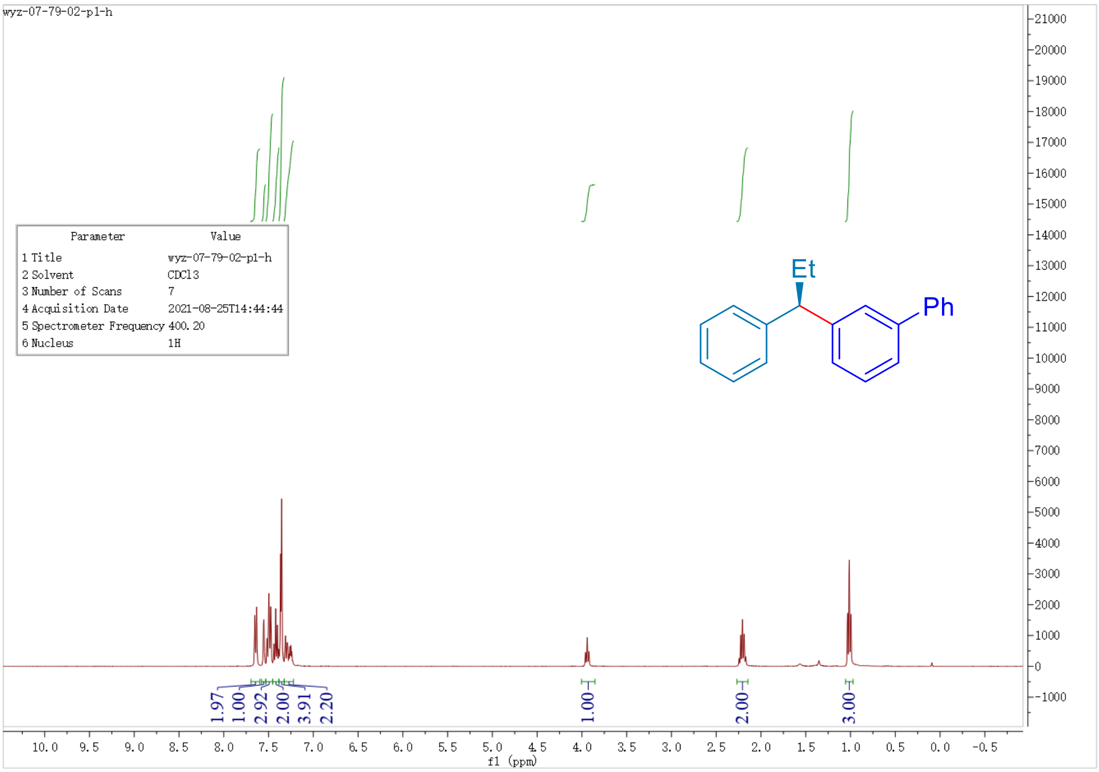

Compound 3n ¹³C NMR (101 MHz, $CDCl_3$)

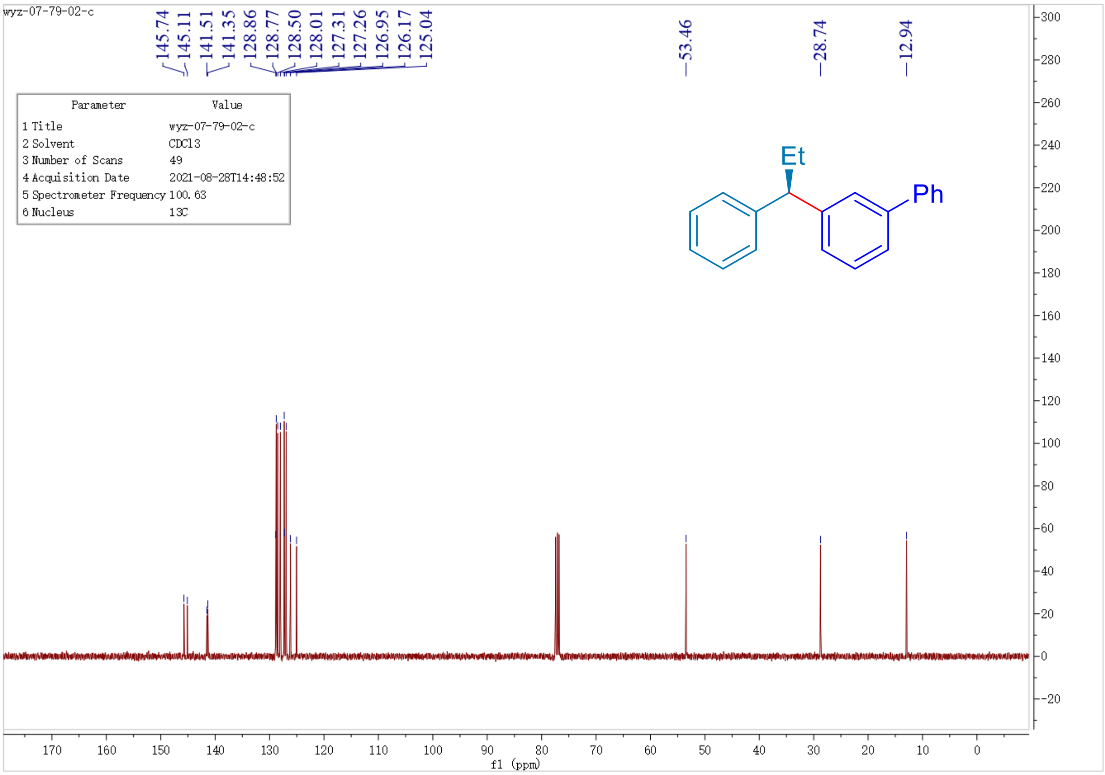

Compound 3o ^{1}H NMR (400 MHz, $CDCl_3$)

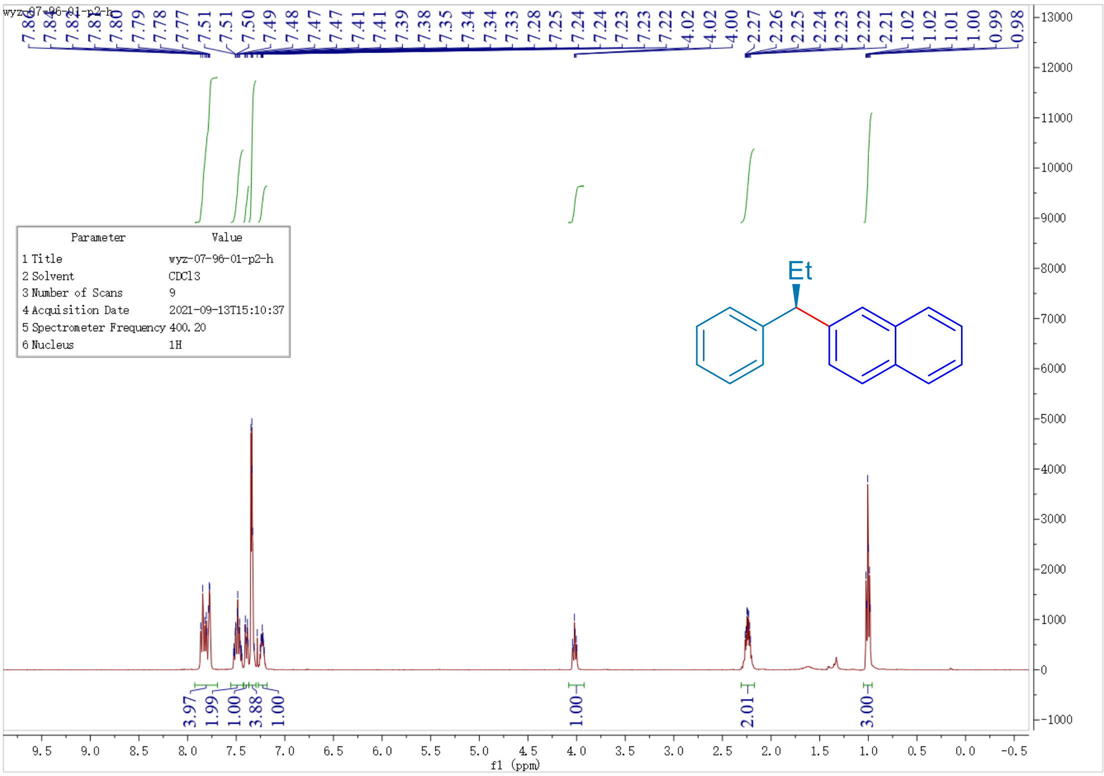

Compound 3o ^{13}C NMR (101 MHz, $CDCl_3$)

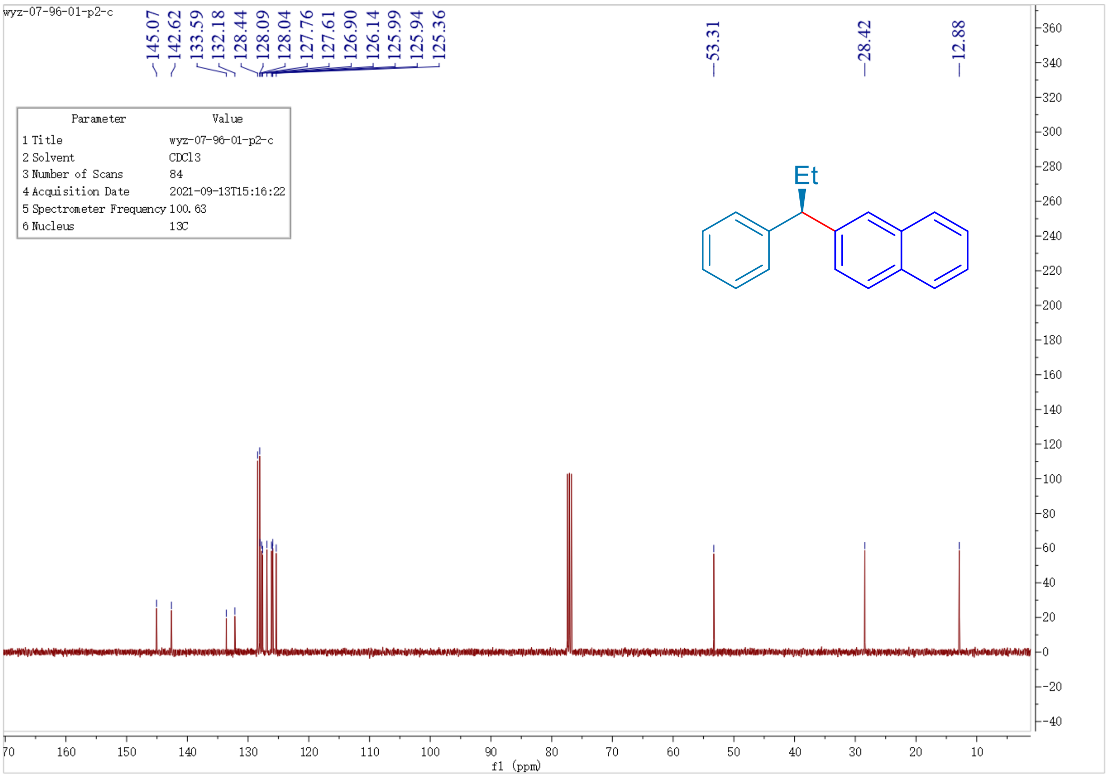

Compound 3p ^{1}H NMR (400 MHz, CDCl$_3$)

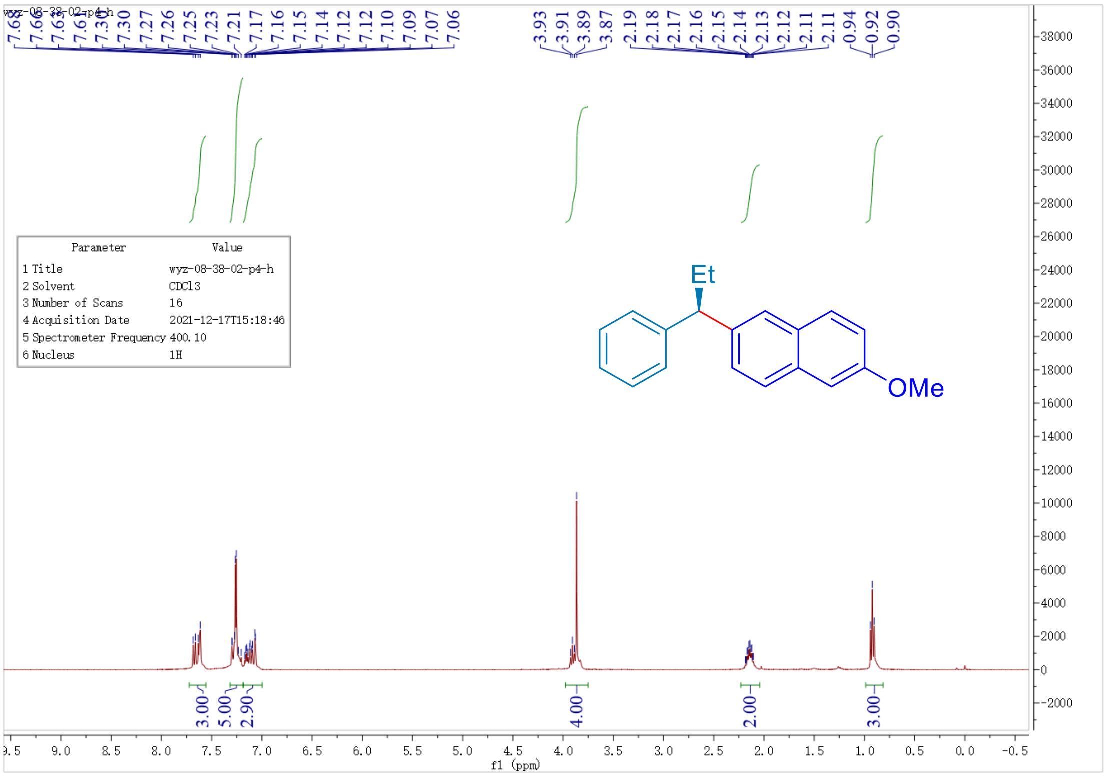

Compound 3p ^{13}C NMR (101 MHz, CDCl$_3$)

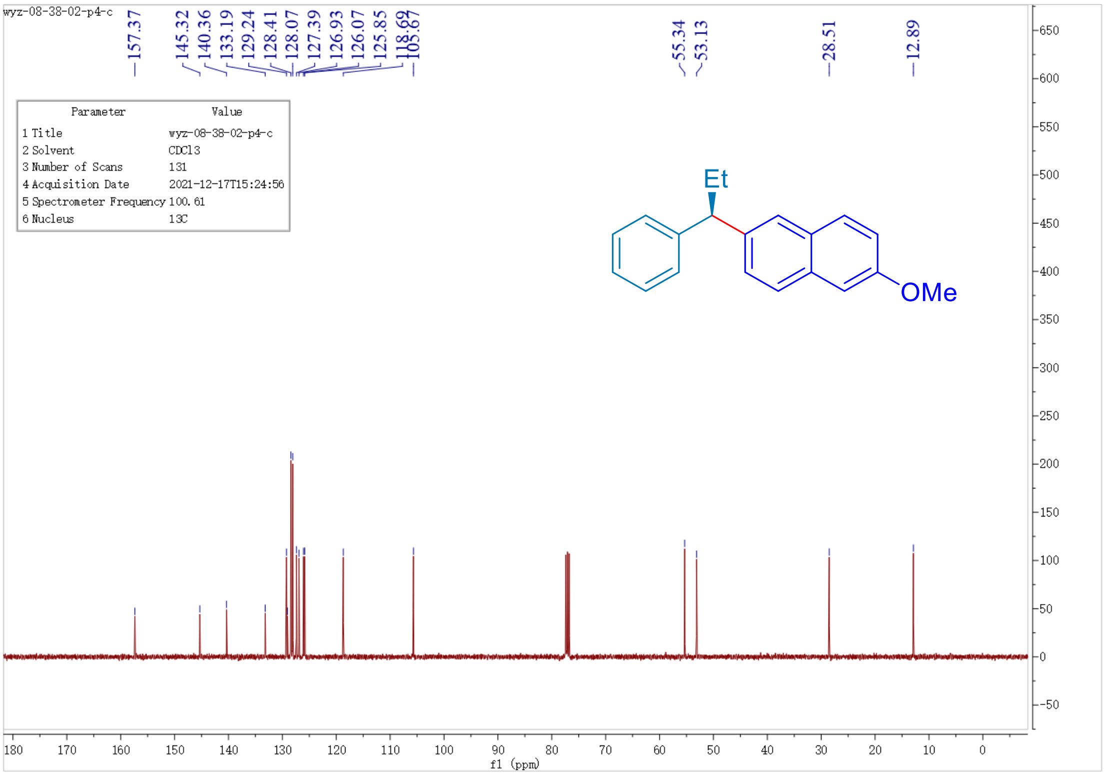

Compound 3q ^{1}H NMR (400 MHz, $CDCl_3$)

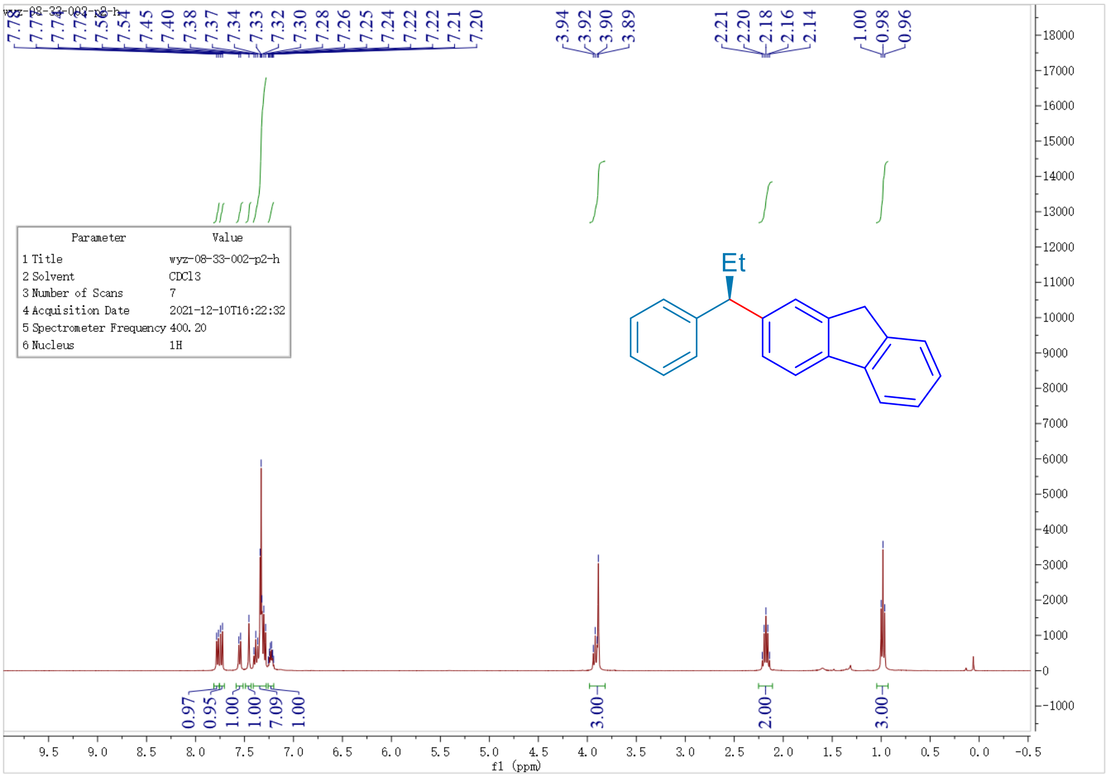

Compound 3q ^{13}C NMR (101 MHz, $CDCl_3$)

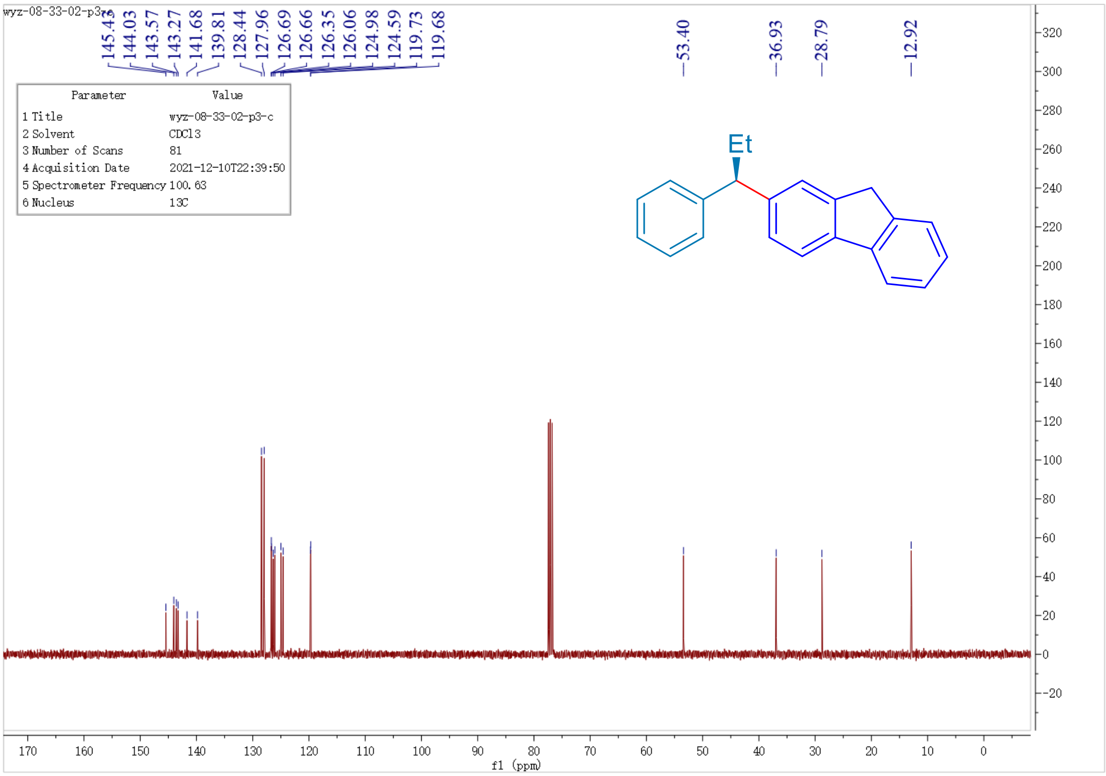

Compound 3r ^{1}H NMR (400 MHz, $CDCl_3$)

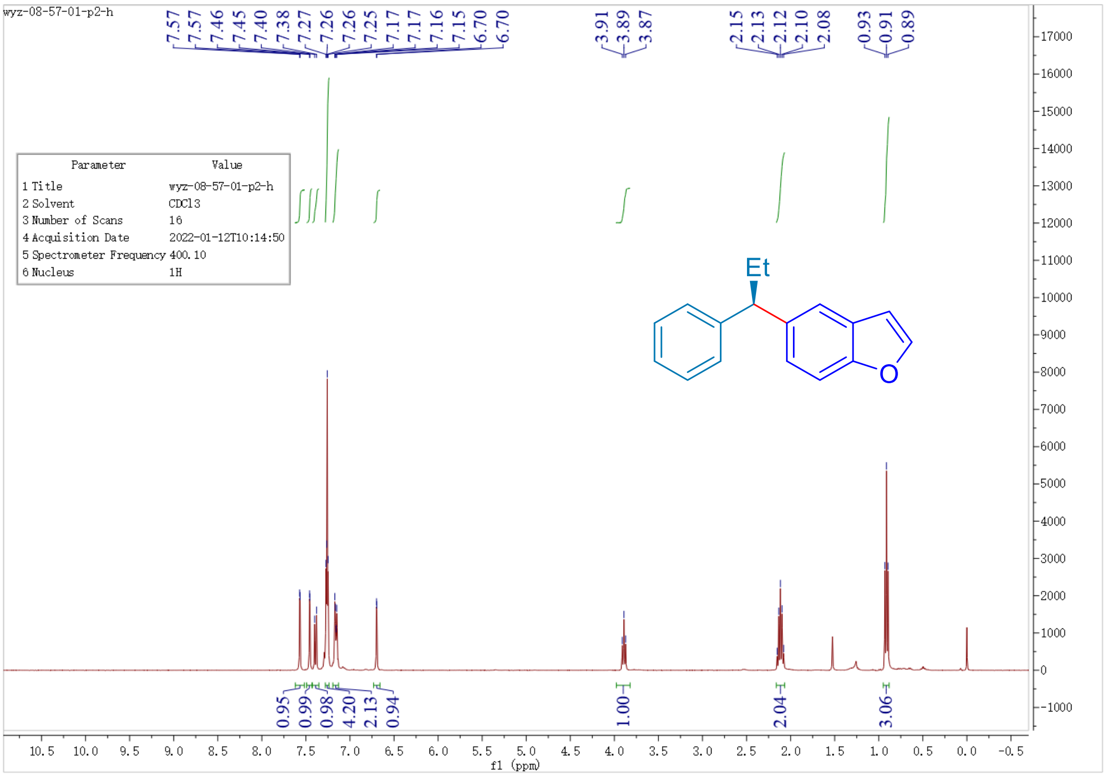

Compound 3r ^{13}C NMR (101 MHz, $CDCl_3$)

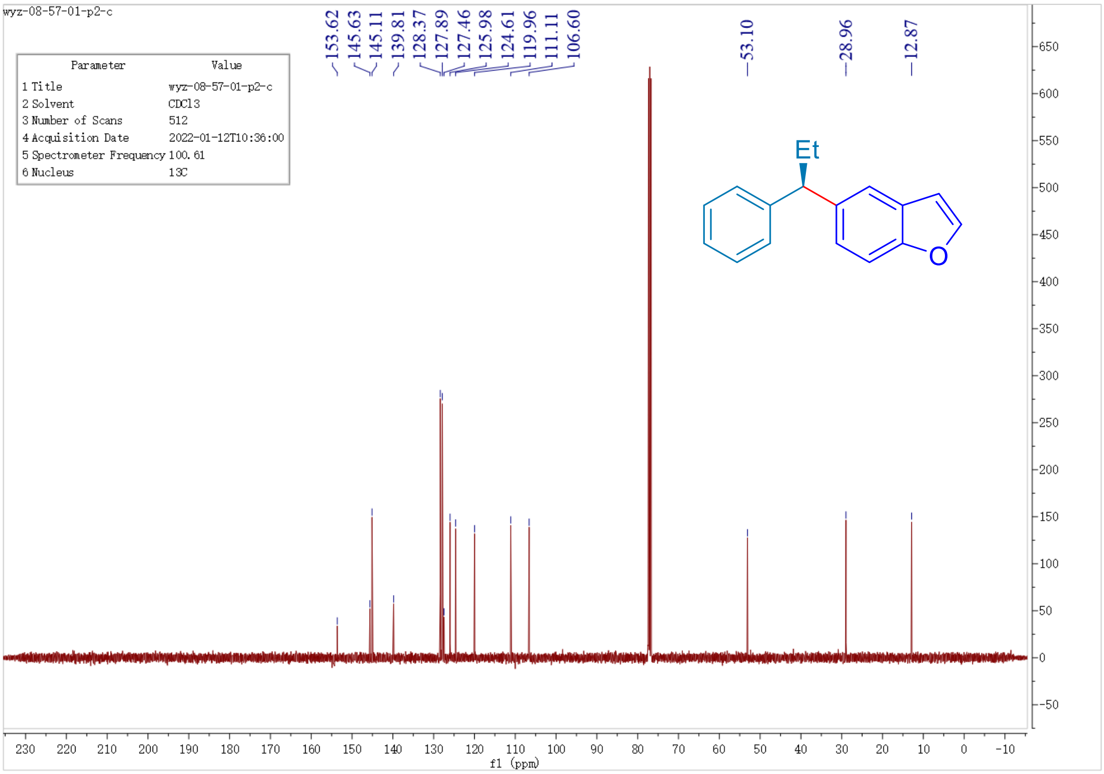

Compound 3s ^{1}H NMR (400 MHz, CDCl$_3$)

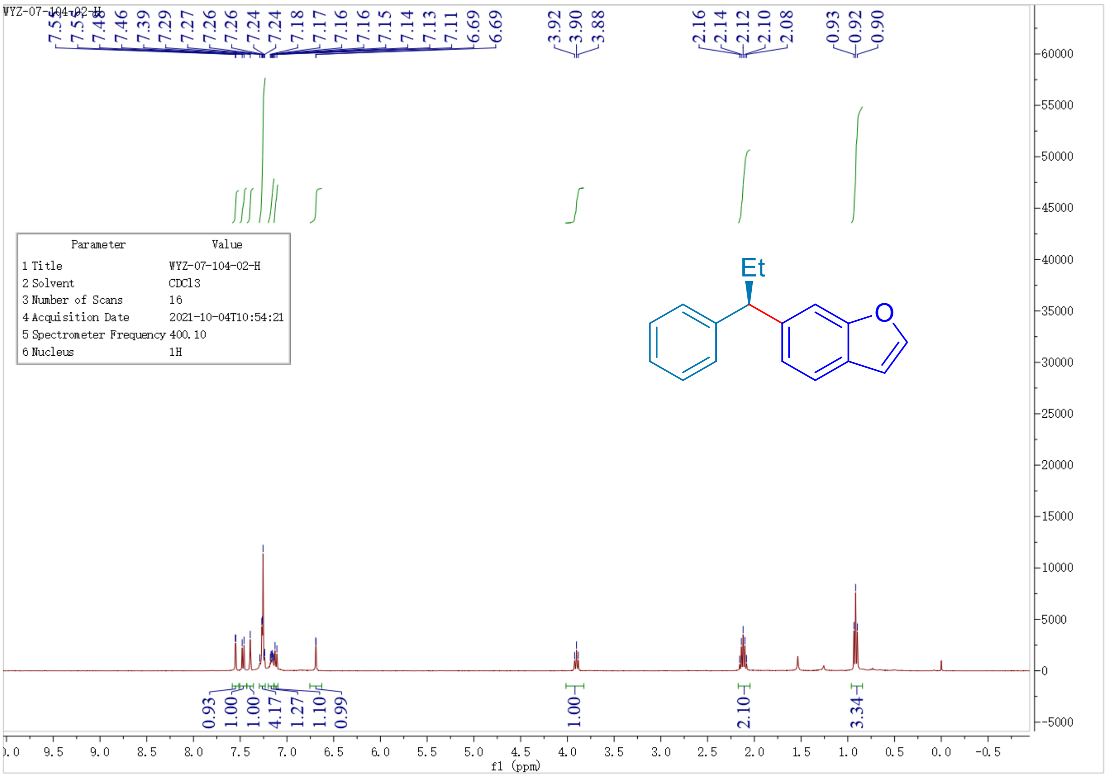

Compound 3s ^{13}C NMR (101 MHz, CDCl$_3$)

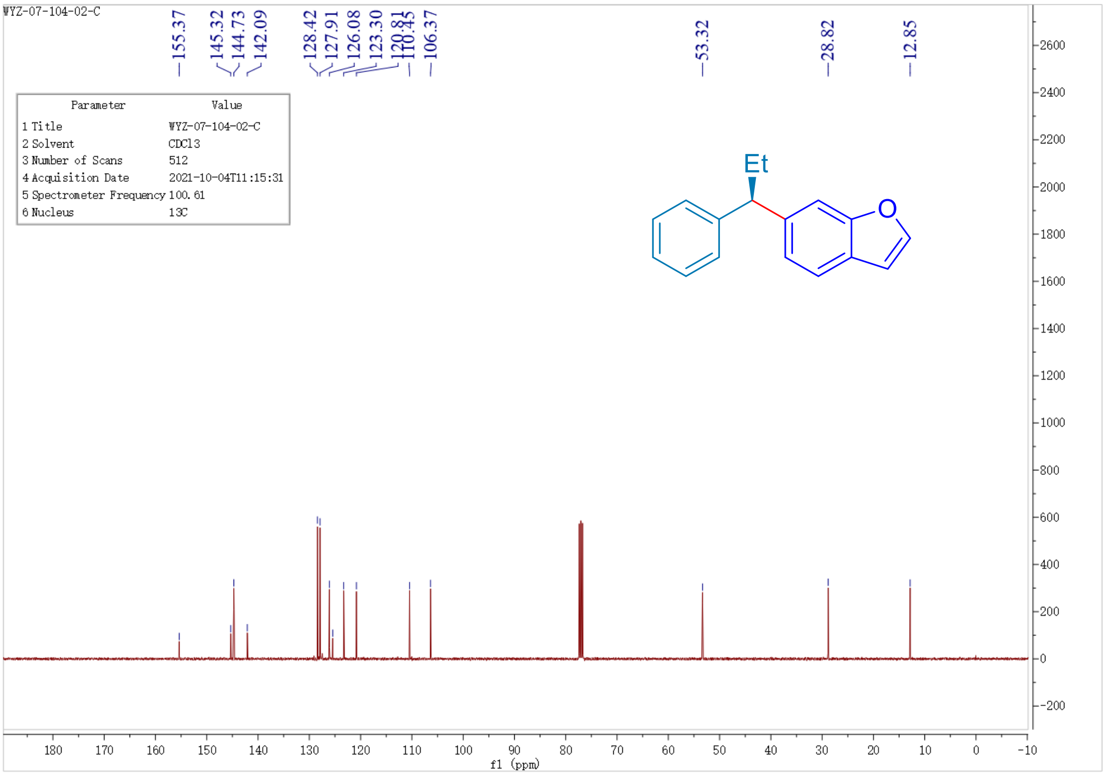

Compound 3t ^{1}H NMR (400 MHz, $CDCl_3$)

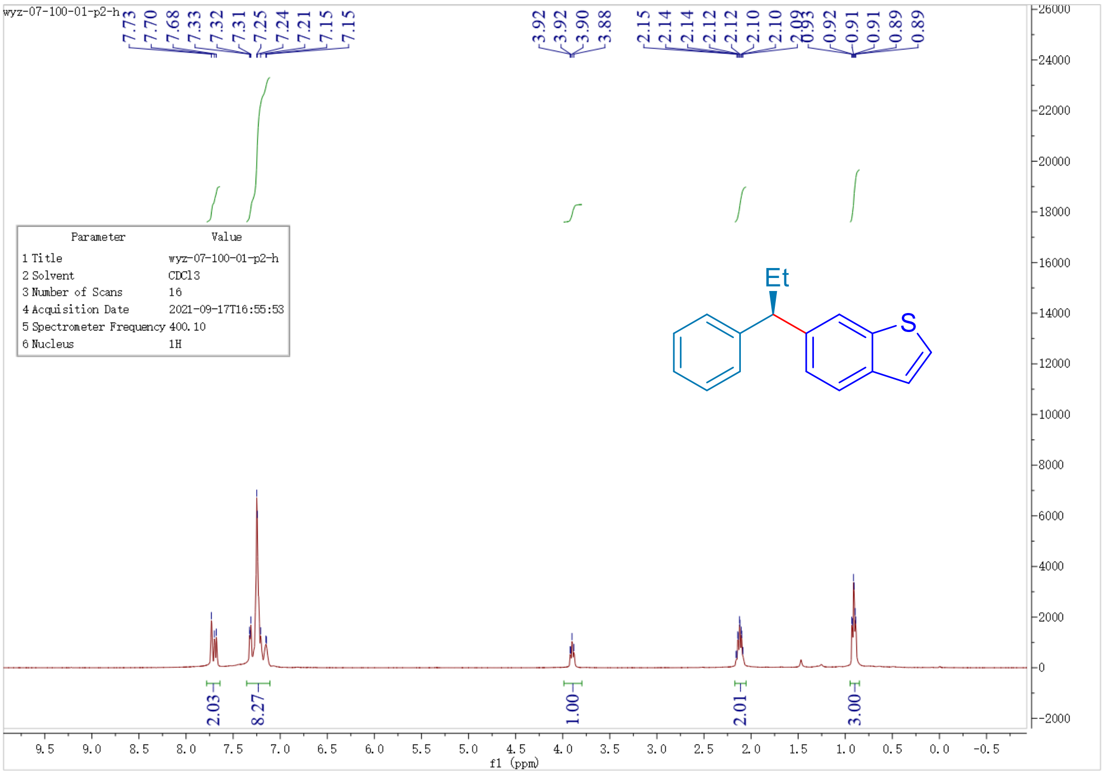

Compound 3t ^{13}C NMR (101 MHz, $CDCl_3$)

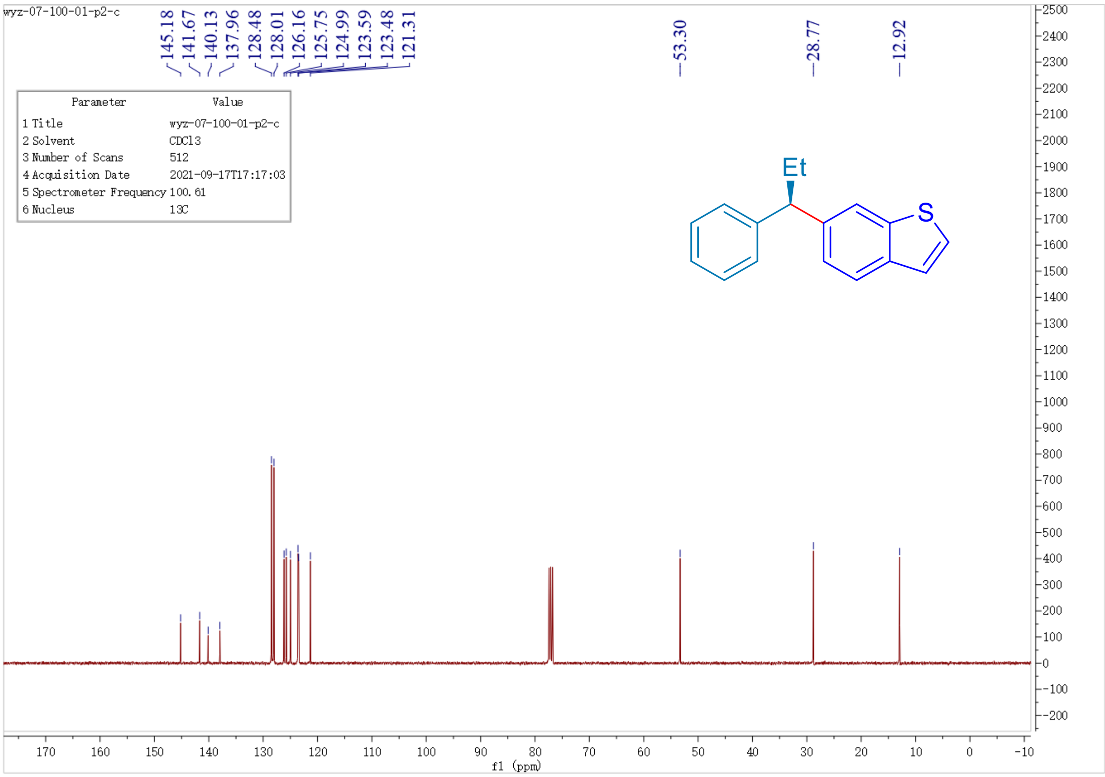

Compound 3u ^{1}H NMR (400 MHz, $CDCl_3$)

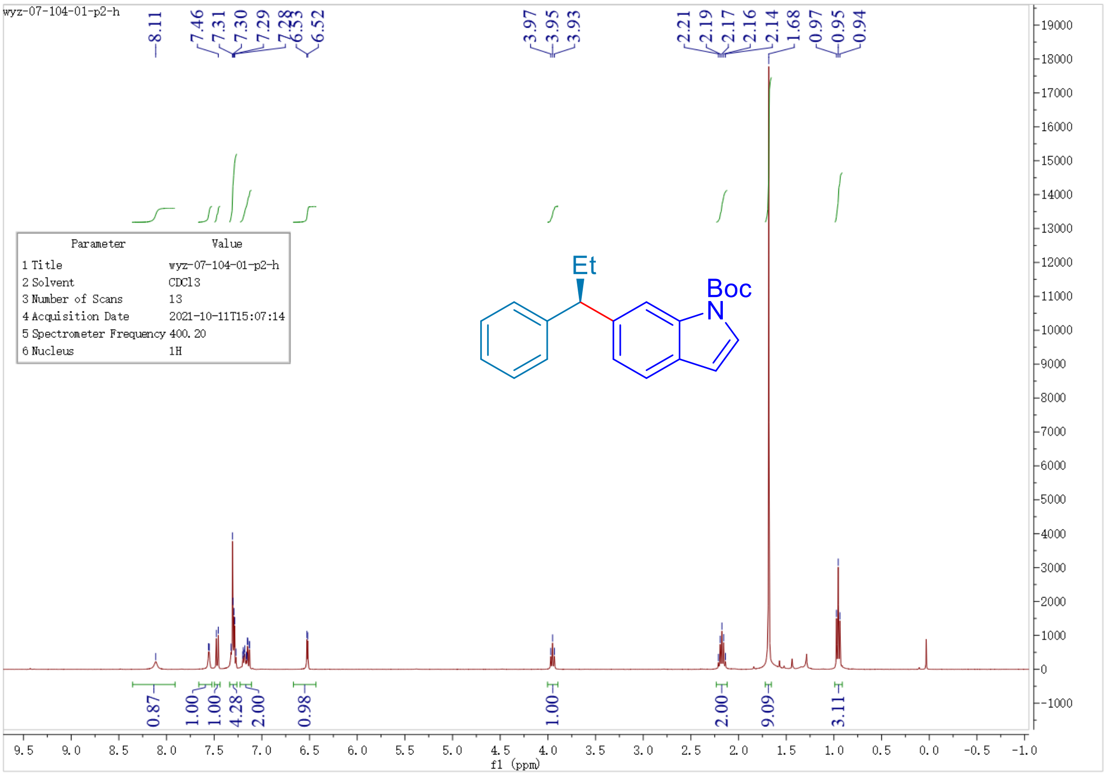

Compound 3u ^{13}C NMR (101 MHz, $CDCl_3$)

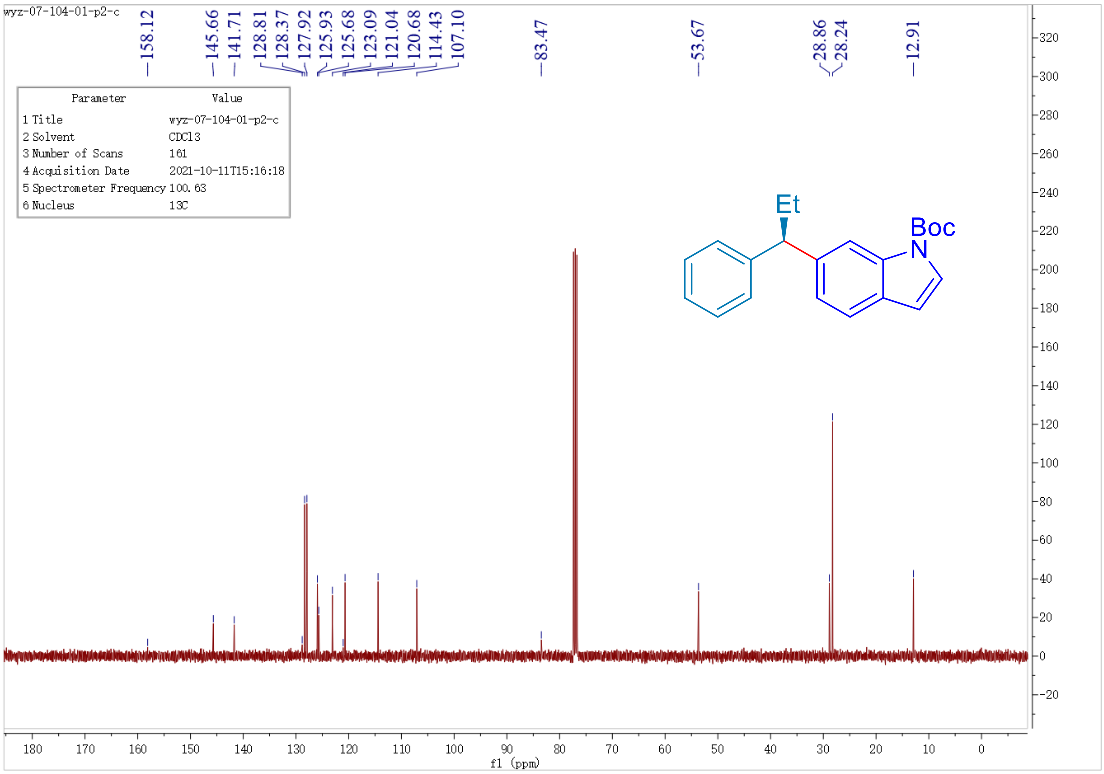

Compound 3v ^{1}H NMR (400 MHz, $CDCl_3$)

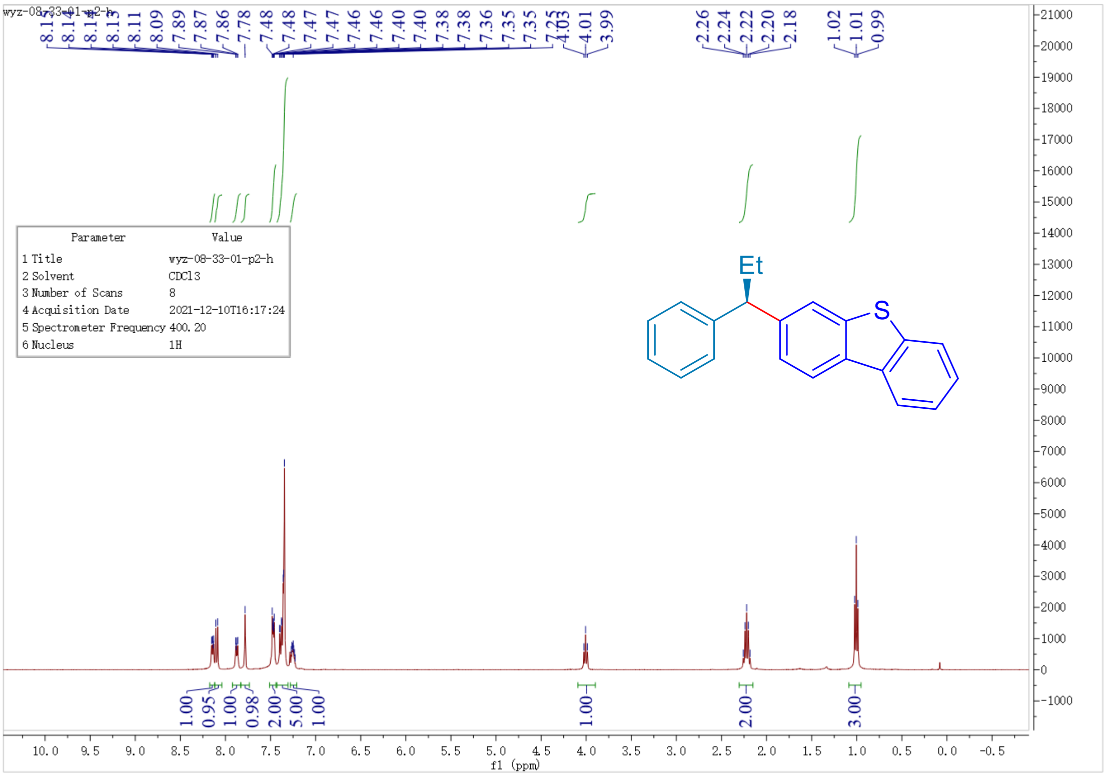

Compound 3v ^{13}C NMR (101 MHz, $CDCl_3$)

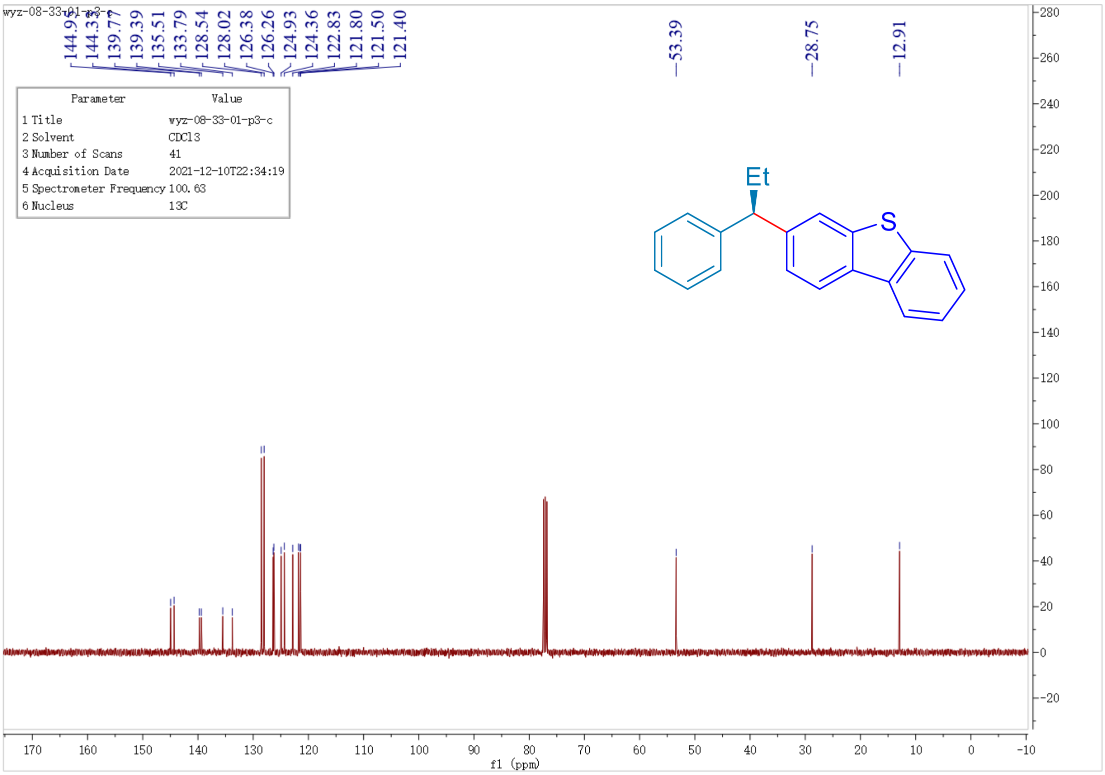

Compound 3w ^{1}H NMR (400 MHz, $CDCl_3$)

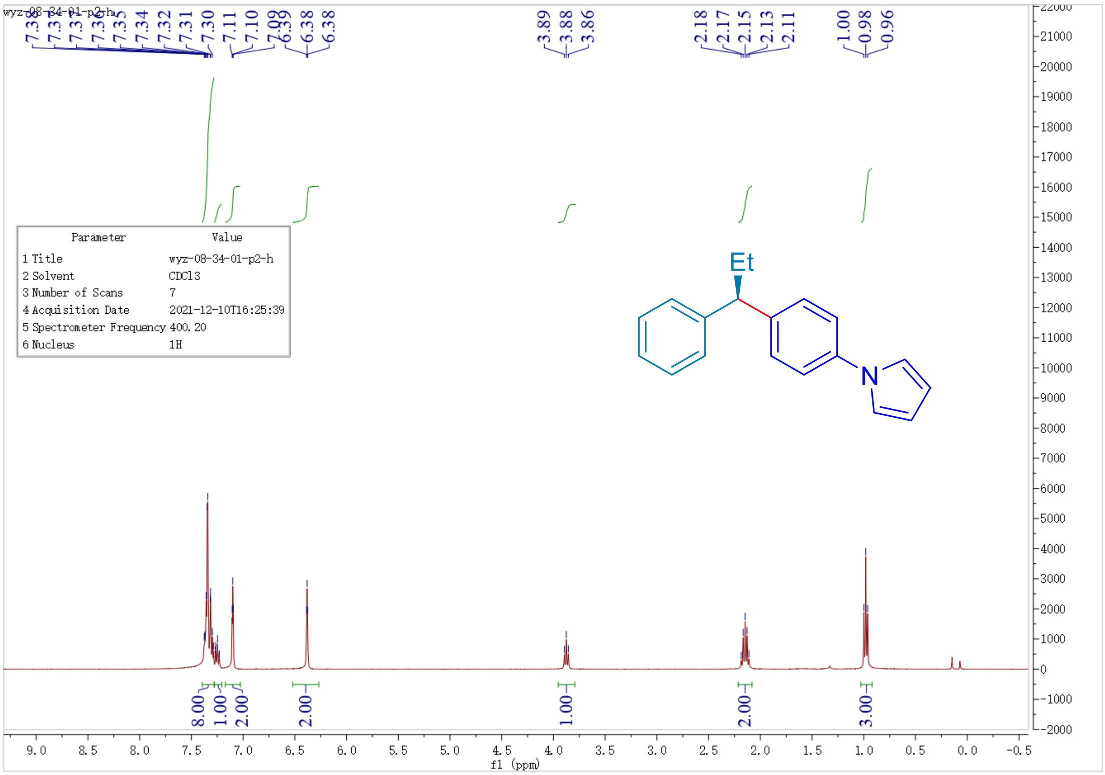

Compound 3w ^{13}C NMR (101 MHz, $CDCl_3$)

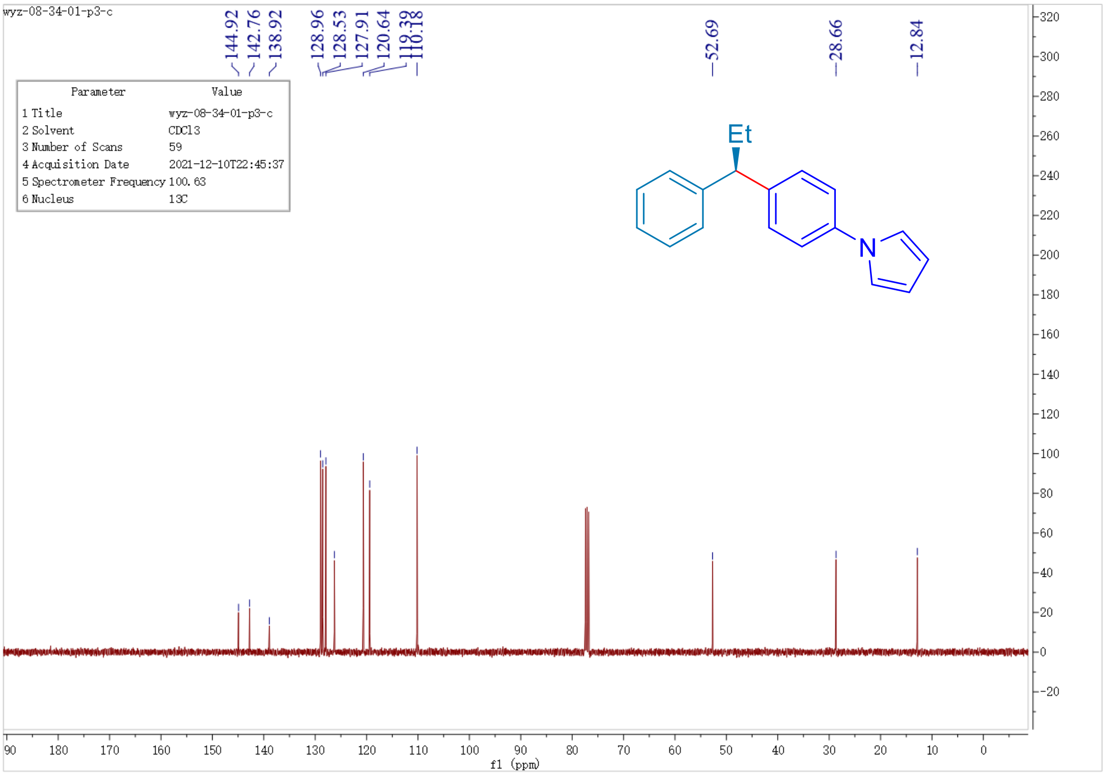

Compound 3x ^{1}H NMR (400 MHz, $CDCl_3$)

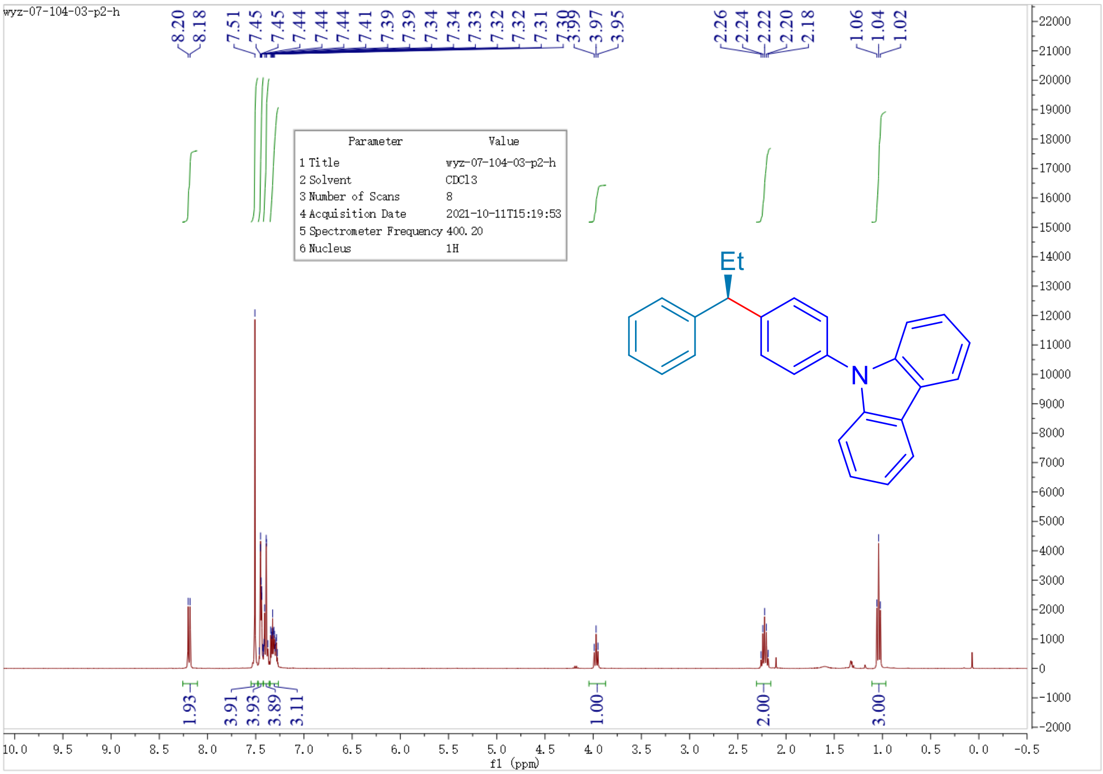

Compound 3x ^{13}C NMR (101 MHz, $CDCl_3$)

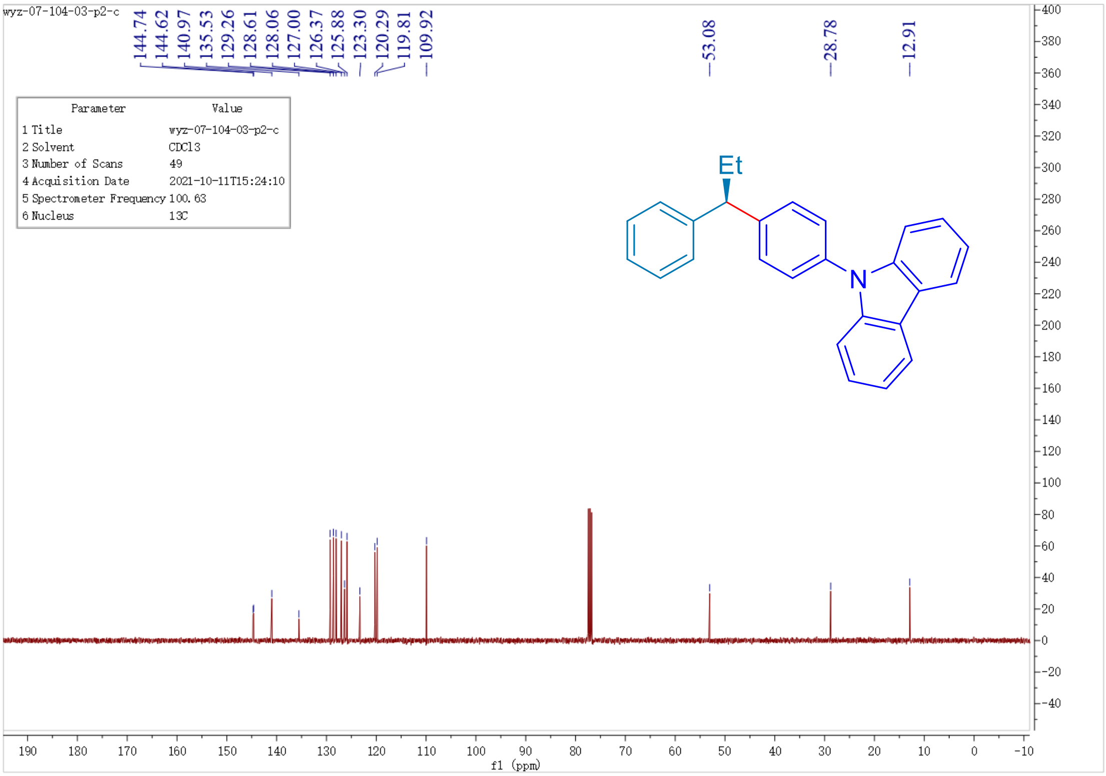

Compound 3y ^{1}H NMR (400 MHz, $CDCl_3$)

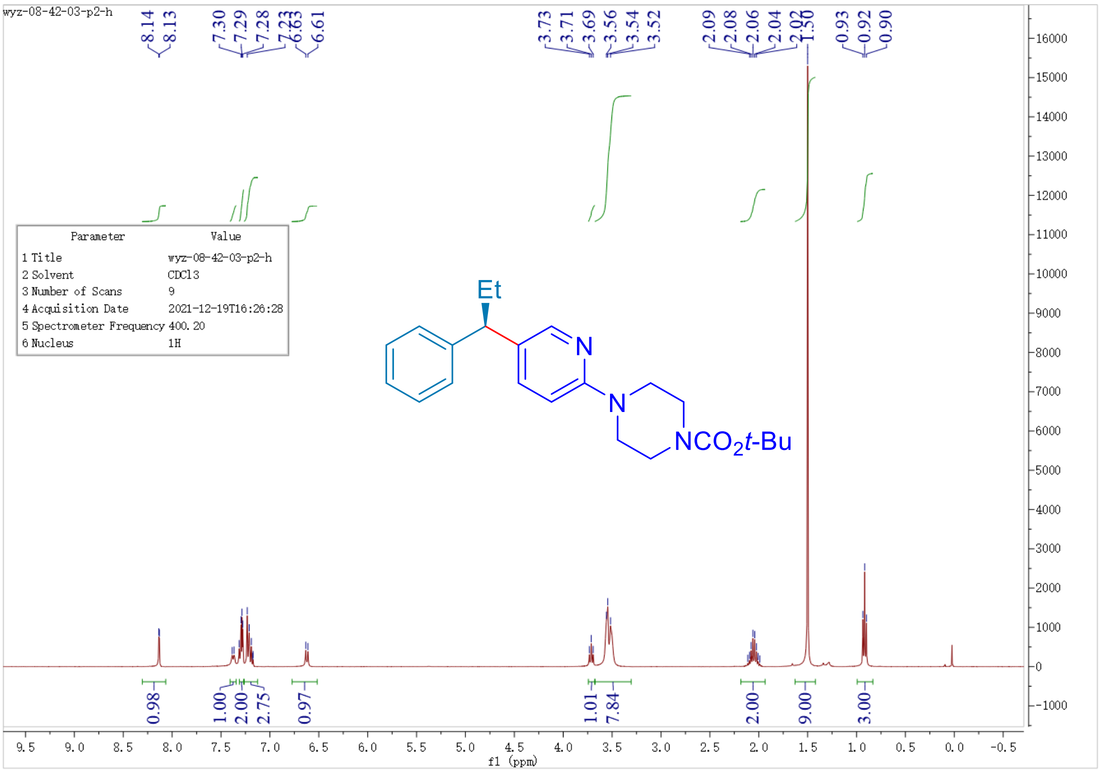

Compound 3y ^{13}C NMR (101 MHz, $CDCl_3$)

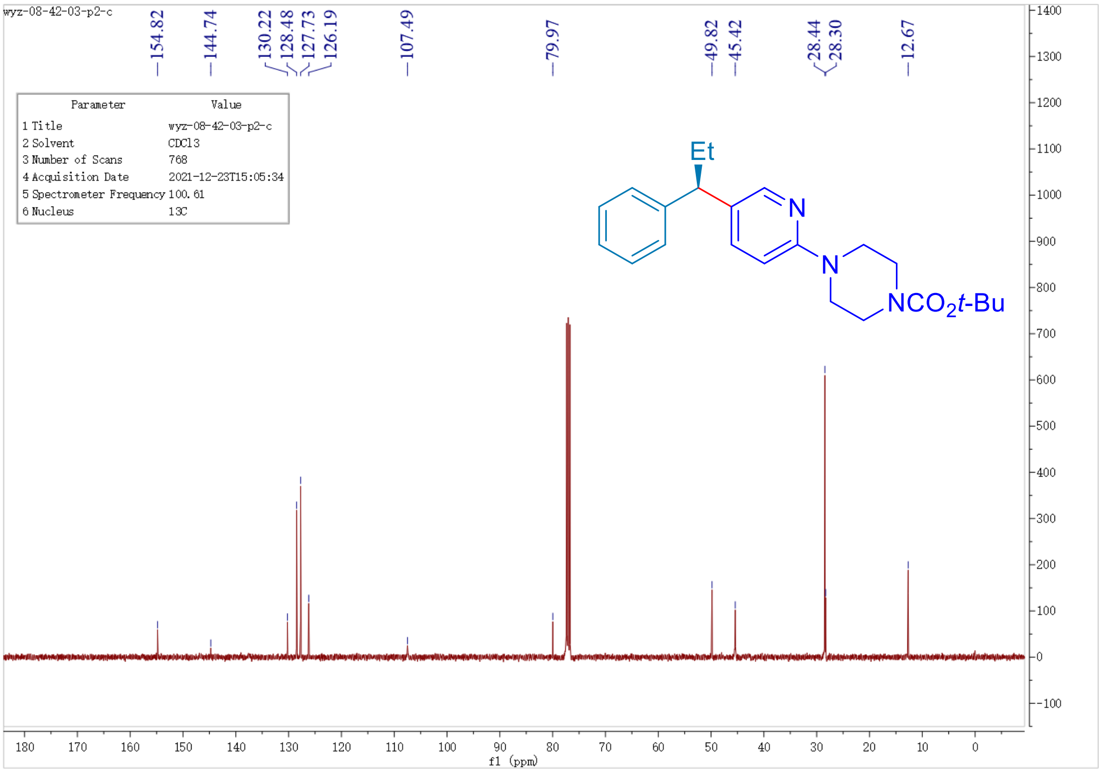

Compound 3z ^{1}H NMR (400 MHz, $CDCl_3$)

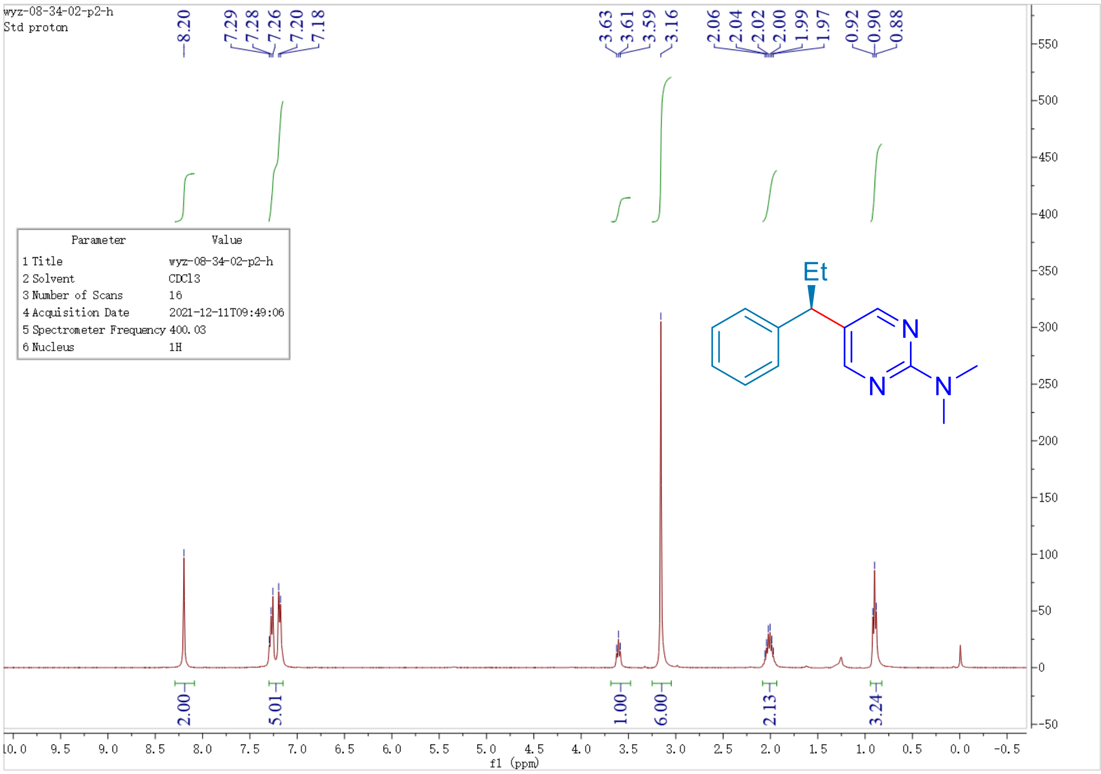

Compound 3z ^{13}C NMR (101 MHz, $CDCl_3$)

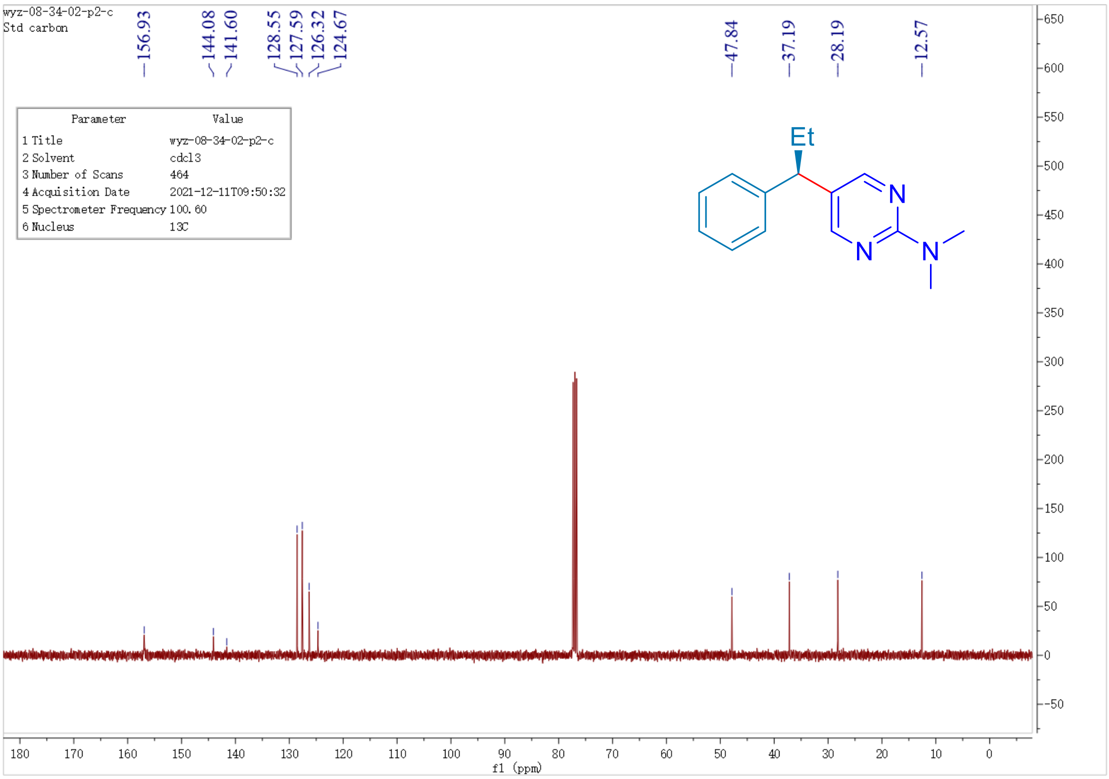

Compound 3aa ^{1}H NMR (400 MHz, $CDCl_3$)

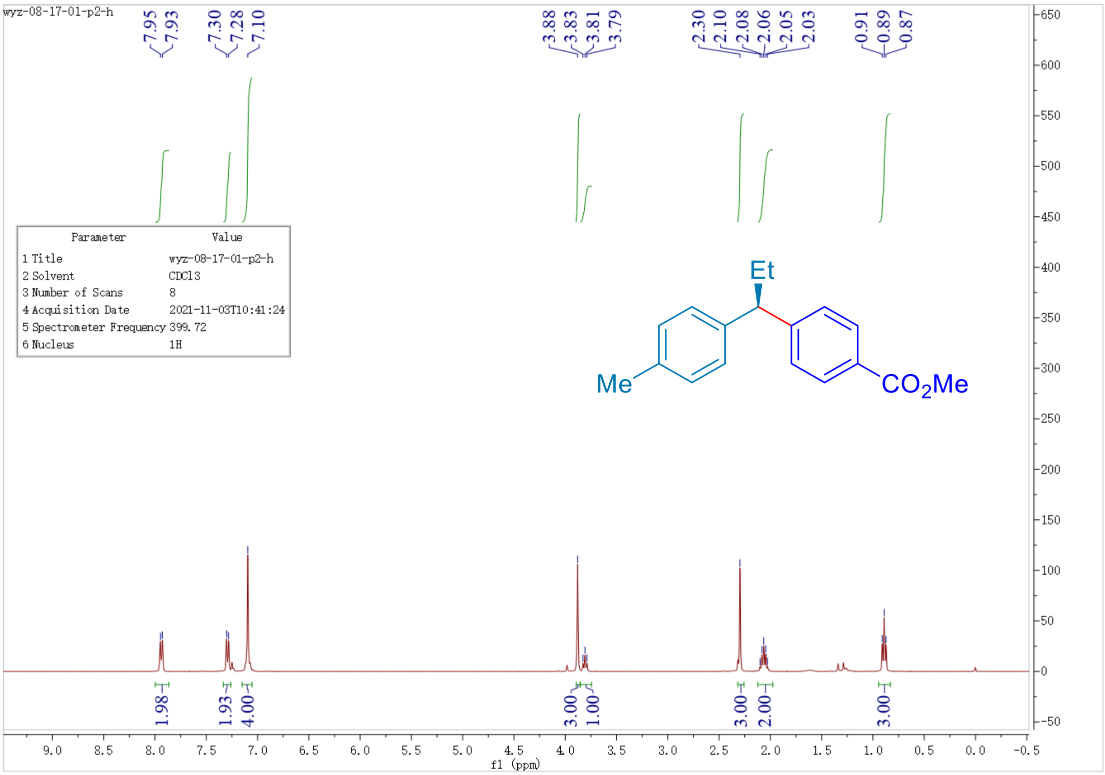

Compound 3aa ^{13}C NMR (101 MHz, $CDCl_3$)

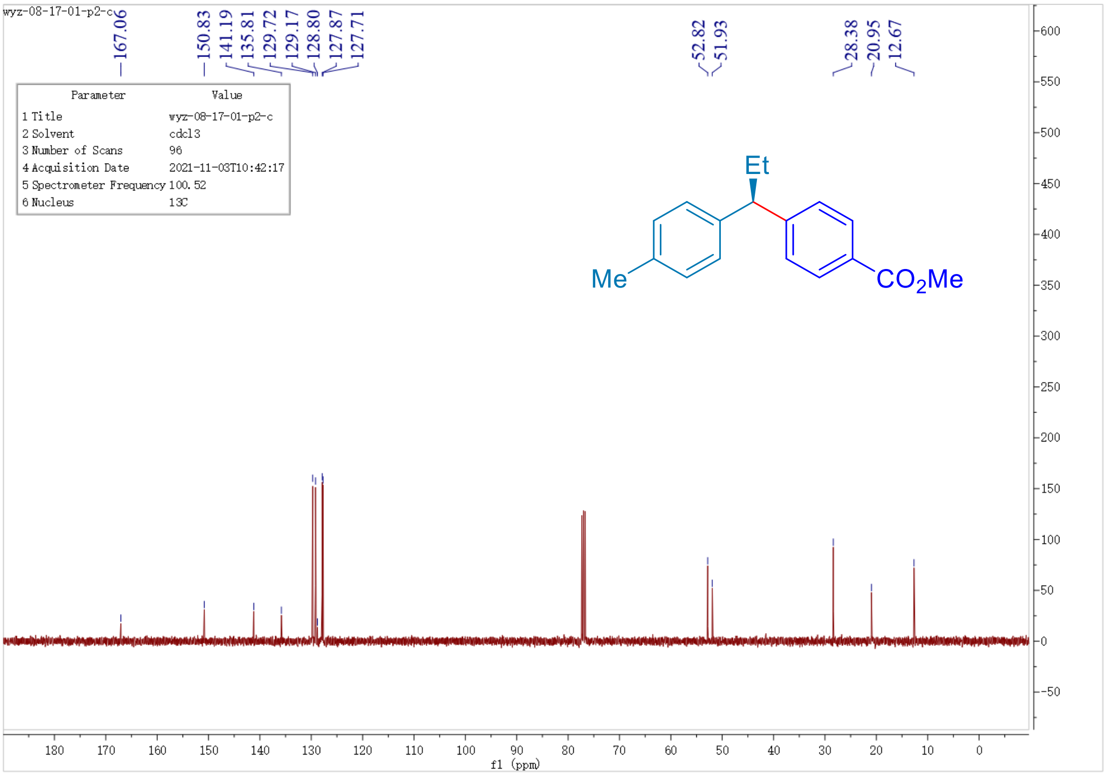

Compound 3ab ^{1}H NMR (400 MHz, $CDCl_3$)

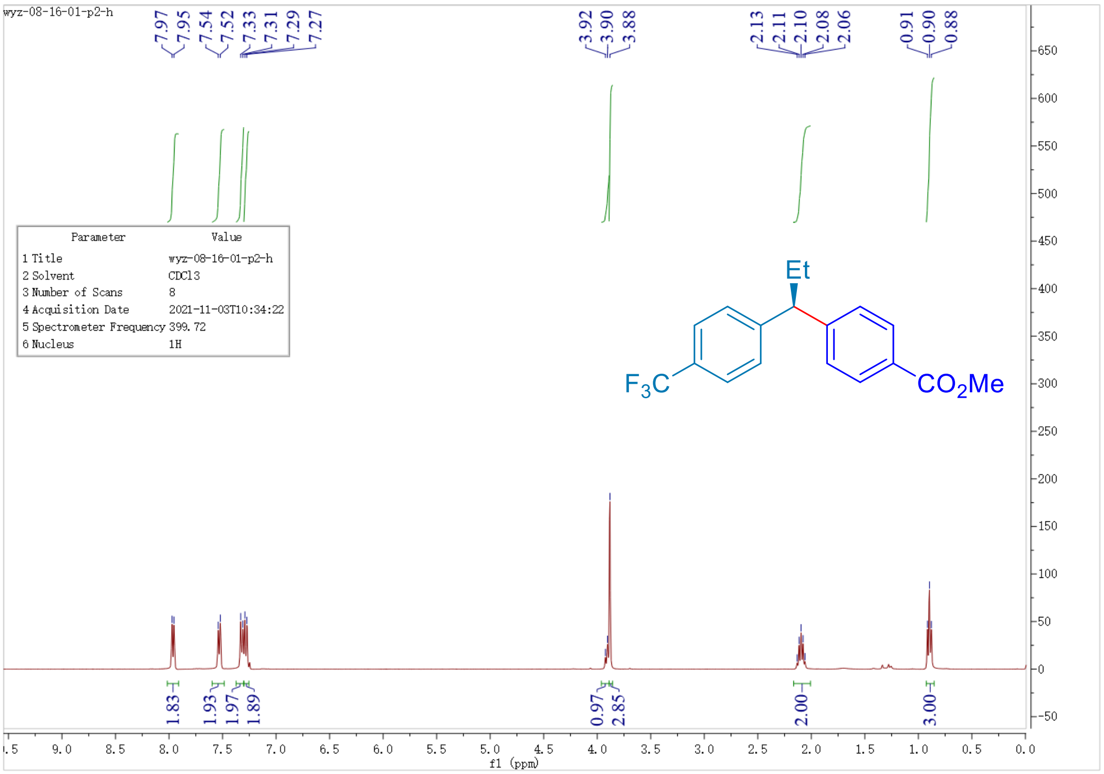

Compound 3ab ^{13}C NMR (101 MHz, $CDCl_3$)

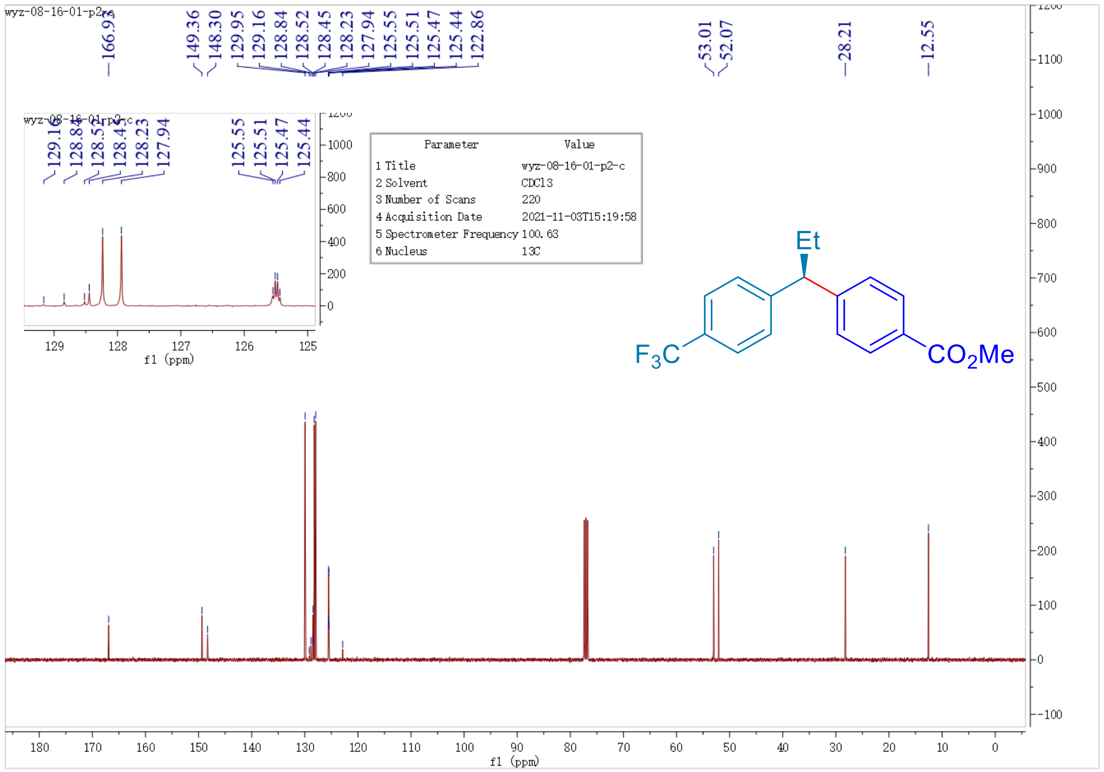

Compound 3ab ^{19}F NMR (377 MHz, $CDCl_3$)

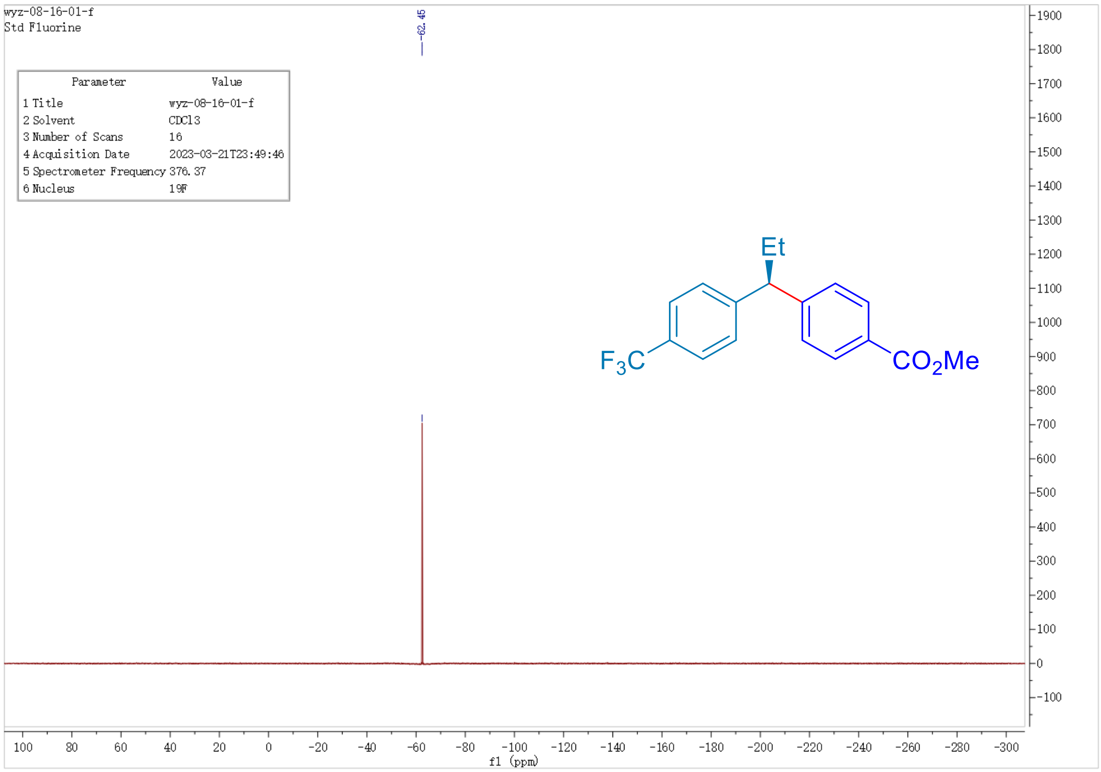

Compound 3ac ^{1}H NMR (400 MHz, $CDCl_3$)

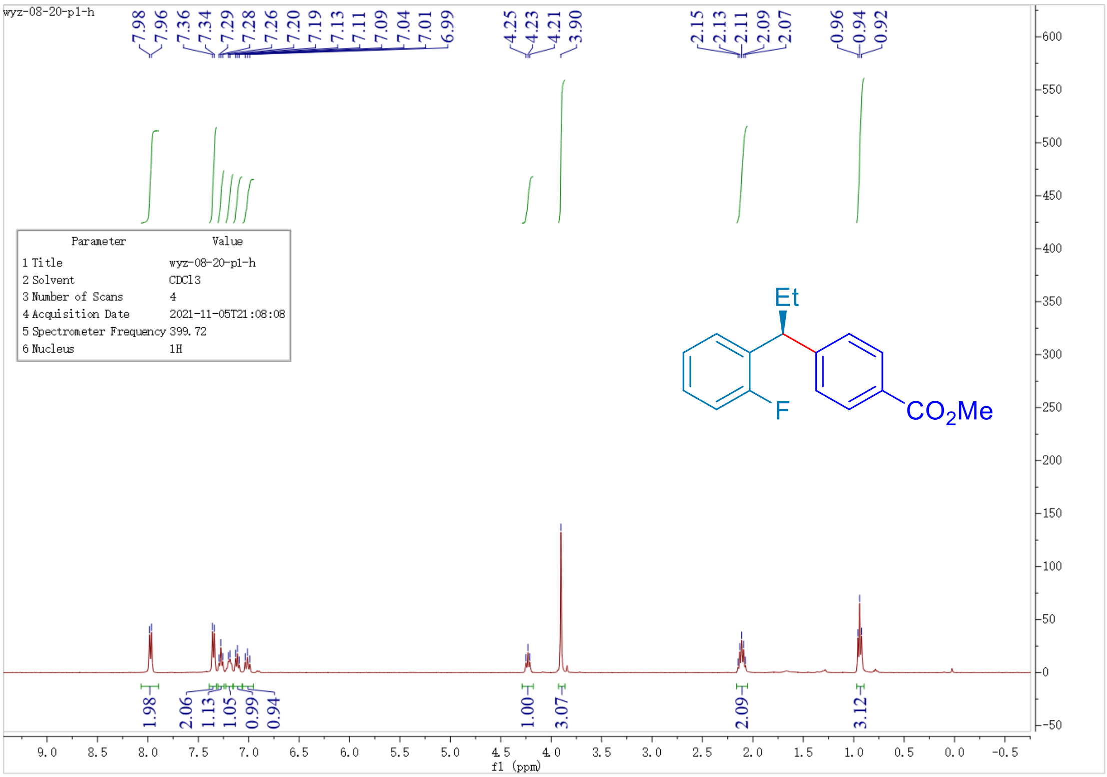

Compound 3ac ^{13}C NMR (101 MHz, $CDCl_3$)

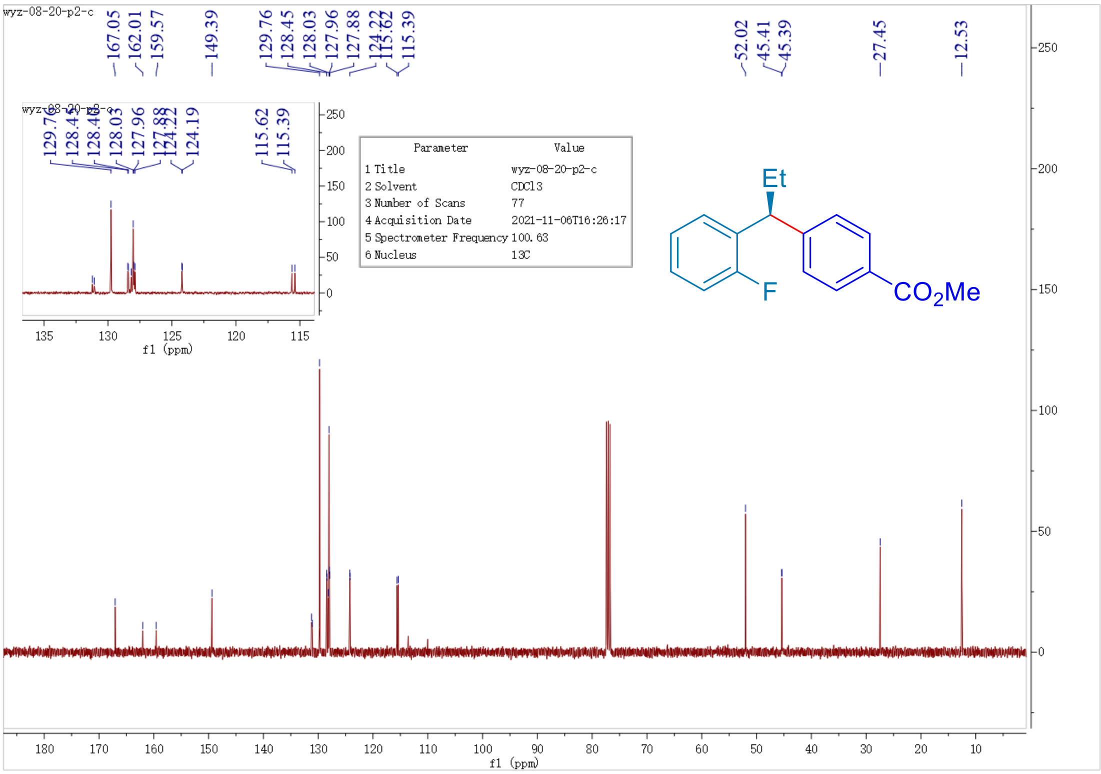

Compound 3ac ^{19}F NMR (377 MHz, $CDCl_3$)

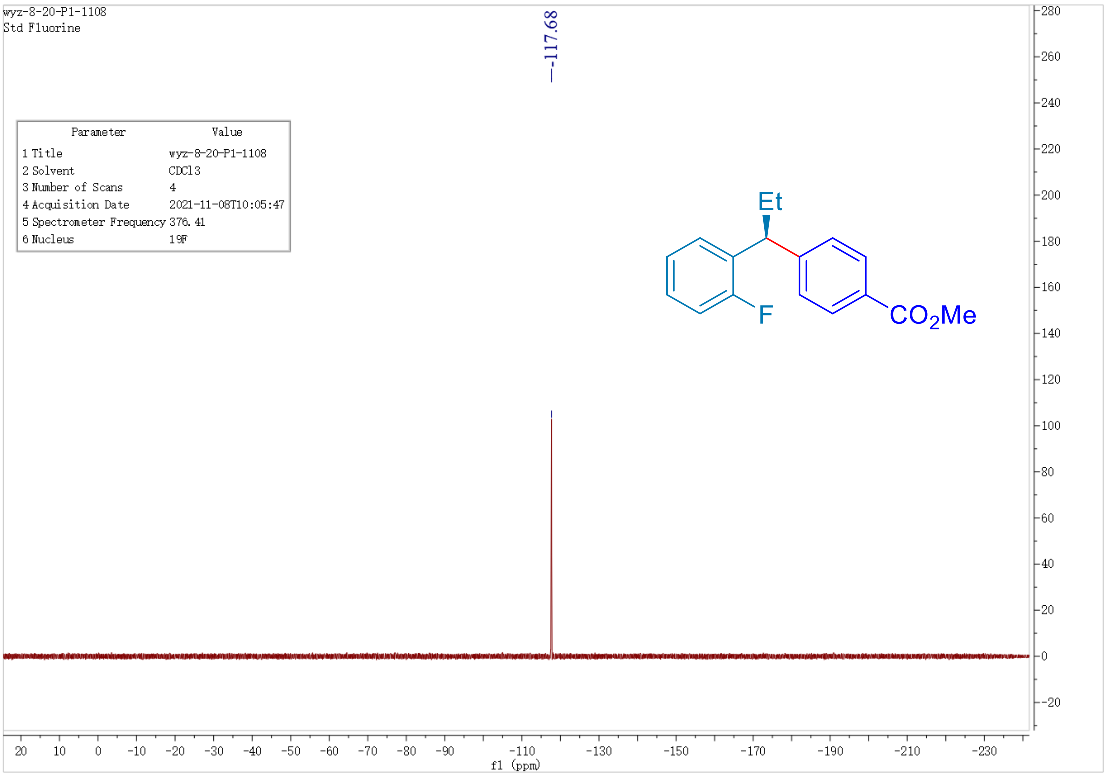

Compound 3ad ^{1}H NMR (400 MHz, $CDCl_3$)

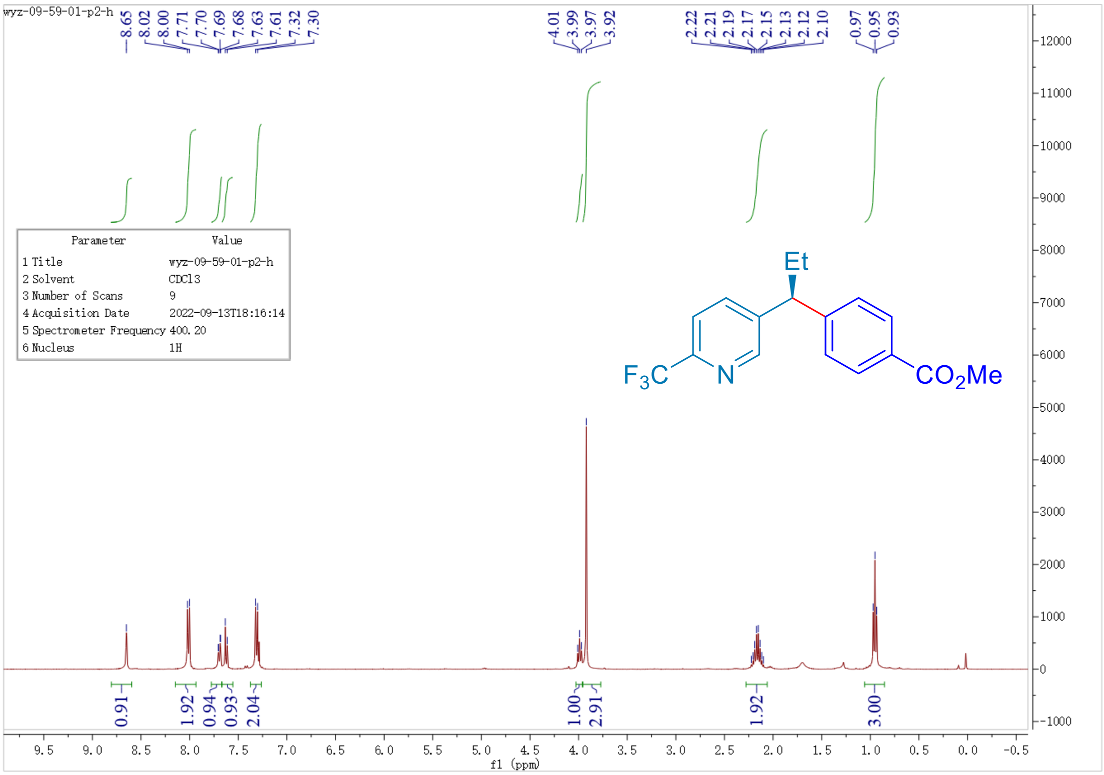

Compound 3ad ^{13}C NMR (101 MHz, $CDCl_3$)

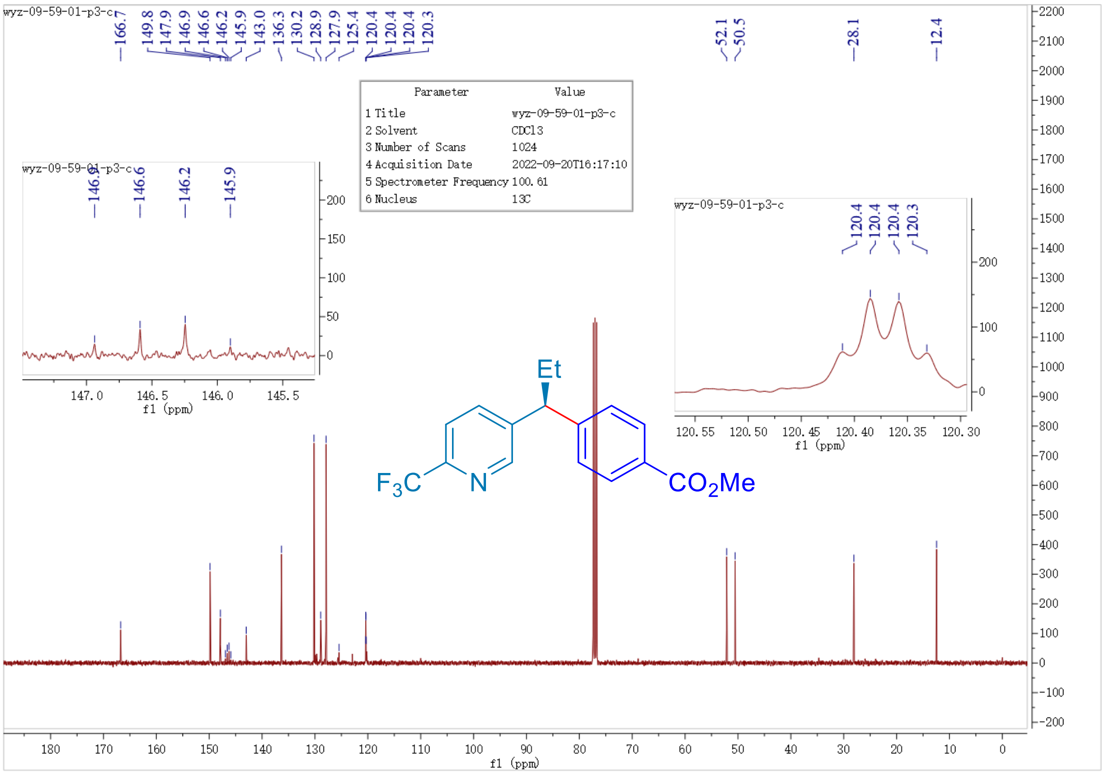

Compound 3ad ^{19}F NMR (377 MHz, $CDCl_3$)

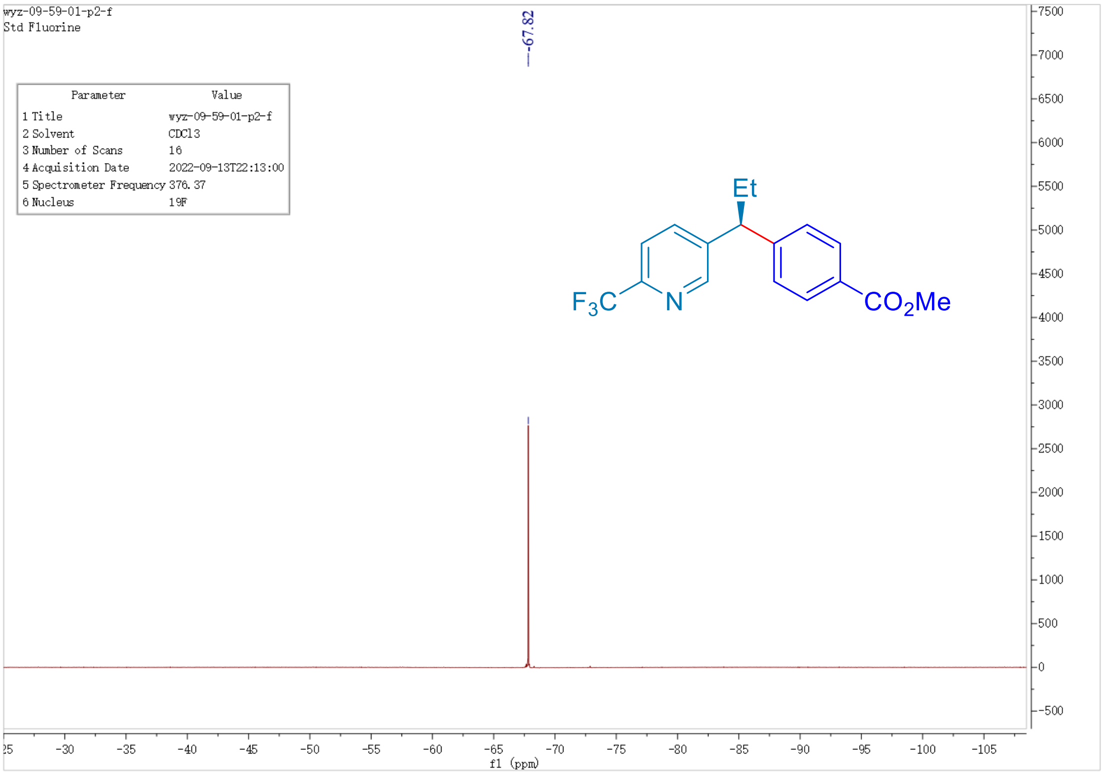

Compound 3ae ^{1}H NMR (400 MHz, $CDCl_3$)

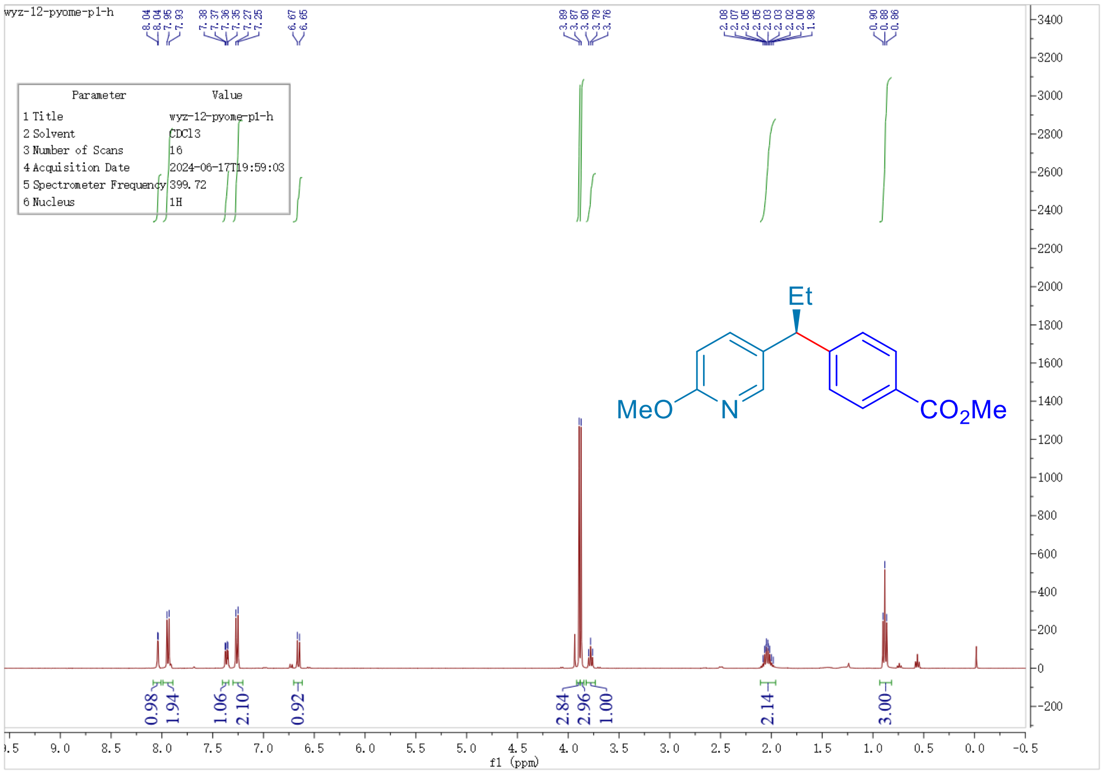

Compound 3ae ^{13}C NMR (101 MHz, $CDCl_3$)

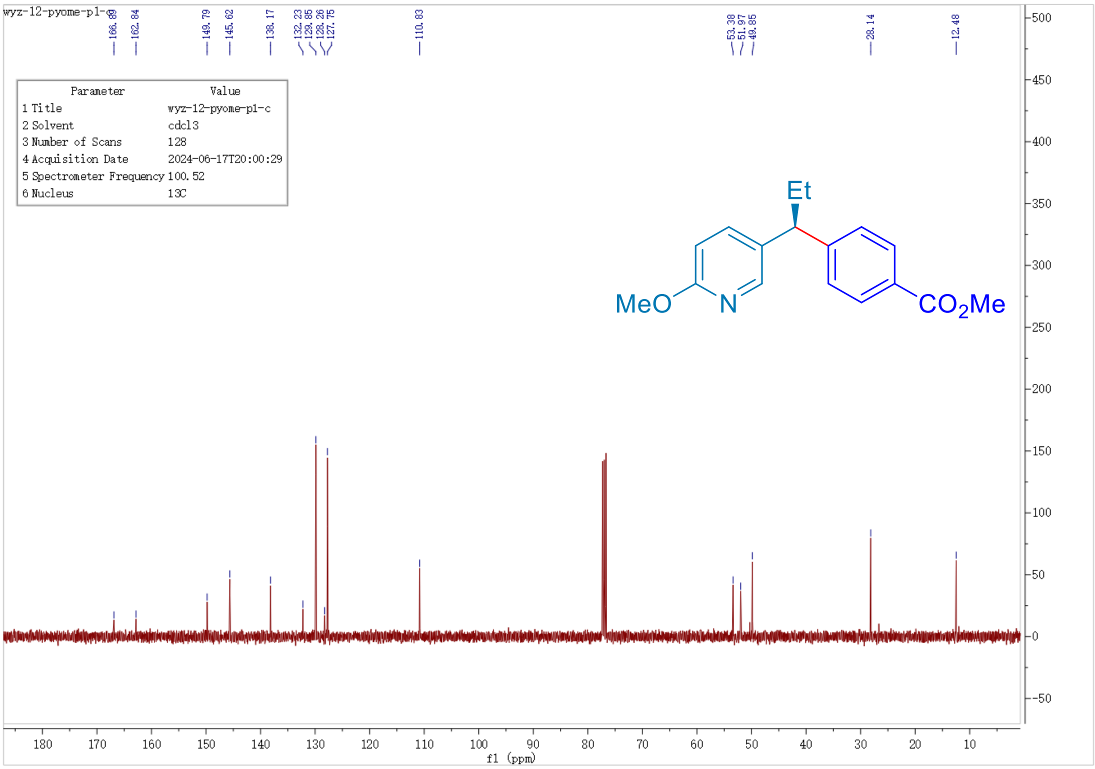

Compound 3af ^{1}H NMR (400 MHz, $CDCl_3$)

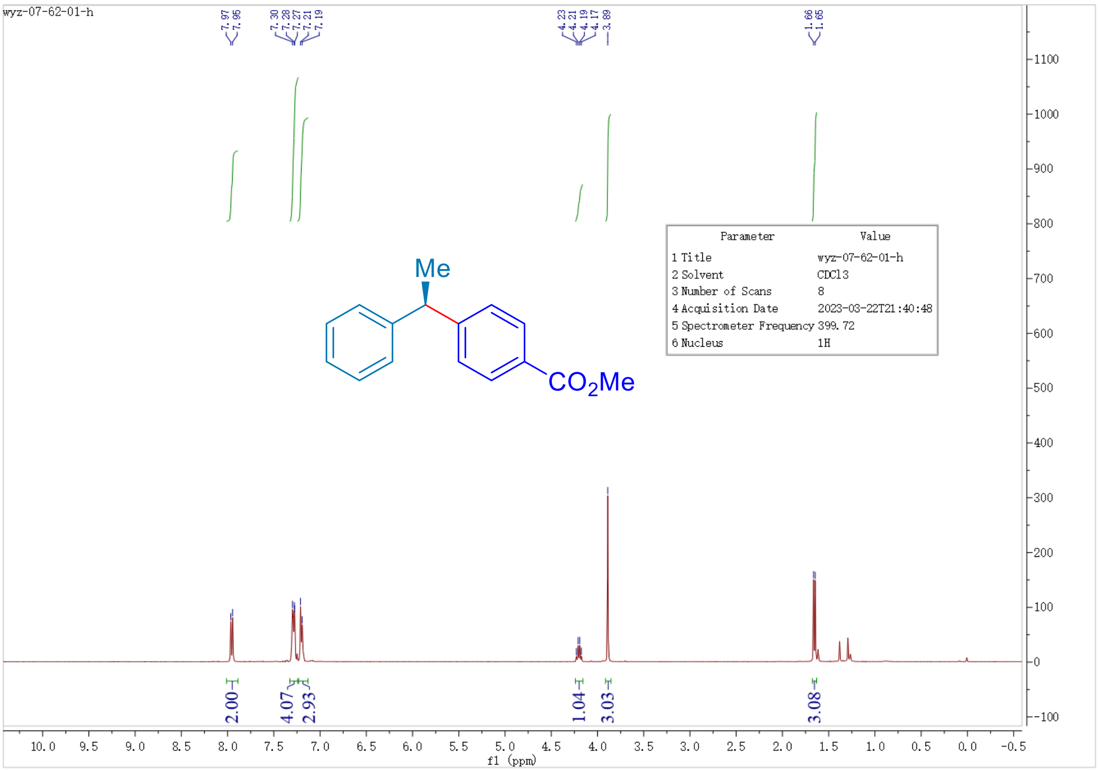

Compound 3af ^{13}C NMR (101 MHz, $CDCl_3$)

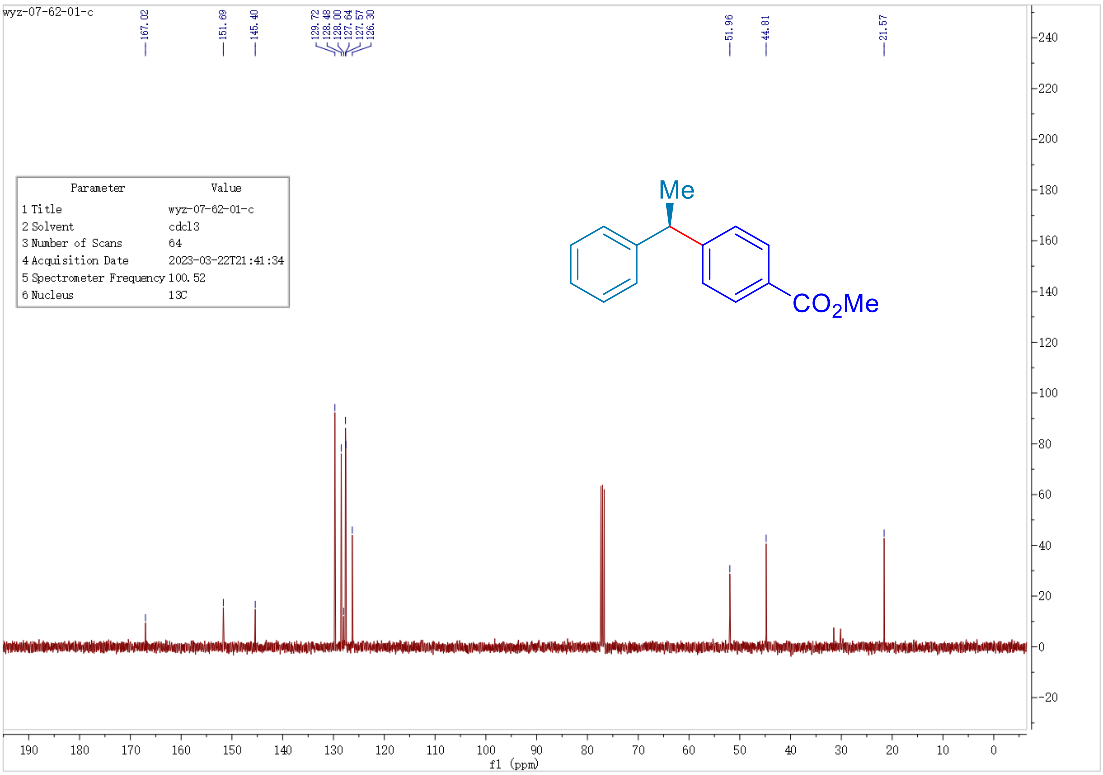

Compound 3ag ^{1}H NMR (400 MHz, $CDCl_3$)

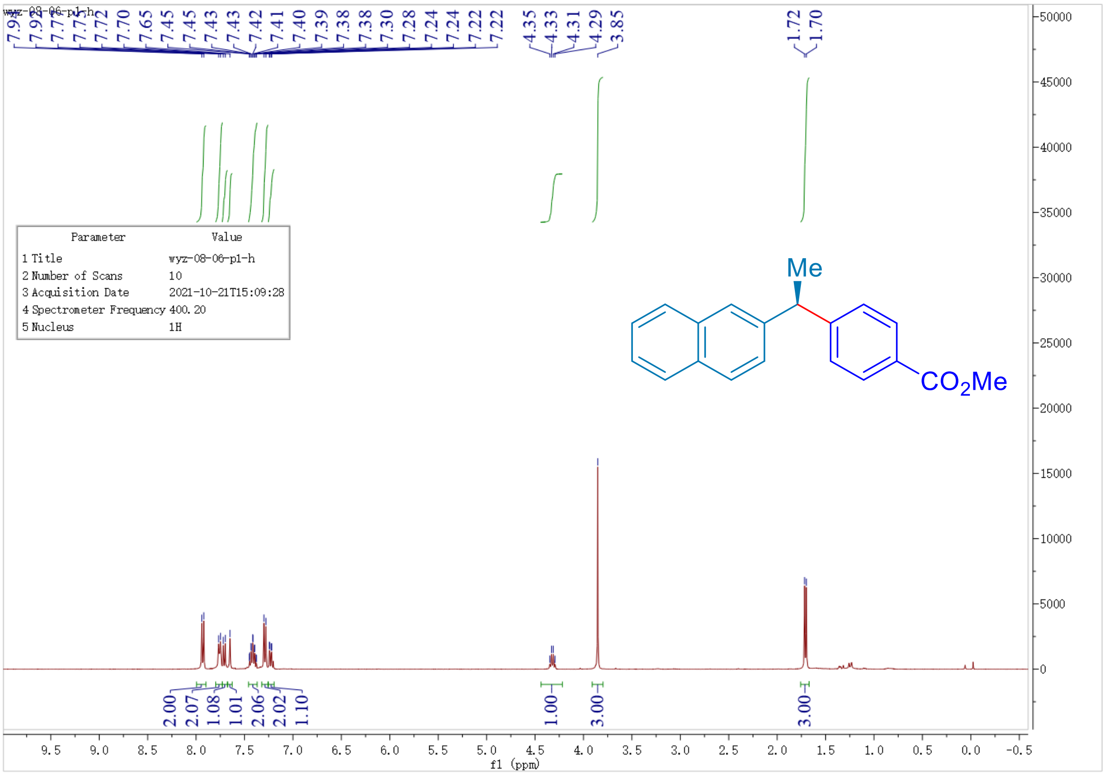

Compound 3ag ^{13}C NMR (101 MHz, $CDCl_3$)

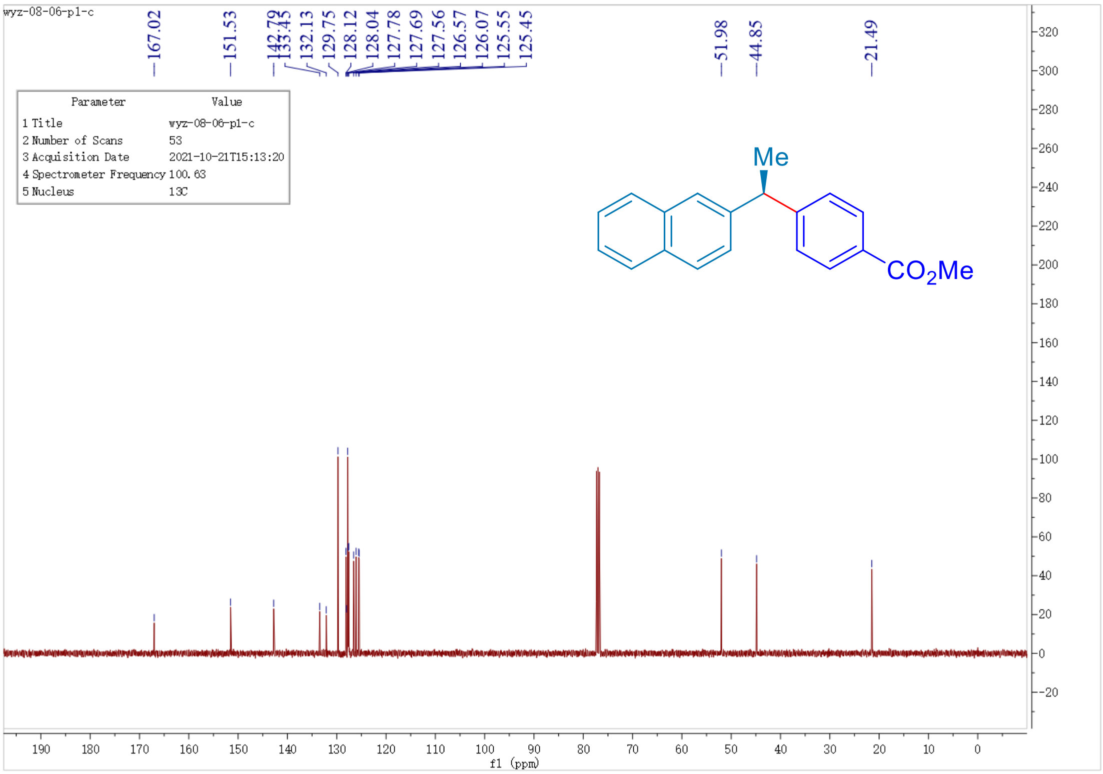

Compound 3ah ^{1}H NMR (400 MHz, $CDCl_3$)

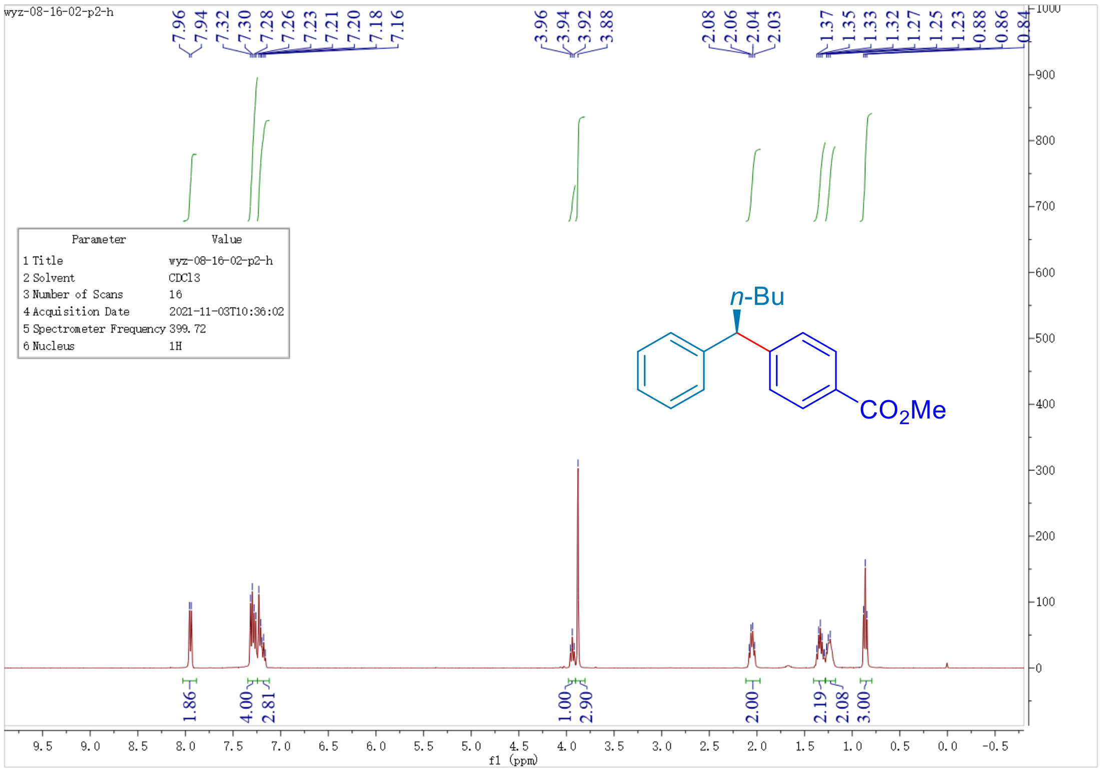

Compound 3ah ^{13}C NMR (101 MHz, $CDCl_3$)

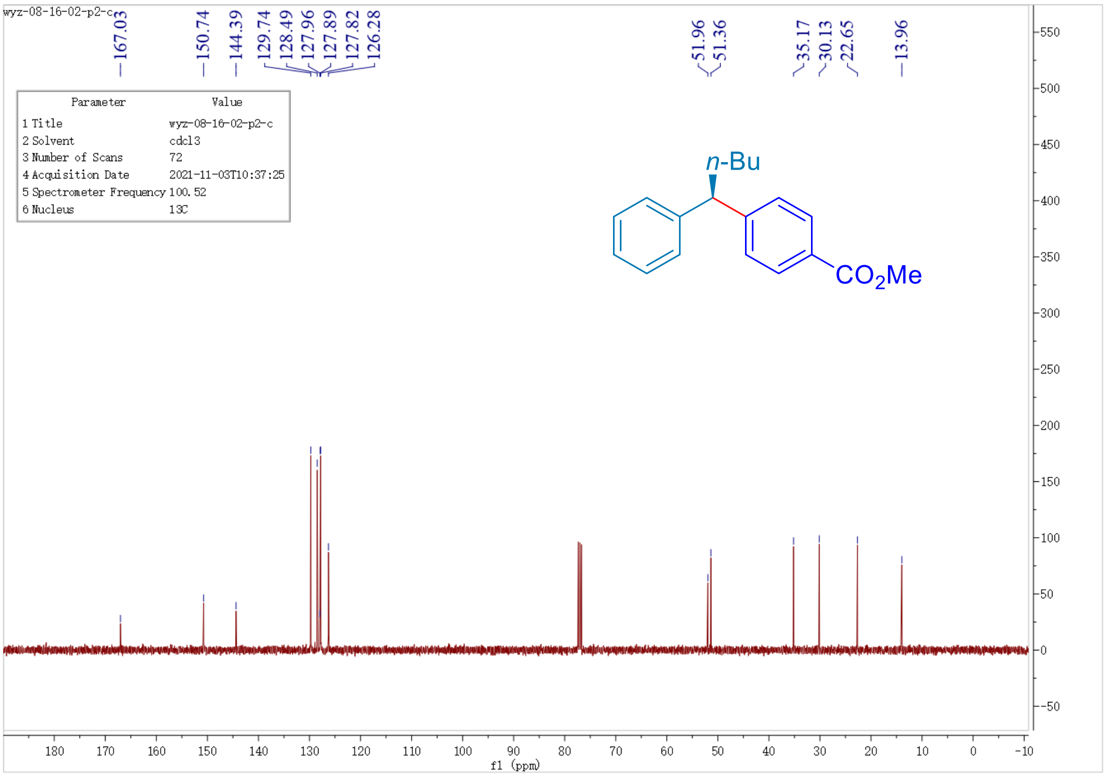

Compound 3ai ^{1}H NMR (400 MHz, $CDCl_3$)

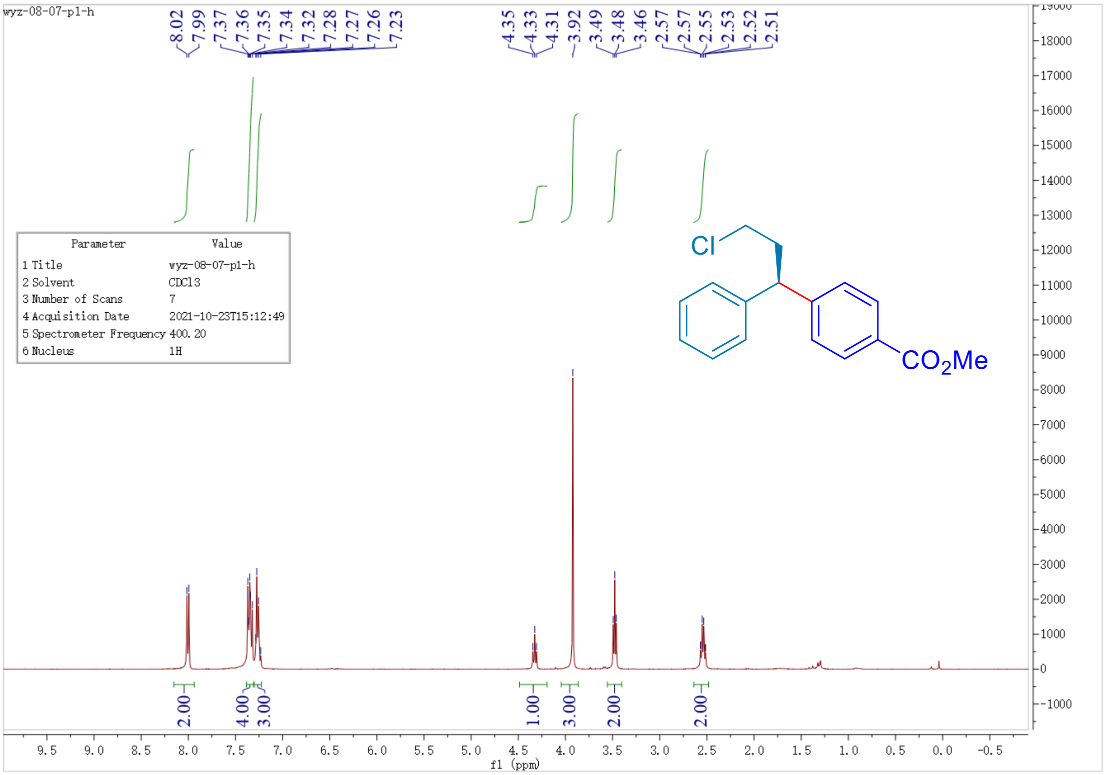

Compound 3ai ^{13}C NMR (101 MHz, $CDCl_3$)

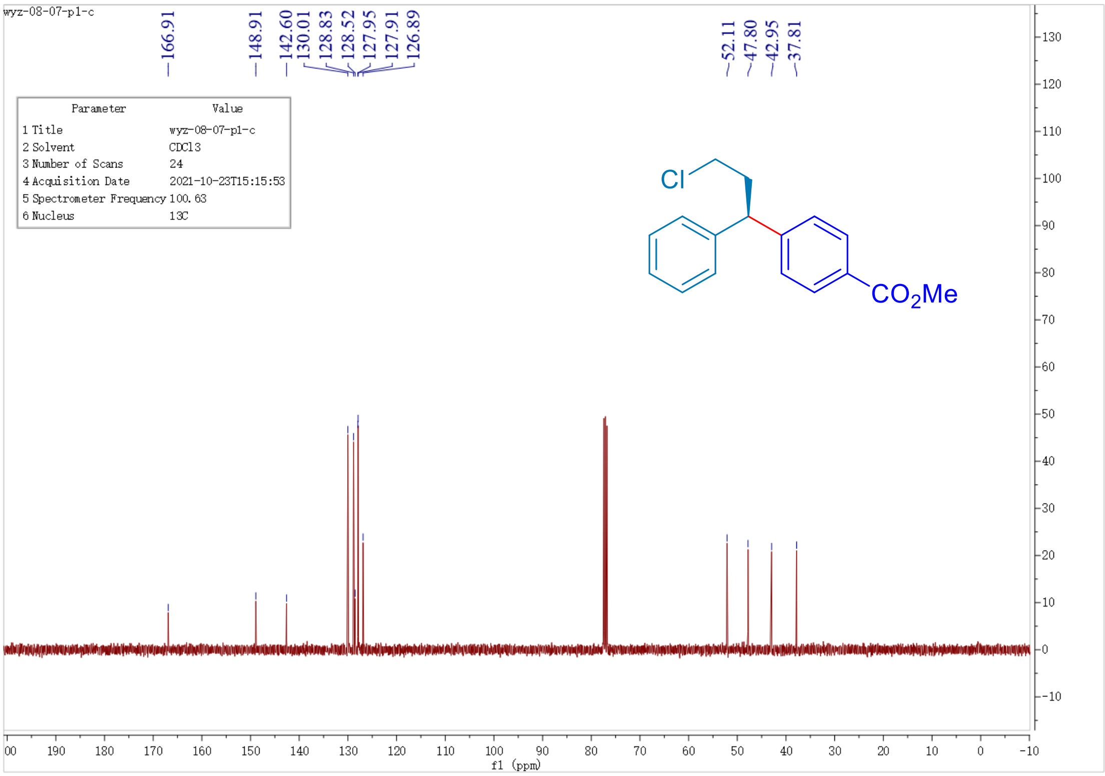

Compound 3aj ^{1}H NMR (400 MHz, $CDCl_3$)

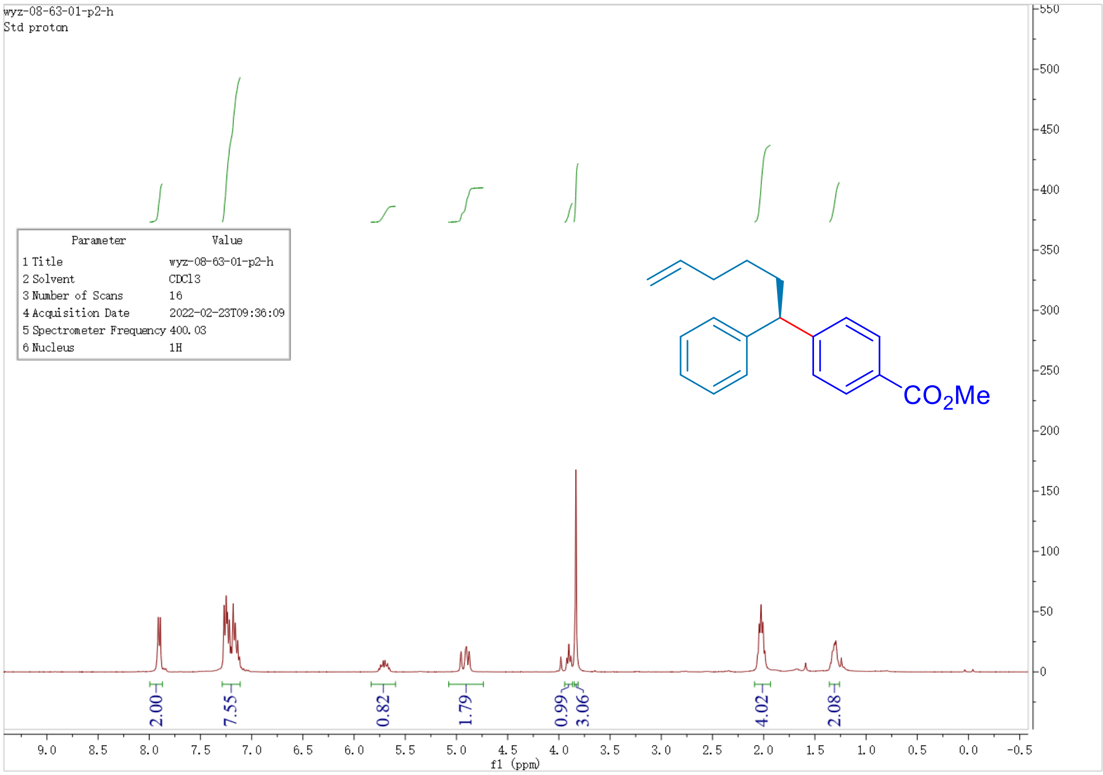

Compound 3aj ^{13}C NMR (101 MHz, $CDCl_3$)

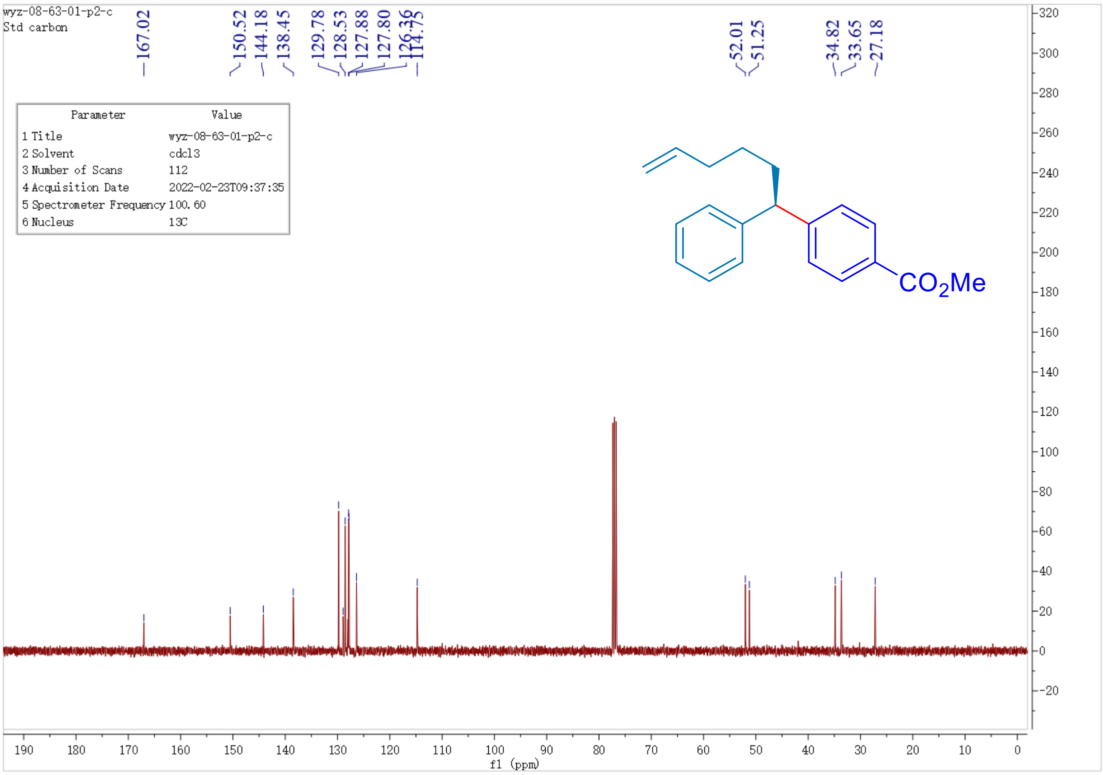

Compound 3ak ^{1}H NMR (400 MHz, $CDCl_3$)

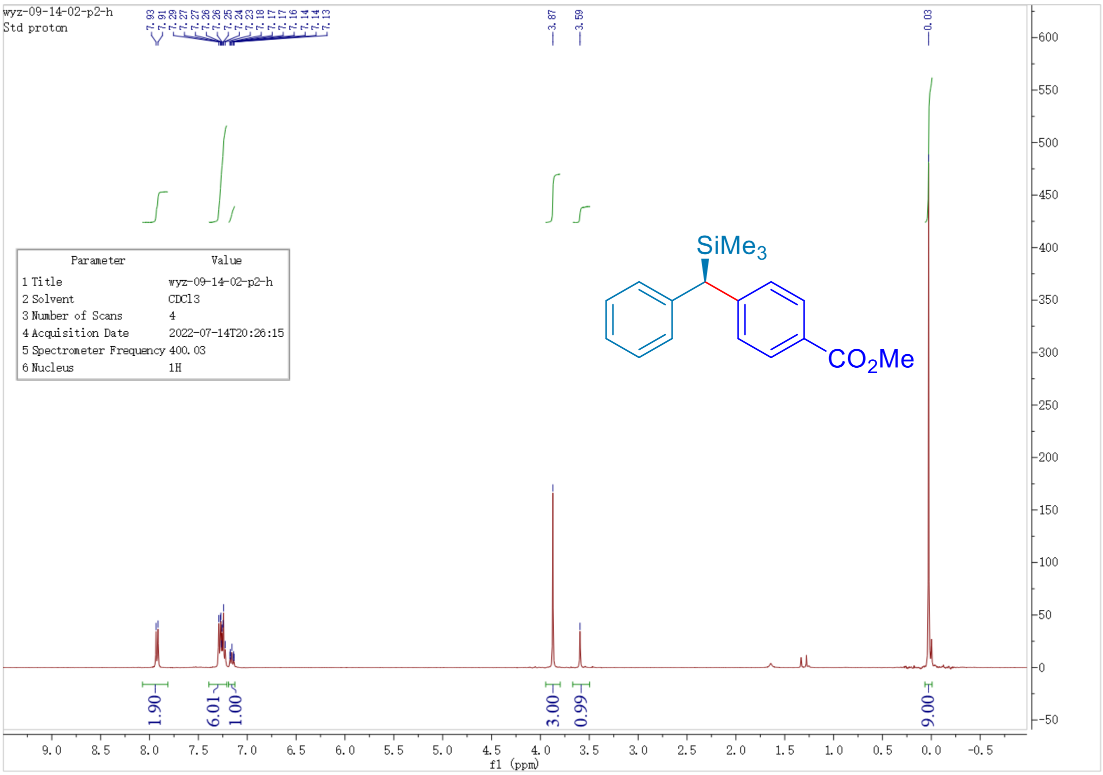

Compound 3ak ^{13}C NMR (101 MHz, $CDCl_3$)

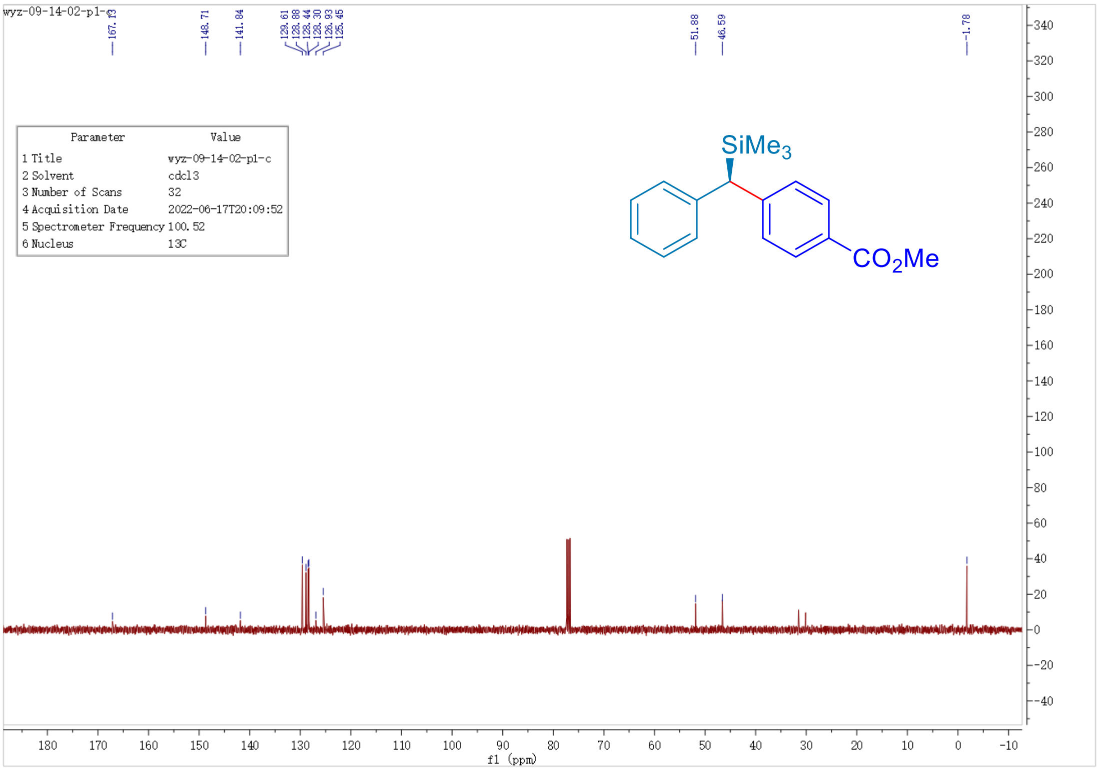

Compound 3al ^{1}H NMR (400 MHz, $CDCl_3$)

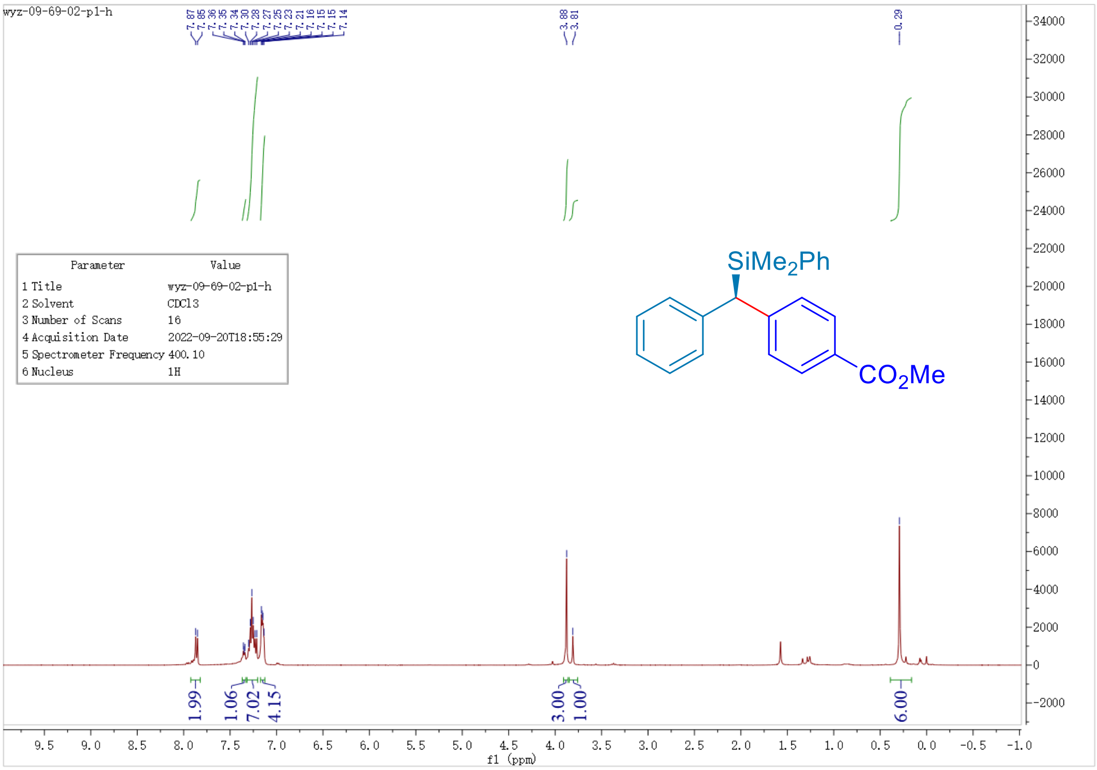

Compound 3al ^{13}C NMR (101 MHz, $CDCl_3$)

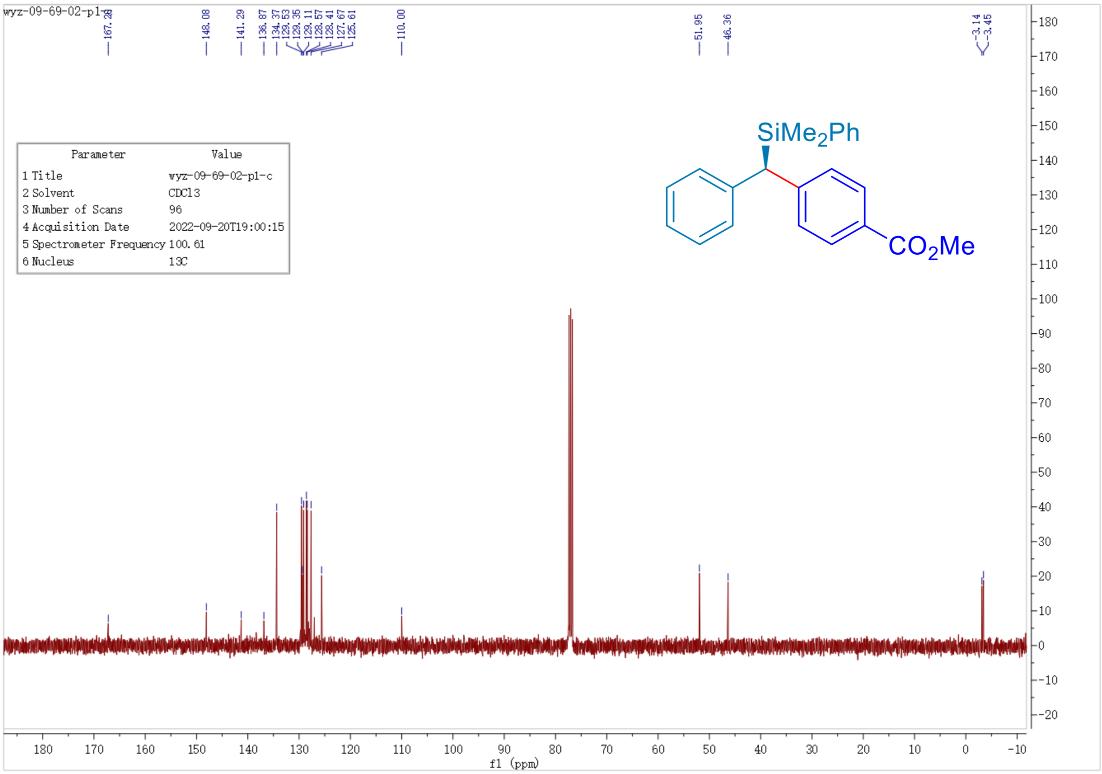

Compound 3am ^{1}H NMR (400 MHz, $CDCl_3$)

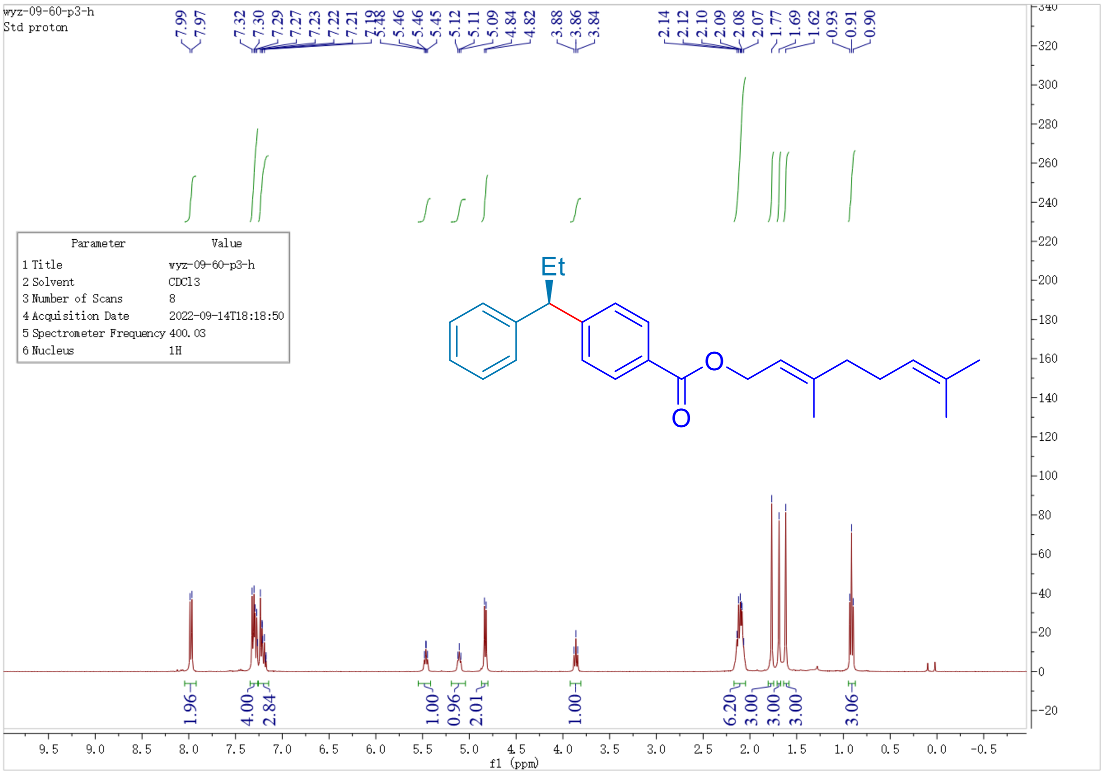

Compound 3am ^{13}C NMR (101 MHz, $CDCl_3$)

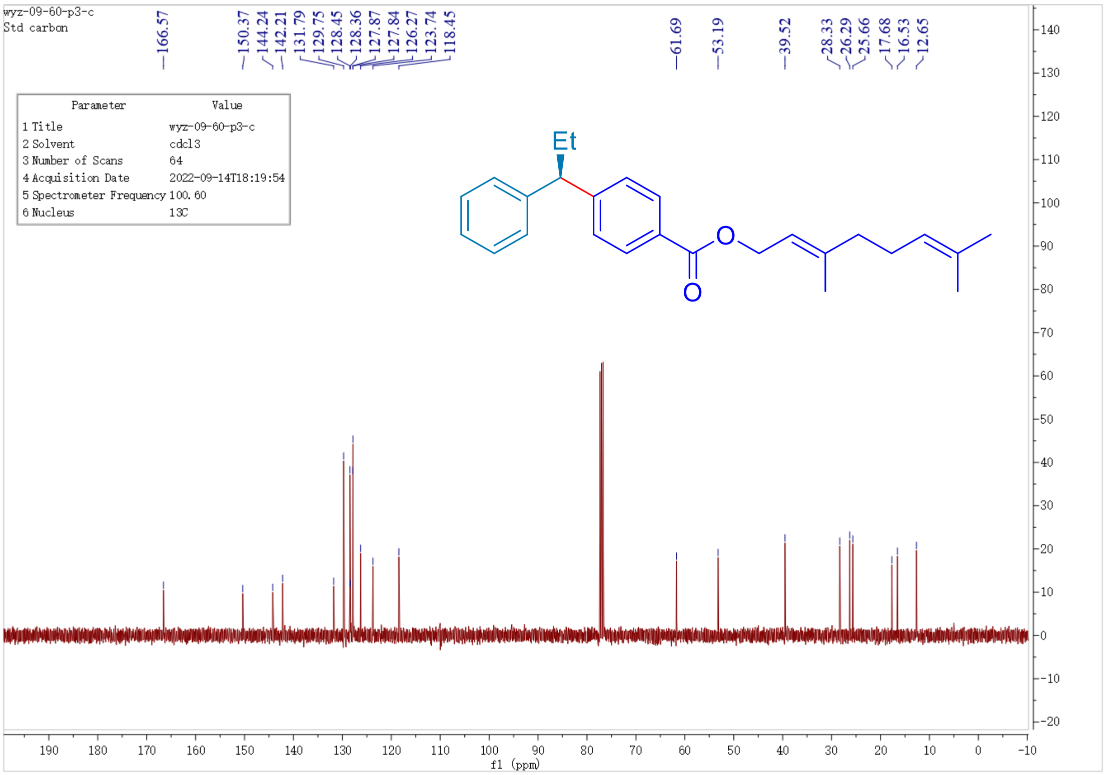

Compound 3an ^{1}H NMR (400 MHz, $CDCl_3$)

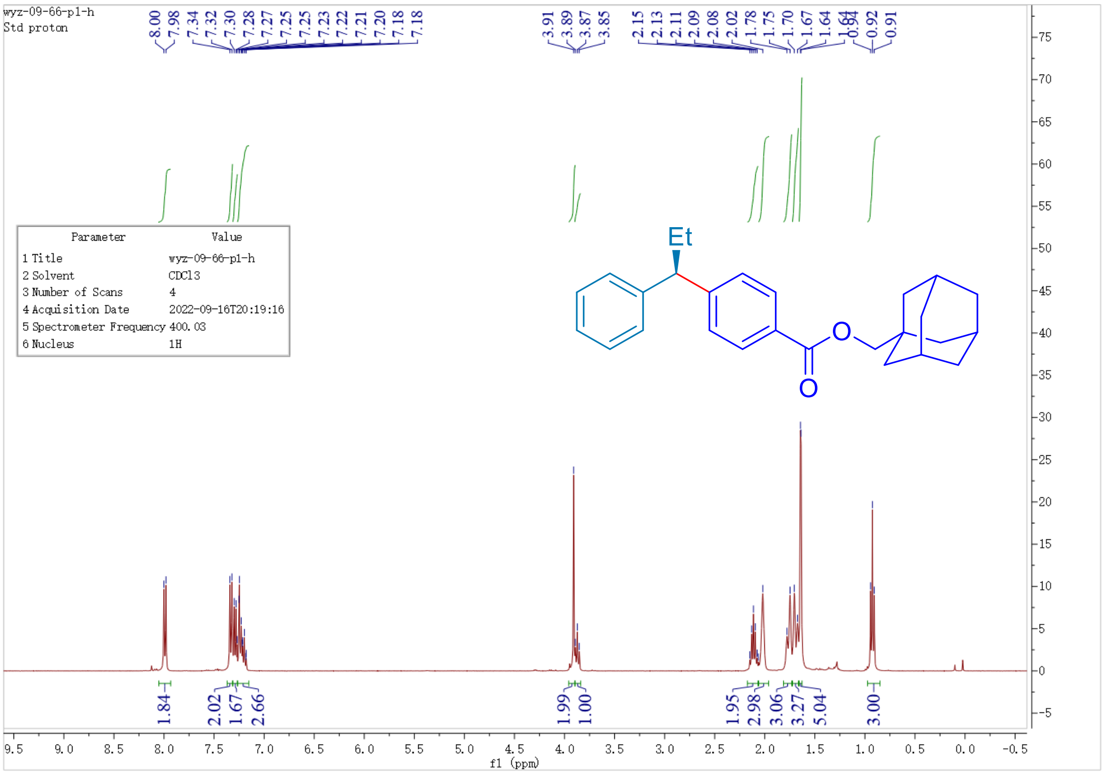

Compound 3an ^{13}C NMR (101 MHz, $CDCl_3$)

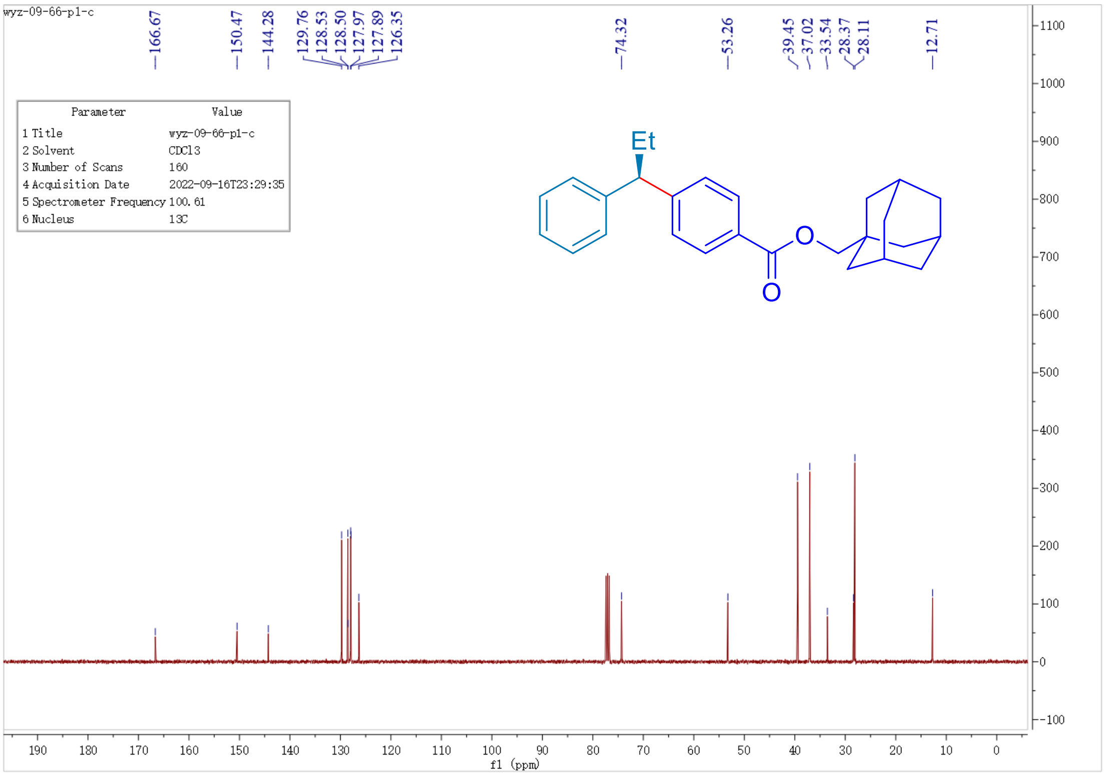

Compound 3ao ^{1}H NMR (400 MHz, $CDCl_3$)

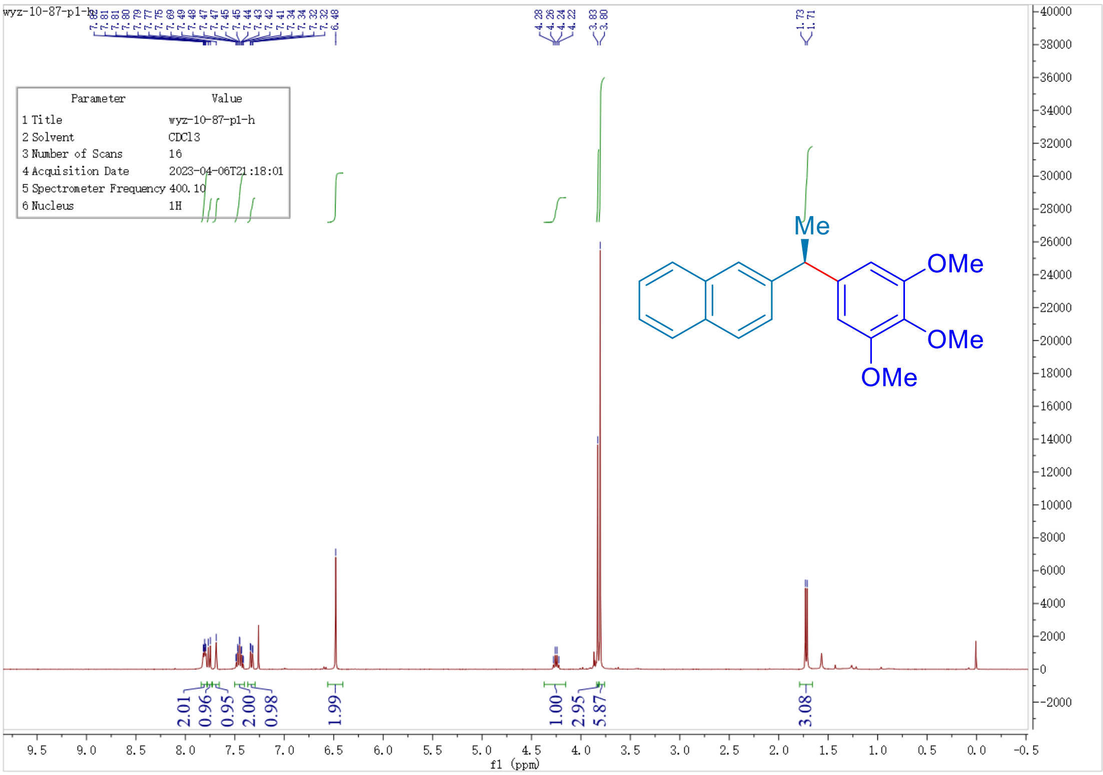

Compound 3ao ^{13}C NMR (101 MHz, $CDCl_3$)

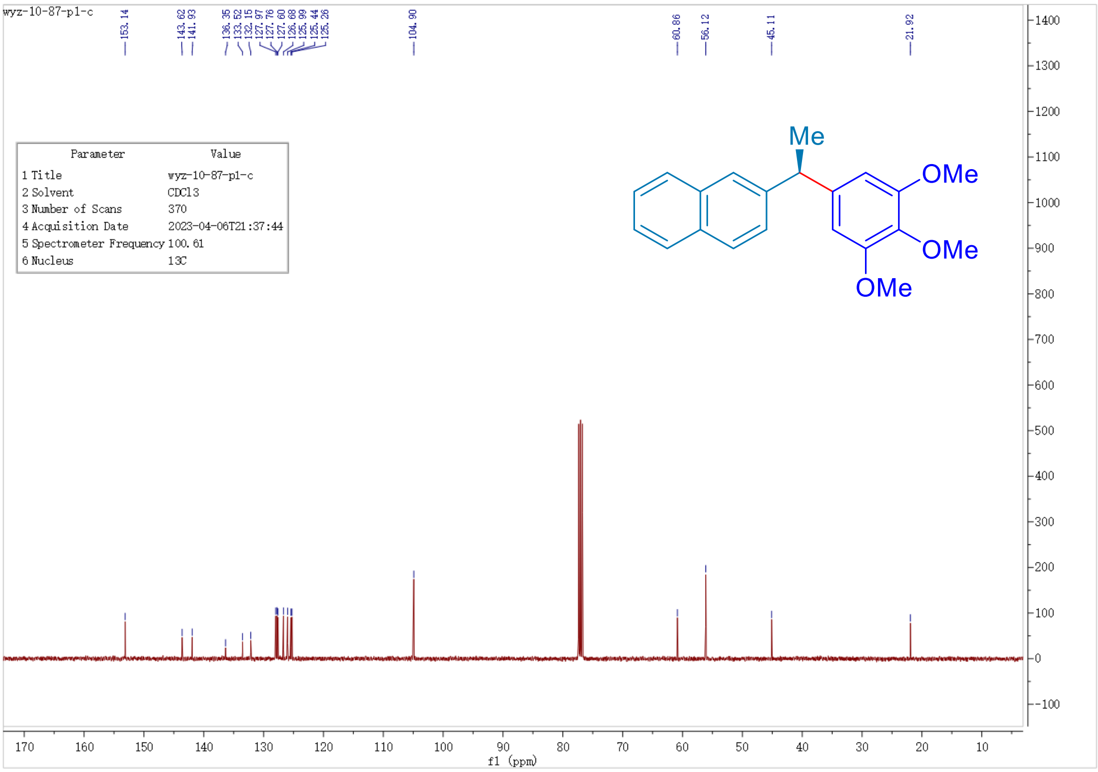

Compound 3ap ^{1}H NMR (400 MHz, $CDCl_3$)

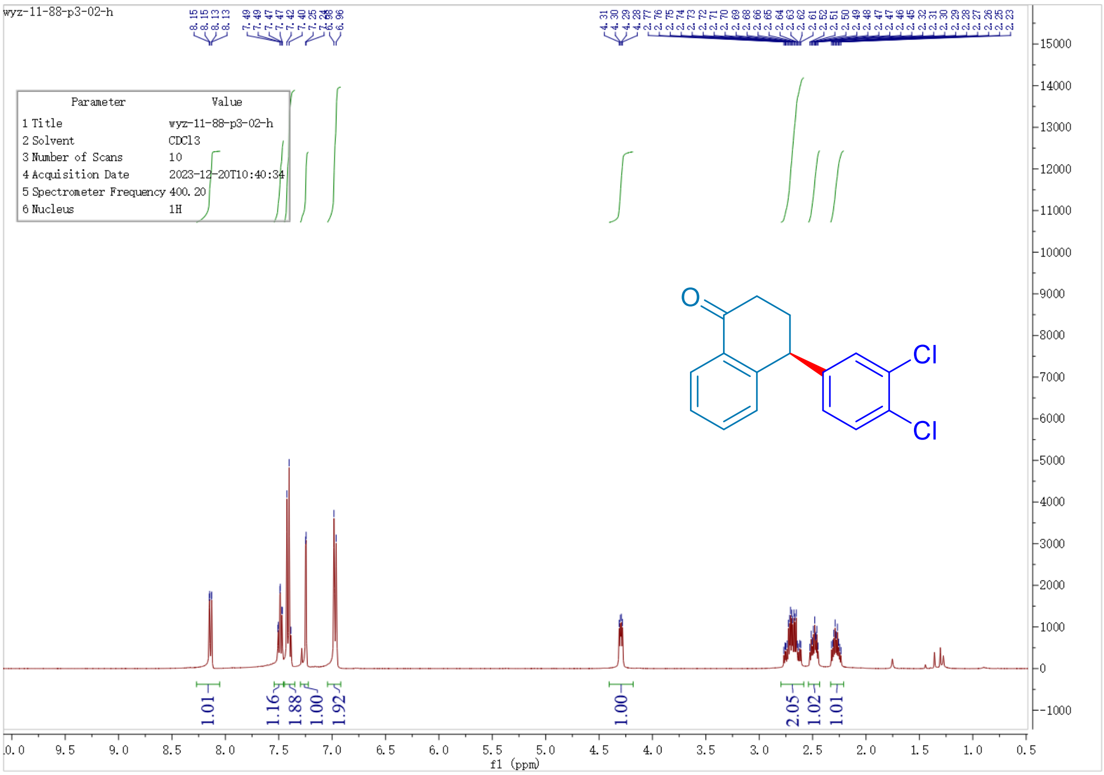

Compound 3ap ^{13}C NMR (101 MHz, $CDCl_3$)

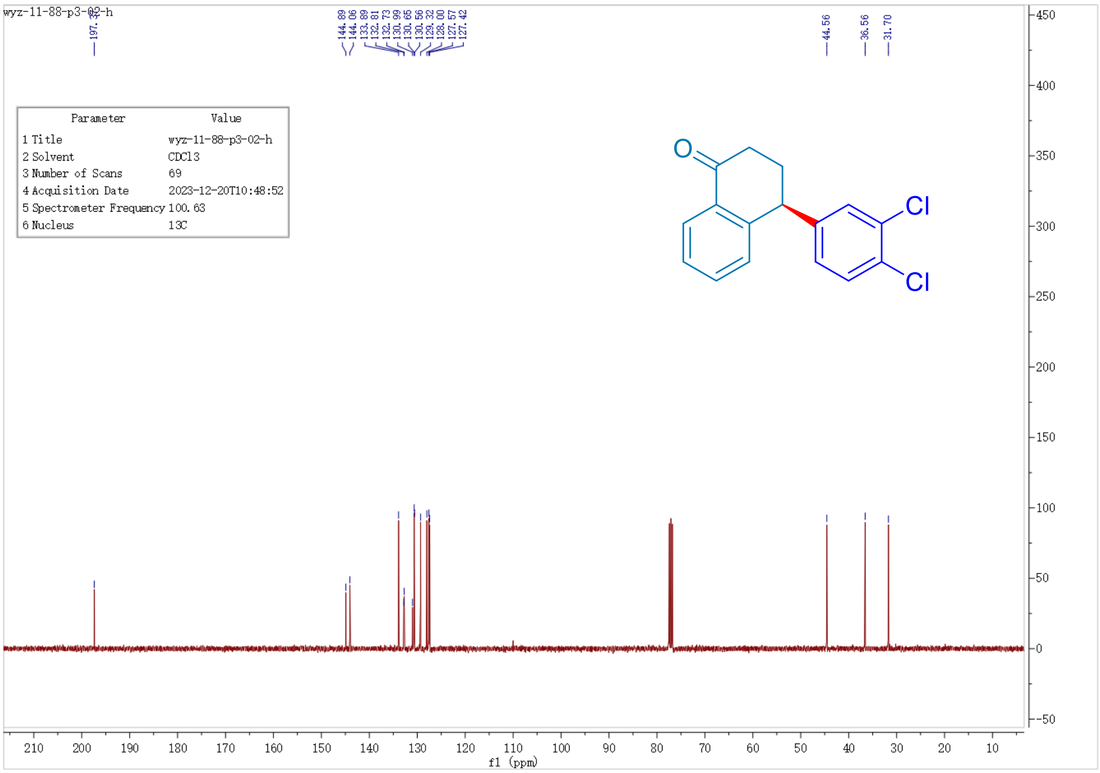

Compound 3aq ^{1}H NMR (400 MHz, $CDCl_3$)

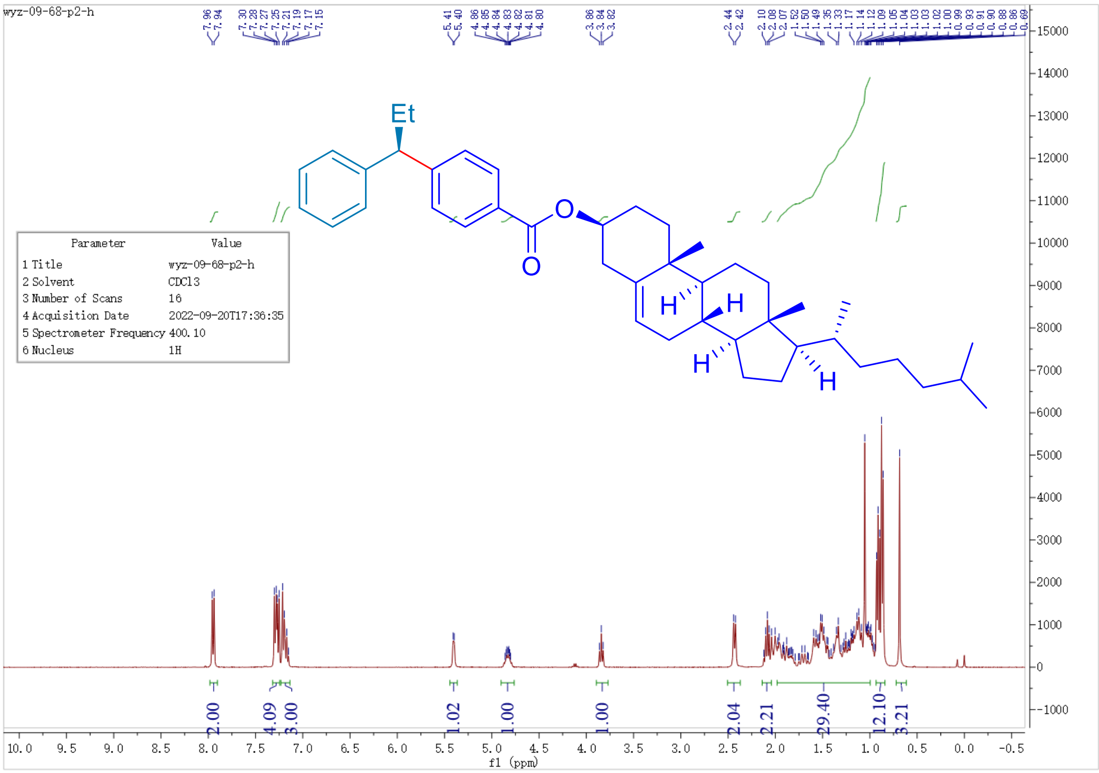

Compound 3aq ^{13}C NMR (101 MHz, $CDCl_3$)

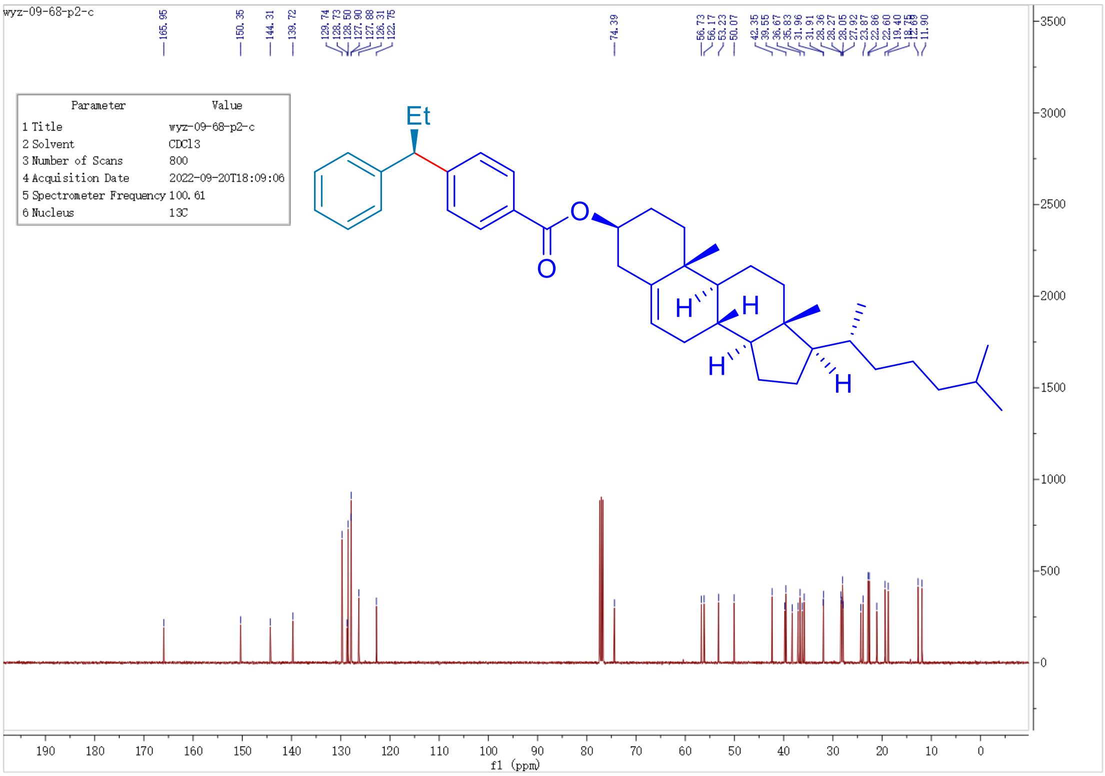

Compound 3ar ^{1}H NMR (400 MHz, $CDCl_3$)

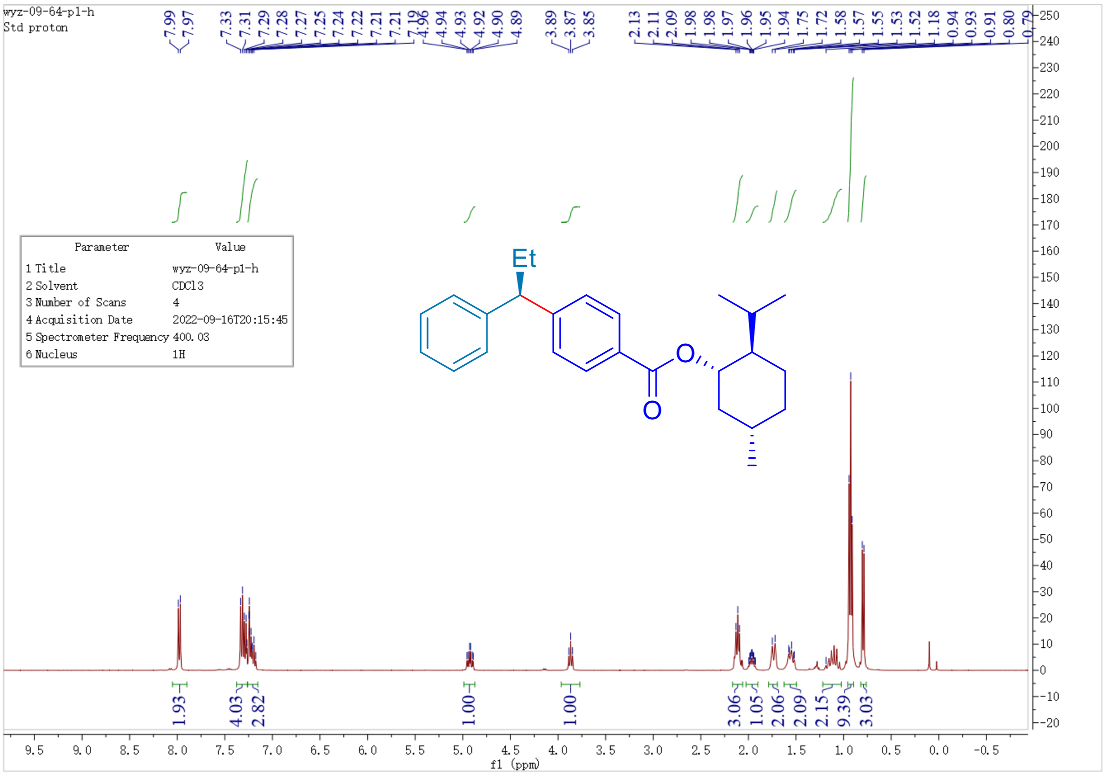

Compound 3ar ^{13}C NMR (101 MHz, $CDCl_3$)

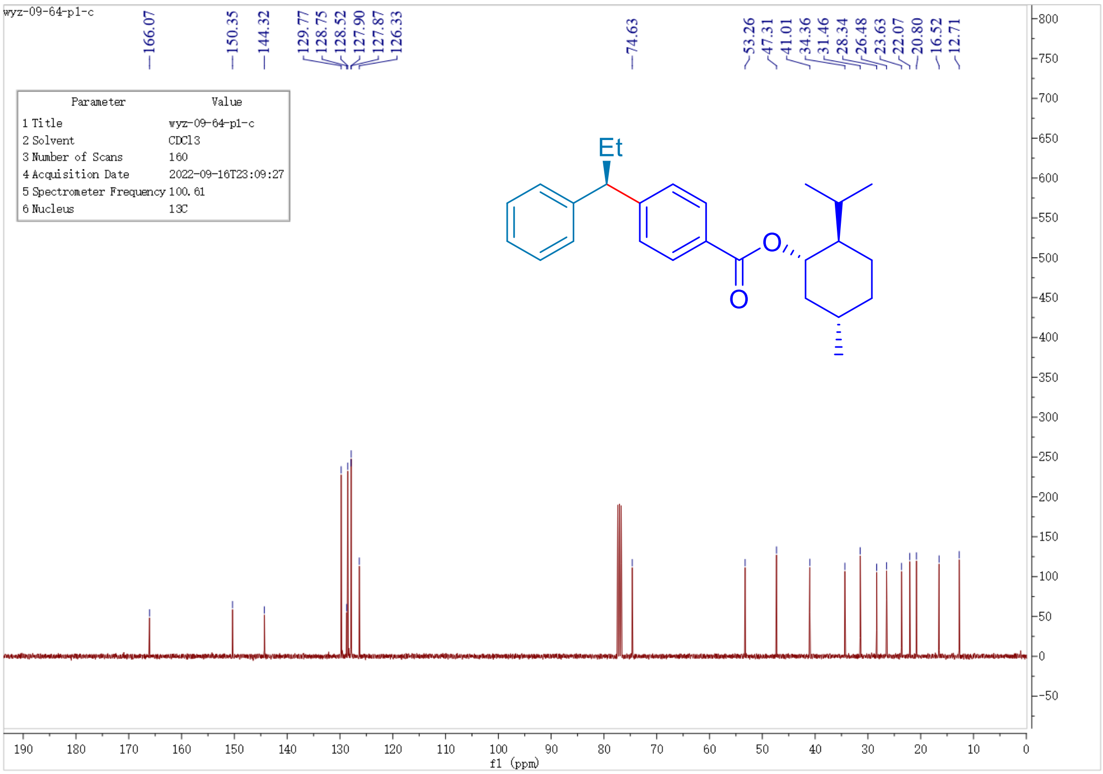

Compound 3as ^{1}H NMR (400 MHz, $CDCl_3$)

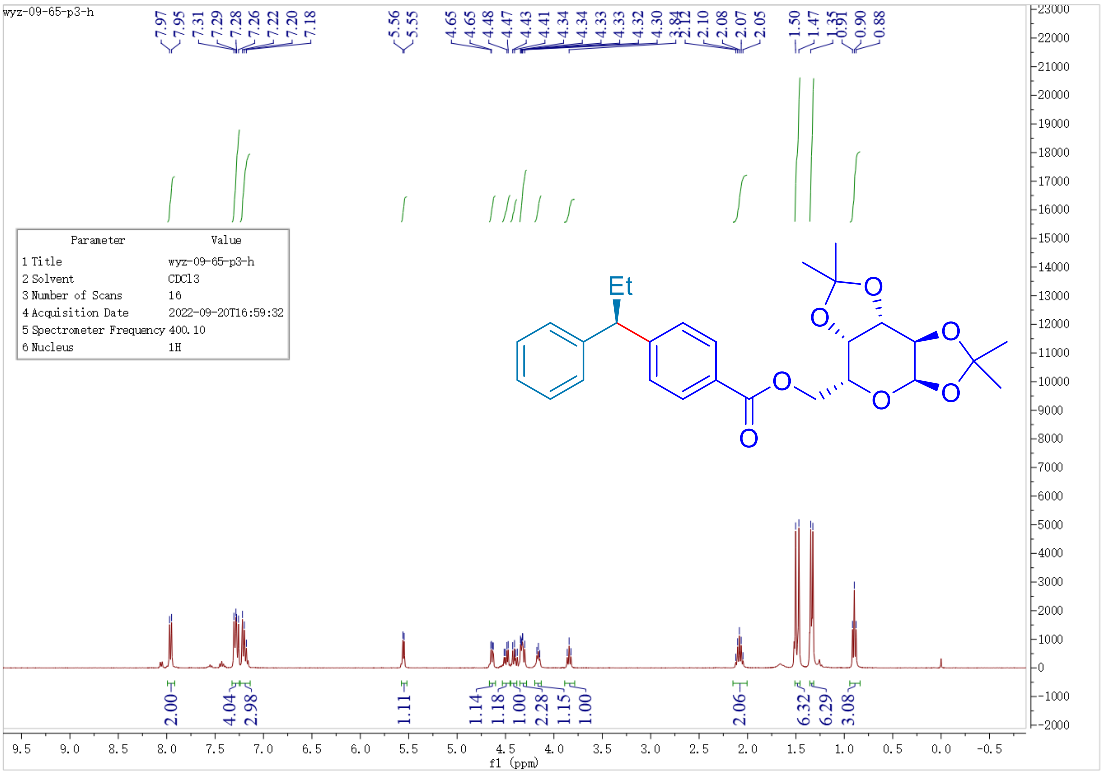

Compound 3as ^{13}C NMR (101 MHz, $CDCl_3$)

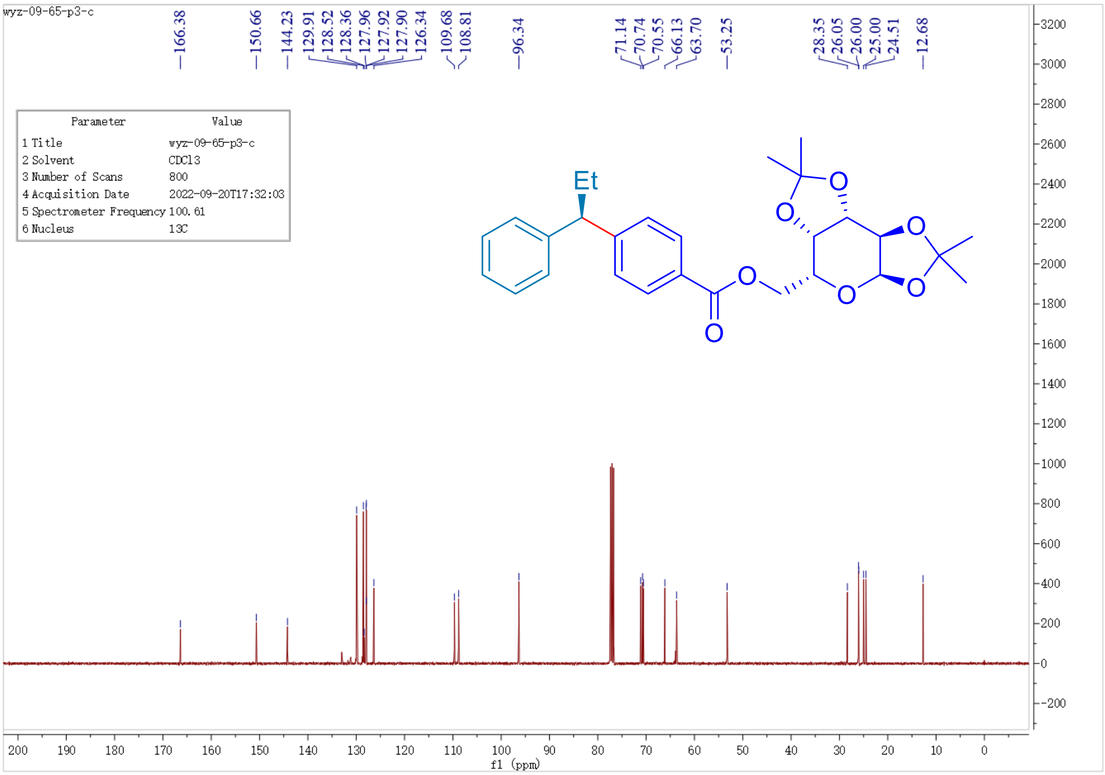

4. References

1. Y. He, C. Liu, L. Yu, S. Zhu, *Angew. Chem.* **2020**, *132*, 21714.
2. K. E. Poremba, N. T. Kadunce, N. Suzuki, A. H. Cherney, S. E. Reisman, *J. Am. Chem. Soc.* **2017**, *139*, 5684.
3. H. Yue, C. Zhu, L. Shen, Q. Geng, K. J. Hock, T. Yuan, L. Cavallo, M. Rueping, *Chem. Sci*. **2019**, *10*, 4430.
4. Q. Zhou, H. D. Srinivas, S. Dasgupta, M. P. Watson, *J. Am. Chem. Soc.* **2013**, *135*, 3307.
5. L. Peng, Z. Li, G. Yin, *Org. Lett*. **2018**, *20*, 1880.
6. C. C. Tyrol, N. S. Yone, C. F. Gallin, J. A. Byers, *Chem. Commun*. **2020**, *56*, 14661.
7. Y.-G. Chen, B. Shuai, X.-T. Xu, Y.-Q. Li, Q.-L. Yang, H. Qiu, K. Zhang, P. Fang, T.-S. Mei, *J. Am. Chem. Soc.* **2019**, *141*, 3395.
8. Chen, W. W.; Fernández, N. P.; Baranda, M. D.; Cunillera, A.; Rodríguez, L. G.; Shafir, A.; Cuenca, A. B, *Chem. Sci.* **2021**, *12*, 10514.
9. Ohmiya, H.; Makida, Y.; Li, D.; Tanabe, M.; Sawamura, M, *J. Am. Chem. Soc.* **2010**, *132*, 879.
